# Metallothionein-3 promotes cisplatin chemoresistance remodelling in neuroblastoma

**DOI:** 10.1038/s41598-021-84185-x

**Published:** 2021-03-09

**Authors:** Miguel Angel Merlos Rodrigo, Hana Michalkova, Vladislav Strmiska, Berta Casar, Piero Crespo, Vivian de los Rios, J. Ignacio Casal, Yazan Haddad, Roman Guran, Tomas Eckschlager, Petra Pokorna, Zbynek Heger, Vojtech Adam

**Affiliations:** 1 Department of Chemistry and Faculty of AgriSciences, Mendel University in Brno, Zemedelska 1, 613 00 Brno, Czech Republic; 2 Central European Institute of Technology, Brno University of Technology, Technicka 3058/10, 616 00 Brno, Czech Republic; 3 Instituto de Biomedicina Y Biotecnología de Cantabria (IBBTEC), Consejo Superior de Investigaciones Científicas (CSIC), Universidad de Cantabria, 39011 Santander, Spain; 4 Functional Proteomics, Department of Cellular and Molecular Medicine and Proteomic Facility, Centro de Investigaciones Biológicas (CIB-CSIC), Ramiro de Maeztu 9, 28040 Madrid, Spain; 5 Department of Paediatric Haematology and Oncology, Charles University and University Hospital Motol, V Uvalu 84/1, 150 06 Prague 5, Czech Republic; 6 Department of Oncology, Charles University and University Hospital Motol, V Uvalu 84/1, 150 06 Prague 5, Czech Republic

**Keywords:** Biochemistry, Cancer

## Abstract

Metallothionein-3 has poorly characterized functions in neuroblastoma. Cisplatin-based chemotherapy is a major regimen to treat neuroblastoma, but its clinical efficacy is limited by chemoresistance. We investigated the impact of human metallothionein-3 (hMT3) up-regulation in neuroblastoma cells and the mechanisms underlying the cisplatin-resistance. We confirmed the cisplatin-metallothionein complex formation using mass spectrometry. Overexpression of hMT3 decreased the sensitivity of neuroblastoma UKF-NB-4 cells to cisplatin. We report, for the first time, cisplatin-sensitive human UKF-NB-4 cells remodelled into cisplatin-resistant cells via high and constitutive hMT3 expression in an in vivo model using chick chorioallantoic membrane assay. Comparative proteomic analysis demonstrated that several biological pathways related to apoptosis, transport, proteasome, and cellular stress were involved in cisplatin-resistance in hMT3 overexpressing UKF-NB-4 cells. Overall, our data confirmed that up-regulation of hMT3 positively correlated with increased cisplatin-chemoresistance in neuroblastoma, and a high level of hMT3 could be one of the causes of frequent tumour relapses.

## Introduction

Metallothionein (MT) family is a class of low molecular mass, intracellular, and cysteine-rich proteins with a high affinity for metals^1,2^. MTs are involved in numerous cellular processes, such as binding and transport of zinc and copper ions, differentiation, proliferation, and apoptosis, therefore contributing to carcinogenesis^3–5^. Human metallothionein-3 (hMT3) is predominantly expressed in the central nervous system^6^ and found in kidney, in addition to some types of cancers, including the urinary bladder, prostate, oesophagus, stomach, non-small cell lung, and breast malignancies^7–12^. However, the significance of hMT3 expression outside the central nervous system and its involvement in the carcinogenesis is unclear^13^.

Neuroblastoma (Nbl) is a paediatric malignancy that typically arises in early childhood. Nbl is derived from the developing sympathetic nervous system^14–16^. It is the most common cancer in infants and also one of the most common extracranial solid tumours in children younger than 5 years^17–20^. Cisplatin or cis-diamminedichloroplatinum (CDDP) is a platinum coordination compound, which is generally accepted as one of the most effective drugs used alone or in combination with other agents (cyclophosphamide, doxorubicin or etoposide) in the chemotherapy for Nbl^21^. CDDP is a cytotoxic drug that kills cancer cells by damaging DNA and inhibiting its synthesis^22,23^. Despite the high efficiency of CDDP, cancer cells frequently develop a CDDP-chemoresistance phenotype, which could be due to a wide spectrum of causes, including decreased blood flow into tumour mass, inhibited internalization of CDDP, exocytosis of CDDP, intracellular sequestration of CDDP, halted DNA repair or apoptosis-driving mechanisms, or a presence of quiescent and non-cycling cells (non-growing or non-dividing state for a long period of time)^24^. As shown in our previous transcriptomic study, up-regulation of hMT3 induced development of CDDP-chemoresistance in Nbl cells and significantly altered the expression of sets of genes involved in apoptosis and oncogene-induced senescence^25^. Metastasis is a multistep process, including invasion, intravasation, and extravasation, which finally leads to tumour growth at a secondary organ^26–28^. hMT3 and human metallothionein 2A (hMT2A) are frequently reported in invasive human tumours and have been linked to the metastasis in other cancers via induction of metalloproteinase expression^29–31^. However, the importance of hMT3 in the process of the metastasis of Nbl cells, as well as the molecular mechanisms underlying this process, is still not fully understood. Therefore, we carried out an array of experiments designed to understand the role of hMT3 in a complex multi-step process, i.e., carcinogenesis, metastasis, and susceptibility of this process to CDDP administration in the chick chorioallantoic membrane (CAM) assay. In addition, a deep proteomic survey was performed to provide comprehensive insight into the proteome landscape of Nbl cells with transiently up-regulated hMT3.

Overall, we provide the first evidence that up-regulation of hMT3 resulted in increased dissociation, invasion, and intravasation from the primary tumours to the veins. In addition, hMT3 profoundly impacted blood migration of Nbl cells and their extravasation to chicken organs. Moreover, we report that Nbl cells with high constitutive hMT3 expression displayed up-regulation of various proteins that belong to several cellular pathways, such as exocytosis and cellular responses to stress, which are important to escape from the cytotoxic effects of CDDP. In addition, our study provides a comprehensive list of approximately 83 proteins that can be further investigated as prognostic biomarkers for CDDP chemotherapy or as possible druggable targets. Among these proteins, cyclin dependent kinases (cell cycle/apoptosis pathway), taxilin/cathepsins/tubulins (vesicle transport pathways), active transport membrane proteins, proteasome subunits, heat shock proteins (HSP), and other reactive oxygen species (ROS) stress response proteins can be considered.

## Results

### Basal expressions of hMT3 and effect of its up-regulation on susceptibility to CDDP

**Figure 1:**
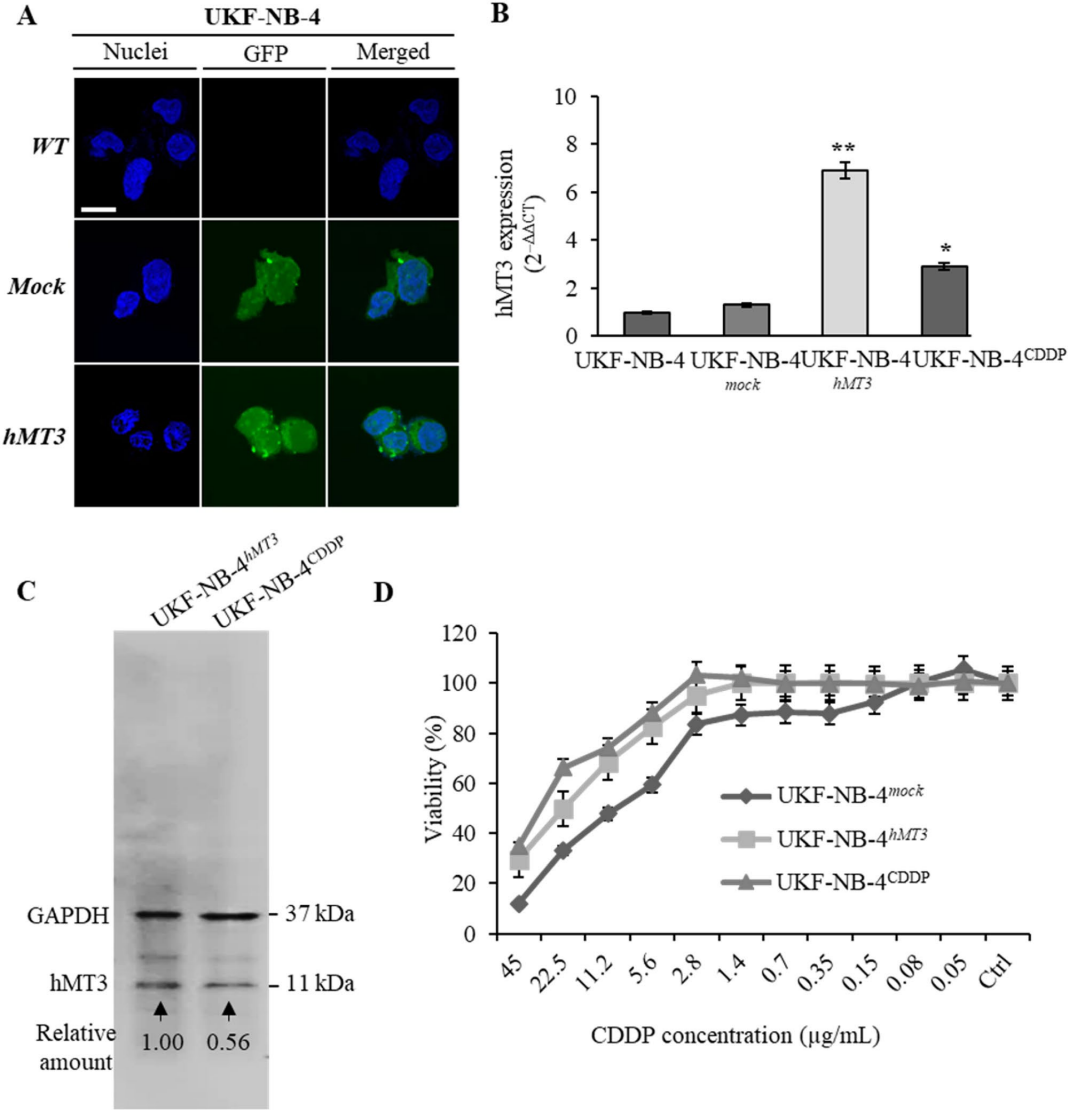
Comparison of WT, mock and hMT3 in UKF-NB-4 neuroblastoma cells. The cells were transfected with either pcDNA3.1-GFP-TOPO (mock transfection) or pcDNA3.1-GFP-hMT3-TOPO (hMT3). (**A**) Transfection was validated by confocal microscopy through fluorescence of expressed GFP. The length of scale bar is 10 µm. (**B**) qRT-PCR showing changes in mRNA encoding hMT3 upon transfection. Data were analysed by comparative CT method and presented as relative fold gene expression (2^–ΔΔCT^). (**C**) Whole-cell lysates immunoblots showing higher expression of hMT3 in UKF-NB-4^hMT3^ compared to UKF-NB-4^CDDP^ cells. GAPDH, loading control. The number below the bands show values obtained by densitometric analysis of bands. (**D**) Percentages of metabolically active cells after treatment with annotated concentrations of CDDP. **p < 0.001, *p < 0.05. WT, wild-type.

The efficiency of transfection analysed through green fluorescence protein (GFP) tagged at the *N*-terminus of hMT3 using our optimized transfection protocol^25^ resulted in approx. 70% transfection efficiency for both constructed plasmids (*mock* and hMT3-transfected cells). Transfection was validated by fluorescence confocal microscopy (Fig. 1A). Furthermore, the qRT-PCR (Fig. 1B) confirmed a significant (*p* < 0.001) increase in the expression of hMT3 upon transfection with hMT3 vector. Of note, chemoresistant UKF-NB-4^CDDP^ cells exhibited significantly (*p* < 0.05) higher basal expression compared to sensitive UKF-NB-4 cells. Using the 2^–ΔΔCT^ method, the transfection with *hMT3* vector resulted in approx. sixfold higher relative expression of *hMT3* compared to wild-type cells and threefold higher expression of *hMT3* compared to UKF-NB-4^CDDP^ cells. In addition, at the protein level, it was confirmed that UKF-NB-4^hMT3^ cells exhibit higher expression of hMT3 compared to the basal expression of hMT3 in UKF-NB-4^CDDP^ cells (Fig. 1C). In order to verify the acquired chemoresistance to CDDP upon transfection, we further investigated the susceptibility of Nbl cells to different concentrations of CDDP. Up-regulation of hMT3 influenced the sensitivity of UKF-NB-4 cells to CDDP (Fig. 1D).

### CDDP inhibits metastasis in UKF-NB-4 cells but not UKF-NB-4^CDDP^

**Figure 2:**
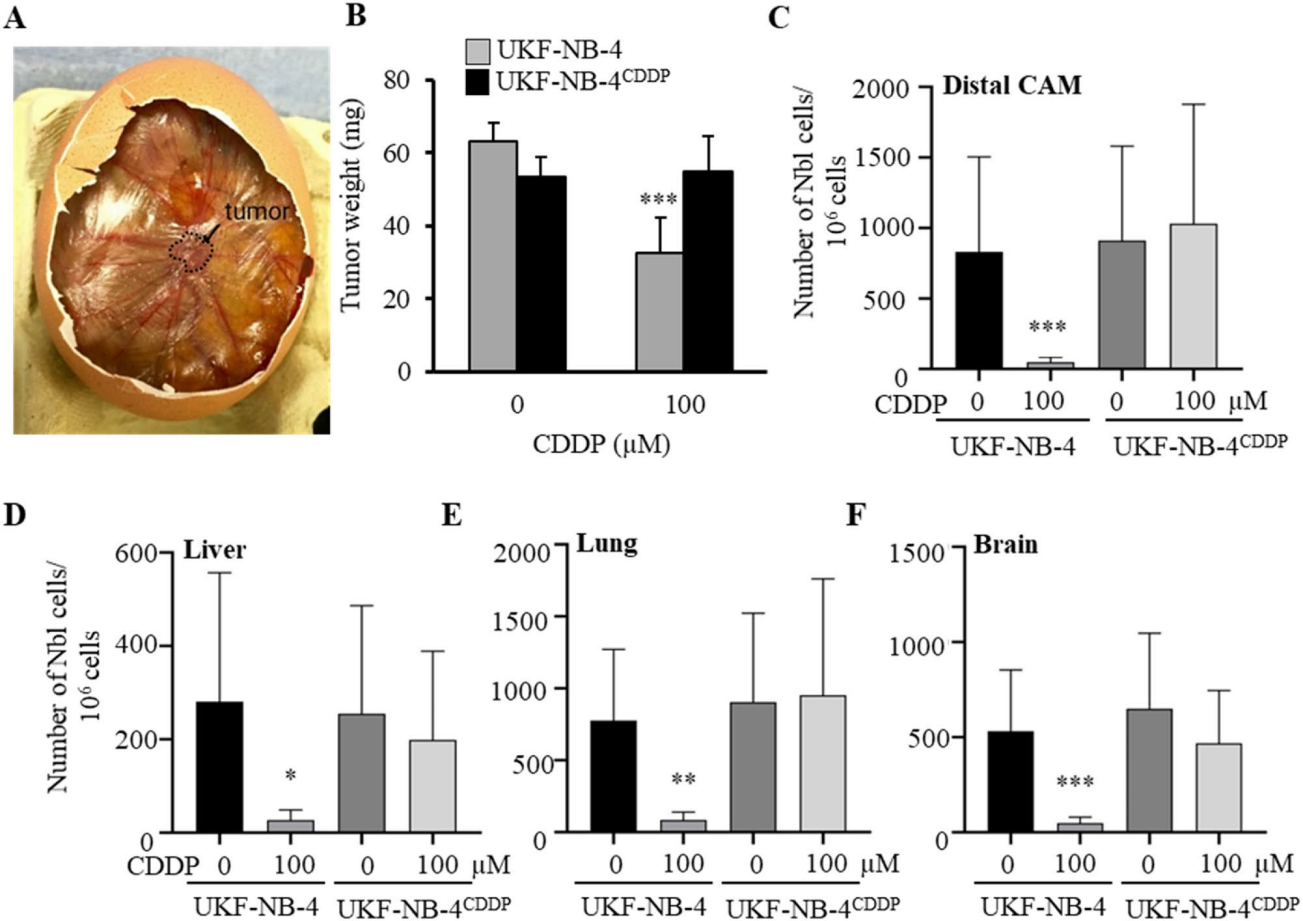
Evaluation of efficiency of CDDP to inhibit tumour growth and metastatic spread of UKF-NB-4 and UKF-NB-4^CDDP^ cells in CAM assay. (**A**) Representative CAM with the tumor formed 7^th^ day after induction. (**B**) Weights of tumors excised from the CAM upon experiment termination (17^th^ day). Numbers of Nbl cells identified in (**C**) distal CAM (extravasation), (**D**) liver, (**E**) lung and (**F**) brain (intravasation). Quantitation of Nbl cells was based on qPCR using Alu primers. In addition, qPCR of chicken GAPDH was performed as an internal control to confirm the presence of equivalent quantities of host genomic DNA. Data shows average ± SEM from three (*n* = 3) independent experiments. *p < 0.05; **p < 0.005, ***p < 0.001.

We employed CAM assay to study whether CDDP had any effect on intravasation, extravasation, and colonisation of Nbl cells in vivo. Within 7 days from induction, 100% (20/20) of eggs survived (Fig. 2A). Upon termination, tumour growth was assessed through weight measurements as well as total Nbl cell counts in the different organs [distal CAM (extravasation), liver, lung, and brain (intravasation)] of the embryos. UKF-NB-4 xenografts treated with 100 µM CDDP exhibited significant inhibition (approx. two-fold) of the weight of primary tumour in the CAM (Fig. 2B). In addition, the number of UKF-NB-4 cells in distal CAM, liver, lung, and brain decreased significantly (Fig. 2C–F), compared to the untreated controls. By contrast, in UKF-NB-4^CDDP^ cells, CDDP did not exhibit any significant inhibitory effect on tumour weight or metastatic spread to tested organs. The obtained results confirmed the chemoresistance of UKF-NB-4^CDDP^ cells and highlighted the failure of CDDP to inhibit intravasation and extravasation of Nbl cells in CAM assay.

### Impact of overexpression of hMT3 on CDDP-chemoresistance and metastasis progression by chick chorioallantoic membrane assay (CAM)

**Figure 3:**
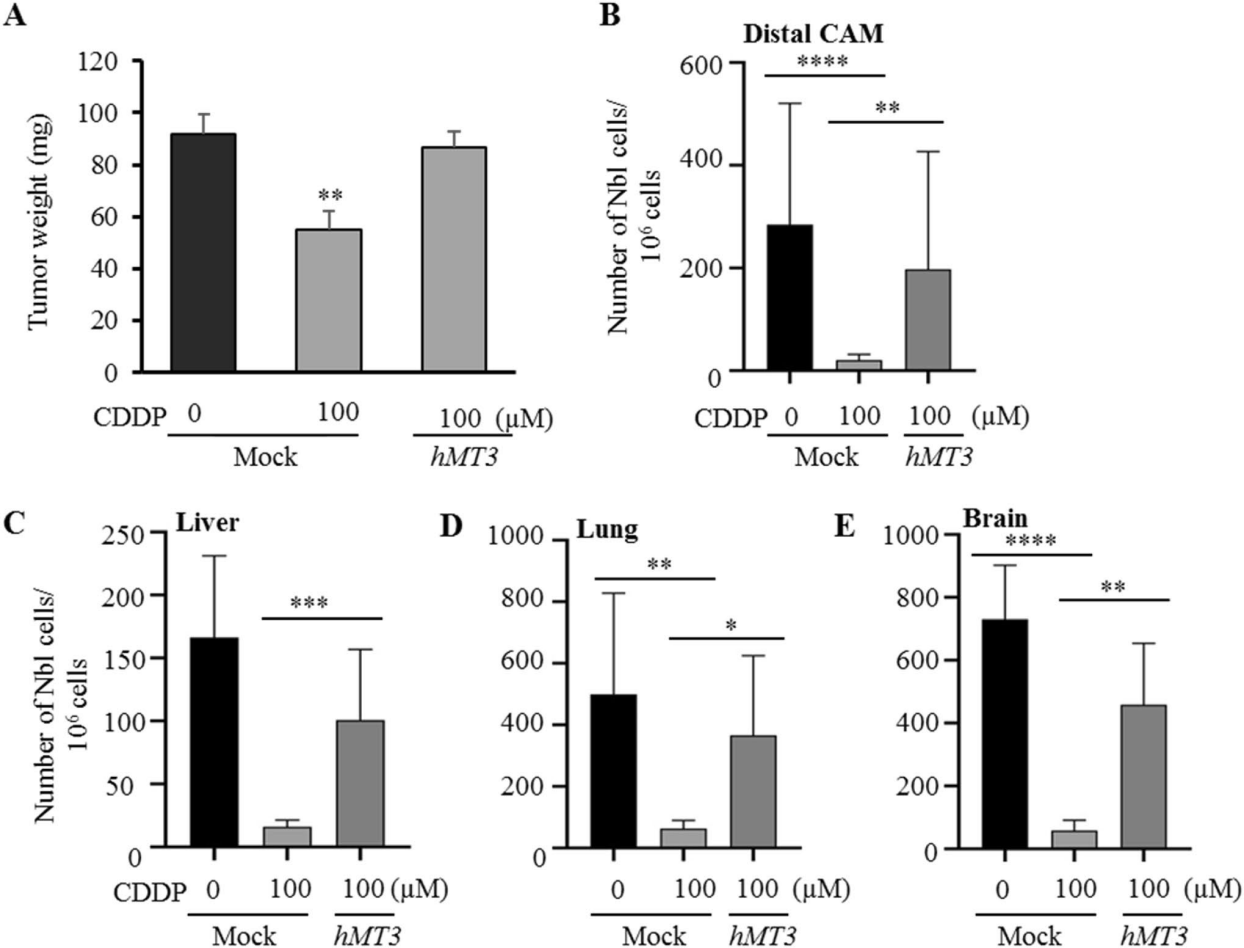
Evaluation of efficiency of CDDP to inhibit tumour growth and metastatic spread in UKF-NB-4^mock^ cells and their hMT3 counterparts. (**A**) Weights of tumours excised from the CAM upon experiment termination (17^th^ day). Numbers of Nbl cells identified in (**B**) distal CAM (extravasation), (**C**) liver, (**D**) lung and (**E**) brain (intravasation). Quantitation of Nbl cells was based on qPCR using Alu primers. In addition, qPCR of chicken GAPDH was performed as an internal control to confirm the presence of equivalent quantities of host genomic DNA. Data shows average ± SEM from three (n = 3) independent experiments. *p < 0.05; **p < 0.005, ***p < 0.001, ****p < 0.0001.

After validation of the reliability of the CAM assay for study of CDDP efficiency on the spread of Nbl cells, we continued with the investigation of an effect of transient up-regulation of hMT3 in UKF-NB-4 cells on susceptibility to CDDP. Importantly, we confirmed the stability of transient transfection during the whole duration of the CAM assay (Supplementary Fig. 1A). The obtained data show that up-regulation of hMT3 resulted in a development of marked chemoresistance to CDDP (Fig. 3A). Tumour weights in mock and hMT3 cells reached similar weights (91.6 mg vs. 86.7 mg, respectively). Interestingly, we provide the first evidence that hMT3 up-regulation promotes improved capacities of intravasation (Fig. 3B) and extravasation to organs (Fig. 3C–E). Importantly, these capacities were only negligibly inhibited by CDDP administration. Taken together, our results suggest that hMT3 could act as a highly efficient inducer of chemoresistance to CDDP.

### MT directly interacts with CDDP in vitro

**Figure 4:**
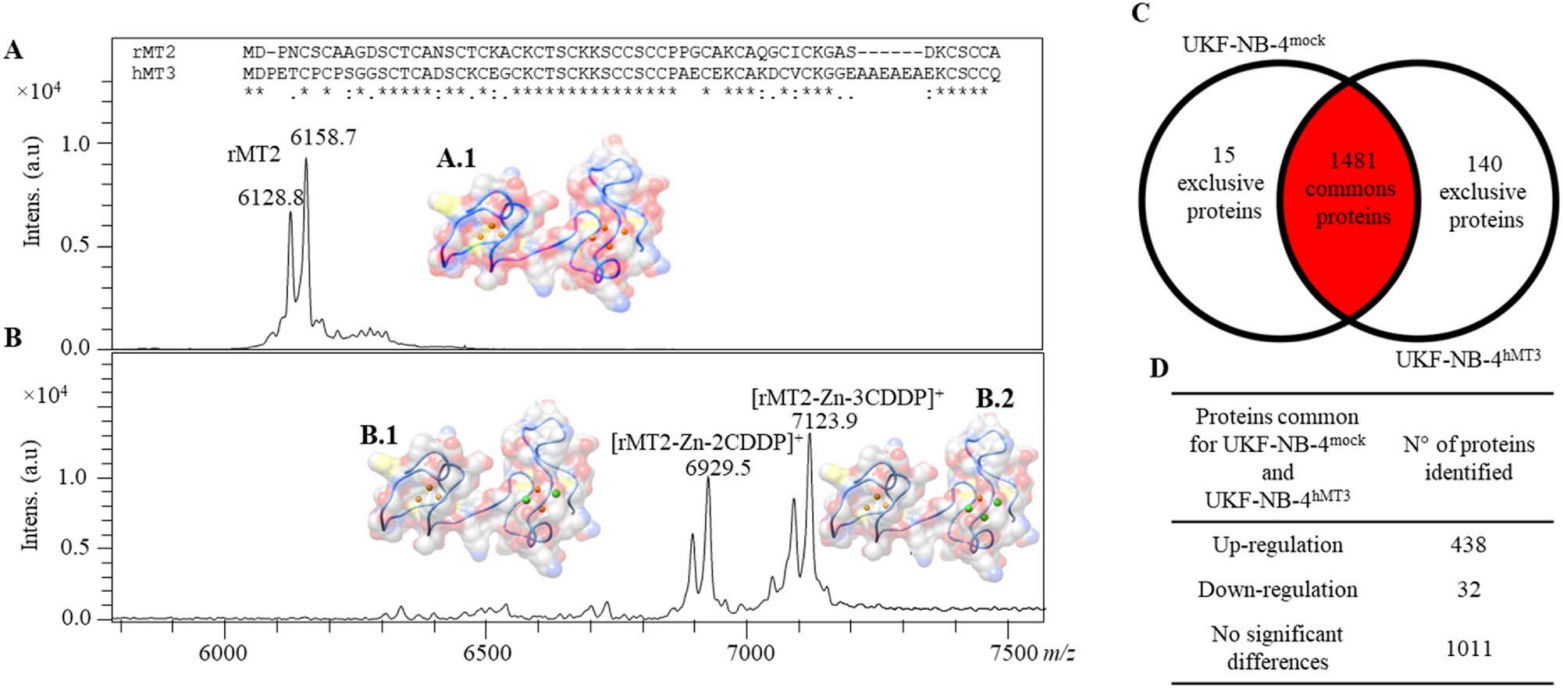
MALDI-TOF mass spectra of rMT2-CDDP complex and purified rMT2 standard. Upper insert shows CLUSTAL multiple sequence alignment of rabbit rMT2 and hMT3. Amino acid from the sequences with asterisk (*) indicate 100% of similarity between rMT2 and hMT3. (**A**) MALDI-TOF spectra of rMT2 and (**B**) complex between rMT2 and CDDP after 24 h incubation in PBS (pH 7.5). Each spectrum was averaged from 2000 subspectra. In each mass spectrum, the hypothetical models of hMT3 complexed with Zn and Pt are shown. hMT3 protein sequence (P25713) was used to build a homology model based on template structure of rat MT2 (PDB ID 4MT2) via SwissModel server. Assuming that superior electronegativity of Pt ions replaced Zn in a similar fashion to Cd ions, the 3D structures of hMT3 Pt/Zn complexes are shown here. (**A.1**) Zn7-MT3 and (**B.1**) Pt2Zn5-hMT3 and (B.2) Pt3Zn4-hMT3. hMT3 is shown in blue ribbon and molecular surface HETATOM colours. Zn and Pt ions are shown in orange and green, respectably. (**C**) Venn diagram showing exclusiveness of proteins identified in proteomic signatures of parental UKF-NB-4 and UKF-NB-4 cells with up-regulated hMT3. (**D**) Table showing the numbers of 1481common proteins and the distribution of their regulation (UKF-NB-4-MT3 vs. UKF-NB-4, up-regulation-fold ratio > 2.5; down-regulation-fold ratio < 0.5; 2.5 > no significant differences > 0.5).

In order to investigate the nature of possible interactions between hMT3 and CDDP, we utilized commercial rabbit MT2 (rMT2, 98% purity), exhibiting high structural similarity to hMT3 (nearly 60% sequence identity, and approx. 0.749 Å root-mean-square deviations for 61 paired-atoms in the homology 3D structures of hMT3 and rMT2 retrieved from SwissModel server—https://swissmodel.expasy.org/). Upon incubation of rMT2 with CDDP, a high rate of complexation was identified by mass spectrometry. The theoretical molecular mass of rMT2 is 6012.10 Da (without Zn). The main observed signals of rMT2 were assigned as follows: [rMT2 + 2Zn]^+^ (*m/z* 6128.8 Da and 61,587 Da) (Fig. 4A). Upon incubation of rMT2 with CDDP, new signals corresponding to complexes [rMT2 + 2CDDP]^+^ (*m/z* 6929.5 Da) and [rMT2 + 3CDDP]^+^ (*m/z* 7123.9 Da) were identified (Fig. 4B). Therefore, it was evident that an intramolecular complex, rMT2-CDDP, was formed. Furthermore, the peak of [rMT2 + 2Zn]^+^ disappeared after incubation with CDDP. This highlighted a high affinity of rMT2 to the CDDP in vitro and the capability of CDDP to remove Zn ions from rMT2 structure. To further elucidate the mode of interaction between hMT3 and CDDP, hMT3 protein sequence (P25713) was used to build a homology model based on template structure of rat MT2 (PDB ID 4MT2). Two pathway models were proposed to explain the binding of Zn/Cd ions to MT, either by cooperative or non-cooperative binding^32^. Experimental evidence showed that the first four Cd metallic ions that replaced Zn ions were all in the alpha domain; however, it was also possible that several intermediate states occurred that could involve the β domain^33,34^. Assuming that the superior electronegativity of Pt ions would replace Zn in a similar fashion to Cd ions, the 3D structures of MT3-Pt/Zn complexes could be equivalent to those containing Cd ions, as shown in Fig. 4.

### hMT3 up-regulation in UKF-NB-4 cells results in widely altered proteomic profile

In order to investigate the quantitative differences in the protein expression patterns resulting from hMT3 up-regulation, the comparative proteomic profiles of UKF-NB-4 cells (hMT3 *vs.* mock) were analysed. Two independent experiments were conducted following the same workflow. The universally accepted requirement is to have identified the proteins with the least two peptides (Σ Unique Peptides). Out of a total 1636 identified proteins, 1481 were commonly expressed in hMT3 *vs.* mock (Fig. 4C). A full list of proteins as well as relevant MS data are all available in Supplementary Table 1. From the total common proteins in dataset (hMT3 *vs.* mock), 438, 32, and 1011 proteins were quantitatively up-regulated (fold ratio > 1.5), down-regulated (fold ratio < 0.5), and showed no significant differences (fold ratio 0.5−1.5, Fig. 4D), respectively (Supplementary Table 2). A total of 140 significant protein expressions were exclusively registered in hMT3 overexpressing UK-NB-4 cells (full list of proteins is shown in Supplementary Table 3). qRT-PCR validation of expression of mRNA encoding selected proteins that were found deregulated by proteomic analysis is shown in Supplementary Fig. 1B.

### hMT3 up-regulation impacts vesicle transport, cellular response to stress, and other pathways

**Figure 5:**
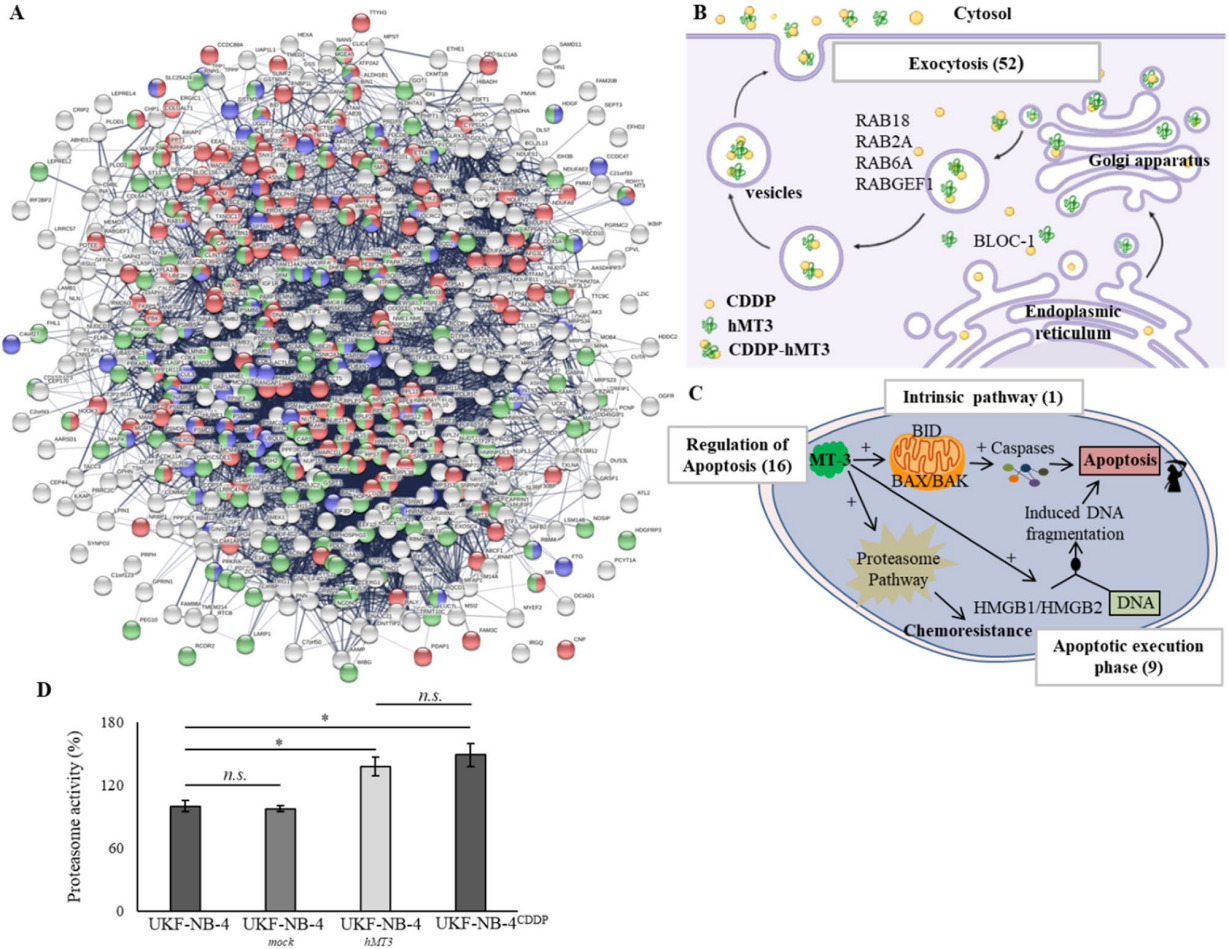
(**A**) STRING interactome network showing the proteins, which were found exclusively expressed and up-regulated in UKF-NB-4^hMT3^ cells compared to mock cell line. These are involved in transport (red nodes), cellular response to stress (blue nodes) and negative regulation of biological process (green nodes), respectively. The colour of the line provides evidences of the different interactions among proteins. A red line indicates the presence of fusion evidence; a green line, neighbourhood evidence; a blue line, concurrence evidence; a purple line, experimental evidence; a light blue line, database evidence; a black line, co-expression evidence. Schematic description of (**B**) vesicles-mediated transport and membrane trafficking, and (**C**) apoptotic pathways. The numbers in brackets indicate amount of proteins, which were identified exclusively deregulated due to hMT3 overexpression in UKF-NB-4 cells compared to mock cells. RAB: member RAS oncogene GTPases, BLOC-1: Biogenesis Of Lysosomal Organelles Complex 1, BID: BH3 Interacting Domain Death Agonist, BAX: BCL2 Associated X, BAK: BCL2 Antagonist/Killer, and HMGB1/2: High Mobility Group Box). (**D**) Proteasome activity analysed in parental UKF-NB-4 cells compared to UKF-NB-4^mock^, UKF-NB-4^hMT3^ and UKF-NB-4^CDDP^ cells. The data are results from three (*n* = 3) independent experiments. *p < 0.01, n.s., not significant.

To classify categories of commonly and exclusively expressed proteins according to canonical pathways, we performed gene ontology (GO) enrichment analysis using the Database for Annotation, Visualization, and Integrated Discovery (DAVID)^35^ and Kyoto Encyclopaedia of Genes and Genomes (KEGG)^36^ using Protein–Protein Interaction Networks Functional Enrichment Analysis (STRING) software. A list of up- and down-regulated proteins in hMT3 overexpressed and exclusive in UK-NB-4 cells is described in Supplementary Tables 2 and 3. A list of processes and/or pathways involved in protein regulation in UK-NB-4 cells (hMT3 *vs*. mock) is shown in Supplementary Table 4. Bioinformatics analyses revealed up-regulation of a number of proteins affecting biological pathways related to transport, cellular responses to stress, and negative regulation of biological process (Fig. 5A). Figure 5B schematizes the action of proteins involved in membrane trafficking and exocytosis. The vesicles can transport CDDP outside the cells through exocytosis to reduce intracellular drugs concentration. We also identified a long list of proteins involved in this process—up-regulated due to hMT3 overexpression—(A2M, ACTN1, ALDOA, ALDOC, BLOC1S6, CAB39, CHP1, CPNE1, CTSA, CTSB, CTSC, DDOST, DIAPH1, DYNC1LI1, FAM3C, FTL, GGH, HMGB1, HMOX2, HUWE1, IST1, LAMP2, LAMTOR1, MAGED2, MANF, NCSTN and NME1). In addition, we confirmed that up-regulation of hMT3 could have profound effects on regulation of proteins involved in the apoptotic pathway (Fig. 5C). In particular, we identified a number of proteins associated with proteasome function (PAK2, PSMA2, PSMA3, PSMA5, PSMB1, PSMB2, PSMB3, PSMB5, PSMB6, PSMB7, PSMC2, PSMC3, PSMD7, PSMD9 and PSMD13), which is frequently responsible for chemoresistance in a wide spectrum of cancers^37^. Further, the stimulatory effect of hMT3 on activity of proteasome complex was successfully validated (Fig. 5D). Interestingly, the UKF-NB-4h^MT3^ cells exhibited a similar proteasome complex activity as UKF-NB-4^CDDP^ cells undoubtedly highlighting the importance of interplay between hMT3 and proteasome complex for chemoresistance of Nbl to CDDP.

### Cells with up-regulated hMT3 exhibit similar proteome to cells with acquired chemoresistance to CDDP

**Figure 6:**
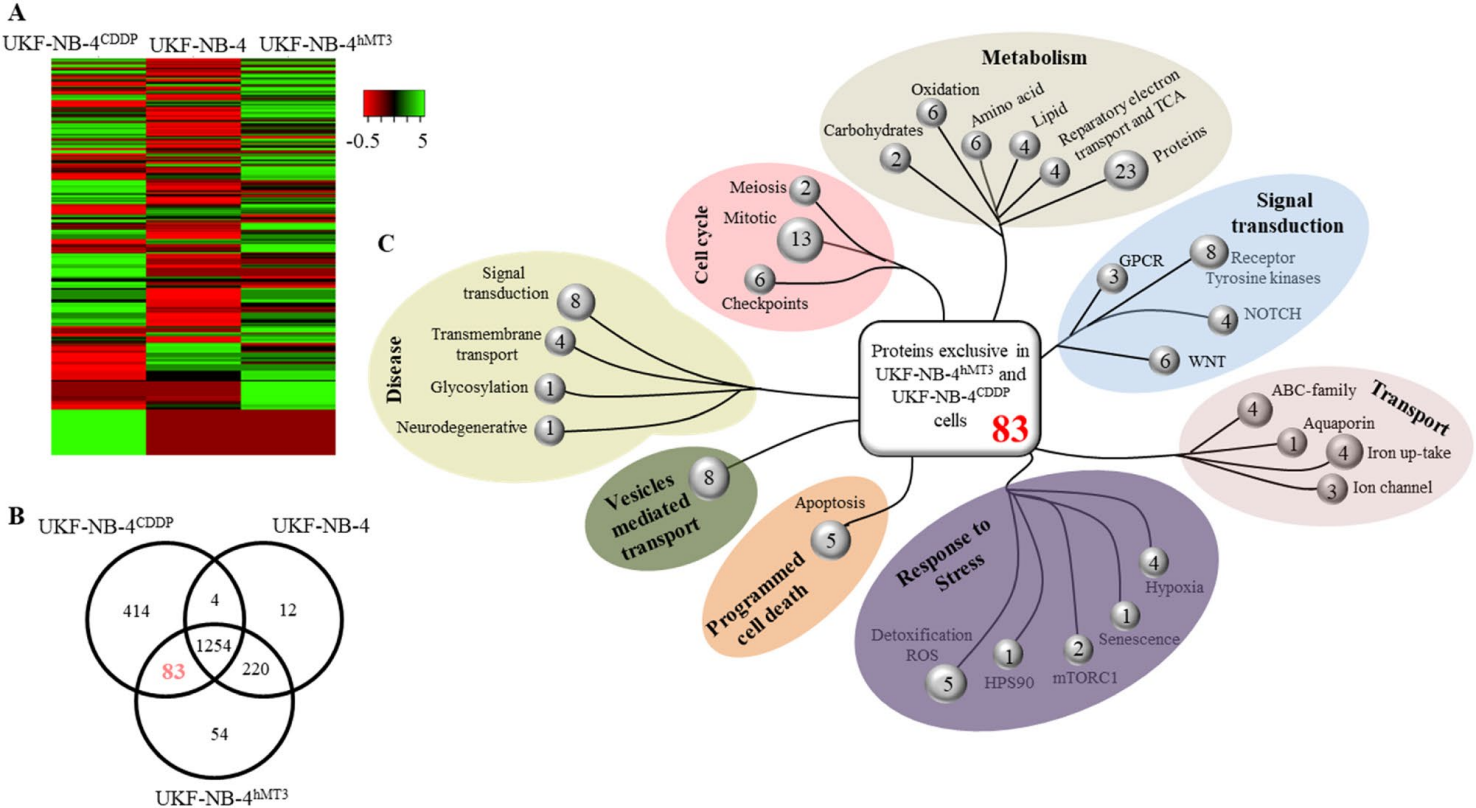
(**A**) Expression-based heatmap of total proteins identified in UKF-NB-4, hMT3 overexpressing UKF-NB-4 and UKF-NB-4^CDDP^ cells. (**B**) Venn diagram showing exclusiveness and commonness of proteins identified in proteomic signatures of parental UKF-NB-4, UKF-NB-4^hMT3^ and UKF-NB-4^CDDP^ cells. (**C**) Classification summary of pathways of 83 commons proteins identified in UKF-NB-4^CDDP^ and UKF-NB-4^hMT3^ cells, as predicted by DAVID and Reactome software.

The proteomic profiles of hMT3 overexpressing cells were further compared to proteomes of parental UKF-NB-4 and chemoresistant UKF-NB-4^CDDP^ cells (list of proteins, along with their accession numbers, is shown in Supplementary Table 5). The differential expression of proteins between analysed cell lines was visualized using an expression-based heatmap depicted in Fig. 6A. Interestingly, the heatmap displayed relatively high rate of similarities between UKF-NB-4^CDDP^ and hMT3 overexpressing UKF-NB-4 cells, while parental UKF-NB-4 cells shared only a negligible portion of similarities.

We note that, out of all proteins identified in hMT3 overexpressing UKF-NB-4, UKF-NB-4 and UKF-NB-4^CDDP^ cell lines, 83 were exclusively expressed in both UKF-NB-4-hMT3 and UKF-NB-4^CDDP^ cells (Fig. 6B). Bioinformatics analyses revealed that up-regulated proteins were mostly involved in the biological pathways related to transport, cellular responses to stress, and metabolism among others (Fig. 6C).

To further classify categories of proteins commonly expressed in UKF-NB-4-hMT3 and UKF-NB-4^CDDP^ cells according to canonical pathways affected by these proteins, we performed GO enrichment analysis using DAVID and KEGG. The top up-regulation scoring was, among others, for protein transport, response to chemicals, response to stress, vesicle-mediated transport, response to oxidative stress and metal ions, and regulation of apoptotic process (Supplementary Fig. 2A). Analysis of canonical pathways revealed some of the most significantly up-regulated functions involving metabolic pathway, ribosome, oxidative phosphorylation, proteasome, drug metabolism and glutathione metabolism (Supplementary Fig. 2B).

## Discussion

The main causes of death in patients with Nbl include metastasis (present in up to 70% of patients at the time of diagnosis) and tumour resistance to conventional treatment^38^. In the development of metastasis, cancer cells must successfully break through a multistep process, including invasion, intravasation, extravasation, and colonisation into other organs. In the current study, we developed a CAM xenograft tumour model protocol using a CDDP-sensitive and resistant Nbl cell line for monitoring CDDP drug influence on Nbl cell invasion in vivo. The CAM assay is a frequently applied model to study cancer metastasis^39–42^. However, there are only a few studies that have used CAM assays to assess Nbl cell invasion and metastasis^43–45^. The UKF-NB-4^CDDP^ cell line displayed high resistance to CDDP and showed higher intravasation and metastasis in liver, lung, and brain than the parental cell line. This phenomenon might be due to CDDP action on several signalling pathways, thereby triggering resistance mechanisms by establishing a complicated self-defence system to escape exogenous cytotoxic compounds of different origins^38^. By contrast, MTs have been implicated in the metastasis in several types of cancers^29,30^, with evidence that MT3 regulated the genes involved in metastasis^25,46^. In this study, we elucidated the potential mechanisms of hMT3-mediated induction of Nbl invasion and CDDP-chemoresistance in a CAM xenograft tumour model. hMT3 was revealed as an important driver in the extravasation, migration, and intravasation of Nbl cells, which is consistent with the reported major regulatory role of MT in the cancer metastasis. However, how hMT3 mediates the resistance process remains unknown.

To address this question, a comparative proteomic study was carried out to identify proteins associated with CDDP resistance in human Nbl cells with hMT3 overexpression. This work provided deeper insights into the hMT3 complex mechanism in the acquired resistance. In the following discussion, we highlight the role of hMT3 in CDDP-resistance by interpretation of the proteomic data according to recognised involvements in cell cycle/apoptosis, vesicle-mediated transport, active membrane transport, proteasome, and response to stress (i.e., ROS) pathways.

It is important to point out that current studies on cancer cells demonstrate that the endogenous up-regulation of hMT3 could inhibit cell growth^47^. In previous experiments, we found that hMT3 cells reached full confluence, despite their chemoresistance to CDDP, much slower than mock in the SiMa Nbl cell line^25^. In this study, we show that hMT3 overexpressing the UKF-NB-4 cell line had reduced growth in a way which correlated with up-regulation of proteins related to senescence and apoptosis. Organisms with renewable tissues commonly have mechanisms that allow them to prevent the development of tumours. Cellular senescence and apoptosis (also known as programmed cell death) are among those mechanisms. Apoptosis plays a pivotal role in maintaining proper tissue homeostasis^48,49^. Our results showed that cell division cycle and apoptosis regulator 1 (CCAR1; Q8IX12) and cyclin-dependent kinase (CDK1; P06493) proteins were up-regulated in the hMT3 overexpressing UK-NB-4 cells. CCAR1 protein mediates apoptosis via interaction with 14–3–3 protein, up-regulation of CDK inhibitor p21^WAF1/CIP1^, and down-regulation of cell cycle regulatory genes *c-Myc* and *cyclin B1*^50^. Other up-regulated proteins include: apoptotic chromatin condensation inducer 1 protein (ACIN1; Q9UKV3; inducing apoptotic chromatin condensation after activation by caspase-3), cyclin dependent kinase 4 (CDK4; P11802), polyhomeotic homolog 2 (PHC2; Q8IXK0; associated with senescence pathway), BH3 interacting-domain death agonist (BID; P55957; pro-apoptotic member of the Bcl-2 protein family), and programmed cell death 10 (PDCD10; Q9BUL8; associated with cell apoptosis as target of C-MYC activation). Previous studies showed that BID expression was positively correlated with cell apoptosis in Nbl^51^. In contrast, over-expression of PDCD10 in parental HeLa cells was reported to increase doxorubicin-resistance while it re-sensitized doxorubicin-resistant MCF7 cells. These results showed a possible dual role of PDCD10 in drug resistance^52–54^.

Cellular inactivation of CDDP and subsequent sequestration could be mediated by MTs^55–59^. MTs chelate platinum ions, which prevents their interaction with DNA in Nbl cells, possibly through excretion of metal ions outside the cell. Indeed, proteomic identification by Orbitrap MS revealed higher expression of proteins within transport, exocytosis, and cellular response to oxidative stress pathways in the UKF-NB-4 cell line with overexpressed hMT3. Vesicle trafficking, including endocytosis and exocytosis, has been implicated in multidrug resistance of tumours, implicating vesicle shedding as a potential drug efflux mechanism^60^. We demonstrated that the Ras-related small GTPases (RAB) protein family was up-regulated in the UKF-NB-4 cell line with overexpressed hMT3 (Q9NP72, P61019, P20340 and Q9UJ41). The RAB proteins are important regulators of vesicular trafficking events and induce chemoresistance towards CDDP in various types of cancers^61,62^. In addition, we showed that α-taxilin (P40222) protein was up-regulated in the UKF-NB-4 cell line with overexpressed hMT3. Taxilin is involved in Ca^2+^-dependent exocytosis in neuroendocrine cells^63^. Its expression is correlated with growth activity and malignant potential of hepatocellular carcinoma^64^. It was previously shown that inhibiting lysosomal exocytosis reversed invasiveness and chemoresistance in aggressive sarcoma cells^65^, which highlights the possible role of taxilin as a key-player in MT3-mediated CDDP trafficking and resistance in Nbl cells.

It is important to note that many proteins that were found up-regulated in the Nbl CDDP-resistant cell line, were also up-regulated in the CDDP-sensitive cell line after overexpression of *hMT3* gene. Among them, we found cathepsins A, B and D (lysosomal protective proteins), which are cysteine proteases that are widely distributed in the lysosomes of cells in various tissues. Cathepsins are responsible for the uptake of pharmacological compounds present in the cytosol and sequestration of the drugs into exocytosis vesicles. The overexpression of cathepsins was associated with increased drug inactivation and, above all, with increased lysosome trafficking to the plasma membrane followed by the secretion of lysosomal cargo^66^.

Another class of proteins involved in vesicle trafficking is the cytoskeletal, neuronal-associated, microtubule protein tubulin, and microtubule-associated protein which also has a role in resistance to microtubule-targeted drugs in Nbl^67^. Our results showed that tubulins α-4A, β, β-3, and γ-1 (P68366, P07437, Q13509, and P23258, respectively), microtubule-associated protein RP/EB family members 1 and 3 (Q15691 and Q9UPY8, respectively), regulator of microtubule dynamics protein 3 (Q96TC7) and tubulin polymerization-promoting protein (O94811) were all up-regulated due to hMT3 transfection and CDDP-resistance Nbl cell lines. This finding highlighted the demand of vesicle transport and reflected the cell’s commitment to build the microtubule network required for motor proteins to carry vesicles.

In addition, the up-regulation of ATP-binding cassette transporters (ABC family members) provides a supporting mechanism for direct efflux of CDDP from the cell via energy-fuelled active transport. CDDP can further activate the proteasome complex pathway, which was also demonstrated by our previous results^24,46^. Interestingly, we also identified a pronounced up-regulation of numerous proteasome subunits in UKF-NB-4 cells overexpressing hMT3. Most of them participate in the intra-cellular proteasome complex activity. This might be considered as an indicator of major transformations in the cell due to the high demand of protein expression of both structural (*e.g.*, microtubules and vesicles) and functional (*e.g.*, ABC transporters) proteins.

It is clear from the overexpressed hMT3 in the CDDP-sensitive (UK-NB-4) cell line that several drug metabolic processes and responses to oxidative stress were activated, including oxygen radical and cellular detoxification pathways. Variations in the expression and activity of several drug-metabolizing enzymes play a critical role in the drug resistance^68,69^. Overexpression of several heat shock proteins (HSP; highly conserved molecular chaperones involved in cellular homeostasis and stress response) could cause resistance to anticancer drugs^70–72^. We identified overexpression of HSP90 (P08238, P14625), HSP60 (P10809), and HSP10 (P61604), in addition to TRAP-1 (Q12931; a mitochondrial heat shock protein), which could be part of a pro-survival signalling pathway aimed to evade toxic effects of oxidants and anticancer drugs^73^. Notably, the DnaJ heat shock protein family (HSP40; P31689, P25685, O60884, Q9UBS4 Q9H3Z4 and Q99543) was also overexpressed, which implied their role in anticancer drug stress response^72,74^. These data are consistent with our previous study results by microarrays, where we observed the upregulation of HSP40 in the SiMa Nbl cell line with overexpressed hMT3^25^. The implications for ROS regulation are highly significant for cancer therapy because commonly used chemotherapeutic drugs influence tumour outcome through ROS modulation^75,76^. CDDP has been shown to generate oxidative stress and ROS, which could also contribute to its anti-tumour effects^77^. One of the most crucial cell pathways to scavenge the ROS is the glutathione (GSH) redox complex. However, free thiol moieties of MTs can be involved in the scavenging of ROS, produced by the CDDP, in the MT redox cycle. The increase in GSH levels, glutathione synthetase (P48637), and glutathione *S*-Transferase (P09211, P78417, P21266) expression is associated with drug resistance in tumour cells^78^. One important finding was that overexpression of hMT3 in CDDP-sensitive cells induced the up-regulation of thioredoxin (P30048, Q16881 and Q9BRA2) and glutaredoxin (O76003) family proteins, which matches the results found in CDDP-resistant cells. It is known that upregulated thioredoxin and glutaredoxin can be a cause of chemotherapy resistance in various tumour entities^79,80^. Another important defence mechanism against ROS is a family of enzymes called superoxide dismutases (SODs). It is known that SODs and MTs cooperate with each other in the intracellular compartments^81,82^. Overexpression of SOD1 (P00441) has been shown to be one of the factors involved in causing CDDP resistance in ovarian cancer^83^. Overexpression of SOD2 (P04179) has also been shown to increase doxorubicin resistance in gastric carcinoma cells^84^. We also detected overexpression of PARK7 (Q99497; redox-sensitive chaperone), which functions as a sensor for oxidative stress to protect neurons against oxidative stress and cell death^85^. PARK7 was up-regulated in the CDDP-resistant cell line and hMT3 overexpressing CDDP-sensitive cell line. Noteworthy, ferritin (P02792) was up-regulated in both CDDP-resistance cells lines.

In conclusion, this study is one of the most detailed analyses of CDDP-chemoresistance in Nbl by overexpressed hMT3. We identified potential proteins responsible for enhanced CDDP-chemoresistance in Nbl cancer cell lines. Our previously published data revealed increased expression of hMT3 in biopsies from Nbl patients compared to normal adrenal tissues. Unfortunately, for Nbl, there is still a lack of valid clinical data verifying up-regulation of hMT3 in CDDP-resistant tumours. We are eager to continue to work on this aspect. Despite this limitation, we believe that overexpression of hMT3 could be a promising predictor of chemoresistance in Nbl. Our results thus provide clues to the development of drugs in chemoresistant-related therapies.

## Methods

### Chemicals

All chemicals and reagents were purchased from Sigma-Aldrich (St. Louis, MO, USA) in ACS purity, unless noted otherwise.

### Transfection and confocal laser scanning microscopy (CLSM)

The parental UKF-NB-4 cells were established from bone marrow metastases of high-risk Nbl harvested in relapse. The UKF-NB-4^CDDP^ cells were previously established by their incubation with increasing concentration of CDDP in Iscove’s modified Dulbecco’s medium with 10% fetal calf serum until the cells developed a chemoresistance characterized by at least 20-fold increase in resistance to CDDP^86^. For the purpose of this study, the cells were transfected with pcDNA3.1-GFP-hMT3-TOPO encoding full-length hMT3 by Lipofectamine LTX with Plus Reagent (Thermo Fisher Scientific, Waltham, MA, USA) according to manufacturer's instructions. An empty pcDNA3.1-GFP-TOPO was used as the mock control. Before the transfection, UKF-NB-4 cells were seeded onto coverslips (~ 4 × 10^5^ cells/coverslip) and left to adhere overnight. Then, UKF-NB-4 cells were transfected and incubated 24 h. After incubation, the cells were washed with phosphate-buffered saline (PBS) and fixed in 4% formaldehyde (15 min, 25 °C). The coverslips were mounted with ProLongTM Gold Antifade Mountant with 4´,6-diamidino-2-phenylindole (DAPI; Thermo Fisher Scientific) to counterstain nuclei. CLSM (LSM 880, Carl Zeiss, Jena, Germany) was used to visualize fluorescence of nuclei and expressed GFP.

### Evaluation of effect of CDDP administration on viability of Nbl cells

The suspension of approximately 5,000 cells was added to each well of microtiter plates and the cells were incubated overnight. Then, 100 µL of medium containing 2 µg of pcDNA3.1-GFP-hMT3-TOPO or pcDNA3.1-GFP-TOPO as control (mock transfection) and Lipofectamine LTX with Plus Reagent (Thermo Fisher Scientific) was added and the cells were incubated for another 12 h. Then, the medium was replaced with new medium and the cells were incubated for additional 24 h. After that, the medium was replaced with medium containing annotated concentrations of CDDP and the cells were incubated for 24 h. Finally, the viability of cells was assayed using 3-(4,5-dimethylthiazol-2-yl)-2,5-diphenyltetrazolium bromide (MTT) assay and validated by trypan blue exclusion according to our previous study^76^.

### CAM assay

Assays to evaluate metastasis in chick embryos were performed as described by Herrero et al.^87^. Fertilized chicken eggs were obtained from Gilbert farm (Tarragona, Spain) and incubated with rotation at 37.5 °C and 85% humidity for 10 days. We inoculated 1 × 10^5^ tumour cells on the chorioallantoic membrane and incubated the eggs for additional 6 days at 37.5 °C. For the experiment of overexpressed hMT3 in UKF-NB-4, we inoculated 1 × 10^6^ tumour cells on the CAM membrane and incubated the eggs for 5 days at 37.5 °C. Then, the 100 µM CDDP was added topically on the upper CAM at 24 h. At the indicated time points, portions of the CAM, lung, liver and brain were harvested to perform biochemical analyses and to determine the number of human tumour cells which had colonized the tissues by *Alu*-qPCR using a standard curve generated by serial dilutions of human tumour cells spiked into a constant number of chick embryo, as previously described^88^. Data processing and statistical analyses were done using GraphPad Prism (GraphPad Software, San Diego, CA, USA). Levels of tissue colonization are expressed as number of human cells determined by *Alu*-qPCR within 10^6^ host cells and presented as means ± SEM calculated from numerical data from representative or pooled experiments. An unpaired, two-tailed Student’s *t*-test was used to determine *p* values for the differences between the experimental data sets.

### Western blotting

After harvesting the cells by trypsin, the suspension was centrifuged (10,000 rpm, 10 min). Subsequent lysis of the pellet, separation of proteins using sodium dodecyl sulfate polyacrylamide gel electrophoresis, electroblotting on poly(vinylidene fluoride) membrane and blocking of non-specific binding was performed according to our previously published study^46^. For experiments, primary anti-MT3 antibody (ab214314; dilution 1:750, Abcam, Cambridge, UK), anti-GFP antibody (ab183734, dilution 1:2,000, Abcam), anti-GAPDH (ab181602, dilution 1:5,000, Abcam) and anti-α-tubulin (ab7291, dilution 1:5,000, Abcam) were utilized. For development of the bands and their visualization using Azure c600 (Azure Biosystems, Dublin, CA, USA), we used Luminata Forte (EMD Millipore, Burlington, MA, USA). After visualization, western blots densitometric quantitation of signals was performed using ImageJ (v1.53 g, National Institute of Health, Bethesda, MA, USA).

### Isolation of RNA, reverse transcription and qRT-PCR

Isolation of RNA, reverse transcription and qRT-PCR analyses were performed according to our previous study^25^. The specificity of the qRT-PCR was checked by melting curve analysis and the relative levels of transcription were calculated using the 2^−ΔΔCT^ method.

### Matrix-assisted laser desorption/ionization time-of-flight (MALDI-TOF) mass spectrometry (MS) for verification of MT-CDDP complex

The rMT2A (Enzo Biochem, Inc. Farmingdale, NY, USA) was validated for its molecular weight and purity using MALD-TOF mass spectrometry. Briefly, an aliquot of 960 µL of the 10 µM rMT2 solution (in phosphate buffer pH 7.5) was mixed with 40 µL of 500 µM CDDP and incubated for 24 h at 37 °C. The MALDI-TOF experiments were performed using dihydroxybenzoic acid as the matrix. The solutions for the analysis were mixed in a ratio of 1:1 (matrix:substance). After obtaining a homogeneous solution, 1 μL was applied on the MTP ground target plate and dried under atmospheric pressure and ambient temperature. All experiments were performed using Bruker UltrafleXtreme (Bruker Daltonik GmbH, Bremen, Germany) according to Guran et al.^89^.

### Sample preparation and nLC-MS/MS analysis

Protein extraction and processing and proteomic profiling using nLC-MS/MS was carried out according to Merlos Rodrigo et al.^46^. The MS analyses were carried out using Q-Exactive mass spectrometer (Thermo Fisher Scientific) utilizing 2000 V of liquid junction voltage for sample ionization and capillary temperature of 270 °C. Mass spectrometer was coupled to Easy-nLC 1000 nano system (Thermo Fisher Scientific). The protein identification by nLC-MS/MS was carried out in the Proteomics and Genomics Facility (CIB-CSIC), a member of ProteoRed-ISCIII network.

### MS data analysis

The obtained MS data were evaluated and analysed using Proteome Discoverer v.1.4.1.14 (Thermo Fisher Scientific). In our case, the obtained spectrum was searched and compared against the human SwissProt 57.15 database using MASCOT search engine. The analyses was performed following our previously published conditions^46^.

### Quantitation of proteasome complex activity

The activity of proteasome complex was assayed by Proteasome Activity Assay Kit (Abcam) according to manufacturer´s instructions through recording the fluorescence of stained cells (λ_exc_ 350 nm, λ_em_ 440 nm) using SpectraMax i3x Multi-Mode Microplate Reader (Molecular Devices, San Jose, CA, USA) in the presence or absence of preasome inhibitor MG132.

### Bioinformatical tools

For the statistical evaluation of the results, the mean was taken as the measurement of the main tendency, while standard deviation was taken as the dispersion measurement. Differences between groups were analysed using paired *t*-test and ANOVA. The list of processes and/or pathways driven by regulated proteins were created by STRING, DAVID and KEGG classification under the STRING software. The involvement of proteins participated in a cellular process was carried out using the Reactome (www.reactome.org).

## Supplementary Information

Supplementary Information

## Data Availability

The data are available at the university repository upon request.
