# Supplementary material for: Metallothionein-3 promotes cisplatin chemoresistance remodelling in neuroblastoma
Source: Sci Rep. 2021 Mar 9;11:5496. doi: 10.1038/s41598-021-84185-x (PMC7943580; doi:10.1038/s41598-021-84185-x)
Supplement: Supplementary file 1 — Supplementary Information [file 41598_2021_84185_MOESM1_ESM.docx]

*Supplementary Information*

**Metallothionein-3 promotes cisplatin chemoresistance remodelling in neuroblastoma**

Miguel Angel Merlos Rodrigo^1,2^, Hana Michalkova^1,2^, Vladislav Strmiska^1,2^, Berta Casar^3^, Piero Crespo^3^, Vivian de los Rios^4^, J. Ignacio Casal^4^, Yazan Haddad^1,2^, Roman Guran^1,2^, Tomas Eckschlager^5^, Petra Pokorna^5^, Zbynek Heger^1,2^, Vojtech Adam^1,2*^

*^1^Department of Chemistry and Biochemistry, Mendel University in Brno, Zemedelska 1, CZ-613 00 Brno, Czech Republic*

*^2^Central European Institute of Technology, Brno University of Technology, Technicka 3058/10, CZ-616 00 Brno, Czech Republic*

*^3^Instituto de Biomedicina y Biotecnología de Cantabria (IBBTEC), Consejo Superior de Investigaciones Científicas (CSIC)—Universidad de Cantabria, Santander, 39011 Spain*

*^4^Functional Proteomics, Department of Cellular and Molecular Medicine and Proteomic Facility, Centro de Investigaciones Biológicas (CIB-CSIC), Ramiro de Maeztu 9, Madrid 28040, Spain*

*^5^Department of Paediatric Haematology and Oncology, Charles University and University Hospital Motol, V Uvalu 84/1, CZ-150 06 Prague 5, Czech Republic*

***Corresponding author**

Vojtech Adam, Department of Chemistry and Biochemistry, Mendel University in Brno, Zemedelska 1, CZ-613 00 Brno, Czech Republic; E-mail: [vojtech.adam@mendelu.cz](mailto:vojtech.adam@mendelu.cz); phone: +420-5-4513-3350; fax: +420-5-4521-2044.

**Table of Content**

**Supplementary Figure 1:** (A) Validation of the transfection stability during the CAM assay through immunobloting of GFP at 6^th^ day post transfection with hMT3-bearing plasmid. (B) qRT-PCR of genes to confirm selected proteomic results.

**Supplementary Figure 2:** Classification summary of (A) biological processes and (B) pathways affected by up-regulated proteins identified in both UKF-NB-4^hMT3^ and UKF-NB-4^CDDP^.

**Supplementary Table 1:** A full list of proteins detected for *mock* and *hMT3* overexpressed in UKF-NB-4 cell line as well as relevant MS data.

**Supplementary Table 2:** A full list of proteins common detected in *mock* and *hMT3* overexpressed in UKF-NB-4 cell line. Out of total 1481 common protein in two datasets, 438 were up-regulated (fold ratio > 1.5) and 32 were downregulated (fold ratio < 0.5).

**Supplementary Table 3:** A full list of proteins exclusively identified in *hMT3* overexpressed in UKF-NB-4 cell line

**Supplementary Table 4:** The list of processes and/or pathways involved in proteins regulation in UKF-NB-4 cells (*hMT3* vs. mock) using Gene Ontology (GO) annotations and KEGG 10 software.

**Supplementary Table 5:** A full list of proteins detected for *mock* and *hMT3* overexpressed in UKF-NB-4 cell line in comparison with UKF-NB-4^CDDP^ cell line.

**Supplementary Table 1:** A full list of proteins detected for *mock* and *hMT3* overexpressed in UKF-NB-4 cell line as well as relevant MS data.

| Accession | Description | Unique Peptides | PSM | PSM | AAs | MW [kDa] | calc. pI |
| --- | --- | --- | --- | --- | --- | --- | --- |
|  |  |  | UKF-NB-4 | UKF-NB-4 |  |  |  |
|  |  |  | *Mock* | *hMT3* |  |  |  |
| P21333 | Filamin-A OS=Homo sapiens GN=FLNA PE=1 SV=4 - [FLNA_HUMAN] | 70 | 57 | 82 | 2647 | 280.6 | 6.06 |
| Q09666 | Neuroblast differentiation-associated protein AHNAK OS=Homo sapiens GN=AHNAK PE=1 SV=2 - [AHNK_HUMAN] | 54 | 37 | 42 | 5890 | 628.7 | 6.15 |
| P46821 | Microtubule-associated protein 1B OS=Homo sapiens GN=MAP1B PE=1 SV=2 - [MAP1B_HUMAN] | 51 | 43 | 59 | 2468 | 270.5 | 4.81 |
| Q13813 | Spectrin alpha chain, brain OS=Homo sapiens GN=SPTAN1 PE=1 SV=3 - [SPTA2_HUMAN] | 45 | 23 | 43 | 2472 | 284.4 | 5.35 |
| P42704 | Leucine-rich PPR motif-containing protein, mitochondrial OS=Homo sapiens GN=LRPPRC PE=1 SV=3 - [LPPRC_HUMAN] | 42 | 40 | 49 | 1394 | 157.8 | 6.13 |
| P14625 | Endoplasmin OS=Homo sapiens GN=HSP90B1 PE=1 SV=1 - [ENPL_HUMAN] | 34 | 36 | 51 | 803 | 92.4 | 4.84 |
| P10809 | 60 kDa heat shock protein, mitochondrial OS=Homo sapiens GN=HSPD1 PE=1 SV=2 - [CH60_HUMAN] | 30 | 55 | 82 | 573 | 61 | 5.87 |
| P22314 | Ubiquitin-like modifier-activating enzyme 1 OS=Homo sapiens GN=UBA1 PE=1 SV=3 - [UBA1_HUMAN] | 30 | 35 | 52 | 1058 | 117.8 | 5.76 |
| P48681 | Nestin OS=Homo sapiens GN=NES PE=1 SV=2 - [NEST_HUMAN] | 30 | 27 | 30 | 1621 | 177.3 | 4.36 |
| Q14980 | Nuclear mitotic apparatus protein 1 OS=Homo sapiens GN=NUMA1 PE=1 SV=2 - [NUMA1_HUMAN] | 29 | 19 | 28 | 2115 | 238.1 | 5.78 |
| P11021 | 78 kDa glucose-regulated protein OS=Homo sapiens GN=HSPA5 PE=1 SV=2 - [GRP78_HUMAN] | 29 | 43 | 58 | 654 | 72.3 | 5.16 |
| P11142 | Heat shock cognate 71 kDa protein OS=Homo sapiens GN=HSPA8 PE=1 SV=1 - [HSP7C_HUMAN] | 27 | 75 | 82 | 646 | 70.9 | 5.52 |
| P13639 | Elongation factor 2 OS=Homo sapiens GN=EEF2 PE=1 SV=4 - [EF2_HUMAN] | 26 | 26 | 36 | 858 | 95.3 | 6.83 |
| Q14315 | Filamin-C OS=Homo sapiens GN=FLNC PE=1 SV=3 - [FLNC_HUMAN] | 26 | 21 | 29 | 2725 | 290.8 | 5.97 |
| Q9Y4L1 | Hypoxia up-regulated protein 1 OS=Homo sapiens GN=HYOU1 PE=1 SV=1 - [HYOU1_HUMAN] | 26 | 25 | 34 | 999 | 111.3 | 5.22 |
| P07900 | Heat shock protein HSP 90-alpha OS=Homo sapiens GN=HSP90AA1 PE=1 SV=5 - [HS90A_HUMAN] | 26 | 74 | 93 | 732 | 84.6 | 5.02 |
| Q01082 | Spectrin beta chain, brain 1 OS=Homo sapiens GN=SPTBN1 PE=1 SV=2 - [SPTB2_HUMAN] | 25 | 14 | 29 | 2364 | 274.4 | 5.57 |
| Q92499 | ATP-dependent RNA helicase DDX1 OS=Homo sapiens GN=DDX1 PE=1 SV=2 - [DDX1_HUMAN] | 25 | 20 | 28 | 740 | 82.4 | 7.23 |
| P78371 | T-complex protein 1 subunit beta OS=Homo sapiens GN=CCT2 PE=1 SV=4 - [TCPB_HUMAN] | 25 | 28 | 36 | 535 | 57.5 | 6.46 |
| P31939 | Bifunctional purine biosynthesis protein PURH OS=Homo sapiens GN=ATIC PE=1 SV=3 - [PUR9_HUMAN] | 25 | 22 | 28 | 592 | 64.6 | 6.71 |
| P08238 | Heat shock protein HSP 90-beta OS=Homo sapiens GN=HSP90AB1 PE=1 SV=4 - [HS90B_HUMAN] | 25 | 64 | 79 | 724 | 83.2 | 5.03 |
| P55072 | Transitional endoplasmic reticulum ATPase OS=Homo sapiens GN=VCP PE=1 SV=4 - [TERA_HUMAN] | 25 | 27 | 30 | 806 | 89.3 | 5.26 |
| P30101 | Protein disulfide-isomerase A3 OS=Homo sapiens GN=PDIA3 PE=1 SV=4 - [PDIA3_HUMAN] | 24 | 26 | 37 | 505 | 56.7 | 6.35 |
| O00410 | Importin-5 OS=Homo sapiens GN=IPO5 PE=1 SV=4 - [IPO5_HUMAN] | 24 | 18 | 25 | 1097 | 123.5 | 4.94 |
| P08670 | Vimentin OS=Homo sapiens GN=VIM PE=1 SV=4 - [VIME_HUMAN] | 24 | 31 | 35 | 466 | 53.6 | 5.12 |
| P14618 | Pyruvate kinase isozymes M1/M2 OS=Homo sapiens GN=PKM2 PE=1 SV=4 - [KPYM_HUMAN] | 24 | 28 | 26 | 531 | 57.9 | 7.84 |
| P13667 | Protein disulfide-isomerase A4 OS=Homo sapiens GN=PDIA4 PE=1 SV=2 - [PDIA4_HUMAN] | 24 | 31 | 25 | 645 | 72.9 | 5.07 |
| P50990 | T-complex protein 1 subunit theta OS=Homo sapiens GN=CCT8 PE=1 SV=4 - [TCPQ_HUMAN] | 23 | 17 | 22 | 548 | 59.6 | 5.6 |
| Q9Y490 | Talin-1 OS=Homo sapiens GN=TLN1 PE=1 SV=3 - [TLN1_HUMAN] | 23 | 16 | 19 | 2541 | 269.6 | 6.07 |
| Q13263 | Transcription intermediary factor 1-beta OS=Homo sapiens GN=TRIM28 PE=1 SV=5 - [TIF1B_HUMAN] | 22 | 24 | 36 | 835 | 88.5 | 5.77 |
| P49368 | T-complex protein 1 subunit gamma OS=Homo sapiens GN=CCT3 PE=1 SV=4 - [TCPG_HUMAN] | 22 | 16 | 23 | 545 | 60.5 | 6.49 |
| Q15084 | Protein disulfide-isomerase A6 OS=Homo sapiens GN=PDIA6 PE=1 SV=1 - [PDIA6_HUMAN] | 22 | 70 | 98 | 440 | 48.1 | 5.08 |
| P08133 | Annexin A6 OS=Homo sapiens GN=ANXA6 PE=1 SV=3 - [ANXA6_HUMAN] | 22 | 21 | 27 | 673 | 75.8 | 5.6 |
| P05455 | Lupus La protein OS=Homo sapiens GN=SSB PE=1 SV=2 - [LA_HUMAN] | 22 | 22 | 25 | 408 | 46.8 | 7.12 |
| Q12906 | Interleukin enhancer-binding factor 3 OS=Homo sapiens GN=ILF3 PE=1 SV=3 - [ILF3_HUMAN] | 22 | 19 | 21 | 894 | 95.3 | 8.76 |
| Q14974 | Importin subunit beta-1 OS=Homo sapiens GN=KPNB1 PE=1 SV=2 - [IMB1_HUMAN] | 22 | 33 | 33 | 876 | 97.1 | 4.78 |
| P49321 | Nuclear autoantigenic sperm protein OS=Homo sapiens GN=NASP PE=1 SV=2 - [NASP_HUMAN] | 21 | 16 | 27 | 788 | 85.2 | 4.3 |
| P41219 | Peripherin OS=Homo sapiens GN=PRPH PE=1 SV=2 - [PERI_HUMAN] | 21 | 17 | 26 | 470 | 53.6 | 5.47 |
| O00571 | ATP-dependent RNA helicase DDX3X OS=Homo sapiens GN=DDX3X PE=1 SV=3 - [DDX3X_HUMAN] | 21 | 18 | 23 | 662 | 73.2 | 7.18 |
| P38646 | Stress-70 protein, mitochondrial OS=Homo sapiens GN=HSPA9 PE=1 SV=2 - [GRP75_HUMAN] | 21 | 34 | 43 | 679 | 73.6 | 6.16 |
| P12956 | ATP-dependent DNA helicase 2 subunit 1 OS=Homo sapiens GN=XRCC6 PE=1 SV=2 - [KU70_HUMAN] | 21 | 19 | 24 | 609 | 69.8 | 6.64 |
| Q07065 | Cytoskeleton-associated protein 4 OS=Homo sapiens GN=CKAP4 PE=1 SV=2 - [CKAP4_HUMAN] | 21 | 24 | 25 | 602 | 66 | 5.92 |
| P02545 | Lamin-A/C OS=Homo sapiens GN=LMNA PE=1 SV=1 - [LMNA_HUMAN] | 20 | 12 | 22 | 664 | 74.1 | 7.02 |
| P55060 | Exportin-2 OS=Homo sapiens GN=CSE1L PE=1 SV=3 - [XPO2_HUMAN] | 20 | 12 | 21 | 971 | 110.3 | 5.77 |
| P06733 | Alpha-enolase OS=Homo sapiens GN=ENO1 PE=1 SV=2 - [ENOA_HUMAN] | 20 | 50 | 66 | 434 | 47.1 | 7.39 |
| O15061 | Synemin OS=Homo sapiens GN=SYNM PE=1 SV=2 - [SYNEM_HUMAN] | 20 | 14 | 18 | 1565 | 172.7 | 5.16 |
| P07237 | Protein disulfide-isomerase OS=Homo sapiens GN=P4HB PE=1 SV=3 - [PDIA1_HUMAN] | 20 | 24 | 30 | 508 | 57.1 | 4.87 |
| Q16555 | Dihydropyrimidinase-related protein 2 OS=Homo sapiens GN=DPYSL2 PE=1 SV=1 - [DPYL2_HUMAN] | 20 | 26 | 28 | 572 | 62.3 | 6.38 |
| Q86VP6 | Cullin-associated NEDD8-dissociated protein 1 OS=Homo sapiens GN=CAND1 PE=1 SV=2 - [CAND1_HUMAN] | 20 | 16 | 17 | 1230 | 136.3 | 5.78 |
| P07355 | Annexin A2 OS=Homo sapiens GN=ANXA2 PE=1 SV=2 - [ANXA2_HUMAN] | 20 | 32 | 27 | 339 | 38.6 | 7.75 |
| Q16891 | Mitochondrial inner membrane protein OS=Homo sapiens GN=IMMT PE=1 SV=1 - [IMMT_HUMAN] | 19 | 10 | 20 | 758 | 83.6 | 6.48 |
| Q04637 | Eukaryotic translation initiation factor 4 gamma 1 OS=Homo sapiens GN=EIF4G1 PE=1 SV=4 - [IF4G1_HUMAN] | 19 | 14 | 24 | 1599 | 175.4 | 5.33 |
| P19338 | Nucleolin OS=Homo sapiens GN=NCL PE=1 SV=3 - [NUCL_HUMAN] | 19 | 29 | 40 | 710 | 76.6 | 4.7 |
| P06576 | ATP synthase subunit beta, mitochondrial OS=Homo sapiens GN=ATP5B PE=1 SV=3 - [ATPB_HUMAN] | 19 | 26 | 31 | 529 | 56.5 | 5.4 |
| Q14152 | Eukaryotic translation initiation factor 3 subunit A OS=Homo sapiens GN=EIF3A PE=1 SV=1 - [EIF3A_HUMAN] | 19 | 16 | 18 | 1382 | 166.5 | 6.79 |
| Q92598 | Heat shock protein 105 kDa OS=Homo sapiens GN=HSPH1 PE=1 SV=1 - [HS105_HUMAN] | 19 | 19 | 21 | 858 | 96.8 | 5.39 |
| P00558 | Phosphoglycerate kinase 1 OS=Homo sapiens GN=PGK1 PE=1 SV=3 - [PGK1_HUMAN] | 19 | 29 | 32 | 417 | 44.6 | 8.1 |
| P12270 | Nucleoprotein TPR OS=Homo sapiens GN=TPR PE=1 SV=3 - [TPR_HUMAN] | 19 | 15 | 16 | 2363 | 267.1 | 5.02 |
| O14980 | Exportin-1 OS=Homo sapiens GN=XPO1 PE=1 SV=1 - [XPO1_HUMAN] | 19 | 16 | 16 | 1071 | 123.3 | 6.06 |
| P33991 | DNA replication licensing factor MCM4 OS=Homo sapiens GN=MCM4 PE=1 SV=5 - [MCM4_HUMAN] | 18 | 6 | 19 | 863 | 96.5 | 6.74 |
| P31948 | Stress-induced-phosphoprotein 1 OS=Homo sapiens GN=STIP1 PE=1 SV=1 - [STIP1_HUMAN] | 18 | 12 | 21 | 543 | 62.6 | 6.8 |
| P04075 | Fructose-bisphosphate aldolase A OS=Homo sapiens GN=ALDOA PE=1 SV=2 - [ALDOA_HUMAN] | 18 | 28 | 48 | 364 | 39.4 | 8.09 |
| P20700 | Lamin-B1 OS=Homo sapiens GN=LMNB1 PE=1 SV=2 - [LMNB1_HUMAN] | 18 | 17 | 28 | 586 | 66.4 | 5.16 |
| P54577 | Tyrosyl-tRNA synthetase, cytoplasmic OS=Homo sapiens GN=YARS PE=1 SV=4 - [SYYC_HUMAN] | 18 | 15 | 16 | 528 | 59.1 | 7.05 |
| P08107 | Heat shock 70 kDa protein 1A/1B OS=Homo sapiens GN=HSPA1A PE=1 SV=5 - [HSP71_HUMAN] | 18 | 27 | 28 | 641 | 70 | 5.66 |
| P78347 | General transcription factor II-I OS=Homo sapiens GN=GTF2I PE=1 SV=2 - [GTF2I_HUMAN] | 18 | 15 | 15 | 998 | 112.3 | 6.39 |
| O75116 | Rho-associated protein kinase 2 OS=Homo sapiens GN=ROCK2 PE=1 SV=4 - [ROCK2_HUMAN] | 18 | 16 | 13 | 1388 | 160.8 | 6.02 |
| P09874 | Poly [ADP-ribose] polymerase 1 OS=Homo sapiens GN=PARP1 PE=1 SV=4 - [PARP1_HUMAN] | 17 | 9 | 17 | 1014 | 113 | 8.88 |
| P30153 | Serine/threonine-protein phosphatase 2A 65 kDa regulatory subunit A alpha isoform OS=Homo sapiens GN=PPP2R1A PE=1 SV=4 - [2AAA_HUMAN] | 17 | 23 | 33 | 589 | 65.3 | 5.11 |
| P61978 | Heterogeneous nuclear ribonucleoprotein K OS=Homo sapiens GN=HNRNPK PE=1 SV=1 - [HNRPK_HUMAN] | 17 | 23 | 30 | 463 | 50.9 | 5.54 |
| P54136 | Arginyl-tRNA synthetase, cytoplasmic OS=Homo sapiens GN=RARS PE=1 SV=2 - [SYRC_HUMAN] | 17 | 12 | 15 | 660 | 75.3 | 6.68 |
| P49411 | Elongation factor Tu, mitochondrial OS=Homo sapiens GN=TUFM PE=1 SV=2 - [EFTU_HUMAN] | 17 | 17 | 20 | 452 | 49.5 | 7.61 |
| Q92973 | Transportin-1 OS=Homo sapiens GN=TNPO1 PE=1 SV=2 - [TNPO1_HUMAN] | 17 | 15 | 17 | 898 | 102.3 | 4.98 |
| P40227 | T-complex protein 1 subunit zeta OS=Homo sapiens GN=CCT6A PE=1 SV=3 - [TCPZ_HUMAN] | 17 | 13 | 14 | 531 | 58 | 6.68 |
| Q9UHD8 | Septin-9 OS=Homo sapiens GN=SEPT9 PE=1 SV=2 - [SEPT9_HUMAN] | 17 | 15 | 16 | 586 | 65.4 | 8.97 |
| O00429 | Dynamin-1-like protein OS=Homo sapiens GN=DNM1L PE=1 SV=2 - [DNM1L_HUMAN] | 16 | 11 | 20 | 736 | 81.8 | 6.81 |
| P52292 | Importin subunit alpha-2 OS=Homo sapiens GN=KPNA2 PE=1 SV=1 - [IMA2_HUMAN] | 16 | 18 | 27 | 529 | 57.8 | 5.4 |
| P25205 | DNA replication licensing factor MCM3 OS=Homo sapiens GN=MCM3 PE=1 SV=3 - [MCM3_HUMAN] | 16 | 10 | 15 | 808 | 90.9 | 5.77 |
| O43175 | D-3-phosphoglycerate dehydrogenase OS=Homo sapiens GN=PHGDH PE=1 SV=4 - [SERA_HUMAN] | 16 | 21 | 30 | 533 | 56.6 | 6.71 |
| P63244 | Guanine nucleotide-binding protein subunit beta-2-like 1 OS=Homo sapiens GN=GNB2L1 PE=1 SV=3 - [GBLP_HUMAN] | 16 | 16 | 22 | 317 | 35.1 | 7.69 |
| P60174 | Triosephosphate isomerase OS=Homo sapiens GN=TPI1 PE=1 SV=3 - [TPIS_HUMAN] | 16 | 27 | 37 | 286 | 30.8 | 5.92 |
| Q86UP2 | Kinectin OS=Homo sapiens GN=KTN1 PE=1 SV=1 - [KTN1_HUMAN] | 16 | 12 | 16 | 1357 | 156.2 | 5.64 |
| Q9Y230 | RuvB-like 2 OS=Homo sapiens GN=RUVBL2 PE=1 SV=3 - [RUVB2_HUMAN] | 16 | 12 | 16 | 463 | 51.1 | 5.64 |
| P50991 | T-complex protein 1 subunit delta OS=Homo sapiens GN=CCT4 PE=1 SV=4 - [TCPD_HUMAN] | 16 | 16 | 20 | 539 | 57.9 | 7.83 |
| O43707 | Alpha-actinin-4 OS=Homo sapiens GN=ACTN4 PE=1 SV=2 - [ACTN4_HUMAN] | 16 | 26 | 31 | 911 | 104.8 | 5.44 |
| P11586 | C-1-tetrahydrofolate synthase, cytoplasmic OS=Homo sapiens GN=MTHFD1 PE=1 SV=3 - [C1TC_HUMAN] | 16 | 13 | 15 | 935 | 101.5 | 7.3 |
| Q8N163 | Protein KIAA1967 OS=Homo sapiens GN=KIAA1967 PE=1 SV=2 - [K1967_HUMAN] | 16 | 13 | 15 | 923 | 102.8 | 5.22 |
| Q02952 | A-kinase anchor protein 12 OS=Homo sapiens GN=AKAP12 PE=1 SV=4 - [AKA12_HUMAN] | 16 | 13 | 13 | 1782 | 191.4 | 4.41 |
| Q03252 | Lamin-B2 OS=Homo sapiens GN=LMNB2 PE=1 SV=4 - [LMNB2_HUMAN] | 15 | 9 | 18 | 620 | 69.9 | 5.59 |
| Q00839 | Heterogeneous nuclear ribonucleoprotein U OS=Homo sapiens GN=HNRNPU PE=1 SV=6 - [HNRPU_HUMAN] | 15 | 13 | 18 | 825 | 90.5 | 6 |
| P34897 | Serine hydroxymethyltransferase, mitochondrial OS=Homo sapiens GN=SHMT2 PE=1 SV=3 - [GLYM_HUMAN] | 15 | 12 | 15 | 504 | 56 | 8.53 |
| P43243 | Matrin-3 OS=Homo sapiens GN=MATR3 PE=1 SV=2 - [MATR3_HUMAN] | 15 | 18 | 22 | 847 | 94.6 | 6.25 |
| Q02790 | Peptidyl-prolyl cis-trans isomerase FKBP4 OS=Homo sapiens GN=FKBP4 PE=1 SV=3 - [FKBP4_HUMAN] | 15 | 14 | 17 | 459 | 51.8 | 5.43 |
| P08758 | Annexin A5 OS=Homo sapiens GN=ANXA5 PE=1 SV=2 - [ANXA5_HUMAN] | 15 | 17 | 19 | 320 | 35.9 | 5.05 |
| P31350 | Ribonucleoside-diphosphate reductase subunit M2 OS=Homo sapiens GN=RRM2 PE=1 SV=1 - [RIR2_HUMAN] | 15 | 17 | 18 | 389 | 44.8 | 5.38 |
| Q92945 | Far upstream element-binding protein 2 OS=Homo sapiens GN=KHSRP PE=1 SV=4 - [FUBP2_HUMAN] | 15 | 16 | 16 | 711 | 73.1 | 7.3 |
| Q8WUM4 | Programmed cell death 6-interacting protein OS=Homo sapiens GN=PDCD6IP PE=1 SV=1 - [PDC6I_HUMAN] | 15 | 14 | 13 | 868 | 96 | 6.52 |
| P29590 | Probable transcription factor PML OS=Homo sapiens GN=PML PE=1 SV=3 - [PML_HUMAN] | 14 | 8 | 15 | 882 | 97.5 | 6.21 |
| P35998 | 26S protease regulatory subunit 7 OS=Homo sapiens GN=PSMC2 PE=1 SV=3 - [PRS7_HUMAN] | 14 | 6 | 11 | 433 | 48.6 | 5.95 |
| P50454 | Serpin H1 OS=Homo sapiens GN=SERPINH1 PE=1 SV=2 - [SERPH_HUMAN] | 14 | 10 | 17 | 418 | 46.4 | 8.69 |
| P12268 | Inosine-5'-monophosphate dehydrogenase 2 OS=Homo sapiens GN=IMPDH2 PE=1 SV=2 - [IMDH2_HUMAN] | 14 | 13 | 19 | 514 | 55.8 | 6.9 |
| Q16658 | Fascin OS=Homo sapiens GN=FSCN1 PE=1 SV=3 - [FSCN1_HUMAN] | 14 | 11 | 16 | 493 | 54.5 | 7.24 |
| P45974 | Ubiquitin carboxyl-terminal hydrolase 5 OS=Homo sapiens GN=USP5 PE=1 SV=2 - [UBP5_HUMAN] | 14 | 8 | 11 | 858 | 95.7 | 5.03 |
| O00231 | 26S proteasome non-ATPase regulatory subunit 11 OS=Homo sapiens GN=PSMD11 PE=1 SV=3 - [PSD11_HUMAN] | 14 | 11 | 15 | 422 | 47.4 | 6.48 |
| Q9Y265 | RuvB-like 1 OS=Homo sapiens GN=RUVBL1 PE=1 SV=1 - [RUVB1_HUMAN] | 14 | 12 | 16 | 456 | 50.2 | 6.42 |
| P11047 | Laminin subunit gamma-1 OS=Homo sapiens GN=LAMC1 PE=1 SV=3 - [LAMC1_HUMAN] | 14 | 10 | 13 | 1609 | 177.5 | 5.12 |
| P06744 | Glucose-6-phosphate isomerase OS=Homo sapiens GN=GPI PE=1 SV=4 - [G6PI_HUMAN] | 14 | 14 | 18 | 558 | 63.1 | 8.32 |
| P07195 | L-lactate dehydrogenase B chain OS=Homo sapiens GN=LDHB PE=1 SV=2 - [LDHB_HUMAN] | 14 | 22 | 28 | 334 | 36.6 | 6.05 |
| P26641 | Elongation factor 1-gamma OS=Homo sapiens GN=EEF1G PE=1 SV=3 - [EF1G_HUMAN] | 14 | 15 | 18 | 437 | 50.1 | 6.67 |
| P49591 | Seryl-tRNA synthetase, cytoplasmic OS=Homo sapiens GN=SARS PE=1 SV=3 - [SYSC_HUMAN] | 14 | 12 | 14 | 514 | 58.7 | 6.43 |
| P35232 | Prohibitin OS=Homo sapiens GN=PHB PE=1 SV=1 - [PHB_HUMAN] | 14 | 16 | 16 | 272 | 29.8 | 5.76 |
| P48735 | Isocitrate dehydrogenase [NADP], mitochondrial OS=Homo sapiens GN=IDH2 PE=1 SV=2 - [IDHP_HUMAN] | 14 | 14 | 14 | 452 | 50.9 | 8.69 |
| Q99798 | Aconitate hydratase, mitochondrial OS=Homo sapiens GN=ACO2 PE=1 SV=2 - [ACON_HUMAN] | 14 | 12 | 12 | 780 | 85.4 | 7.61 |
| Q9NZI8 | Insulin-like growth factor 2 mRNA-binding protein 1 OS=Homo sapiens GN=IGF2BP1 PE=1 SV=2 - [IF2B1_HUMAN] | 14 | 23 | 21 | 577 | 63.4 | 9.2 |
| Q00341 | Vigilin OS=Homo sapiens GN=HDLBP PE=1 SV=2 - [VIGLN_HUMAN] | 14 | 11 | 10 | 1268 | 141.4 | 6.87 |
| P18206 | Vinculin OS=Homo sapiens GN=VCL PE=1 SV=4 - [VINC_HUMAN] | 14 | 15 | 11 | 1134 | 123.7 | 5.66 |
| P22626 | Heterogeneous nuclear ribonucleoproteins A2/B1 OS=Homo sapiens GN=HNRNPA2B1 PE=1 SV=2 - [ROA2_HUMAN] | 13 | 15 | 29 | 353 | 37.4 | 8.95 |
| P25705 | ATP synthase subunit alpha, mitochondrial OS=Homo sapiens GN=ATP5A1 PE=1 SV=1 - [ATPA_HUMAN] | 13 | 9 | 17 | 553 | 59.7 | 9.13 |
| P46060 | Ran GTPase-activating protein 1 OS=Homo sapiens GN=RANGAP1 PE=1 SV=1 - [RAGP1_HUMAN] | 13 | 7 | 13 | 587 | 63.5 | 4.68 |
| P48643 | T-complex protein 1 subunit epsilon OS=Homo sapiens GN=CCT5 PE=1 SV=1 - [TCPE_HUMAN] | 13 | 10 | 18 | 541 | 59.6 | 5.66 |
| P61247 | 40S ribosomal protein S3a OS=Homo sapiens GN=RPS3A PE=1 SV=2 - [RS3A_HUMAN] | 13 | 10 | 17 | 264 | 29.9 | 9.73 |
| Q06830 | Peroxiredoxin-1 OS=Homo sapiens GN=PRDX1 PE=1 SV=1 - [PRDX1_HUMAN] | 13 | 15 | 25 | 199 | 22.1 | 8.13 |
| P49736 | DNA replication licensing factor MCM2 OS=Homo sapiens GN=MCM2 PE=1 SV=4 - [MCM2_HUMAN] | 13 | 8 | 12 | 904 | 101.8 | 5.52 |
| P17844 | Probable ATP-dependent RNA helicase DDX5 OS=Homo sapiens GN=DDX5 PE=1 SV=1 - [DDX5_HUMAN] | 13 | 13 | 19 | 614 | 69.1 | 8.92 |
| P05091 | Aldehyde dehydrogenase, mitochondrial OS=Homo sapiens GN=ALDH2 PE=1 SV=2 - [ALDH2_HUMAN] | 13 | 10 | 14 | 517 | 56.3 | 7.05 |
| Q15181 | Inorganic pyrophosphatase OS=Homo sapiens GN=PPA1 PE=1 SV=2 - [IPYR_HUMAN] | 13 | 13 | 18 | 289 | 32.6 | 5.86 |
| P12081 | Histidyl-tRNA synthetase, cytoplasmic OS=Homo sapiens GN=HARS PE=1 SV=2 - [SYHC_HUMAN] | 13 | 8 | 11 | 509 | 57.4 | 5.88 |
| P49588 | Alanyl-tRNA synthetase, cytoplasmic OS=Homo sapiens GN=AARS PE=1 SV=2 - [SYAC_HUMAN] | 13 | 9 | 12 | 968 | 106.7 | 5.53 |
| P04792 | Heat shock protein beta-1 OS=Homo sapiens GN=HSPB1 PE=1 SV=2 - [HSPB1_HUMAN] | 13 | 21 | 26 | 205 | 22.8 | 6.4 |
| Q13200 | 26S proteasome non-ATPase regulatory subunit 2 OS=Homo sapiens GN=PSMD2 PE=1 SV=3 - [PSMD2_HUMAN] | 13 | 10 | 12 | 908 | 100.1 | 5.2 |
| P40926 | Malate dehydrogenase, mitochondrial OS=Homo sapiens GN=MDH2 PE=1 SV=3 - [MDHM_HUMAN] | 13 | 21 | 25 | 338 | 35.5 | 8.68 |
| P30041 | Peroxiredoxin-6 OS=Homo sapiens GN=PRDX6 PE=1 SV=3 - [PRDX6_HUMAN] | 13 | 16 | 18 | 224 | 25 | 6.38 |
| P26639 | Threonyl-tRNA synthetase, cytoplasmic OS=Homo sapiens GN=TARS PE=1 SV=3 - [SYTC_HUMAN] | 13 | 8 | 9 | 723 | 83.4 | 6.67 |
| P53396 | ATP-citrate synthase OS=Homo sapiens GN=ACLY PE=1 SV=3 - [ACLY_HUMAN] | 13 | 11 | 12 | 1101 | 120.8 | 7.33 |
| P49915 | GMP synthase [glutamine-hydrolyzing] OS=Homo sapiens GN=GMPS PE=1 SV=1 - [GUAA_HUMAN] | 13 | 16 | 17 | 693 | 76.7 | 6.87 |
| O60701 | UDP-glucose 6-dehydrogenase OS=Homo sapiens GN=UGDH PE=1 SV=1 - [UGDH_HUMAN] | 13 | 10 | 10 | 494 | 55 | 7.12 |
| O43390 | Heterogeneous nuclear ribonucleoprotein R OS=Homo sapiens GN=HNRNPR PE=1 SV=1 - [HNRPR_HUMAN] | 13 | 16 | 15 | 633 | 70.9 | 8.13 |
| Q9UIG0 | Tyrosine-protein kinase BAZ1B OS=Homo sapiens GN=BAZ1B PE=1 SV=2 - [BAZ1B_HUMAN] | 12 | 3 | 11 | 1483 | 170.8 | 8.48 |
| Q93009 | Ubiquitin carboxyl-terminal hydrolase 7 OS=Homo sapiens GN=USP7 PE=1 SV=2 - [UBP7_HUMAN] | 12 | 4 | 11 | 1102 | 128.2 | 5.55 |
| Q96QK1 | Vacuolar protein sorting-associated protein 35 OS=Homo sapiens GN=VPS35 PE=1 SV=2 - [VPS35_HUMAN] | 12 | 5 | 13 | 796 | 91.6 | 5.49 |
| P62701 | 40S ribosomal protein S4, X isoform OS=Homo sapiens GN=RPS4X PE=1 SV=2 - [RS4X_HUMAN] | 12 | 9 | 16 | 263 | 29.6 | 10.15 |
| P33993 | DNA replication licensing factor MCM7 OS=Homo sapiens GN=MCM7 PE=1 SV=4 - [MCM7_HUMAN] | 12 | 7 | 12 | 719 | 81.3 | 6.46 |
| P27816 | Microtubule-associated protein 4 OS=Homo sapiens GN=MAP4 PE=1 SV=3 - [MAP4_HUMAN] | 12 | 6 | 10 | 1152 | 120.9 | 5.43 |
| P04406 | Glyceraldehyde-3-phosphate dehydrogenase OS=Homo sapiens GN=GAPDH PE=1 SV=3 - [G3P_HUMAN] | 12 | 24 | 38 | 335 | 36 | 8.46 |
| P09651 | Heterogeneous nuclear ribonucleoprotein A1 OS=Homo sapiens GN=HNRNPA1 PE=1 SV=5 - [ROA1_HUMAN] | 12 | 19 | 29 | 372 | 38.7 | 9.13 |
| P29401 | Transketolase OS=Homo sapiens GN=TKT PE=1 SV=3 - [TKT_HUMAN] | 12 | 10 | 15 | 623 | 67.8 | 7.66 |
| Q9UQ80 | Proliferation-associated protein 2G4 OS=Homo sapiens GN=PA2G4 PE=1 SV=3 - [PA2G4_HUMAN] | 12 | 10 | 15 | 394 | 43.8 | 6.55 |
| Q99459 | Cell division cycle 5-like protein OS=Homo sapiens GN=CDC5L PE=1 SV=2 - [CDC5L_HUMAN] | 12 | 9 | 13 | 802 | 92.2 | 8.18 |
| P27797 | Calreticulin OS=Homo sapiens GN=CALR PE=1 SV=1 - [CALR_HUMAN] | 12 | 14 | 20 | 417 | 48.1 | 4.44 |
| P31943 | Heterogeneous nuclear ribonucleoprotein H OS=Homo sapiens GN=HNRNPH1 PE=1 SV=4 - [HNRH1_HUMAN] | 12 | 19 | 26 | 449 | 49.2 | 6.3 |
| Q16643 | Drebrin OS=Homo sapiens GN=DBN1 PE=1 SV=4 - [DREB_HUMAN] | 12 | 13 | 17 | 649 | 71.4 | 4.45 |
| P52272 | Heterogeneous nuclear ribonucleoprotein M OS=Homo sapiens GN=HNRNPM PE=1 SV=3 - [HNRPM_HUMAN] | 12 | 12 | 15 | 730 | 77.5 | 8.7 |
| P43490 | Nicotinamide phosphoribosyltransferase OS=Homo sapiens GN=NAMPT PE=1 SV=1 - [NAMPT_HUMAN] | 12 | 8 | 10 | 491 | 55.5 | 7.15 |
| P22234 | Multifunctional protein ADE2 OS=Homo sapiens GN=PAICS PE=1 SV=3 - [PUR6_HUMAN] | 12 | 10 | 12 | 425 | 47 | 7.23 |
| P27824 | Calnexin OS=Homo sapiens GN=CANX PE=1 SV=2 - [CALX_HUMAN] | 12 | 15 | 17 | 592 | 67.5 | 4.6 |
| P35579 | Myosin-9 OS=Homo sapiens GN=MYH9 PE=1 SV=4 - [MYH9_HUMAN] | 12 | 11 | 12 | 1960 | 226.4 | 5.6 |
| P34932 | Heat shock 70 kDa protein 4 OS=Homo sapiens GN=HSPA4 PE=1 SV=4 - [HSP74_HUMAN] | 12 | 11 | 12 | 840 | 94.3 | 5.19 |
| P21796 | Voltage-dependent anion-selective channel protein 1 OS=Homo sapiens GN=VDAC1 PE=1 SV=2 - [VDAC1_HUMAN] | 12 | 12 | 13 | 283 | 30.8 | 8.54 |
| Q96AE4 | Far upstream element-binding protein 1 OS=Homo sapiens GN=FUBP1 PE=1 SV=3 - [FUBP1_HUMAN] | 12 | 13 | 14 | 644 | 67.5 | 7.61 |
| Q12905 | Interleukin enhancer-binding factor 2 OS=Homo sapiens GN=ILF2 PE=1 SV=2 - [ILF2_HUMAN] | 12 | 16 | 16 | 390 | 43 | 5.26 |
| O60841 | Eukaryotic translation initiation factor 5B OS=Homo sapiens GN=EIF5B PE=1 SV=4 - [IF2P_HUMAN] | 12 | 10 | 10 | 1220 | 138.7 | 5.49 |
| Q99832 | T-complex protein 1 subunit eta OS=Homo sapiens GN=CCT7 PE=1 SV=2 - [TCPH_HUMAN] | 12 | 16 | 15 | 543 | 59.3 | 7.65 |
| P22102 | Trifunctional purine biosynthetic protein adenosine-3 OS=Homo sapiens GN=GART PE=1 SV=1 - [PUR2_HUMAN] | 12 | 9 | 8 | 1010 | 107.7 | 6.7 |
| P13797 | Plastin-3 OS=Homo sapiens GN=PLS3 PE=1 SV=4 - [PLST_HUMAN] | 12 | 11 | 9 | 630 | 70.8 | 5.6 |
| P05023 | Sodium/potassium-transporting ATPase subunit alpha-1 OS=Homo sapiens GN=ATP1A1 PE=1 SV=1 - [AT1A1_HUMAN] | 12 | 12 | 8 | 1023 | 112.8 | 5.49 |
| P28331 | NADH-ubiquinone oxidoreductase 75 kDa subunit, mitochondrial OS=Homo sapiens GN=NDUFS1 PE=1 SV=3 - [NDUS1_HUMAN] | 11 | 4 | 13 | 727 | 79.4 | 6.23 |
| P13489 | Ribonuclease inhibitor OS=Homo sapiens GN=RNH1 PE=1 SV=2 - [RINI_HUMAN] | 11 | 5 | 12 | 461 | 49.9 | 4.82 |
| P78344 | Eukaryotic translation initiation factor 4 gamma 2 OS=Homo sapiens GN=EIF4G2 PE=1 SV=1 - [IF4G2_HUMAN] | 11 | 5 | 11 | 907 | 102.3 | 7.14 |
| Q14697 | Neutral alpha-glucosidase AB OS=Homo sapiens GN=GANAB PE=1 SV=3 - [GANAB_HUMAN] | 11 | 5 | 11 | 944 | 106.8 | 6.14 |
| P35249 | Replication factor C subunit 4 OS=Homo sapiens GN=RFC4 PE=1 SV=2 - [RFC4_HUMAN] | 11 | 5 | 11 | 363 | 39.7 | 8.02 |
| Q14157 | Ubiquitin-associated protein 2-like OS=Homo sapiens GN=UBAP2L PE=1 SV=2 - [UBP2L_HUMAN] | 11 | 7 | 15 | 1087 | 114.5 | 7.11 |
| Q99623 | Prohibitin-2 OS=Homo sapiens GN=PHB2 PE=1 SV=2 - [PHB2_HUMAN] | 11 | 7 | 14 | 299 | 33.3 | 9.83 |
| P12814 | Alpha-actinin-1 OS=Homo sapiens GN=ACTN1 PE=1 SV=2 - [ACTN1_HUMAN] | 11 | 17 | 29 | 892 | 103 | 5.41 |
| Q7KZF4 | Staphylococcal nuclease domain-containing protein 1 OS=Homo sapiens GN=SND1 PE=1 SV=1 - [SND1_HUMAN] | 11 | 6 | 10 | 910 | 101.9 | 7.17 |
| P49419 | Alpha-aminoadipic semialdehyde dehydrogenase OS=Homo sapiens GN=ALDH7A1 PE=1 SV=5 - [AL7A1_HUMAN] | 11 | 7 | 11 | 539 | 58.5 | 7.99 |
| Q13283 | Ras GTPase-activating protein-binding protein 1 OS=Homo sapiens GN=G3BP1 PE=1 SV=1 - [G3BP1_HUMAN] | 11 | 15 | 22 | 466 | 52.1 | 5.52 |
| O95373 | Importin-7 OS=Homo sapiens GN=IPO7 PE=1 SV=1 - [IPO7_HUMAN] | 11 | 11 | 16 | 1038 | 119.4 | 4.82 |
| P00338 | L-lactate dehydrogenase A chain OS=Homo sapiens GN=LDHA PE=1 SV=2 - [LDHA_HUMAN] | 11 | 11 | 16 | 332 | 36.7 | 8.27 |
| P09211 | Glutathione S-transferase P OS=Homo sapiens GN=GSTP1 PE=1 SV=2 - [GSTP1_HUMAN] | 11 | 13 | 18 | 210 | 23.3 | 5.64 |
| P26599 | Polypyrimidine tract-binding protein 1 OS=Homo sapiens GN=PTBP1 PE=1 SV=1 - [PTBP1_HUMAN] | 11 | 16 | 22 | 531 | 57.2 | 9.17 |
| Q96CX2 | BTB/POZ domain-containing protein KCTD12 OS=Homo sapiens GN=KCTD12 PE=1 SV=1 - [KCD12_HUMAN] | 11 | 11 | 15 | 325 | 35.7 | 5.64 |
| Q96AG4 | Leucine-rich repeat-containing protein 59 OS=Homo sapiens GN=LRRC59 PE=1 SV=1 - [LRC59_HUMAN] | 11 | 10 | 13 | 307 | 34.9 | 9.57 |
| P08195 | 4F2 cell-surface antigen heavy chain OS=Homo sapiens GN=SLC3A2 PE=1 SV=3 - [4F2_HUMAN] | 11 | 7 | 9 | 630 | 68 | 5.01 |
| P23396 | 40S ribosomal protein S3 OS=Homo sapiens GN=RPS3 PE=1 SV=2 - [RS3_HUMAN] | 11 | 7 | 9 | 243 | 26.7 | 9.66 |
| O00299 | Chloride intracellular channel protein 1 OS=Homo sapiens GN=CLIC1 PE=1 SV=4 - [CLIC1_HUMAN] | 11 | 8 | 10 | 241 | 26.9 | 5.17 |
| P38159 | Heterogeneous nuclear ribonucleoprotein G OS=Homo sapiens GN=RBMX PE=1 SV=3 - [HNRPG_HUMAN] | 11 | 9 | 11 | 391 | 42.3 | 10.05 |
| Q08J23 | tRNA (cytosine-5-)-methyltransferase NSUN2 OS=Homo sapiens GN=NSUN2 PE=1 SV=2 - [NSUN2_HUMAN] | 11 | 7 | 8 | 767 | 86.4 | 6.77 |
| P11940 | Polyadenylate-binding protein 1 OS=Homo sapiens GN=PABPC1 PE=1 SV=2 - [PABP1_HUMAN] | 11 | 17 | 19 | 636 | 70.6 | 9.5 |
| Q9NSD9 | Phenylalanine--tRNA ligase beta subunit OS=Homo sapiens GN=FARSB PE=1 SV=3 - [SYFB_HUMAN] | 11 | 10 | 11 | 589 | 66.1 | 6.84 |
| O60506 | Heterogeneous nuclear ribonucleoprotein Q OS=Homo sapiens GN=SYNCRIP PE=1 SV=2 - [HNRPQ_HUMAN] | 11 | 14 | 15 | 623 | 69.6 | 8.59 |
| P63104 | 14-3-3 protein zeta/delta OS=Homo sapiens GN=YWHAZ PE=1 SV=1 - [1433Z_HUMAN] | 11 | 29 | 29 | 245 | 27.7 | 4.79 |
| Q99615 | DnaJ homolog subfamily C member 7 OS=Homo sapiens GN=DNAJC7 PE=1 SV=2 - [DNJC7_HUMAN] | 11 | 10 | 10 | 494 | 56.4 | 6.96 |
| Q15459 | Splicing factor 3A subunit 1 OS=Homo sapiens GN=SF3A1 PE=1 SV=1 - [SF3A1_HUMAN] | 11 | 8 | 8 | 793 | 88.8 | 5.22 |
| Q00610 | Clathrin heavy chain 1 OS=Homo sapiens GN=CLTC PE=1 SV=5 - [CLH1_HUMAN] | 11 | 10 | 9 | 1675 | 191.5 | 5.69 |
| Q15019 | Septin-2 OS=Homo sapiens GN=SEPT2 PE=1 SV=1 - [SEPT2_HUMAN] | 11 | 13 | 11 | 361 | 41.5 | 6.6 |
| P02786 | Transferrin receptor protein 1 OS=Homo sapiens GN=TFRC PE=1 SV=2 - [TFR1_HUMAN] | 11 | 10 | 6 | 760 | 84.8 | 6.61 |
| Q16352 | Alpha-internexin OS=Homo sapiens GN=INA PE=1 SV=2 - [AINX_HUMAN] | 10 | 3 | 11 | 499 | 55.4 | 5.4 |
| P52788 | Spermine synthase OS=Homo sapiens GN=SMS PE=1 SV=2 - [SPSY_HUMAN] | 10 | 3 | 11 | 366 | 41.2 | 5.02 |
| Q6PKG0 | La-related protein 1 OS=Homo sapiens GN=LARP1 PE=1 SV=2 - [LARP1_HUMAN] | 10 | 5 | 11 | 1096 | 123.4 | 8.82 |
| Q9Y696 | Chloride intracellular channel protein 4 OS=Homo sapiens GN=CLIC4 PE=1 SV=4 - [CLIC4_HUMAN] | 10 | 7 | 14 | 253 | 28.8 | 5.59 |
| O14776 | Transcription elongation regulator 1 OS=Homo sapiens GN=TCERG1 PE=1 SV=2 - [TCRG1_HUMAN] | 10 | 5 | 10 | 1098 | 123.8 | 8.65 |
| P36776 | Lon protease homolog, mitochondrial OS=Homo sapiens GN=LONP1 PE=1 SV=2 - [LONM_HUMAN] | 10 | 6 | 11 | 959 | 106.4 | 6.39 |
| Q13177 | Serine/threonine-protein kinase PAK 2 OS=Homo sapiens GN=PAK2 PE=1 SV=3 - [PAK2_HUMAN] | 10 | 6 | 11 | 524 | 58 | 5.96 |
| P17174 | Aspartate aminotransferase, cytoplasmic OS=Homo sapiens GN=GOT1 PE=1 SV=3 - [AATC_HUMAN] | 10 | 6 | 11 | 413 | 46.2 | 7.01 |
| Q9UKX7 | Nuclear pore complex protein Nup50 OS=Homo sapiens GN=NUP50 PE=1 SV=2 - [NUP50_HUMAN] | 10 | 5 | 9 | 468 | 50.1 | 7.06 |
| Q9NTK5 | Obg-like ATPase 1 OS=Homo sapiens GN=OLA1 PE=1 SV=2 - [OLA1_HUMAN] | 10 | 7 | 11 | 396 | 44.7 | 7.81 |
| P36578 | 60S ribosomal protein L4 OS=Homo sapiens GN=RPL4 PE=1 SV=5 - [RL4_HUMAN] | 10 | 7 | 11 | 427 | 47.7 | 11.06 |
| P05388 | 60S acidic ribosomal protein P0 OS=Homo sapiens GN=RPLP0 PE=1 SV=1 - [RLA0_HUMAN] | 10 | 10 | 15 | 317 | 34.3 | 5.97 |
| P52209 | 6-phosphogluconate dehydrogenase, decarboxylating OS=Homo sapiens GN=PGD PE=1 SV=3 - [6PGD_HUMAN] | 10 | 6 | 9 | 483 | 53.1 | 7.23 |
| P67809 | Nuclease-sensitive element-binding protein 1 OS=Homo sapiens GN=YBX1 PE=1 SV=3 - [YBOX1_HUMAN] | 10 | 11 | 16 | 324 | 35.9 | 9.88 |
| P49327 | Fatty acid synthase OS=Homo sapiens GN=FASN PE=1 SV=3 - [FAS_HUMAN] | 10 | 5 | 7 | 2511 | 273.3 | 6.44 |
| P38606 | V-type proton ATPase catalytic subunit A OS=Homo sapiens GN=ATP6V1A PE=1 SV=2 - [VATA_HUMAN] | 10 | 8 | 11 | 617 | 68.3 | 5.52 |
| Q9Y5M8 | Signal recognition particle receptor subunit beta OS=Homo sapiens GN=SRPRB PE=1 SV=3 - [SRPRB_HUMAN] | 10 | 6 | 8 | 271 | 29.7 | 9.04 |
| P62937 | Peptidyl-prolyl cis-trans isomerase A OS=Homo sapiens GN=PPIA PE=1 SV=2 - [PPIA_HUMAN] | 10 | 17 | 22 | 165 | 18 | 7.81 |
| Q9HB71 | Calcyclin-binding protein OS=Homo sapiens GN=CACYBP PE=1 SV=2 - [CYBP_HUMAN] | 10 | 9 | 11 | 228 | 26.2 | 8.25 |
| P51149 | Ras-related protein Rab-7a OS=Homo sapiens GN=RAB7A PE=1 SV=1 - [RAB7A_HUMAN] | 10 | 9 | 11 | 207 | 23.5 | 6.7 |
| P45880 | Voltage-dependent anion-selective channel protein 2 OS=Homo sapiens GN=VDAC2 PE=1 SV=2 - [VDAC2_HUMAN] | 10 | 9 | 11 | 294 | 31.5 | 7.56 |
| Q15785 | Mitochondrial import receptor subunit TOM34 OS=Homo sapiens GN=TOMM34 PE=1 SV=2 - [TOM34_HUMAN] | 10 | 10 | 12 | 309 | 34.5 | 8.98 |
| P42765 | 3-ketoacyl-CoA thiolase, mitochondrial OS=Homo sapiens GN=ACAA2 PE=1 SV=2 - [THIM_HUMAN] | 10 | 7 | 8 | 397 | 41.9 | 8.09 |
| P27348 | 14-3-3 protein theta OS=Homo sapiens GN=YWHAQ PE=1 SV=1 - [1433T_HUMAN] | 10 | 23 | 26 | 245 | 27.7 | 4.78 |
| Q14566 | DNA replication licensing factor MCM6 OS=Homo sapiens GN=MCM6 PE=1 SV=1 - [MCM6_HUMAN] | 10 | 8 | 9 | 821 | 92.8 | 5.41 |
| Q9UNZ2 | NSFL1 cofactor p47 OS=Homo sapiens GN=NSFL1C PE=1 SV=2 - [NSF1C_HUMAN] | 10 | 8 | 9 | 370 | 40.5 | 5.1 |
| P23284 | Peptidyl-prolyl cis-trans isomerase B OS=Homo sapiens GN=PPIB PE=1 SV=2 - [PPIB_HUMAN] | 10 | 10 | 11 | 216 | 23.7 | 9.41 |
| P51991 | Heterogeneous nuclear ribonucleoprotein A3 OS=Homo sapiens GN=HNRNPA3 PE=1 SV=2 - [ROA3_HUMAN] | 10 | 12 | 13 | 378 | 39.6 | 9.01 |
| P12277 | Creatine kinase B-type OS=Homo sapiens GN=CKB PE=1 SV=1 - [KCRB_HUMAN] | 10 | 15 | 16 | 381 | 42.6 | 5.59 |
| P62258 | 14-3-3 protein epsilon OS=Homo sapiens GN=YWHAE PE=1 SV=1 - [1433E_HUMAN] | 10 | 22 | 23 | 255 | 29.2 | 4.74 |
| P33176 | Kinesin-1 heavy chain OS=Homo sapiens GN=KIF5B PE=1 SV=1 - [KINH_HUMAN] | 10 | 12 | 12 | 963 | 109.6 | 6.51 |
| P43686 | 26S protease regulatory subunit 6B OS=Homo sapiens GN=PSMC4 PE=1 SV=2 - [PRS6B_HUMAN] | 10 | 10 | 10 | 418 | 47.3 | 5.21 |
| P27695 | DNA-(apurinic or apyrimidinic site) lyase OS=Homo sapiens GN=APEX1 PE=1 SV=2 - [APEX1_HUMAN] | 10 | 10 | 10 | 318 | 35.5 | 8.12 |
| Q08257 | Quinone oxidoreductase OS=Homo sapiens GN=CRYZ PE=1 SV=1 - [QOR_HUMAN] | 10 | 10 | 10 | 329 | 35.2 | 8.44 |
| P13010 | ATP-dependent DNA helicase 2 subunit 2 OS=Homo sapiens GN=XRCC5 PE=1 SV=3 - [KU86_HUMAN] | 10 | 8 | 8 | 732 | 82.7 | 5.81 |
| Q08211 | ATP-dependent RNA helicase A OS=Homo sapiens GN=DHX9 PE=1 SV=4 - [DHX9_HUMAN] | 10 | 7 | 7 | 1270 | 140.9 | 6.84 |
| Q96AY3 | Peptidyl-prolyl cis-trans isomerase FKBP10 OS=Homo sapiens GN=FKBP10 PE=1 SV=1 - [FKB10_HUMAN] | 10 | 13 | 11 | 582 | 64.2 | 5.62 |
| P16152 | Carbonyl reductase [NADPH] 1 OS=Homo sapiens GN=CBR1 PE=1 SV=3 - [CBR1_HUMAN] | 10 | 11 | 9 | 277 | 30.4 | 8.32 |
| P55084 | Trifunctional enzyme subunit beta, mitochondrial OS=Homo sapiens GN=HADHB PE=1 SV=3 - [ECHB_HUMAN] | 10 | 8 | 6 | 474 | 51.3 | 9.41 |
| P28838 | Cytosol aminopeptidase OS=Homo sapiens GN=LAP3 PE=1 SV=3 - [AMPL_HUMAN] | 10 | 9 | 6 | 519 | 56.1 | 7.93 |
| O15042 | U2-associated protein SR140 OS=Homo sapiens GN=SR140 PE=1 SV=2 - [SR140_HUMAN] | 9 | 2 | 8 | 1029 | 118.2 | 8.47 |
| O76003 | Glutaredoxin-3 OS=Homo sapiens GN=GLRX3 PE=1 SV=2 - [GLRX3_HUMAN] | 9 | 3 | 11 | 335 | 37.4 | 5.39 |
| Q9UQE7 | Structural maintenance of chromosomes protein 3 OS=Homo sapiens GN=SMC3 PE=1 SV=2 - [SMC3_HUMAN] | 9 | 3 | 8 | 1217 | 141.5 | 7.18 |
| P98175 | RNA-binding protein 10 OS=Homo sapiens GN=RBM10 PE=1 SV=3 - [RBM10_HUMAN] | 9 | 3 | 8 | 930 | 103.5 | 5.97 |
| Q05682 | Caldesmon OS=Homo sapiens GN=CALD1 PE=1 SV=3 - [CALD1_HUMAN] | 9 | 3 | 8 | 793 | 93.2 | 5.66 |
| Q99497 | Protein DJ-1 OS=Homo sapiens GN=PARK7 PE=1 SV=2 - [PARK7_HUMAN] | 9 | 6 | 15 | 189 | 19.9 | 6.79 |
| Q9NQG5 | Regulation of nuclear pre-mRNA domain-containing protein 1B OS=Homo sapiens GN=RPRD1B PE=1 SV=1 - [RPR1B_HUMAN] | 9 | 7 | 14 | 326 | 36.9 | 5.97 |
| P37802 | Transgelin-2 OS=Homo sapiens GN=TAGLN2 PE=1 SV=3 - [TAGL2_HUMAN] | 9 | 7 | 14 | 199 | 22.4 | 8.25 |
| P29692 | Elongation factor 1-delta OS=Homo sapiens GN=EEF1D PE=1 SV=5 - [EF1D_HUMAN] | 9 | 6 | 12 | 281 | 31.1 | 5.01 |
| P52306 | Rap1 GTPase-GDP dissociation stimulator 1 OS=Homo sapiens GN=RAP1GDS1 PE=1 SV=3 - [GDS1_HUMAN] | 9 | 4 | 8 | 607 | 66.3 | 5.31 |
| Q9UNF1 | Melanoma-associated antigen D2 OS=Homo sapiens GN=MAGED2 PE=1 SV=2 - [MAGD2_HUMAN] | 9 | 5 | 9 | 606 | 64.9 | 9.32 |
| P16615 | Sarcoplasmic/endoplasmic reticulum calcium ATPase 2 OS=Homo sapiens GN=ATP2A2 PE=1 SV=1 - [AT2A2_HUMAN] | 9 | 5 | 9 | 1042 | 114.7 | 5.34 |
| P46781 | 40S ribosomal protein S9 OS=Homo sapiens GN=RPS9 PE=1 SV=3 - [RS9_HUMAN] | 9 | 5 | 9 | 194 | 22.6 | 10.65 |
| P30086 | Phosphatidylethanolamine-binding protein 1 OS=Homo sapiens GN=PEBP1 PE=1 SV=3 - [PEBP1_HUMAN] | 9 | 9 | 15 | 187 | 21 | 7.53 |
| Q8N1G4 | Leucine-rich repeat-containing protein 47 OS=Homo sapiens GN=LRRC47 PE=1 SV=1 - [LRC47_HUMAN] | 9 | 6 | 10 | 583 | 63.4 | 8.28 |
| O94826 | Mitochondrial import receptor subunit TOM70 OS=Homo sapiens GN=TOMM70A PE=1 SV=1 - [TOM70_HUMAN] | 9 | 6 | 10 | 608 | 67.4 | 7.12 |
| P13804 | Electron transfer flavoprotein subunit alpha, mitochondrial OS=Homo sapiens GN=ETFA PE=1 SV=1 - [ETFA_HUMAN] | 9 | 7 | 11 | 333 | 35.1 | 8.38 |
| P61289 | Proteasome activator complex subunit 3 OS=Homo sapiens GN=PSME3 PE=1 SV=1 - [PSME3_HUMAN] | 9 | 10 | 15 | 254 | 29.5 | 5.95 |
| P37235 | Hippocalcin-like protein 1 OS=Homo sapiens GN=HPCAL1 PE=1 SV=3 - [HPCL1_HUMAN] | 9 | 10 | 15 | 193 | 22.3 | 5.35 |
| Q13162 | Peroxiredoxin-4 OS=Homo sapiens GN=PRDX4 PE=1 SV=1 - [PRDX4_HUMAN] | 9 | 11 | 16 | 271 | 30.5 | 6.29 |
| Q9UBT2 | SUMO-activating enzyme subunit 2 OS=Homo sapiens GN=UBA2 PE=1 SV=2 - [SAE2_HUMAN] | 9 | 7 | 10 | 640 | 71.2 | 5.29 |
| P23246 | Splicing factor, proline- and glutamine-rich OS=Homo sapiens GN=SFPQ PE=1 SV=2 - [SFPQ_HUMAN] | 9 | 10 | 14 | 707 | 76.1 | 9.44 |
| Q9Y3F4 | Serine-threonine kinase receptor-associated protein OS=Homo sapiens GN=STRAP PE=1 SV=1 - [STRAP_HUMAN] | 9 | 5 | 7 | 350 | 38.4 | 5.12 |
| P78417 | Glutathione S-transferase omega-1 OS=Homo sapiens GN=GSTO1 PE=1 SV=2 - [GSTO1_HUMAN] | 9 | 8 | 11 | 241 | 27.5 | 6.6 |
| P32119 | Peroxiredoxin-2 OS=Homo sapiens GN=PRDX2 PE=1 SV=5 - [PRDX2_HUMAN] | 9 | 14 | 19 | 198 | 21.9 | 5.97 |
| Q9Y2Z0 | Suppressor of G2 allele of SKP1 homolog OS=Homo sapiens GN=SUGT1 PE=1 SV=3 - [SUGT1_HUMAN] | 9 | 6 | 8 | 365 | 41 | 5.16 |
| O43852 | Calumenin OS=Homo sapiens GN=CALU PE=1 SV=2 - [CALU_HUMAN] | 9 | 6 | 8 | 315 | 37.1 | 4.64 |
| P60228 | Eukaryotic translation initiation factor 3 subunit E OS=Homo sapiens GN=EIF3E PE=1 SV=1 - [EIF3E_HUMAN] | 9 | 7 | 9 | 445 | 52.2 | 6.04 |
| P62269 | 40S ribosomal protein S18 OS=Homo sapiens GN=RPS18 PE=1 SV=3 - [RS18_HUMAN] | 9 | 7 | 9 | 152 | 17.7 | 10.99 |
| Q12765 | Secernin-1 OS=Homo sapiens GN=SCRN1 PE=1 SV=2 - [SCRN1_HUMAN] | 9 | 8 | 10 | 414 | 46.4 | 4.75 |
| P07910 | Heterogeneous nuclear ribonucleoproteins C1/C2 OS=Homo sapiens GN=HNRNPC PE=1 SV=4 - [HNRPC_HUMAN] | 9 | 9 | 11 | 306 | 33.6 | 5.08 |
| Q99714 | 3-hydroxyacyl-CoA dehydrogenase type-2 OS=Homo sapiens GN=HSD17B10 PE=1 SV=3 - [HCD2_HUMAN] | 9 | 9 | 11 | 261 | 26.9 | 7.78 |
| P62826 | GTP-binding nuclear protein Ran OS=Homo sapiens GN=RAN PE=1 SV=3 - [RAN_HUMAN] | 9 | 9 | 11 | 216 | 24.4 | 7.49 |
| O60313 | Dynamin-like 120 kDa protein, mitochondrial OS=Homo sapiens GN=OPA1 PE=1 SV=3 - [OPA1_HUMAN] | 9 | 6 | 7 | 960 | 111.6 | 7.87 |
| P25786 | Proteasome subunit alpha type-1 OS=Homo sapiens GN=PSMA1 PE=1 SV=1 - [PSA1_HUMAN] | 9 | 6 | 7 | 263 | 29.5 | 6.61 |
| O00425 | Insulin-like growth factor 2 mRNA-binding protein 3 OS=Homo sapiens GN=IGF2BP3 PE=1 SV=2 - [IF2B3_HUMAN] | 9 | 10 | 11 | 579 | 63.7 | 8.87 |
| Q92841 | Probable ATP-dependent RNA helicase DDX17 OS=Homo sapiens GN=DDX17 PE=1 SV=2 - [DDX17_HUMAN] | 9 | 11 | 12 | 729 | 80.2 | 8.27 |
| P55209 | Nucleosome assembly protein 1-like 1 OS=Homo sapiens GN=NAP1L1 PE=1 SV=1 - [NP1L1_HUMAN] | 9 | 12 | 13 | 391 | 45.3 | 4.46 |
| P08865 | 40S ribosomal protein SA OS=Homo sapiens GN=RPSA PE=1 SV=4 - [RSSA_HUMAN] | 9 | 15 | 16 | 295 | 32.8 | 4.87 |
| Q9NQC3 | Reticulon-4 OS=Homo sapiens GN=RTN4 PE=1 SV=2 - [RTN4_HUMAN] | 9 | 12 | 12 | 1192 | 129.9 | 4.5 |
| Q96I24 | Far upstream element-binding protein 3 OS=Homo sapiens GN=FUBP3 PE=1 SV=2 - [FUBP3_HUMAN] | 9 | 9 | 9 | 572 | 61.6 | 8.38 |
| O14818 | Proteasome subunit alpha type-7 OS=Homo sapiens GN=PSMA7 PE=1 SV=1 - [PSA7_HUMAN] | 9 | 8 | 8 | 248 | 27.9 | 8.46 |
| Q5JTV8 | Torsin-1A-interacting protein 1 OS=Homo sapiens GN=TOR1AIP1 PE=1 SV=2 - [TOIP1_HUMAN] | 9 | 5 | 5 | 583 | 66.2 | 8.18 |
| Q12874 | Splicing factor 3A subunit 3 OS=Homo sapiens GN=SF3A3 PE=1 SV=1 - [SF3A3_HUMAN] | 9 | 9 | 7 | 501 | 58.8 | 5.38 |
| Q9BT78 | COP9 signalosome complex subunit 4 OS=Homo sapiens GN=COPS4 PE=1 SV=1 - [CSN4_HUMAN] | 9 | 8 | 6 | 406 | 46.2 | 5.83 |
| Q99613 | Eukaryotic translation initiation factor 3 subunit C OS=Homo sapiens GN=EIF3C PE=1 SV=1 - [EIF3C_HUMAN] | 9 | 8 | 3 | 913 | 105.3 | 5.68 |
| P51665 | 26S proteasome non-ATPase regulatory subunit 7 OS=Homo sapiens GN=PSMD7 PE=1 SV=2 - [PSD7_HUMAN] | 8 | 2 | 10 | 324 | 37 | 6.77 |
| Q9Y224 | UPF0568 protein C14orf166 OS=Homo sapiens GN=C14orf166 PE=1 SV=1 - [CN166_HUMAN] | 8 | 3 | 11 | 244 | 28.1 | 6.65 |
| O60610 | Protein diaphanous homolog 1 OS=Homo sapiens GN=DIAPH1 PE=1 SV=2 - [DIAP1_HUMAN] | 8 | 2 | 7 | 1272 | 141.3 | 5.41 |
| P54819 | Adenylate kinase 2, mitochondrial OS=Homo sapiens GN=AK2 PE=1 SV=2 - [KAD2_HUMAN] | 8 | 3 | 10 | 239 | 26.5 | 7.81 |
| O75533 | Splicing factor 3B subunit 1 OS=Homo sapiens GN=SF3B1 PE=1 SV=3 - [SF3B1_HUMAN] | 8 | 2 | 6 | 1304 | 145.7 | 7.09 |
| O43290 | U4/U6.U5 tri-snRNP-associated protein 1 OS=Homo sapiens GN=SART1 PE=1 SV=1 - [SNUT1_HUMAN] | 8 | 3 | 8 | 800 | 90.2 | 6.13 |
| Q14693 | Phosphatidate phosphatase LPIN1 OS=Homo sapiens GN=LPIN1 PE=2 SV=2 - [LPIN1_HUMAN] | 8 | 3 | 8 | 890 | 98.6 | 6.58 |
| Q9UL46 | Proteasome activator complex subunit 2 OS=Homo sapiens GN=PSME2 PE=1 SV=4 - [PSME2_HUMAN] | 8 | 3 | 8 | 239 | 27.4 | 5.73 |
| Q9UNM6 | 26S proteasome non-ATPase regulatory subunit 13 OS=Homo sapiens GN=PSMD13 PE=1 SV=2 - [PSD13_HUMAN] | 8 | 4 | 10 | 376 | 42.9 | 5.81 |
| P21266 | Glutathione S-transferase Mu 3 OS=Homo sapiens GN=GSTM3 PE=1 SV=3 - [GSTM3_HUMAN] | 8 | 4 | 10 | 225 | 26.5 | 5.54 |
| P09661 | U2 small nuclear ribonucleoprotein A' OS=Homo sapiens GN=SNRPA1 PE=1 SV=2 - [RU2A_HUMAN] | 8 | 4 | 10 | 255 | 28.4 | 8.62 |
| P49959 | Double-strand break repair protein MRE11A OS=Homo sapiens GN=MRE11A PE=1 SV=3 - [MRE11_HUMAN] | 8 | 3 | 7 | 708 | 80.5 | 5.9 |
| P25685 | DnaJ homolog subfamily B member 1 OS=Homo sapiens GN=DNAJB1 PE=1 SV=4 - [DNJB1_HUMAN] | 8 | 4 | 9 | 340 | 38 | 8.63 |
| P68400 | Casein kinase II subunit alpha OS=Homo sapiens GN=CSNK2A1 PE=1 SV=1 - [CSK21_HUMAN] | 8 | 4 | 9 | 391 | 45.1 | 7.74 |
| P00492 | Hypoxanthine-guanine phosphoribosyltransferase OS=Homo sapiens GN=HPRT1 PE=1 SV=2 - [HPRT_HUMAN] | 8 | 4 | 9 | 218 | 24.6 | 6.68 |
| P33316 | Deoxyuridine 5'-triphosphate nucleotidohydrolase, mitochondrial OS=Homo sapiens GN=DUT PE=1 SV=4 - [DUT_HUMAN] | 8 | 7 | 15 | 252 | 26.5 | 9.36 |
| Q7Z460 | CLIP-associating protein 1 OS=Homo sapiens GN=CLASP1 PE=1 SV=1 - [CLAP1_HUMAN] | 8 | 5 | 10 | 1538 | 169.3 | 9.03 |
| P11216 | Glycogen phosphorylase, brain form OS=Homo sapiens GN=PYGB PE=1 SV=5 - [PYGB_HUMAN] | 8 | 3 | 6 | 843 | 96.6 | 6.86 |
| Q15691 | Microtubule-associated protein RP/EB family member 1 OS=Homo sapiens GN=MAPRE1 PE=1 SV=3 - [MARE1_HUMAN] | 8 | 6 | 11 | 268 | 30 | 5.14 |
| O75534 | Cold shock domain-containing protein E1 OS=Homo sapiens GN=CSDE1 PE=1 SV=2 - [CSDE1_HUMAN] | 8 | 4 | 7 | 798 | 88.8 | 6.25 |
| Q15417 | Calponin-3 OS=Homo sapiens GN=CNN3 PE=1 SV=1 - [CNN3_HUMAN] | 8 | 7 | 12 | 329 | 36.4 | 6.05 |
| P07737 | Profilin-1 OS=Homo sapiens GN=PFN1 PE=1 SV=2 - [PROF1_HUMAN] | 8 | 7 | 12 | 140 | 15 | 8.27 |
| P18669 | Phosphoglycerate mutase 1 OS=Homo sapiens GN=PGAM1 PE=1 SV=2 - [PGAM1_HUMAN] | 8 | 8 | 13 | 254 | 28.8 | 7.18 |
| P43246 | DNA mismatch repair protein Msh2 OS=Homo sapiens GN=MSH2 PE=1 SV=1 - [MSH2_HUMAN] | 8 | 5 | 8 | 934 | 104.7 | 5.77 |
| O00499 | Myc box-dependent-interacting protein 1 OS=Homo sapiens GN=BIN1 PE=1 SV=1 - [BIN1_HUMAN] | 8 | 5 | 8 | 593 | 64.7 | 5.06 |
| P40939 | Trifunctional enzyme subunit alpha, mitochondrial OS=Homo sapiens GN=HADHA PE=1 SV=2 - [ECHA_HUMAN] | 8 | 5 | 8 | 763 | 82.9 | 9.04 |
| P62081 | 40S ribosomal protein S7 OS=Homo sapiens GN=RPS7 PE=1 SV=1 - [RS7_HUMAN] | 8 | 7 | 11 | 194 | 22.1 | 10.1 |
| Q07955 | Splicing factor, arginine/serine-rich 1 OS=Homo sapiens GN=SFRS1 PE=1 SV=2 - [SFRS1_HUMAN] | 8 | 12 | 18 | 248 | 27.7 | 10.36 |
| P61158 | Actin-related protein 3 OS=Homo sapiens GN=ACTR3 PE=1 SV=3 - [ARP3_HUMAN] | 8 | 6 | 9 | 418 | 47.3 | 5.88 |
| Q9H0D6 | 5'-3' exoribonuclease 2 OS=Homo sapiens GN=XRN2 PE=1 SV=1 - [XRN2_HUMAN] | 8 | 4 | 6 | 950 | 108.5 | 7.47 |
| Q02878 | 60S ribosomal protein L6 OS=Homo sapiens GN=RPL6 PE=1 SV=3 - [RL6_HUMAN] | 8 | 7 | 10 | 288 | 32.7 | 10.58 |
| Q15907 | Ras-related protein Rab-11B OS=Homo sapiens GN=RAB11B PE=1 SV=4 - [RB11B_HUMAN] | 8 | 7 | 10 | 218 | 24.5 | 5.94 |
| P62979 | Ubiquitin-40S ribosomal protein S27a OS=Homo sapiens GN=RPS27A PE=1 SV=2 - [RS27A_HUMAN] | 8 | 10 | 14 | 156 | 18 | 9.64 |
| P08621 | U1 small nuclear ribonucleoprotein 70 kDa OS=Homo sapiens GN=SNRNP70 PE=1 SV=2 - [RU17_HUMAN] | 8 | 5 | 7 | 437 | 51.5 | 9.94 |
| P54578 | Ubiquitin carboxyl-terminal hydrolase 14 OS=Homo sapiens GN=USP14 PE=1 SV=3 - [UBP14_HUMAN] | 8 | 5 | 7 | 494 | 56 | 5.3 |
| P11310 | Medium-chain specific acyl-CoA dehydrogenase, mitochondrial OS=Homo sapiens GN=ACADM PE=1 SV=1 - [ACADM_HUMAN] | 8 | 5 | 7 | 421 | 46.6 | 8.37 |
| Q9HC38 | Glyoxalase domain-containing protein 4 OS=Homo sapiens GN=GLOD4 PE=1 SV=1 - [GLOD4_HUMAN] | 8 | 5 | 7 | 313 | 34.8 | 5.6 |
| Q12931 | Heat shock protein 75 kDa, mitochondrial OS=Homo sapiens GN=TRAP1 PE=1 SV=3 - [TRAP1_HUMAN] | 8 | 8 | 11 | 704 | 80.1 | 8.21 |
| Q9NVA2 | Septin-11 OS=Homo sapiens GN=SEPT11 PE=1 SV=3 - [SEP11_HUMAN] | 8 | 8 | 11 | 429 | 49.4 | 6.81 |
| P46782 | 40S ribosomal protein S5 OS=Homo sapiens GN=RPS5 PE=1 SV=4 - [RS5_HUMAN] | 8 | 11 | 15 | 204 | 22.9 | 9.72 |
| O76021 | Ribosomal L1 domain-containing protein 1 OS=Homo sapiens GN=RSL1D1 PE=1 SV=3 - [RL1D1_HUMAN] | 8 | 6 | 8 | 490 | 54.9 | 10.13 |
| Q9UHB9 | Signal recognition particle 68 kDa protein OS=Homo sapiens GN=SRP68 PE=1 SV=2 - [SRP68_HUMAN] | 8 | 6 | 8 | 627 | 70.7 | 8.56 |
| Q9Y6E2 | Basic leucine zipper and W2 domain-containing protein 2 OS=Homo sapiens GN=BZW2 PE=1 SV=1 - [BZW2_HUMAN] | 8 | 6 | 8 | 419 | 48.1 | 6.68 |
| P50213 | Isocitrate dehydrogenase [NAD] subunit alpha, mitochondrial OS=Homo sapiens GN=IDH3A PE=1 SV=1 - [IDH3A_HUMAN] | 8 | 6 | 8 | 366 | 39.6 | 6.92 |
| P42166 | Lamina-associated polypeptide 2, isoform alpha OS=Homo sapiens GN=TMPO PE=1 SV=2 - [LAP2A_HUMAN] | 8 | 12 | 15 | 694 | 75.4 | 7.66 |
| P47756 | F-actin-capping protein subunit beta OS=Homo sapiens GN=CAPZB PE=1 SV=4 - [CAPZB_HUMAN] | 8 | 8 | 10 | 277 | 31.3 | 5.59 |
| O43396 | Thioredoxin-like protein 1 OS=Homo sapiens GN=TXNL1 PE=1 SV=3 - [TXNL1_HUMAN] | 8 | 8 | 10 | 289 | 32.2 | 4.96 |
| Q7Z4S6 | Kinesin-like protein KIF21A OS=Homo sapiens GN=KIF21A PE=1 SV=2 - [KI21A_HUMAN] | 8 | 4 | 5 | 1674 | 187.1 | 6.42 |
| P30040 | Endoplasmic reticulum resident protein 29 OS=Homo sapiens GN=ERP29 PE=1 SV=4 - [ERP29_HUMAN] | 8 | 9 | 11 | 261 | 29 | 7.31 |
| Q9BTE3 | Mini-chromosome maintenance complex-binding protein OS=Homo sapiens GN=MCMBP PE=1 SV=2 - [MCMBP_HUMAN] | 8 | 5 | 6 | 642 | 72.9 | 5.87 |
| P14866 | Heterogeneous nuclear ribonucleoprotein L OS=Homo sapiens GN=HNRNPL PE=1 SV=2 - [HNRPL_HUMAN] | 8 | 5 | 6 | 589 | 64.1 | 8.22 |
| P00505 | Aspartate aminotransferase, mitochondrial OS=Homo sapiens GN=GOT2 PE=1 SV=3 - [AATM_HUMAN] | 8 | 5 | 6 | 430 | 47.5 | 9.01 |
| O95433 | Activator of 90 kDa heat shock protein ATPase homolog 1 OS=Homo sapiens GN=AHSA1 PE=1 SV=1 - [AHSA1_HUMAN] | 8 | 5 | 6 | 338 | 38.3 | 5.53 |
| P23528 | Cofilin-1 OS=Homo sapiens GN=CFL1 PE=1 SV=3 - [COF1_HUMAN] | 8 | 16 | 19 | 166 | 18.5 | 8.09 |
| P17812 | CTP synthase 1 OS=Homo sapiens GN=CTPS PE=1 SV=2 - [PYRG1_HUMAN] | 8 | 6 | 7 | 591 | 66.6 | 6.46 |
| Q14684 | Ribosomal RNA processing protein 1 homolog B OS=Homo sapiens GN=RRP1B PE=1 SV=3 - [RRP1B_HUMAN] | 8 | 6 | 7 | 758 | 84.4 | 9.76 |
| Q14247 | Src substrate cortactin OS=Homo sapiens GN=CTTN PE=1 SV=2 - [SRC8_HUMAN] | 8 | 7 | 8 | 550 | 61.5 | 5.4 |
| Q8IV08 | Phospholipase D3 OS=Homo sapiens GN=PLD3 PE=1 SV=1 - [PLD3_HUMAN] | 8 | 9 | 10 | 490 | 54.7 | 6.47 |
| P09936 | Ubiquitin carboxyl-terminal hydrolase isozyme L1 OS=Homo sapiens GN=UCHL1 PE=1 SV=2 - [UCHL1_HUMAN] | 8 | 10 | 11 | 223 | 24.8 | 5.48 |
| Q9UJZ1 | Stomatin-like protein 2 OS=Homo sapiens GN=STOML2 PE=1 SV=1 - [STML2_HUMAN] | 8 | 10 | 10 | 356 | 38.5 | 7.39 |
| Q16181 | Septin-7 OS=Homo sapiens GN=SEPT7 PE=1 SV=2 - [SEPT7_HUMAN] | 8 | 9 | 9 | 437 | 50.6 | 8.63 |
| P12004 | Proliferating cell nuclear antigen OS=Homo sapiens GN=PCNA PE=1 SV=1 - [PCNA_HUMAN] | 8 | 9 | 9 | 261 | 28.8 | 4.69 |
| P41250 | Glycine--tRNA ligase OS=Homo sapiens GN=GARS PE=1 SV=3 - [SYG_HUMAN] | 8 | 8 | 8 | 739 | 83.1 | 7.03 |
| Q01518 | Adenylyl cyclase-associated protein 1 OS=Homo sapiens GN=CAP1 PE=1 SV=5 - [CAP1_HUMAN] | 8 | 8 | 8 | 475 | 51.9 | 8.06 |
| Q07866 | Kinesin light chain 1 OS=Homo sapiens GN=KLC1 PE=1 SV=2 - [KLC1_HUMAN] | 8 | 7 | 7 | 573 | 65.3 | 6.2 |
| Q8NBS9 | Thioredoxin domain-containing protein 5 OS=Homo sapiens GN=TXNDC5 PE=1 SV=2 - [TXND5_HUMAN] | 8 | 7 | 7 | 432 | 47.6 | 5.97 |
| P11387 | DNA topoisomerase 1 OS=Homo sapiens GN=TOP1 PE=1 SV=2 - [TOP1_HUMAN] | 8 | 6 | 6 | 765 | 90.7 | 9.31 |
| P12955 | Xaa-Pro dipeptidase OS=Homo sapiens GN=PEPD PE=1 SV=3 - [PEPD_HUMAN] | 8 | 6 | 6 | 493 | 54.5 | 6 |
| O15355 | Protein phosphatase 1G OS=Homo sapiens GN=PPM1G PE=1 SV=1 - [PPM1G_HUMAN] | 8 | 6 | 6 | 546 | 59.2 | 4.36 |
| P49354 | Protein farnesyltransferase/geranylgeranyltransferase type-1 subunit alpha OS=Homo sapiens GN=FNTA PE=1 SV=1 - [FNTA_HUMAN] | 8 | 6 | 6 | 379 | 44.4 | 5.08 |
| Q9HDC9 | Adipocyte plasma membrane-associated protein OS=Homo sapiens GN=APMAP PE=1 SV=2 - [APMAP_HUMAN] | 8 | 6 | 6 | 416 | 46.5 | 6.16 |
| P06748 | Nucleophosmin OS=Homo sapiens GN=NPM1 PE=1 SV=2 - [NPM_HUMAN] | 8 | 26 | 25 | 294 | 32.6 | 4.78 |
| P80723 | Brain acid soluble protein 1 OS=Homo sapiens GN=BASP1 PE=1 SV=2 - [BASP1_HUMAN] | 8 | 8 | 7 | 227 | 22.7 | 4.63 |
| P38117 | Electron transfer flavoprotein subunit beta OS=Homo sapiens GN=ETFB PE=1 SV=3 - [ETFB_HUMAN] | 8 | 7 | 6 | 255 | 27.8 | 8.1 |
| P40925 | Malate dehydrogenase, cytoplasmic OS=Homo sapiens GN=MDH1 PE=1 SV=4 - [MDHC_HUMAN] | 8 | 12 | 10 | 334 | 36.4 | 7.36 |
| Q08752 | Peptidyl-prolyl cis-trans isomerase D OS=Homo sapiens GN=PPID PE=1 SV=3 - [PPID_HUMAN] | 8 | 5 | 4 | 370 | 40.7 | 7.21 |
| Q9HAV4 | Exportin-5 OS=Homo sapiens GN=XPO5 PE=1 SV=1 - [XPO5_HUMAN] | 8 | 9 | 6 | 1204 | 136.2 | 5.8 |
| P00367 | Glutamate dehydrogenase 1, mitochondrial OS=Homo sapiens GN=GLUD1 PE=1 SV=2 - [DHE3_HUMAN] | 8 | 8 | 5 | 558 | 61.4 | 7.8 |
| Q9P289 | Serine/threonine-protein kinase MST4 OS=Homo sapiens GN=MST4 PE=1 SV=2 - [MST4_HUMAN] | 8 | 8 | 5 | 416 | 46.5 | 5.29 |
| Q7L0Y3 | Mitochondrial ribonuclease P protein 1 OS=Homo sapiens GN=RG9MTD1 PE=1 SV=2 - [MRRP1_HUMAN] | 7 | 1 | 7 | 403 | 47.3 | 9.36 |
| O76094 | Signal recognition particle 72 kDa protein OS=Homo sapiens GN=SRP72 PE=1 SV=3 - [SRP72_HUMAN] | 7 | 2 | 7 | 671 | 74.6 | 9.26 |
| Q8WU90 | Zinc finger CCCH domain-containing protein 15 OS=Homo sapiens GN=ZC3H15 PE=1 SV=1 - [ZC3HF_HUMAN] | 7 | 2 | 7 | 426 | 48.6 | 5.31 |
| Q9BY77 | Polymerase delta-interacting protein 3 OS=Homo sapiens GN=POLDIP3 PE=1 SV=2 - [PDIP3_HUMAN] | 7 | 2 | 7 | 421 | 46.1 | 9.99 |
| P48047 | ATP synthase subunit O, mitochondrial OS=Homo sapiens GN=ATP5O PE=1 SV=1 - [ATPO_HUMAN] | 7 | 3 | 9 | 213 | 23.3 | 9.96 |
| O14497 | AT-rich interactive domain-containing protein 1A OS=Homo sapiens GN=ARID1A PE=1 SV=3 - [ARI1A_HUMAN] | 7 | 2 | 6 | 2285 | 241.9 | 6.7 |
| Q9NWY4 | Histone PARylation factor 1 OS=Homo sapiens GN=HPF1 PE=1 SV=2 - [HPF1_HUMAN] | 7 | 2 | 6 | 346 | 39.4 | 6.8 |
| Q12904 | Aminoacyl tRNA synthetase complex-interacting multifunctional protein 1 OS=Homo sapiens GN=AIMP1 PE=1 SV=2 - [AIMP1_HUMAN] | 7 | 3 | 8 | 312 | 34.3 | 8.43 |
| P06493 | Cyclin-dependent kinase 1 OS=Homo sapiens GN=CDK1 PE=1 SV=3 - [CDK1_HUMAN] | 7 | 3 | 8 | 297 | 34.1 | 8.4 |
| P35637 | RNA-binding protein FUS OS=Homo sapiens GN=FUS PE=1 SV=1 - [FUS_HUMAN] | 7 | 5 | 12 | 526 | 53.4 | 9.36 |
| P15121 | Aldose reductase OS=Homo sapiens GN=AKR1B1 PE=1 SV=3 - [ALDR_HUMAN] | 7 | 3 | 7 | 316 | 35.8 | 6.98 |
| Q9Y5K5 | Ubiquitin carboxyl-terminal hydrolase isozyme L5 OS=Homo sapiens GN=UCHL5 PE=1 SV=3 - [UCHL5_HUMAN] | 7 | 3 | 7 | 329 | 37.6 | 5.33 |
| P61586 | Transforming protein RhoA OS=Homo sapiens GN=RHOA PE=1 SV=1 - [RHOA_HUMAN] | 7 | 4 | 9 | 193 | 21.8 | 6.1 |
| Q15365 | Poly(rC)-binding protein 1 OS=Homo sapiens GN=PCBP1 PE=1 SV=2 - [PCBP1_HUMAN] | 7 | 8 | 16 | 356 | 37.5 | 7.09 |
| P50502 | Hsc70-interacting protein OS=Homo sapiens GN=ST13 PE=1 SV=2 - [F10A1_HUMAN] | 7 | 5 | 10 | 369 | 41.3 | 5.27 |
| P17980 | 26S protease regulatory subunit 6A OS=Homo sapiens GN=PSMC3 PE=1 SV=3 - [PRS6A_HUMAN] | 7 | 4 | 8 | 439 | 49.2 | 5.24 |
| P31689 | DnaJ homolog subfamily A member 1 OS=Homo sapiens GN=DNAJA1 PE=1 SV=2 - [DNJA1_HUMAN] | 7 | 4 | 8 | 397 | 44.8 | 7.08 |
| P00568 | Adenylate kinase isoenzyme 1 OS=Homo sapiens GN=AK1 PE=1 SV=3 - [KAD1_HUMAN] | 7 | 4 | 8 | 194 | 21.6 | 8.63 |
| Q14444 | Caprin-1 OS=Homo sapiens GN=CAPRIN1 PE=1 SV=2 - [CAPR1_HUMAN] | 7 | 3 | 6 | 709 | 78.3 | 5.25 |
| Q9UKM9 | RNA-binding protein Raly OS=Homo sapiens GN=RALY PE=1 SV=1 - [RALY_HUMAN] | 7 | 3 | 6 | 306 | 32.4 | 9.17 |
| Q9Y3I0 | UPF0027 protein C22orf28 OS=Homo sapiens GN=C22orf28 PE=1 SV=1 - [CV028_HUMAN] | 7 | 4 | 7 | 505 | 55.2 | 7.23 |
| P22695 | Cytochrome b-c1 complex subunit 2, mitochondrial OS=Homo sapiens GN=UQCRC2 PE=1 SV=3 - [QCR2_HUMAN] | 7 | 4 | 7 | 453 | 48.4 | 8.63 |
| Q9H3N1 | Thioredoxin-related transmembrane protein 1 OS=Homo sapiens GN=TMX1 PE=1 SV=1 - [TMX1_HUMAN] | 7 | 4 | 7 | 280 | 31.8 | 4.98 |
| Q9UKK9 | ADP-sugar pyrophosphatase OS=Homo sapiens GN=NUDT5 PE=1 SV=1 - [NUDT5_HUMAN] | 7 | 4 | 7 | 219 | 24.3 | 4.94 |
| P09104 | Gamma-enolase OS=Homo sapiens GN=ENO2 PE=1 SV=3 - [ENOG_HUMAN] | 7 | 13 | 22 | 434 | 47.2 | 5.03 |
| Q9Y285 | Phenylalanyl-tRNA synthetase alpha chain OS=Homo sapiens GN=FARSA PE=1 SV=3 - [SYFA_HUMAN] | 7 | 6 | 10 | 508 | 57.5 | 7.8 |
| P61604 | 10 kDa heat shock protein, mitochondrial OS=Homo sapiens GN=HSPE1 PE=1 SV=2 - [CH10_HUMAN] | 7 | 6 | 10 | 102 | 10.9 | 8.92 |
| Q15758 | Neutral amino acid transporter B(0) OS=Homo sapiens GN=SLC1A5 PE=1 SV=2 - [AAAT_HUMAN] | 7 | 3 | 5 | 541 | 56.6 | 5.48 |
| P63151 | Serine/threonine-protein phosphatase 2A 55 kDa regulatory subunit B alpha isoform OS=Homo sapiens GN=PPP2R2A PE=1 SV=1 - [2ABA_HUMAN] | 7 | 3 | 5 | 447 | 51.7 | 6.2 |
| P23193 | Transcription elongation factor A protein 1 OS=Homo sapiens GN=TCEA1 PE=1 SV=2 - [TCEA1_HUMAN] | 7 | 3 | 5 | 301 | 33.9 | 8.38 |
| P07954 | Fumarate hydratase, mitochondrial OS=Homo sapiens GN=FH PE=1 SV=3 - [FUMH_HUMAN] | 7 | 5 | 8 | 510 | 54.6 | 8.76 |
| O43837 | Isocitrate dehydrogenase [NAD] subunit beta, mitochondrial OS=Homo sapiens GN=IDH3B PE=1 SV=2 - [IDH3B_HUMAN] | 7 | 5 | 8 | 385 | 42.2 | 8.46 |
| P31930 | Cytochrome b-c1 complex subunit 1, mitochondrial OS=Homo sapiens GN=UQCRC1 PE=1 SV=3 - [QCR1_HUMAN] | 7 | 5 | 8 | 480 | 52.6 | 6.37 |
| P09429 | High mobility group protein B1 OS=Homo sapiens GN=HMGB1 PE=1 SV=3 - [HMGB1_HUMAN] | 7 | 7 | 11 | 215 | 24.9 | 5.74 |
| P26583 | High mobility group protein B2 OS=Homo sapiens GN=HMGB2 PE=1 SV=2 - [HMGB2_HUMAN] | 7 | 9 | 14 | 209 | 24 | 7.81 |
| Q9Y266 | Nuclear migration protein nudC OS=Homo sapiens GN=NUDC PE=1 SV=1 - [NUDC_HUMAN] | 7 | 6 | 9 | 331 | 38.2 | 5.38 |
| P15880 | 40S ribosomal protein S2 OS=Homo sapiens GN=RPS2 PE=1 SV=2 - [RS2_HUMAN] | 7 | 6 | 9 | 293 | 31.3 | 10.24 |
| Q8WWM7 | Ataxin-2-like protein OS=Homo sapiens GN=ATXN2L PE=1 SV=2 - [ATX2L_HUMAN] | 7 | 4 | 6 | 1075 | 113.3 | 8.59 |
| Q96KR1 | Zinc finger RNA-binding protein OS=Homo sapiens GN=ZFR PE=1 SV=2 - [ZFR_HUMAN] | 7 | 4 | 6 | 1074 | 116.9 | 9.04 |
| Q86UE4 | Protein LYRIC OS=Homo sapiens GN=MTDH PE=1 SV=2 - [LYRIC_HUMAN] | 7 | 4 | 6 | 582 | 63.8 | 9.32 |
| Q9H773 | dCTP pyrophosphatase 1 OS=Homo sapiens GN=DCTPP1 PE=1 SV=1 - [DCTP1_HUMAN] | 7 | 4 | 6 | 170 | 18.7 | 5.03 |
| Q08945 | FACT complex subunit SSRP1 OS=Homo sapiens GN=SSRP1 PE=1 SV=1 - [SSRP1_HUMAN] | 7 | 5 | 7 | 709 | 81 | 6.87 |
| Q00534 | Cell division protein kinase 6 OS=Homo sapiens GN=CDK6 PE=1 SV=1 - [CDK6_HUMAN] | 7 | 5 | 7 | 326 | 36.9 | 6.46 |
| Q13126 | S-methyl-5'-thioadenosine phosphorylase OS=Homo sapiens GN=MTAP PE=1 SV=2 - [MTAP_HUMAN] | 7 | 5 | 7 | 283 | 31.2 | 7.18 |
| Q13561 | Dynactin subunit 2 OS=Homo sapiens GN=DCTN2 PE=1 SV=4 - [DCTN2_HUMAN] | 7 | 6 | 8 | 401 | 44.2 | 5.21 |
| Q99733 | Nucleosome assembly protein 1-like 4 OS=Homo sapiens GN=NAP1L4 PE=1 SV=1 - [NP1L4_HUMAN] | 7 | 7 | 9 | 375 | 42.8 | 4.69 |
| P29966 | Myristoylated alanine-rich C-kinase substrate OS=Homo sapiens GN=MARCKS PE=1 SV=4 - [MARCS_HUMAN] | 7 | 7 | 9 | 332 | 31.5 | 4.45 |
| Q15154 | Pericentriolar material 1 protein OS=Homo sapiens GN=PCM1 PE=1 SV=4 - [PCM1_HUMAN] | 7 | 4 | 5 | 2024 | 228.4 | 5.02 |
| P00966 | Argininosuccinate synthase OS=Homo sapiens GN=ASS1 PE=1 SV=2 - [ASSY_HUMAN] | 7 | 4 | 5 | 412 | 46.5 | 8.02 |
| O43776 | Asparaginyl-tRNA synthetase, cytoplasmic OS=Homo sapiens GN=NARS PE=1 SV=1 - [SYNC_HUMAN] | 7 | 4 | 5 | 548 | 62.9 | 6.25 |
| P50395 | Rab GDP dissociation inhibitor beta OS=Homo sapiens GN=GDI2 PE=1 SV=2 - [GDIB_HUMAN] | 7 | 10 | 12 | 445 | 50.6 | 6.47 |
| Q13435 | Splicing factor 3B subunit 2 OS=Homo sapiens GN=SF3B2 PE=1 SV=2 - [SF3B2_HUMAN] | 7 | 5 | 6 | 895 | 100.2 | 5.67 |
| O95202 | LETM1 and EF-hand domain-containing protein 1, mitochondrial OS=Homo sapiens GN=LETM1 PE=1 SV=1 - [LETM1_HUMAN] | 7 | 5 | 6 | 739 | 83.3 | 6.7 |
| Q5SSJ5 | Heterochromatin protein 1-binding protein 3 OS=Homo sapiens GN=HP1BP3 PE=1 SV=1 - [HP1B3_HUMAN] | 7 | 5 | 6 | 553 | 61.2 | 9.67 |
| Q99848 | Probable rRNA-processing protein EBP2 OS=Homo sapiens GN=EBNA1BP2 PE=1 SV=2 - [EBP2_HUMAN] | 7 | 5 | 6 | 306 | 34.8 | 10.1 |
| Q96HE7 | ERO1-like protein alpha OS=Homo sapiens GN=ERO1L PE=1 SV=2 - [ERO1A_HUMAN] | 7 | 5 | 6 | 468 | 54.4 | 5.68 |
| Q00688 | Peptidyl-prolyl cis-trans isomerase FKBP3 OS=Homo sapiens GN=FKBP3 PE=1 SV=1 - [FKBP3_HUMAN] | 7 | 5 | 6 | 224 | 25.2 | 9.28 |
| Q9UBB4 | Ataxin-10 OS=Homo sapiens GN=ATXN10 PE=1 SV=1 - [ATX10_HUMAN] | 7 | 6 | 7 | 475 | 53.5 | 5.25 |
| Q9UBE0 | SUMO-activating enzyme subunit 1 OS=Homo sapiens GN=SAE1 PE=1 SV=1 - [SAE1_HUMAN] | 7 | 6 | 7 | 346 | 38.4 | 5.3 |
| P60900 | Proteasome subunit alpha type-6 OS=Homo sapiens GN=PSMA6 PE=1 SV=1 - [PSA6_HUMAN] | 7 | 6 | 7 | 246 | 27.4 | 6.76 |
| P55036 | 26S proteasome non-ATPase regulatory subunit 4 OS=Homo sapiens GN=PSMD4 PE=1 SV=1 - [PSMD4_HUMAN] | 7 | 6 | 7 | 377 | 40.7 | 4.79 |
| Q99729 | Heterogeneous nuclear ribonucleoprotein A/B OS=Homo sapiens GN=HNRNPAB PE=1 SV=2 - [ROAA_HUMAN] | 7 | 8 | 9 | 332 | 36.2 | 8.21 |
| P62805 | Histone H4 OS=Homo sapiens GN=HIST1H4A PE=1 SV=2 - [H4_HUMAN] | 7 | 14 | 15 | 103 | 11.4 | 11.36 |
| Q9BXP5 | Serrate RNA effector molecule homolog OS=Homo sapiens GN=SRRT PE=1 SV=1 - [SRRT_HUMAN] | 7 | 8 | 8 | 876 | 100.6 | 5.96 |
| O00505 | Importin subunit alpha-3 OS=Homo sapiens GN=KPNA3 PE=1 SV=2 - [IMA3_HUMAN] | 7 | 7 | 7 | 521 | 57.8 | 4.94 |
| P61088 | Ubiquitin-conjugating enzyme E2 N OS=Homo sapiens GN=UBE2N PE=1 SV=1 - [UBE2N_HUMAN] | 7 | 7 | 7 | 152 | 17.1 | 6.57 |
| Q9Y2W1 | Thyroid hormone receptor-associated protein 3 OS=Homo sapiens GN=THRAP3 PE=1 SV=2 - [TR150_HUMAN] | 7 | 6 | 6 | 955 | 108.6 | 10.15 |
| P04843 | Dolichyl-diphosphooligosaccharide--protein glycosyltransferase subunit 1 OS=Homo sapiens GN=RPN1 PE=1 SV=1 - [RPN1_HUMAN] | 7 | 6 | 6 | 607 | 68.5 | 6.38 |
| O95747 | Serine/threonine-protein kinase OSR1 OS=Homo sapiens GN=OXSR1 PE=1 SV=1 - [OXSR1_HUMAN] | 7 | 6 | 6 | 527 | 58 | 6.43 |
| P04181 | Ornithine aminotransferase, mitochondrial OS=Homo sapiens GN=OAT PE=1 SV=1 - [OAT_HUMAN] | 7 | 6 | 6 | 439 | 48.5 | 7.03 |
| P39019 | 40S ribosomal protein S19 OS=Homo sapiens GN=RPS19 PE=1 SV=2 - [RS19_HUMAN] | 7 | 6 | 6 | 145 | 16.1 | 10.32 |
| P24534 | Elongation factor 1-beta OS=Homo sapiens GN=EEF1B2 PE=1 SV=3 - [EF1B_HUMAN] | 7 | 6 | 6 | 225 | 24.7 | 4.67 |
| Q96P70 | Importin-9 OS=Homo sapiens GN=IPO9 PE=1 SV=3 - [IPO9_HUMAN] | 7 | 5 | 5 | 1041 | 115.9 | 4.81 |
| Q96PK6 | RNA-binding protein 14 OS=Homo sapiens GN=RBM14 PE=1 SV=2 - [RBM14_HUMAN] | 7 | 5 | 5 | 669 | 69.4 | 9.67 |
| Q13347 | Eukaryotic translation initiation factor 3 subunit I OS=Homo sapiens GN=EIF3I PE=1 SV=1 - [EIF3I_HUMAN] | 7 | 5 | 5 | 325 | 36.5 | 5.64 |
| Q9BTV4 | Transmembrane protein 43 OS=Homo sapiens GN=TMEM43 PE=1 SV=1 - [TMM43_HUMAN] | 7 | 5 | 5 | 400 | 44.8 | 8.13 |
| P61981 | 14-3-3 protein gamma OS=Homo sapiens GN=YWHAG PE=1 SV=2 - [1433G_HUMAN] | 7 | 18 | 17 | 247 | 28.3 | 4.89 |
| Q13247 | Splicing factor, arginine/serine-rich 6 OS=Homo sapiens GN=SFRS6 PE=1 SV=2 - [SFRS6_HUMAN] | 7 | 9 | 8 | 344 | 39.6 | 11.43 |
| P62241 | 40S ribosomal protein S8 OS=Homo sapiens GN=RPS8 PE=1 SV=2 - [RS8_HUMAN] | 7 | 7 | 6 | 208 | 24.2 | 10.32 |
| P61201 | COP9 signalosome complex subunit 2 OS=Homo sapiens GN=COPS2 PE=1 SV=1 - [CSN2_HUMAN] | 7 | 6 | 5 | 443 | 51.6 | 5.53 |
| P46459 | Vesicle-fusing ATPase OS=Homo sapiens GN=NSF PE=1 SV=3 - [NSF_HUMAN] | 7 | 5 | 4 | 744 | 82.5 | 6.95 |
| Q13310 | Polyadenylate-binding protein 4 OS=Homo sapiens GN=PABPC4 PE=1 SV=1 - [PABP4_HUMAN] | 7 | 13 | 10 | 644 | 70.7 | 9.26 |
| P51114 | Fragile X mental retardation syndrome-related protein 1 OS=Homo sapiens GN=FXR1 PE=1 SV=3 - [FXR1_HUMAN] | 7 | 7 | 5 | 621 | 69.7 | 6.15 |
| P62333 | 26S protease regulatory subunit S10B OS=Homo sapiens GN=PSMC6 PE=1 SV=1 - [PRS10_HUMAN] | 7 | 10 | 7 | 389 | 44.1 | 7.49 |
| Q06323 | Proteasome activator complex subunit 1 OS=Homo sapiens GN=PSME1 PE=1 SV=1 - [PSME1_HUMAN] | 7 | 7 | 4 | 249 | 28.7 | 6.02 |
| P23526 | Adenosylhomocysteinase OS=Homo sapiens GN=AHCY PE=1 SV=4 - [SAHH_HUMAN] | 7 | 8 | 4 | 432 | 47.7 | 6.34 |
| Q99543 | DnaJ homolog subfamily C member 2 OS=Homo sapiens GN=DNAJC2 PE=1 SV=4 - [DNJC2_HUMAN] | 6 | 0 | 6 | 621 | 72 | 8.7 |
| Q9Y520 | Protein PRRC2C OS=Homo sapiens GN=PRRC2C PE=1 SV=4 - [PRC2C_HUMAN] | 6 | 1 | 6 | 2896 | 316.7 | 9.13 |
| O00451 | GDNF family receptor alpha-2 OS=Homo sapiens GN=GFRA2 PE=2 SV=2 - [GFRA2_HUMAN] | 6 | 1 | 6 | 464 | 51.5 | 7.66 |
| Q9BVJ6 | U3 small nucleolar RNA-associated protein 14 homolog A OS=Homo sapiens GN=UTP14A PE=1 SV=1 - [UT14A_HUMAN] | 6 | 1 | 6 | 771 | 87.9 | 7.87 |
| Q8IWX8 | Calcium homeostasis endoplasmic reticulum protein OS=Homo sapiens GN=CHERP PE=1 SV=3 - [CHERP_HUMAN] | 6 | 1 | 6 | 916 | 103.6 | 9.04 |
| P21283 | V-type proton ATPase subunit C 1 OS=Homo sapiens GN=ATP6V1C1 PE=1 SV=4 - [VATC1_HUMAN] | 6 | 1 | 6 | 382 | 43.9 | 7.46 |
| P27635 | 60S ribosomal protein L10 OS=Homo sapiens GN=RPL10 PE=1 SV=4 - [RL10_HUMAN] | 6 | 1 | 5 | 214 | 24.6 | 10.08 |
| Q9UQ35 | Serine/arginine repetitive matrix protein 2 OS=Homo sapiens GN=SRRM2 PE=1 SV=2 - [SRRM2_HUMAN] | 6 | 2 | 7 | 2752 | 299.4 | 12.06 |
| Q9UKV3 | Apoptotic chromatin condensation inducer in the nucleus OS=Homo sapiens GN=ACIN1 PE=1 SV=2 - [ACINU_HUMAN] | 6 | 2 | 7 | 1341 | 151.8 | 6.43 |
| P30520 | Adenylosuccinate synthetase isozyme 2 OS=Homo sapiens GN=ADSS PE=1 SV=3 - [PURA2_HUMAN] | 6 | 2 | 7 | 456 | 50.1 | 6.55 |
| Q8NE71 | ATP-binding cassette sub-family F member 1 OS=Homo sapiens GN=ABCF1 PE=1 SV=2 - [ABCF1_HUMAN] | 6 | 2 | 6 | 845 | 95.9 | 6.8 |
| P53634 | Dipeptidyl peptidase 1 OS=Homo sapiens GN=CTSC PE=1 SV=2 - [CATC_HUMAN] | 6 | 2 | 6 | 463 | 51.8 | 6.99 |
| O00264 | Membrane-associated progesterone receptor component 1 OS=Homo sapiens GN=PGRMC1 PE=1 SV=3 - [PGRC1_HUMAN] | 6 | 2 | 6 | 195 | 21.7 | 4.7 |
| Q8NC51 | Plasminogen activator inhibitor 1 RNA-binding protein OS=Homo sapiens GN=SERBP1 PE=1 SV=2 - [PAIRB_HUMAN] | 6 | 3 | 8 | 408 | 44.9 | 8.65 |
| O43809 | Cleavage and polyadenylation specificity factor subunit 5 OS=Homo sapiens GN=NUDT21 PE=1 SV=1 - [CPSF5_HUMAN] | 6 | 3 | 8 | 227 | 26.2 | 8.82 |
| P25787 | Proteasome subunit alpha type-2 OS=Homo sapiens GN=PSMA2 PE=1 SV=2 - [PSA2_HUMAN] | 6 | 4 | 10 | 234 | 25.9 | 7.43 |
| P49792 | E3 SUMO-protein ligase RanBP2 OS=Homo sapiens GN=RANBP2 PE=1 SV=2 - [RBP2_HUMAN] | 6 | 2 | 5 | 3224 | 358 | 6.2 |
| Q99829 | Copine-1 OS=Homo sapiens GN=CPNE1 PE=1 SV=1 - [CPNE1_HUMAN] | 6 | 2 | 5 | 537 | 59 | 5.83 |
| P13591 | Neural cell adhesion molecule 1 OS=Homo sapiens GN=NCAM1 PE=1 SV=3 - [NCAM1_HUMAN] | 6 | 2 | 5 | 858 | 94.5 | 4.87 |
| Q13148 | TAR DNA-binding protein 43 OS=Homo sapiens GN=TARDBP PE=1 SV=1 - [TADBP_HUMAN] | 6 | 3 | 7 | 414 | 44.7 | 6.19 |
| Q8NEJ9 | Neuroguidin OS=Homo sapiens GN=NGDN PE=1 SV=1 - [NGDN_HUMAN] | 6 | 3 | 7 | 315 | 35.9 | 9.57 |
| Q96KB5 | Lymphokine-activated killer T-cell-originated protein kinase OS=Homo sapiens GN=PBK PE=1 SV=3 - [TOPK_HUMAN] | 6 | 3 | 7 | 322 | 36.1 | 5.12 |
| O75340 | Programmed cell death protein 6 OS=Homo sapiens GN=PDCD6 PE=1 SV=1 - [PDCD6_HUMAN] | 6 | 3 | 7 | 191 | 21.9 | 5.4 |
| P17677 | Neuromodulin OS=Homo sapiens GN=GAP43 PE=1 SV=1 - [NEUM_HUMAN] | 6 | 3 | 7 | 238 | 24.8 | 4.72 |
| O14979 | Heterogeneous nuclear ribonucleoprotein D-like OS=Homo sapiens GN=HNRPDL PE=1 SV=3 - [HNRDL_HUMAN] | 6 | 5 | 10 | 420 | 46.4 | 9.57 |
| Q5SW79 | Centrosomal protein of 170 kDa OS=Homo sapiens GN=CEP170 PE=1 SV=1 - [CE170_HUMAN] | 6 | 3 | 6 | 1584 | 175.2 | 7.11 |
| Q02809 | Procollagen-lysine,2-oxoglutarate 5-dioxygenase 1 OS=Homo sapiens GN=PLOD1 PE=1 SV=2 - [PLOD1_HUMAN] | 6 | 3 | 6 | 727 | 83.5 | 6.95 |
| Q7L2H7 | Eukaryotic translation initiation factor 3 subunit M OS=Homo sapiens GN=EIF3M PE=1 SV=1 - [EIF3M_HUMAN] | 6 | 3 | 6 | 374 | 42.5 | 5.63 |
| Q96PZ0 | Pseudouridylate synthase 7 homolog OS=Homo sapiens GN=PUS7 PE=1 SV=2 - [PUS7_HUMAN] | 6 | 3 | 6 | 661 | 75 | 6.37 |
| Q96KP4 | Cytosolic non-specific dipeptidase OS=Homo sapiens GN=CNDP2 PE=1 SV=2 - [CNDP2_HUMAN] | 6 | 3 | 6 | 475 | 52.8 | 5.97 |
| P49720 | Proteasome subunit beta type-3 OS=Homo sapiens GN=PSMB3 PE=1 SV=2 - [PSB3_HUMAN] | 6 | 3 | 6 | 205 | 22.9 | 6.55 |
| Q15631 | Translin OS=Homo sapiens GN=TSN PE=1 SV=1 - [TSN_HUMAN] | 6 | 3 | 6 | 228 | 26.2 | 6.44 |
| Q16881 | Thioredoxin reductase 1, cytoplasmic OS=Homo sapiens GN=TXNRD1 PE=1 SV=3 - [TRXR1_HUMAN] | 6 | 2 | 4 | 649 | 70.9 | 7.39 |
| Q9BWF3 | RNA-binding protein 4 OS=Homo sapiens GN=RBM4 PE=1 SV=1 - [RBM4_HUMAN] | 6 | 5 | 9 | 364 | 40.3 | 7.08 |
| Q15185 | Prostaglandin E synthase 3 OS=Homo sapiens GN=PTGES3 PE=1 SV=1 - [TEBP_HUMAN] | 6 | 5 | 9 | 160 | 18.7 | 4.54 |
| P39687 | Acidic leucine-rich nuclear phosphoprotein 32 family member A OS=Homo sapiens GN=ANP32A PE=1 SV=1 - [AN32A_HUMAN] | 6 | 9 | 16 | 249 | 28.6 | 4.09 |
| Q9P2K5 | Myelin expression factor 2 OS=Homo sapiens GN=MYEF2 PE=1 SV=3 - [MYEF2_HUMAN] | 6 | 4 | 7 | 600 | 64.1 | 8.75 |
| Q04760 | Lactoylglutathione lyase OS=Homo sapiens GN=GLO1 PE=1 SV=4 - [LGUL_HUMAN] | 6 | 4 | 7 | 184 | 20.8 | 5.31 |
| O75489 | NADH dehydrogenase [ubiquinone] iron-sulfur protein 3, mitochondrial OS=Homo sapiens GN=NDUFS3 PE=1 SV=1 - [NDUS3_HUMAN] | 6 | 6 | 10 | 264 | 30.2 | 7.5 |
| P25398 | 40S ribosomal protein S12 OS=Homo sapiens GN=RPS12 PE=1 SV=3 - [RS12_HUMAN] | 6 | 6 | 10 | 132 | 14.5 | 7.21 |
| Q96A33 | Coiled-coil domain-containing protein 47 OS=Homo sapiens GN=CCDC47 PE=1 SV=1 - [CCD47_HUMAN] | 6 | 3 | 5 | 483 | 55.8 | 4.87 |
| Q9UHX1 | Poly(U)-binding-splicing factor PUF60 OS=Homo sapiens GN=PUF60 PE=1 SV=1 - [PUF60_HUMAN] | 6 | 3 | 5 | 559 | 59.8 | 5.29 |
| Q86V81 | THO complex subunit 4 OS=Homo sapiens GN=THOC4 PE=1 SV=3 - [THOC4_HUMAN] | 6 | 5 | 8 | 257 | 26.9 | 11.15 |
| P63010 | AP-2 complex subunit beta OS=Homo sapiens GN=AP2B1 PE=1 SV=1 - [AP2B1_HUMAN] | 6 | 7 | 11 | 937 | 104.5 | 5.38 |
| P30048 | Thioredoxin-dependent peroxide reductase, mitochondrial OS=Homo sapiens GN=PRDX3 PE=1 SV=3 - [PRDX3_HUMAN] | 6 | 6 | 9 | 256 | 27.7 | 7.78 |
| P14314 | Glucosidase 2 subunit beta OS=Homo sapiens GN=PRKCSH PE=1 SV=2 - [GLU2B_HUMAN] | 6 | 4 | 6 | 528 | 59.4 | 4.41 |
| Q9NZL9 | Methionine adenosyltransferase 2 subunit beta OS=Homo sapiens GN=MAT2B PE=1 SV=1 - [MAT2B_HUMAN] | 6 | 4 | 6 | 334 | 37.5 | 7.36 |
| P56537 | Eukaryotic translation initiation factor 6 OS=Homo sapiens GN=EIF6 PE=1 SV=1 - [IF6_HUMAN] | 6 | 4 | 6 | 245 | 26.6 | 4.68 |
| Q96F85 | CB1 cannabinoid receptor-interacting protein 1 OS=Homo sapiens GN=CNRIP1 PE=1 SV=1 - [CNRP1_HUMAN] | 6 | 4 | 6 | 164 | 18.6 | 7.94 |
| P63241 | Eukaryotic translation initiation factor 5A-1 OS=Homo sapiens GN=EIF5A PE=1 SV=2 - [IF5A1_HUMAN] | 6 | 7 | 10 | 154 | 16.8 | 5.24 |
| P35613 | Basigin OS=Homo sapiens GN=BSG PE=1 SV=2 - [BASI_HUMAN] | 6 | 5 | 7 | 385 | 42.2 | 5.66 |
| Q96FW1 | Ubiquitin thioesterase OTUB1 OS=Homo sapiens GN=OTUB1 PE=1 SV=2 - [OTUB1_HUMAN] | 6 | 5 | 7 | 271 | 31.3 | 4.94 |
| P16949 | Stathmin OS=Homo sapiens GN=STMN1 PE=1 SV=3 - [STMN1_HUMAN] | 6 | 5 | 7 | 149 | 17.3 | 5.97 |
| Q01469 | Fatty acid-binding protein, epidermal OS=Homo sapiens GN=FABP5 PE=1 SV=3 - [FABP5_HUMAN] | 6 | 5 | 7 | 135 | 15.2 | 7.01 |
| P30050 | 60S ribosomal protein L12 OS=Homo sapiens GN=RPL12 PE=1 SV=1 - [RL12_HUMAN] | 6 | 8 | 11 | 165 | 17.8 | 9.42 |
| P67936 | Tropomyosin alpha-4 chain OS=Homo sapiens GN=TPM4 PE=1 SV=3 - [TPM4_HUMAN] | 6 | 9 | 12 | 248 | 28.5 | 4.69 |
| Q92922 | SWI/SNF complex subunit SMARCC1 OS=Homo sapiens GN=SMARCC1 PE=1 SV=3 - [SMRC1_HUMAN] | 6 | 6 | 8 | 1105 | 122.8 | 5.76 |
| O75390 | Citrate synthase, mitochondrial OS=Homo sapiens GN=CS PE=1 SV=2 - [CISY_HUMAN] | 6 | 6 | 8 | 466 | 51.7 | 8.32 |
| P38919 | Eukaryotic initiation factor 4A-III OS=Homo sapiens GN=EIF4A3 PE=1 SV=4 - [IF4A3_HUMAN] | 6 | 6 | 8 | 411 | 46.8 | 6.73 |
| P41252 | Isoleucyl-tRNA synthetase, cytoplasmic OS=Homo sapiens GN=IARS PE=1 SV=2 - [SYIC_HUMAN] | 6 | 3 | 4 | 1262 | 144.4 | 6.15 |
| Q9Y678 | Coatomer subunit gamma OS=Homo sapiens GN=COPG PE=1 SV=1 - [COPG_HUMAN] | 6 | 3 | 4 | 874 | 97.7 | 5.47 |
| Q9H2J4 | Phosducin-like protein 3 OS=Homo sapiens GN=PDCL3 PE=1 SV=1 - [PDCL3_HUMAN] | 6 | 3 | 4 | 239 | 27.6 | 4.84 |
| P15311 | Ezrin OS=Homo sapiens GN=EZR PE=1 SV=4 - [EZRI_HUMAN] | 6 | 4 | 5 | 586 | 69.4 | 6.27 |
| Q53FA7 | Quinone oxidoreductase PIG3 OS=Homo sapiens GN=TP53I3 PE=1 SV=2 - [QORX_HUMAN] | 6 | 4 | 5 | 332 | 35.5 | 7.17 |
| Q9Y617 | Phosphoserine aminotransferase OS=Homo sapiens GN=PSAT1 PE=1 SV=2 - [SERC_HUMAN] | 6 | 4 | 5 | 370 | 40.4 | 7.66 |
| Q15293 | Reticulocalbin-1 OS=Homo sapiens GN=RCN1 PE=1 SV=1 - [RCN1_HUMAN] | 6 | 4 | 5 | 331 | 38.9 | 5 |
| Q9NXG2 | THUMP domain-containing protein 1 OS=Homo sapiens GN=THUMPD1 PE=1 SV=2 - [THUM1_HUMAN] | 6 | 4 | 5 | 353 | 39.3 | 7.88 |
| P37198 | Nuclear pore glycoprotein p62 OS=Homo sapiens GN=NUP62 PE=1 SV=3 - [NUP62_HUMAN] | 6 | 5 | 6 | 522 | 53.2 | 5.31 |
| Q14498 | RNA-binding protein 39 OS=Homo sapiens GN=RBM39 PE=1 SV=2 - [RBM39_HUMAN] | 6 | 5 | 6 | 530 | 59.3 | 10.1 |
| Q9BZZ5 | Apoptosis inhibitor 5 OS=Homo sapiens GN=API5 PE=1 SV=3 - [API5_HUMAN] | 6 | 5 | 6 | 524 | 59 | 7.34 |
| P46109 | Crk-like protein OS=Homo sapiens GN=CRKL PE=1 SV=1 - [CRKL_HUMAN] | 6 | 5 | 6 | 303 | 33.8 | 6.74 |
| P24666 | Low molecular weight phosphotyrosine protein phosphatase OS=Homo sapiens GN=ACP1 PE=1 SV=3 - [PPAC_HUMAN] | 6 | 5 | 6 | 158 | 18 | 6.74 |
| P10515 | Dihydrolipoyllysine-residue acetyltransferase component of pyruvate dehydrogenase complex, mitochondrial OS=Homo sapiens GN=DLAT PE=1 SV=3 - [ODP2_HUMAN] | 6 | 6 | 7 | 647 | 69 | 7.84 |
| O94925 | Glutaminase kidney isoform, mitochondrial OS=Homo sapiens GN=GLS PE=1 SV=1 - [GLSK_HUMAN] | 6 | 6 | 7 | 669 | 73.4 | 7.77 |
| Q15233 | Non-POU domain-containing octamer-binding protein OS=Homo sapiens GN=NONO PE=1 SV=4 - [NONO_HUMAN] | 6 | 7 | 8 | 471 | 54.2 | 8.95 |
| Q14257 | Reticulocalbin-2 OS=Homo sapiens GN=RCN2 PE=1 SV=1 - [RCN2_HUMAN] | 6 | 7 | 8 | 317 | 36.9 | 4.4 |
| P52907 | F-actin-capping protein subunit alpha-1 OS=Homo sapiens GN=CAPZA1 PE=1 SV=3 - [CAZA1_HUMAN] | 6 | 9 | 9 | 286 | 32.9 | 5.69 |
| O75874 | Isocitrate dehydrogenase [NADP] cytoplasmic OS=Homo sapiens GN=IDH1 PE=1 SV=2 - [IDHC_HUMAN] | 6 | 8 | 8 | 414 | 46.6 | 7.01 |
| P30084 | Enoyl-CoA hydratase, mitochondrial OS=Homo sapiens GN=ECHS1 PE=1 SV=4 - [ECHM_HUMAN] | 6 | 8 | 8 | 290 | 31.4 | 8.07 |
| O95232 | Luc7-like protein 3 OS=Homo sapiens GN=LUC7L3 PE=1 SV=2 - [LC7L3_HUMAN] | 6 | 7 | 7 | 432 | 51.4 | 9.79 |
| P62906 | 60S ribosomal protein L10a OS=Homo sapiens GN=RPL10A PE=1 SV=2 - [RL10A_HUMAN] | 6 | 7 | 7 | 217 | 24.8 | 9.94 |
| Q00796 | Sorbitol dehydrogenase OS=Homo sapiens GN=SORD PE=1 SV=4 - [DHSO_HUMAN] | 6 | 6 | 6 | 357 | 38.3 | 7.97 |
| P20042 | Eukaryotic translation initiation factor 2 subunit 2 OS=Homo sapiens GN=EIF2S2 PE=1 SV=2 - [IF2B_HUMAN] | 6 | 6 | 6 | 333 | 38.4 | 5.8 |
| P46379 | Large proline-rich protein BAT3 OS=Homo sapiens GN=BAT3 PE=1 SV=2 - [BAT3_HUMAN] | 6 | 5 | 5 | 1132 | 119.3 | 5.6 |
| Q16698 | 2,4-dienoyl-CoA reductase, mitochondrial OS=Homo sapiens GN=DECR1 PE=1 SV=1 - [DECR_HUMAN] | 6 | 5 | 5 | 335 | 36 | 9.28 |
| Q9BS26 | Endoplasmic reticulum resident protein 44 OS=Homo sapiens GN=ERP44 PE=1 SV=1 - [ERP44_HUMAN] | 6 | 5 | 5 | 406 | 46.9 | 5.26 |
| P62195 | 26S protease regulatory subunit 8 OS=Homo sapiens GN=PSMC5 PE=1 SV=1 - [PRS8_HUMAN] | 6 | 5 | 5 | 406 | 45.6 | 7.55 |
| O15067 | Phosphoribosylformylglycinamidine synthase OS=Homo sapiens GN=PFAS PE=1 SV=4 - [PUR4_HUMAN] | 6 | 4 | 4 | 1338 | 144.6 | 5.76 |
| O94906 | Pre-mRNA-processing factor 6 OS=Homo sapiens GN=PRPF6 PE=1 SV=1 - [PRP6_HUMAN] | 6 | 4 | 4 | 941 | 106.9 | 8.25 |
| O75351 | Vacuolar protein sorting-associated protein 4B OS=Homo sapiens GN=VPS4B PE=1 SV=2 - [VPS4B_HUMAN] | 6 | 4 | 4 | 444 | 49.3 | 7.23 |
| Q16531 | DNA damage-binding protein 1 OS=Homo sapiens GN=DDB1 PE=1 SV=1 - [DDB1_HUMAN] | 6 | 3 | 3 | 1140 | 126.9 | 5.26 |
| Q9H4A4 | Aminopeptidase B OS=Homo sapiens GN=RNPEP PE=1 SV=2 - [AMPB_HUMAN] | 6 | 3 | 3 | 650 | 72.5 | 5.74 |
| O14813 | Paired mesoderm homeobox protein 2A OS=Homo sapiens GN=PHOX2A PE=1 SV=2 - [PHX2A_HUMAN] | 6 | 3 | 3 | 284 | 29.6 | 8.76 |
| P16401 | Histone H1.5 OS=Homo sapiens GN=HIST1H1B PE=1 SV=3 - [H15_HUMAN] | 6 | 10 | 9 | 226 | 22.6 | 10.92 |
| Q01105 | Protein SET OS=Homo sapiens GN=SET PE=1 SV=3 - [SET_HUMAN] | 6 | 8 | 7 | 290 | 33.5 | 4.32 |
| Q8TAT6 | Nuclear protein localization protein 4 homolog OS=Homo sapiens GN=NPLOC4 PE=1 SV=3 - [NPL4_HUMAN] | 6 | 7 | 6 | 608 | 68.1 | 6.38 |
| Q16851 | UTP--glucose-1-phosphate uridylyltransferase OS=Homo sapiens GN=UGP2 PE=1 SV=5 - [UGPA_HUMAN] | 6 | 6 | 5 | 508 | 56.9 | 8.15 |
| O75306 | NADH dehydrogenase [ubiquinone] iron-sulfur protein 2, mitochondrial OS=Homo sapiens GN=NDUFS2 PE=1 SV=2 - [NDUS2_HUMAN] | 6 | 6 | 5 | 463 | 52.5 | 7.55 |
| P30566 | Adenylosuccinate lyase OS=Homo sapiens GN=ADSL PE=1 SV=2 - [PUR8_HUMAN] | 6 | 6 | 5 | 484 | 54.9 | 7.11 |
| Q5RKV6 | Exosome complex exonuclease MTR3 OS=Homo sapiens GN=EXOSC6 PE=1 SV=1 - [EXOS6_HUMAN] | 6 | 6 | 5 | 272 | 28.2 | 6.28 |
| Q04917 | 14-3-3 protein eta OS=Homo sapiens GN=YWHAH PE=1 SV=4 - [1433F_HUMAN] | 6 | 15 | 12 | 246 | 28.2 | 4.84 |
| Q9H9A6 | Leucine-rich repeat-containing protein 40 OS=Homo sapiens GN=LRRC40 PE=1 SV=1 - [LRC40_HUMAN] | 6 | 5 | 4 | 602 | 68.2 | 6.43 |
| P17987 | T-complex protein 1 subunit alpha OS=Homo sapiens GN=TCP1 PE=1 SV=1 - [TCPA_HUMAN] | 6 | 5 | 4 | 556 | 60.3 | 6.11 |
| P13674 | Prolyl 4-hydroxylase subunit alpha-1 OS=Homo sapiens GN=P4HA1 PE=1 SV=2 - [P4HA1_HUMAN] | 6 | 5 | 4 | 534 | 61 | 6.01 |
| P62424 | 60S ribosomal protein L7a OS=Homo sapiens GN=RPL7A PE=1 SV=2 - [RL7A_HUMAN] | 6 | 5 | 4 | 266 | 30 | 10.61 |
| O95336 | 6-phosphogluconolactonase OS=Homo sapiens GN=PGLS PE=1 SV=2 - [6PGL_HUMAN] | 6 | 5 | 4 | 258 | 27.5 | 6.05 |
| P09382 | Galectin-1 OS=Homo sapiens GN=LGALS1 PE=1 SV=2 - [LEG1_HUMAN] | 6 | 5 | 4 | 135 | 14.7 | 5.5 |
| Q14008 | Cytoskeleton-associated protein 5 OS=Homo sapiens GN=CKAP5 PE=1 SV=3 - [CKAP5_HUMAN] | 6 | 4 | 3 | 2032 | 225.4 | 7.8 |
| P02768 | Serum albumin OS=Homo sapiens GN=ALB PE=1 SV=2 - [ALBU_HUMAN] | 6 | 15 | 11 | 609 | 69.3 | 6.28 |
| P62191 | 26S protease regulatory subunit 4 OS=Homo sapiens GN=PSMC1 PE=1 SV=1 - [PRS4_HUMAN] | 6 | 7 | 5 | 440 | 49.2 | 6.21 |
| P10768 | S-formylglutathione hydrolase OS=Homo sapiens GN=ESD PE=1 SV=2 - [ESTD_HUMAN] | 6 | 7 | 5 | 282 | 31.4 | 7.02 |
| Q8WVM8 | Sec1 family domain-containing protein 1 OS=Homo sapiens GN=SCFD1 PE=1 SV=4 - [SCFD1_HUMAN] | 6 | 5 | 3 | 642 | 72.3 | 6.27 |
| Q9P2J5 | Leucyl-tRNA synthetase, cytoplasmic OS=Homo sapiens GN=LARS PE=1 SV=2 - [SYLC_HUMAN] | 6 | 6 | 3 | 1176 | 134.4 | 7.3 |
| Q96T88 | E3 ubiquitin-protein ligase UHRF1 OS=Homo sapiens GN=UHRF1 PE=1 SV=1 - [UHRF1_HUMAN] | 6 | 6 | 3 | 793 | 89.8 | 7.56 |
| Q13098 | COP9 signalosome complex subunit 1 OS=Homo sapiens GN=GPS1 PE=1 SV=4 - [CSN1_HUMAN] | 6 | 6 | 3 | 491 | 55.5 | 6.74 |
| Q14683 | Structural maintenance of chromosomes protein 1A OS=Homo sapiens GN=SMC1A PE=1 SV=2 - [SMC1A_HUMAN] | 6 | 4 | 2 | 1233 | 143.1 | 7.64 |
| P17655 | Calpain-2 catalytic subunit OS=Homo sapiens GN=CAPN2 PE=1 SV=6 - [CAN2_HUMAN] | 6 | 4 | 2 | 700 | 79.9 | 4.98 |
| P41223 | Protein BUD31 homolog OS=Homo sapiens GN=BUD31 PE=1 SV=2 - [BUD31_HUMAN] | 5 | 0 | 5 | 144 | 17 | 8.82 |
| Q53EL6 | Programmed cell death protein 4 OS=Homo sapiens GN=PDCD4 PE=1 SV=2 - [PDCD4_HUMAN] | 5 | 0 | 5 | 469 | 51.7 | 5.21 |
| Q13907 | Isopentenyl-diphosphate Delta-isomerase 1 OS=Homo sapiens GN=IDI1 PE=1 SV=2 - [IDI1_HUMAN] | 5 | 1 | 7 | 227 | 26.3 | 6.34 |
| P25713 | Metallothionein-3, Growth inhibitory factor, OS=Homo sapiens GN=MT3 PE=1 SV=2 - [MT3_HUMAN] | 5 | 1 | 7 | 211 | 6.5 | 5.36 |
| Q9UBQ5 | Eukaryotic translation initiation factor 3 subunit K OS=Homo sapiens GN=EIF3K PE=1 SV=1 - [EIF3K_HUMAN] | 5 | 1 | 7 | 218 | 25 | 4.93 |
| Q96C86 | m7GpppX diphosphatase OS=Homo sapiens GN=DCPS PE=1 SV=2 - [DCPS_HUMAN] | 5 | 1 | 6 | 337 | 38.6 | 6.38 |
| O94903 | Proline synthetase co-transcribed bacterial homolog protein OS=Homo sapiens GN=PROSC PE=1 SV=1 - [PROSC_HUMAN] | 5 | 1 | 6 | 275 | 30.3 | 7.5 |
| Q5QJE6 | Deoxynucleotidyltransferase terminal-interacting protein 2 OS=Homo sapiens GN=DNTTIP2 PE=1 SV=2 - [TDIF2_HUMAN] | 5 | 1 | 5 | 756 | 84.4 | 6.16 |
| Q9BYT8 | Neurolysin, mitochondrial OS=Homo sapiens GN=NLN PE=1 SV=1 - [NEUL_HUMAN] | 5 | 1 | 5 | 704 | 80.6 | 6.64 |
| Q13451 | Peptidyl-prolyl cis-trans isomerase FKBP5 OS=Homo sapiens GN=FKBP5 PE=1 SV=2 - [FKBP5_HUMAN] | 5 | 1 | 5 | 457 | 51.2 | 5.9 |
| P55010 | Eukaryotic translation initiation factor 5 OS=Homo sapiens GN=EIF5 PE=1 SV=2 - [IF5_HUMAN] | 5 | 1 | 5 | 431 | 49.2 | 5.58 |
| Q9H2W6 | 39S ribosomal protein L46, mitochondrial OS=Homo sapiens GN=MRPL46 PE=1 SV=1 - [RM46_HUMAN] | 5 | 1 | 5 | 279 | 31.7 | 7.05 |
| P35269 | General transcription factor IIF subunit 1 OS=Homo sapiens GN=GTF2F1 PE=1 SV=2 - [T2FA_HUMAN] | 5 | 1 | 5 | 517 | 58.2 | 7.49 |
| Q8WZA9 | Immunity-related GTPase family Q protein OS=Homo sapiens GN=IRGQ PE=1 SV=1 - [IRGQ_HUMAN] | 5 | 1 | 5 | 623 | 62.7 | 4.88 |
| P49721 | Proteasome subunit beta type-2 OS=Homo sapiens GN=PSMB2 PE=1 SV=1 - [PSB2_HUMAN] | 5 | 1 | 5 | 201 | 22.8 | 7.02 |
| Q9BSD7 | Nucleoside-triphosphatase C1orf57 OS=Homo sapiens GN=C1orf57 PE=1 SV=1 - [CA057_HUMAN] | 5 | 1 | 5 | 190 | 20.7 | 9.54 |
| Q6IAA8 | RhoA activator C11orf59 OS=Homo sapiens GN=C11orf59 PE=1 SV=2 - [CK059_HUMAN] | 5 | 1 | 5 | 161 | 17.7 | 5.15 |
| P43487 | Ran-specific GTPase-activating protein OS=Homo sapiens GN=RANBP1 PE=1 SV=1 - [RANG_HUMAN] | 5 | 2 | 8 | 201 | 23.3 | 5.29 |
| Q15021 | Condensin complex subunit 1 OS=Homo sapiens GN=NCAPD2 PE=1 SV=3 - [CND1_HUMAN] | 5 | 1 | 4 | 1401 | 157.1 | 6.61 |
| O95793 | Double-stranded RNA-binding protein Staufen homolog 1 OS=Homo sapiens GN=STAU1 PE=1 SV=2 - [STAU1_HUMAN] | 5 | 1 | 4 | 577 | 63.1 | 9.44 |
| P12109 | Collagen alpha-1(VI) chain OS=Homo sapiens GN=COL6A1 PE=1 SV=3 - [CO6A1_HUMAN] | 5 | 1 | 4 | 1028 | 108.5 | 5.43 |
| Q9Y5X3 | Sorting nexin-5 OS=Homo sapiens GN=SNX5 PE=1 SV=1 - [SNX5_HUMAN] | 5 | 1 | 4 | 404 | 46.8 | 6.76 |
| P62633 | Cellular nucleic acid-binding protein OS=Homo sapiens GN=CNBP PE=1 SV=1 - [CNBP_HUMAN] | 5 | 2 | 7 | 177 | 19.4 | 7.71 |
| P20290 | Transcription factor BTF3 OS=Homo sapiens GN=BTF3 PE=1 SV=1 - [BTF3_HUMAN] | 5 | 3 | 9 | 206 | 22.2 | 9.38 |
| Q13596 | Sorting nexin-1 OS=Homo sapiens GN=SNX1 PE=1 SV=3 - [SNX1_HUMAN] | 5 | 2 | 6 | 522 | 59 | 5.15 |
| Q92522 | Histone H1x OS=Homo sapiens GN=H1FX PE=1 SV=1 - [H1X_HUMAN] | 5 | 2 | 6 | 213 | 22.5 | 10.76 |
| Q9BTT0 | Acidic leucine-rich nuclear phosphoprotein 32 family member E OS=Homo sapiens GN=ANP32E PE=1 SV=1 - [AN32E_HUMAN] | 5 | 3 | 8 | 268 | 30.7 | 3.85 |
| Q9NQX3 | Gephyrin OS=Homo sapiens GN=GPHN PE=1 SV=1 - [GEPH_HUMAN] | 5 | 2 | 5 | 736 | 79.7 | 5.43 |
| P39656 | Dolichyl-diphosphooligosaccharide--protein glycosyltransferase 48 kDa subunit OS=Homo sapiens GN=DDOST PE=1 SV=4 - [OST48_HUMAN] | 5 | 2 | 5 | 456 | 50.8 | 6.55 |
| Q9C0B1 | Protein fto OS=Homo sapiens GN=FTO PE=1 SV=3 - [FTO_HUMAN] | 5 | 2 | 5 | 505 | 58.2 | 5.22 |
| P37268 | Squalene synthase OS=Homo sapiens GN=FDFT1 PE=1 SV=1 - [FDFT_HUMAN] | 5 | 2 | 5 | 417 | 48.1 | 6.54 |
| P12532 | Creatine kinase U-type, mitochondrial OS=Homo sapiens GN=CKMT1A PE=1 SV=1 - [KCRU_HUMAN] | 5 | 2 | 5 | 417 | 47 | 8.34 |
| P25788 | Proteasome subunit alpha type-3 OS=Homo sapiens GN=PSMA3 PE=1 SV=2 - [PSA3_HUMAN] | 5 | 2 | 5 | 255 | 28.4 | 5.33 |
| P60660 | Myosin light polypeptide 6 OS=Homo sapiens GN=MYL6 PE=1 SV=2 - [MYL6_HUMAN] | 5 | 2 | 5 | 151 | 16.9 | 4.65 |
| P49756 | RNA-binding protein 25 OS=Homo sapiens GN=RBM25 PE=1 SV=3 - [RBM25_HUMAN] | 5 | 3 | 7 | 843 | 100.1 | 6.32 |
| Q96I25 | Splicing factor 45 OS=Homo sapiens GN=RBM17 PE=1 SV=1 - [SPF45_HUMAN] | 5 | 3 | 7 | 401 | 44.9 | 5.97 |
| Q13573 | SNW domain-containing protein 1 OS=Homo sapiens GN=SNW1 PE=1 SV=1 - [SNW1_HUMAN] | 5 | 3 | 7 | 536 | 61.5 | 9.52 |
| P28066 | Proteasome subunit alpha type-5 OS=Homo sapiens GN=PSMA5 PE=1 SV=3 - [PSA5_HUMAN] | 5 | 3 | 7 | 241 | 26.4 | 4.79 |
| P05387 | 60S acidic ribosomal protein P2 OS=Homo sapiens GN=RPLP2 PE=1 SV=1 - [RLA2_HUMAN] | 5 | 4 | 8 | 115 | 11.7 | 4.54 |
| Q8TEX9 | Importin-4 OS=Homo sapiens GN=IPO4 PE=1 SV=2 - [IPO4_HUMAN] | 5 | 3 | 6 | 1081 | 118.6 | 4.96 |
| Q8WXI9 | Transcriptional repressor p66-beta OS=Homo sapiens GN=GATAD2B PE=1 SV=1 - [P66B_HUMAN] | 5 | 3 | 6 | 593 | 65.2 | 9.7 |
| Q7L1Q6 | Basic leucine zipper and W2 domain-containing protein 1 OS=Homo sapiens GN=BZW1 PE=1 SV=1 - [BZW1_HUMAN] | 5 | 3 | 6 | 419 | 48 | 5.92 |
| Q16630 | Cleavage and polyadenylation specificity factor subunit 6 OS=Homo sapiens GN=CPSF6 PE=1 SV=2 - [CPSF6_HUMAN] | 5 | 3 | 6 | 551 | 59.2 | 7.15 |
| P18621 | 60S ribosomal protein L17 OS=Homo sapiens GN=RPL17 PE=1 SV=3 - [RL17_HUMAN] | 5 | 3 | 6 | 184 | 21.4 | 10.17 |
| P32322 | Pyrroline-5-carboxylate reductase 1, mitochondrial OS=Homo sapiens GN=PYCR1 PE=1 SV=2 - [P5CR1_HUMAN] | 5 | 3 | 6 | 319 | 33.3 | 7.61 |
| P19105 | Myosin regulatory light chain 12A OS=Homo sapiens GN=MYL12A PE=1 SV=2 - [ML12A_HUMAN] | 5 | 3 | 6 | 171 | 19.8 | 4.81 |
| Q15075 | Early endosome antigen 1 OS=Homo sapiens GN=EEA1 PE=1 SV=2 - [EEA1_HUMAN] | 5 | 2 | 4 | 1411 | 162.4 | 5.68 |
| Q14160 | Protein scribble homolog OS=Homo sapiens GN=SCRIB PE=1 SV=4 - [SCRIB_HUMAN] | 5 | 2 | 4 | 1630 | 174.8 | 5.07 |
| P49790 | Nuclear pore complex protein Nup153 OS=Homo sapiens GN=NUP153 PE=1 SV=2 - [NU153_HUMAN] | 5 | 2 | 4 | 1475 | 153.8 | 8.73 |
| O15371 | Eukaryotic translation initiation factor 3 subunit D OS=Homo sapiens GN=EIF3D PE=1 SV=1 - [EIF3D_HUMAN] | 5 | 2 | 4 | 548 | 63.9 | 6.05 |
| P51858 | Hepatoma-derived growth factor OS=Homo sapiens GN=HDGF PE=1 SV=1 - [HDGF_HUMAN] | 5 | 2 | 4 | 240 | 26.8 | 4.73 |
| P30626 | Sorcin OS=Homo sapiens GN=SRI PE=1 SV=1 - [SORCN_HUMAN] | 5 | 2 | 4 | 198 | 21.7 | 5.59 |
| Q13151 | Heterogeneous nuclear ribonucleoprotein A0 OS=Homo sapiens GN=HNRNPA0 PE=1 SV=1 - [ROA0_HUMAN] | 5 | 5 | 9 | 305 | 30.8 | 9.29 |
| P46783 | 40S ribosomal protein S10 OS=Homo sapiens GN=RPS10 PE=1 SV=1 - [RS10_HUMAN] | 5 | 8 | 14 | 165 | 18.9 | 10.15 |
| Q9GZS3 | WD repeat-containing protein 61 OS=Homo sapiens GN=WDR61 PE=1 SV=1 - [WDR61_HUMAN] | 5 | 4 | 7 | 305 | 33.6 | 5.47 |
| O75369 | Filamin-B OS=Homo sapiens GN=FLNB PE=1 SV=2 - [FLNB_HUMAN] | 5 | 6 | 10 | 2602 | 278 | 5.73 |
| P51784 | Ubiquitin carboxyl-terminal hydrolase 11 OS=Homo sapiens GN=USP11 PE=1 SV=3 - [UBP11_HUMAN] | 5 | 3 | 5 | 963 | 109.7 | 5.45 |
| Q92542 | Nicastrin OS=Homo sapiens GN=NCSTN PE=1 SV=2 - [NICA_HUMAN] | 5 | 3 | 5 | 709 | 78.4 | 5.99 |
| O75152 | Zinc finger CCCH domain-containing protein 11A OS=Homo sapiens GN=ZC3H11A PE=1 SV=3 - [ZC11A_HUMAN] | 5 | 3 | 5 | 810 | 89.1 | 8.37 |
| Q6NUK1 | Calcium-binding mitochondrial carrier protein SCaMC-1 OS=Homo sapiens GN=SLC25A24 PE=1 SV=2 - [SCMC1_HUMAN] | 5 | 3 | 5 | 477 | 53.3 | 6.33 |
| P18754 | Regulator of chromosome condensation OS=Homo sapiens GN=RCC1 PE=1 SV=1 - [RCC1_HUMAN] | 5 | 3 | 5 | 421 | 44.9 | 7.52 |
| O94888 | UBX domain-containing protein 7 OS=Homo sapiens GN=UBXN7 PE=1 SV=2 - [UBXN7_HUMAN] | 5 | 3 | 5 | 489 | 54.8 | 5.16 |
| Q92820 | Gamma-glutamyl hydrolase OS=Homo sapiens GN=GGH PE=1 SV=2 - [GGH_HUMAN] | 5 | 3 | 5 | 318 | 35.9 | 7.11 |
| Q7Z434 | Mitochondrial antiviral-signaling protein OS=Homo sapiens GN=MAVS PE=1 SV=2 - [MAVS_HUMAN] | 5 | 3 | 5 | 540 | 56.5 | 5.52 |
| Q9NRN7 | L-aminoadipate-semialdehyde dehydrogenase-phosphopantetheinyl transferase OS=Homo sapiens GN=AASDHPPT PE=1 SV=2 - [ADPPT_HUMAN] | 5 | 3 | 5 | 309 | 35.8 | 6.8 |
| Q15006 | Tetratricopeptide repeat protein 35 OS=Homo sapiens GN=TTC35 PE=1 SV=1 - [TTC35_HUMAN] | 5 | 3 | 5 | 297 | 34.8 | 6.57 |
| O60884 | DnaJ homolog subfamily A member 2 OS=Homo sapiens GN=DNAJA2 PE=1 SV=1 - [DNJA2_HUMAN] | 5 | 3 | 5 | 412 | 45.7 | 6.48 |
| P19623 | Spermidine synthase OS=Homo sapiens GN=SRM PE=1 SV=1 - [SPEE_HUMAN] | 5 | 3 | 5 | 302 | 33.8 | 5.49 |
| P18124 | 60S ribosomal protein L7 OS=Homo sapiens GN=RPL7 PE=1 SV=1 - [RL7_HUMAN] | 5 | 3 | 5 | 248 | 29.2 | 10.65 |
| P25325 | 3-mercaptopyruvate sulfurtransferase OS=Homo sapiens GN=MPST PE=1 SV=3 - [THTM_HUMAN] | 5 | 3 | 5 | 297 | 33.2 | 6.6 |
| P83731 | 60S ribosomal protein L24 OS=Homo sapiens GN=RPL24 PE=1 SV=1 - [RL24_HUMAN] | 5 | 3 | 5 | 157 | 17.8 | 11.25 |
| P00441 | Superoxide dismutase [Cu-Zn] OS=Homo sapiens GN=SOD1 PE=1 SV=2 - [SODC_HUMAN] | 5 | 7 | 11 | 154 | 15.9 | 6.13 |
| Q13185 | Chromobox protein homolog 3 OS=Homo sapiens GN=CBX3 PE=1 SV=4 - [CBX3_HUMAN] | 5 | 6 | 9 | 183 | 20.8 | 5.33 |
| Q29RF7 | Sister chromatid cohesion protein PDS5 homolog A OS=Homo sapiens GN=PDS5A PE=1 SV=1 - [PDS5A_HUMAN] | 5 | 4 | 6 | 1337 | 150.7 | 7.91 |
| P54687 | Branched-chain-amino-acid aminotransferase, cytosolic OS=Homo sapiens GN=BCAT1 PE=1 SV=3 - [BCAT1_HUMAN] | 5 | 4 | 6 | 386 | 42.9 | 5.3 |
| P62495 | Eukaryotic peptide chain release factor subunit 1 OS=Homo sapiens GN=ETF1 PE=1 SV=3 - [ERF1_HUMAN] | 5 | 4 | 6 | 437 | 49 | 5.71 |
| P14550 | Alcohol dehydrogenase [NADP+] OS=Homo sapiens GN=AKR1A1 PE=1 SV=3 - [AK1A1_HUMAN] | 5 | 4 | 6 | 325 | 36.5 | 6.79 |
| Q15102 | Platelet-activating factor acetylhydrolase IB subunit gamma OS=Homo sapiens GN=PAFAH1B3 PE=1 SV=1 - [PA1B3_HUMAN] | 5 | 4 | 6 | 231 | 25.7 | 6.84 |
| Q16629 | Splicing factor, arginine/serine-rich 7 OS=Homo sapiens GN=SFRS7 PE=1 SV=1 - [SFRS7_HUMAN] | 5 | 4 | 6 | 238 | 27.4 | 11.82 |
| O75937 | DnaJ homolog subfamily C member 8 OS=Homo sapiens GN=DNAJC8 PE=1 SV=2 - [DNJC8_HUMAN] | 5 | 4 | 6 | 253 | 29.8 | 9.06 |
| P32969 | 60S ribosomal protein L9 OS=Homo sapiens GN=RPL9 PE=1 SV=1 - [RL9_HUMAN] | 5 | 4 | 6 | 192 | 21.8 | 9.95 |
| P07339 | Cathepsin D OS=Homo sapiens GN=CTSD PE=1 SV=1 - [CATD_HUMAN] | 5 | 2 | 3 | 412 | 44.5 | 6.54 |
| O75400 | Pre-mRNA-processing factor 40 homolog A OS=Homo sapiens GN=PRPF40A PE=1 SV=2 - [PR40A_HUMAN] | 5 | 5 | 7 | 957 | 108.7 | 7.56 |
| O60749 | Sorting nexin-2 OS=Homo sapiens GN=SNX2 PE=1 SV=2 - [SNX2_HUMAN] | 5 | 5 | 7 | 519 | 58.4 | 5.12 |
| O00303 | Eukaryotic translation initiation factor 3 subunit F OS=Homo sapiens GN=EIF3F PE=1 SV=1 - [EIF3F_HUMAN] | 5 | 5 | 7 | 357 | 37.5 | 5.45 |
| P26368 | Splicing factor U2AF 65 kDa subunit OS=Homo sapiens GN=U2AF2 PE=1 SV=4 - [U2AF2_HUMAN] | 5 | 5 | 7 | 475 | 53.5 | 9.09 |
| P61163 | Alpha-centractin OS=Homo sapiens GN=ACTR1A PE=1 SV=1 - [ACTZ_HUMAN] | 5 | 5 | 7 | 376 | 42.6 | 6.64 |
| O00629 | Importin subunit alpha-4 OS=Homo sapiens GN=KPNA4 PE=1 SV=1 - [IMA4_HUMAN] | 5 | 6 | 8 | 521 | 57.9 | 4.96 |
| P05556 | Integrin beta-1 OS=Homo sapiens GN=ITGB1 PE=1 SV=2 - [ITB1_HUMAN] | 5 | 3 | 4 | 798 | 88.4 | 5.39 |
| Q15029 | 116 kDa U5 small nuclear ribonucleoprotein component OS=Homo sapiens GN=EFTUD2 PE=1 SV=1 - [U5S1_HUMAN] | 5 | 3 | 4 | 972 | 109.4 | 5 |
| O95831 | Apoptosis-inducing factor 1, mitochondrial OS=Homo sapiens GN=AIFM1 PE=1 SV=1 - [AIFM1_HUMAN] | 5 | 3 | 4 | 613 | 66.9 | 8.95 |
| Q9BXJ9 | NMDA receptor-regulated protein 1 OS=Homo sapiens GN=NARG1 PE=1 SV=1 - [NARG1_HUMAN] | 5 | 3 | 4 | 866 | 101.2 | 7.42 |
| Q9UMS4 | Pre-mRNA-processing factor 19 OS=Homo sapiens GN=PRPF19 PE=1 SV=1 - [PRP19_HUMAN] | 5 | 3 | 4 | 504 | 55.1 | 6.61 |
| Q08380 | Galectin-3-binding protein OS=Homo sapiens GN=LGALS3BP PE=1 SV=1 - [LG3BP_HUMAN] | 5 | 3 | 4 | 585 | 65.3 | 5.27 |
| O43237 | Cytoplasmic dynein 1 light intermediate chain 2 OS=Homo sapiens GN=DYNC1LI2 PE=1 SV=1 - [DC1L2_HUMAN] | 5 | 3 | 4 | 492 | 54.1 | 6.38 |
| Q9UNH7 | Sorting nexin-6 OS=Homo sapiens GN=SNX6 PE=1 SV=1 - [SNX6_HUMAN] | 5 | 3 | 4 | 406 | 46.6 | 6.16 |
| Q14696 | LDLR chaperone MESD OS=Homo sapiens GN=MESDC2 PE=1 SV=2 - [MESD_HUMAN] | 5 | 3 | 4 | 234 | 26.1 | 7.78 |
| P13995 | Bifunctional methylenetetrahydrofolate dehydrogenase/cyclohydrolase, mitochondrial OS=Homo sapiens GN=MTHFD2 PE=1 SV=2 - [MTDC_HUMAN] | 5 | 3 | 4 | 350 | 37.9 | 8.73 |
| Q86Y82 | Syntaxin-12 OS=Homo sapiens GN=STX12 PE=1 SV=1 - [STX12_HUMAN] | 5 | 3 | 4 | 276 | 31.6 | 5.59 |
| P22061 | Protein-L-isoaspartate(D-aspartate) O-methyltransferase OS=Homo sapiens GN=PCMT1 PE=1 SV=4 - [PIMT_HUMAN] | 5 | 3 | 4 | 227 | 24.6 | 7.21 |
| Q86U42 | Polyadenylate-binding protein 2 OS=Homo sapiens GN=PABPN1 PE=1 SV=3 - [PABP2_HUMAN] | 5 | 3 | 4 | 306 | 32.7 | 5.06 |
| Q07021 | Complement component 1 Q subcomponent-binding protein, mitochondrial OS=Homo sapiens GN=C1QBP PE=1 SV=1 - [C1QBP_HUMAN] | 5 | 7 | 9 | 282 | 31.3 | 4.84 |
| Q14103 | Heterogeneous nuclear ribonucleoprotein D0 OS=Homo sapiens GN=HNRNPD PE=1 SV=1 - [HNRPD_HUMAN] | 5 | 8 | 10 | 355 | 38.4 | 7.81 |
| Q8IZL8 | Proline-, glutamic acid- and leucine-rich protein 1 OS=Homo sapiens GN=PELP1 PE=1 SV=2 - [PELP1_HUMAN] | 5 | 4 | 5 | 1130 | 119.6 | 4.34 |
| P23588 | Eukaryotic translation initiation factor 4B OS=Homo sapiens GN=EIF4B PE=1 SV=2 - [IF4B_HUMAN] | 5 | 4 | 5 | 611 | 69.1 | 5.73 |
| P39748 | Flap endonuclease 1 OS=Homo sapiens GN=FEN1 PE=1 SV=1 - [FEN1_HUMAN] | 5 | 4 | 5 | 380 | 42.6 | 8.62 |
| Q13564 | NEDD8-activating enzyme E1 regulatory subunit OS=Homo sapiens GN=NAE1 PE=1 SV=1 - [ULA1_HUMAN] | 5 | 4 | 5 | 534 | 60.2 | 5.4 |
| P50579 | Methionine aminopeptidase 2 OS=Homo sapiens GN=METAP2 PE=1 SV=1 - [AMPM2_HUMAN] | 5 | 4 | 5 | 478 | 52.9 | 5.82 |
| Q9H9B4 | Sideroflexin-1 OS=Homo sapiens GN=SFXN1 PE=1 SV=4 - [SFXN1_HUMAN] | 5 | 4 | 5 | 322 | 35.6 | 9.07 |
| Q9BQA1 | Methylosome protein 50 OS=Homo sapiens GN=WDR77 PE=1 SV=1 - [MEP50_HUMAN] | 5 | 4 | 5 | 342 | 36.7 | 5.17 |
| Q3ZCQ8 | Mitochondrial import inner membrane translocase subunit TIM50 OS=Homo sapiens GN=TIMM50 PE=1 SV=2 - [TIM50_HUMAN] | 5 | 4 | 5 | 353 | 39.6 | 8.37 |
| Q9NZL4 | Hsp70-binding protein 1 OS=Homo sapiens GN=HSPBP1 PE=1 SV=1 - [HPBP1_HUMAN] | 5 | 4 | 5 | 362 | 39.4 | 5.21 |
| P25789 | Proteasome subunit alpha type-4 OS=Homo sapiens GN=PSMA4 PE=1 SV=1 - [PSA4_HUMAN] | 5 | 4 | 5 | 261 | 29.5 | 7.72 |
| P61081 | NEDD8-conjugating enzyme Ubc12 OS=Homo sapiens GN=UBE2M PE=1 SV=1 - [UBC12_HUMAN] | 5 | 4 | 5 | 183 | 20.9 | 7.69 |
| O60684 | Importin subunit alpha-7 OS=Homo sapiens GN=KPNA6 PE=1 SV=1 - [IMA7_HUMAN] | 5 | 5 | 6 | 536 | 60 | 4.98 |
| Q07666 | KH domain-containing, RNA-binding, signal transduction-associated protein 1 OS=Homo sapiens GN=KHDRBS1 PE=1 SV=1 - [KHDR1_HUMAN] | 5 | 5 | 6 | 443 | 48.2 | 8.66 |
| Q13765 | Nascent polypeptide-associated complex subunit alpha OS=Homo sapiens GN=NACA PE=1 SV=1 - [NACA_HUMAN] | 5 | 6 | 7 | 215 | 23.4 | 4.56 |
| P51148 | Ras-related protein Rab-5C OS=Homo sapiens GN=RAB5C PE=1 SV=2 - [RAB5C_HUMAN] | 5 | 6 | 7 | 216 | 23.5 | 8.41 |
| P08708 | 40S ribosomal protein S17 OS=Homo sapiens GN=RPS17 PE=1 SV=2 - [RS17_HUMAN] | 5 | 7 | 8 | 135 | 15.5 | 9.85 |
| O75822 | Eukaryotic translation initiation factor 3 subunit J OS=Homo sapiens GN=EIF3J PE=1 SV=2 - [EIF3J_HUMAN] | 5 | 9 | 10 | 258 | 29 | 4.83 |
| Q13509 | Tubulin beta-3 chain OS=Homo sapiens GN=TUBB3 PE=1 SV=2 - [TBB3_HUMAN] | 5 | 42 | 46 | 450 | 50.4 | 4.93 |
| P54727 | UV excision repair protein RAD23 homolog B OS=Homo sapiens GN=RAD23B PE=1 SV=1 - [RD23B_HUMAN] | 5 | 9 | 9 | 409 | 43.1 | 4.84 |
| Q14194 | Dihydropyrimidinase-related protein 1 OS=Homo sapiens GN=CRMP1 PE=1 SV=1 - [DPYL1_HUMAN] | 5 | 6 | 6 | 572 | 62.1 | 7.03 |
| P63092 | Guanine nucleotide-binding protein G(s) subunit alpha isoforms short OS=Homo sapiens GN=GNAS PE=1 SV=1 - [GNAS2_HUMAN] | 5 | 6 | 6 | 394 | 45.6 | 5.82 |
| P62277 | 40S ribosomal protein S13 OS=Homo sapiens GN=RPS13 PE=1 SV=2 - [RS13_HUMAN] | 5 | 6 | 6 | 151 | 17.2 | 10.54 |
| P52565 | Rho GDP-dissociation inhibitor 1 OS=Homo sapiens GN=ARHGDIA PE=1 SV=3 - [GDIR1_HUMAN] | 5 | 6 | 6 | 204 | 23.2 | 5.11 |
| Q9UBC2 | Epidermal growth factor receptor substrate 15-like 1 OS=Homo sapiens GN=EPS15L1 PE=1 SV=1 - [EP15R_HUMAN] | 5 | 5 | 5 | 864 | 94.2 | 5.11 |
| P09622 | Dihydrolipoyl dehydrogenase, mitochondrial OS=Homo sapiens GN=DLD PE=1 SV=2 - [DLDH_HUMAN] | 5 | 5 | 5 | 509 | 54.1 | 7.85 |
| Q8TBC4 | NEDD8-activating enzyme E1 catalytic subunit OS=Homo sapiens GN=UBA3 PE=1 SV=2 - [UBA3_HUMAN] | 5 | 5 | 5 | 463 | 51.8 | 5.45 |
| Q15274 | Nicotinate-nucleotide pyrophosphorylase [carboxylating] OS=Homo sapiens GN=QPRT PE=1 SV=3 - [NADC_HUMAN] | 5 | 5 | 5 | 297 | 30.8 | 6.21 |
| P52888 | Thimet oligopeptidase OS=Homo sapiens GN=THOP1 PE=1 SV=2 - [THOP1_HUMAN] | 5 | 4 | 4 | 689 | 78.8 | 6.05 |
| Q86XP3 | ATP-dependent RNA helicase DDX42 OS=Homo sapiens GN=DDX42 PE=1 SV=1 - [DDX42_HUMAN] | 5 | 4 | 4 | 938 | 102.9 | 7.02 |
| P31153 | S-adenosylmethionine synthetase isoform type-2 OS=Homo sapiens GN=MAT2A PE=1 SV=1 - [METK2_HUMAN] | 5 | 4 | 4 | 395 | 43.6 | 6.48 |
| P23381 | Tryptophanyl-tRNA synthetase, cytoplasmic OS=Homo sapiens GN=WARS PE=1 SV=2 - [SYWC_HUMAN] | 5 | 4 | 4 | 471 | 53.1 | 6.23 |
| Q9UH62 | Armadillo repeat-containing X-linked protein 3 OS=Homo sapiens GN=ARMCX3 PE=1 SV=1 - [ARMX3_HUMAN] | 5 | 4 | 4 | 379 | 42.5 | 8.37 |
| P62993 | Growth factor receptor-bound protein 2 OS=Homo sapiens GN=GRB2 PE=1 SV=1 - [GRB2_HUMAN] | 5 | 4 | 4 | 217 | 25.2 | 6.32 |
| Q9BYG3 | MKI67 FHA domain-interacting nucleolar phosphoprotein OS=Homo sapiens GN=NIFK PE=1 SV=1 - [MK67I_HUMAN] | 5 | 4 | 4 | 293 | 34.2 | 9.88 |
| P82979 | SAP domain-containing ribonucleoprotein OS=Homo sapiens GN=SARNP PE=1 SV=3 - [SARNP_HUMAN] | 5 | 4 | 4 | 210 | 23.7 | 6.42 |
| O94979 | Protein transport protein Sec31A OS=Homo sapiens GN=SEC31A PE=1 SV=3 - [SC31A_HUMAN] | 5 | 3 | 3 | 1220 | 132.9 | 6.89 |
| P19525 | Interferon-induced, double-stranded RNA-activated protein kinase OS=Homo sapiens GN=EIF2AK2 PE=1 SV=2 - [E2AK2_HUMAN] | 5 | 3 | 3 | 551 | 62.1 | 8.4 |
| Q9Y4P1 | Cysteine protease ATG4B OS=Homo sapiens GN=ATG4B PE=1 SV=2 - [ATG4B_HUMAN] | 5 | 3 | 3 | 393 | 44.3 | 5.07 |
| P31946 | 14-3-3 protein beta/alpha OS=Homo sapiens GN=YWHAB PE=1 SV=3 - [1433B_HUMAN] | 5 | 19 | 17 | 246 | 28.1 | 4.83 |
| P62995 | Transformer-2 protein homolog beta OS=Homo sapiens GN=TRA2B PE=1 SV=1 - [TRA2B_HUMAN] | 5 | 6 | 5 | 288 | 33.6 | 11.25 |
| P11177 | Pyruvate dehydrogenase E1 component subunit beta, mitochondrial OS=Homo sapiens GN=PDHB PE=1 SV=3 - [ODPB_HUMAN] | 5 | 5 | 4 | 359 | 39.2 | 6.65 |
| O43765 | Small glutamine-rich tetratricopeptide repeat-containing protein alpha OS=Homo sapiens GN=SGTA PE=1 SV=1 - [SGTA_HUMAN] | 5 | 5 | 4 | 313 | 34 | 4.87 |
| Q00577 | Transcriptional activator protein Pur-alpha OS=Homo sapiens GN=PURA PE=1 SV=2 - [PURA_HUMAN] | 5 | 5 | 4 | 322 | 34.9 | 6.44 |
| P46087 | Putative ribosomal RNA methyltransferase NOP2 OS=Homo sapiens GN=NOP2 PE=1 SV=2 - [NOP2_HUMAN] | 5 | 4 | 3 | 812 | 89.2 | 9.23 |
| P05198 | Eukaryotic translation initiation factor 2 subunit 1 OS=Homo sapiens GN=EIF2S1 PE=1 SV=3 - [IF2A_HUMAN] | 5 | 4 | 3 | 315 | 36.1 | 5.08 |
| Q16718 | NADH dehydrogenase [ubiquinone] 1 alpha subcomplex subunit 5 OS=Homo sapiens GN=NDUFA5 PE=1 SV=3 - [NDUA5_HUMAN] | 5 | 4 | 3 | 116 | 13.5 | 5.99 |
| O75347 | Tubulin-specific chaperone A OS=Homo sapiens GN=TBCA PE=1 SV=3 - [TBCA_HUMAN] | 5 | 4 | 3 | 108 | 12.8 | 5.29 |
| P62873 | Guanine nucleotide-binding protein G(I)/G(S)/G(T) subunit beta-1 OS=Homo sapiens GN=GNB1 PE=1 SV=3 - [GBB1_HUMAN] | 5 | 6 | 4 | 340 | 37.4 | 6 |
| P02461 | Collagen alpha-1(III) chain OS=Homo sapiens GN=COL3A1 PE=1 SV=4 - [CO3A1_HUMAN] | 5 | 4 | 2 | 1466 | 138.5 | 6.61 |
| Q9Y2X3 | Nucleolar protein 58 OS=Homo sapiens GN=NOP58 PE=1 SV=1 - [NOP58_HUMAN] | 5 | 4 | 2 | 529 | 59.5 | 8.92 |
| P37837 | Transaldolase OS=Homo sapiens GN=TALDO1 PE=1 SV=2 - [TALDO_HUMAN] | 5 | 4 | 1 | 337 | 37.5 | 6.81 |
| P02792 | Ferritin light chain OS=Homo sapiens GN=FTL PE=1 SV=2 - [FRIL_HUMAN] | 4 | 0 | 5 | 175 | 20 | 5.78 |
| P61923 | Coatomer subunit zeta-1 OS=Homo sapiens GN=COPZ1 PE=1 SV=1 - [COPZ1_HUMAN] | 4 | 0 | 4 | 177 | 20.2 | 4.81 |
| P20618 | Proteasome subunit beta type-1 OS=Homo sapiens GN=PSMB1 PE=1 SV=2 - [PSB1_HUMAN] | 4 | 0 | 4 | 241 | 26.5 | 8.13 |
| Q9Y3D9 | 28S ribosomal protein S23, mitochondrial OS=Homo sapiens GN=MRPS23 PE=1 SV=2 - [RT23_HUMAN] | 4 | 0 | 4 | 190 | 21.8 | 8.9 |
| P30519 | Heme oxygenase 2 OS=Homo sapiens GN=HMOX2 PE=1 SV=2 - [HMOX2_HUMAN] | 4 | 0 | 4 | 316 | 36 | 5.41 |
| P00374 | Dihydrofolate reductase OS=Homo sapiens GN=DHFR PE=1 SV=2 - [DYR_HUMAN] | 4 | 0 | 4 | 187 | 21.4 | 7.42 |
| P15170 | Eukaryotic peptide chain release factor GTP-binding subunit ERF3A OS=Homo sapiens GN=GSPT1 PE=1 SV=1 - [ERF3A_HUMAN] | 4 | 0 | 4 | 499 | 55.7 | 5.62 |
| Q8IVD9 | NudC domain-containing protein 3 OS=Homo sapiens GN=NUDCD3 PE=1 SV=3 - [NUDC3_HUMAN] | 4 | 0 | 4 | 361 | 40.8 | 5.25 |
| Q15904 | V-type proton ATPase subunit S1 OS=Homo sapiens GN=ATP6AP1 PE=1 SV=2 - [VAS1_HUMAN] | 4 | 0 | 4 | 470 | 52 | 6.14 |
| Q9Y3Z3 | SAM domain and HD domain-containing protein 1 OS=Homo sapiens GN=SAMHD1 PE=1 SV=2 - [SAMH1_HUMAN] | 4 | 0 | 4 | 626 | 72.2 | 7.14 |
| Q9Y6G9 | Cytoplasmic dynein 1 light intermediate chain 1 OS=Homo sapiens GN=DYNC1LI1 PE=1 SV=3 - [DC1L1_HUMAN] | 4 | 0 | 4 | 523 | 56.5 | 6.42 |
| Q8IVL6 | Prolyl 3-hydroxylase 3 OS=Homo sapiens GN=LEPREL2 PE=2 SV=1 - [P3H3_HUMAN] | 4 | 0 | 4 | 736 | 81.8 | 6.32 |
| Q68E01 | Integrator complex subunit 3 OS=Homo sapiens GN=INTS3 PE=1 SV=1 - [INT3_HUMAN] | 4 | 0 | 4 | 1043 | 118 | 5.8 |
| Q9H6T3 | RNA polymerase II-associated protein 3 OS=Homo sapiens GN=RPAP3 PE=1 SV=2 - [RPAP3_HUMAN] | 4 | 0 | 4 | 665 | 75.7 | 6.84 |
| Q9H307 | Pinin OS=Homo sapiens GN=PNN PE=1 SV=4 - [PININ_HUMAN] | 4 | 0 | 4 | 717 | 81.6 | 7.14 |
| Q96TA2 | ATP-dependent metalloprotease YME1L1 OS=Homo sapiens GN=YME1L1 PE=1 SV=2 - [YMEL1_HUMAN] | 4 | 1 | 6 | 773 | 86.4 | 8.76 |
| P49755 | Transmembrane emp24 domain-containing protein 10 OS=Homo sapiens GN=TMED10 PE=1 SV=2 - [TMEDA_HUMAN] | 4 | 1 | 6 | 219 | 25 | 7.44 |
| Q14978 | Nucleolar and coiled-body phosphoprotein 1 OS=Homo sapiens GN=NOLC1 PE=1 SV=2 - [NOLC1_HUMAN] | 4 | 1 | 5 | 699 | 73.6 | 9.47 |
| O75475 | PC4 and SFRS1-interacting protein OS=Homo sapiens GN=PSIP1 PE=1 SV=1 - [PSIP1_HUMAN] | 4 | 1 | 5 | 530 | 60.1 | 9.13 |
| Q9Y295 | Developmentally-regulated GTP-binding protein 1 OS=Homo sapiens GN=DRG1 PE=1 SV=1 - [DRG1_HUMAN] | 4 | 1 | 5 | 367 | 40.5 | 8.9 |
| Q15435 | Protein phosphatase 1 regulatory subunit 7 OS=Homo sapiens GN=PPP1R7 PE=1 SV=1 - [PP1R7_HUMAN] | 4 | 1 | 5 | 360 | 41.5 | 4.91 |
| Q99986 | Serine/threonine-protein kinase VRK1 OS=Homo sapiens GN=VRK1 PE=1 SV=1 - [VRK1_HUMAN] | 4 | 1 | 5 | 396 | 45.4 | 8.91 |
| Q9BVK6 | Transmembrane emp24 domain-containing protein 9 OS=Homo sapiens GN=TMED9 PE=1 SV=2 - [TMED9_HUMAN] | 4 | 1 | 5 | 235 | 27.3 | 8.02 |
| P45973 | Chromobox protein homolog 5 OS=Homo sapiens GN=CBX5 PE=1 SV=1 - [CBX5_HUMAN] | 4 | 1 | 5 | 191 | 22.2 | 5.86 |
| P16455 | Methylated-DNA--protein-cysteine methyltransferase OS=Homo sapiens GN=MGMT PE=1 SV=1 - [MGMT_HUMAN] | 4 | 1 | 5 | 207 | 21.6 | 8.1 |
| Q15813 | Tubulin-specific chaperone E OS=Homo sapiens GN=TBCE PE=1 SV=1 - [TBCE_HUMAN] | 4 | 1 | 4 | 527 | 59.3 | 6.76 |
| Q8WYA6 | Beta-catenin-like protein 1 OS=Homo sapiens GN=CTNNBL1 PE=1 SV=1 - [CTBL1_HUMAN] | 4 | 1 | 4 | 563 | 65.1 | 5.05 |
| Q96JB5 | CDK5 regulatory subunit-associated protein 3 OS=Homo sapiens GN=CDK5RAP3 PE=1 SV=2 - [CK5P3_HUMAN] | 4 | 1 | 4 | 506 | 56.9 | 4.75 |
| O00567 | Nucleolar protein 56 OS=Homo sapiens GN=NOP56 PE=1 SV=4 - [NOP56_HUMAN] | 4 | 1 | 4 | 594 | 66 | 9.19 |
| Q01844 | RNA-binding protein EWS OS=Homo sapiens GN=EWSR1 PE=1 SV=1 - [EWS_HUMAN] | 4 | 1 | 4 | 656 | 68.4 | 9.33 |
| Q14677 | Clathrin interactor 1 OS=Homo sapiens GN=CLINT1 PE=1 SV=1 - [EPN4_HUMAN] | 4 | 1 | 4 | 625 | 68.2 | 6.42 |
| Q16836 | Hydroxyacyl-coenzyme A dehydrogenase, mitochondrial OS=Homo sapiens GN=HADH PE=1 SV=3 - [HCDH_HUMAN] | 4 | 1 | 4 | 314 | 34.3 | 8.85 |
| O95218 | Zinc finger Ran-binding domain-containing protein 2 OS=Homo sapiens GN=ZRANB2 PE=1 SV=2 - [ZRAB2_HUMAN] | 4 | 1 | 4 | 330 | 37.4 | 10.01 |
| Q96GM5 | SWI/SNF-related matrix-associated actin-dependent regulator of chromatin subfamily D member 1 OS=Homo sapiens GN=SMARCD1 PE=1 SV=2 - [SMRD1_HUMAN] | 4 | 1 | 4 | 515 | 58.2 | 9.25 |
| Q7Z5L9 | Interferon regulatory factor 2-binding protein 2 OS=Homo sapiens GN=IRF2BP2 PE=1 SV=2 - [I2BP2_HUMAN] | 4 | 1 | 4 | 587 | 61 | 8.69 |
| P40222 | Alpha-taxilin OS=Homo sapiens GN=TXLNA PE=1 SV=3 - [TXLNA_HUMAN] | 4 | 1 | 4 | 546 | 61.9 | 6.52 |
| P23258 | Tubulin gamma-1 chain OS=Homo sapiens GN=TUBG1 PE=1 SV=2 - [TBG1_HUMAN] | 4 | 1 | 4 | 451 | 51.1 | 6.14 |
| Q96DH6 | RNA-binding protein Musashi homolog 2 OS=Homo sapiens GN=MSI2 PE=1 SV=1 - [MSI2H_HUMAN] | 4 | 1 | 4 | 328 | 35.2 | 8.48 |
| P54920 | Alpha-soluble NSF attachment protein OS=Homo sapiens GN=NAPA PE=1 SV=3 - [SNAA_HUMAN] | 4 | 1 | 4 | 295 | 33.2 | 5.36 |
| P28074 | Proteasome subunit beta type-5 OS=Homo sapiens GN=PSMB5 PE=1 SV=3 - [PSB5_HUMAN] | 4 | 1 | 4 | 263 | 28.5 | 6.92 |
| P53701 | Cytochrome c-type heme lyase OS=Homo sapiens GN=HCCS PE=1 SV=1 - [CCHL_HUMAN] | 4 | 1 | 4 | 268 | 30.6 | 6.68 |
| P55957 | BH3-interacting domain death agonist OS=Homo sapiens GN=BID PE=1 SV=1 - [BID_HUMAN] | 4 | 1 | 4 | 195 | 22 | 5.44 |
| Q07020 | 60S ribosomal protein L18 OS=Homo sapiens GN=RPL18 PE=1 SV=2 - [RL18_HUMAN] | 4 | 1 | 4 | 188 | 21.6 | 11.72 |
| Q3MHD2 | Protein LSM12 homolog OS=Homo sapiens GN=LSM12 PE=1 SV=2 - [LSM12_HUMAN] | 4 | 1 | 4 | 195 | 21.7 | 7.74 |
| Q13442 | 28 kDa heat- and acid-stable phosphoprotein OS=Homo sapiens GN=PDAP1 PE=1 SV=1 - [HAP28_HUMAN] | 4 | 1 | 4 | 181 | 20.6 | 8.87 |
| Q8IY81 | pre-rRNA processing protein FTSJ3 OS=Homo sapiens GN=FTSJ3 PE=1 SV=2 - [SPB1_HUMAN] | 4 | 2 | 6 | 847 | 96.5 | 8.4 |
| P30837 | Aldehyde dehydrogenase X, mitochondrial OS=Homo sapiens GN=ALDH1B1 PE=1 SV=3 - [AL1B1_HUMAN] | 4 | 2 | 6 | 517 | 57.2 | 6.8 |
| Q9Y4W6 | AFG3-like protein 2 OS=Homo sapiens GN=AFG3L2 PE=1 SV=2 - [AFG32_HUMAN] | 4 | 1 | 3 | 797 | 88.5 | 8.66 |
| P35221 | Catenin alpha-1 OS=Homo sapiens GN=CTNNA1 PE=1 SV=1 - [CTNA1_HUMAN] | 4 | 1 | 3 | 906 | 100 | 6.29 |
| Q6PJT7 | Zinc finger CCCH domain-containing protein 14 OS=Homo sapiens GN=ZC3H14 PE=1 SV=1 - [ZC3HE_HUMAN] | 4 | 1 | 3 | 736 | 82.8 | 7.31 |
| Q86VS8 | Protein Hook homolog 3 OS=Homo sapiens GN=HOOK3 PE=1 SV=2 - [HOOK3_HUMAN] | 4 | 1 | 3 | 718 | 83.1 | 5.17 |
| Q99816 | Tumor susceptibility gene 101 protein OS=Homo sapiens GN=TSG101 PE=1 SV=2 - [TS101_HUMAN] | 4 | 1 | 3 | 390 | 43.9 | 6.46 |
| Q16795 | NADH dehydrogenase [ubiquinone] 1 alpha subcomplex subunit 9, mitochondrial OS=Homo sapiens GN=NDUFA9 PE=1 SV=2 - [NDUA9_HUMAN] | 4 | 1 | 3 | 377 | 42.5 | 9.8 |
| O95400 | CD2 antigen cytoplasmic tail-binding protein 2 OS=Homo sapiens GN=CD2BP2 PE=1 SV=1 - [CD2B2_HUMAN] | 4 | 1 | 3 | 341 | 37.6 | 4.61 |
| Q15014 | Mortality factor 4-like protein 2 OS=Homo sapiens GN=MORF4L2 PE=1 SV=1 - [MO4L2_HUMAN] | 4 | 1 | 3 | 288 | 32.3 | 9.72 |
| Q9BRX5 | DNA replication complex GINS protein PSF3 OS=Homo sapiens GN=GINS3 PE=1 SV=1 - [PSF3_HUMAN] | 4 | 1 | 3 | 216 | 24.5 | 5.34 |
| P53990 | IST1 homolog OS=Homo sapiens GN=KIAA0174 PE=1 SV=1 - [IST1_HUMAN] | 4 | 2 | 5 | 364 | 39.7 | 5.35 |
| Q15050 | Ribosome biogenesis regulatory protein homolog OS=Homo sapiens GN=RRS1 PE=1 SV=2 - [RRS1_HUMAN] | 4 | 2 | 5 | 365 | 41.2 | 10.7 |
| Q9BTE6 | Alanyl-tRNA editing protein Aarsd1 OS=Homo sapiens GN=AARSD1 PE=1 SV=2 - [AASD1_HUMAN] | 4 | 2 | 5 | 412 | 45.5 | 6.42 |
| P07858 | Cathepsin B OS=Homo sapiens GN=CTSB PE=1 SV=3 - [CATB_HUMAN] | 4 | 2 | 5 | 339 | 37.8 | 6.3 |
| P46108 | Adapter molecule crk OS=Homo sapiens GN=CRK PE=1 SV=2 - [CRK_HUMAN] | 4 | 2 | 5 | 304 | 33.8 | 5.55 |
| Q99653 | Calcium-binding protein p22 OS=Homo sapiens GN=CHP PE=1 SV=3 - [CHP1_HUMAN] | 4 | 2 | 5 | 195 | 22.4 | 5.1 |
| Q15126 | Phosphomevalonate kinase OS=Homo sapiens GN=PMVK PE=1 SV=3 - [PMVK_HUMAN] | 4 | 2 | 5 | 192 | 22 | 5.73 |
| P62913 | 60S ribosomal protein L11 OS=Homo sapiens GN=RPL11 PE=1 SV=2 - [RL11_HUMAN] | 4 | 2 | 5 | 178 | 20.2 | 9.6 |
| Q9GZT3 | SRA stem-loop-interacting RNA-binding protein, mitochondrial OS=Homo sapiens GN=SLIRP PE=1 SV=1 - [SLIRP_HUMAN] | 4 | 2 | 5 | 109 | 12.3 | 10.24 |
| P63173 | 60S ribosomal protein L38 OS=Homo sapiens GN=RPL38 PE=1 SV=2 - [RL38_HUMAN] | 4 | 3 | 6 | 70 | 8.2 | 10.1 |
| P07942 | Laminin subunit beta-1 OS=Homo sapiens GN=LAMB1 PE=1 SV=2 - [LAMB1_HUMAN] | 4 | 2 | 4 | 1786 | 197.9 | 4.94 |
| Q8NBJ5 | Procollagen galactosyltransferase 1 OS=Homo sapiens GN=GLT25D1 PE=1 SV=1 - [GT251_HUMAN] | 4 | 2 | 4 | 622 | 71.6 | 7.31 |
| Q9NW13 | RNA-binding protein 28 OS=Homo sapiens GN=RBM28 PE=1 SV=3 - [RBM28_HUMAN] | 4 | 2 | 4 | 759 | 85.7 | 9.22 |
| Q9BWU0 | Kanadaptin OS=Homo sapiens GN=SLC4A1AP PE=1 SV=1 - [NADAP_HUMAN] | 4 | 2 | 4 | 796 | 88.8 | 5.19 |
| Q9UQ88 | Cyclin-dependent kinase 11A OS=Homo sapiens GN=CDK11A PE=1 SV=4 - [CD11A_HUMAN] | 4 | 2 | 4 | 783 | 91.3 | 5.36 |
| Q13895 | Bystin OS=Homo sapiens GN=BYSL PE=1 SV=3 - [BYST_HUMAN] | 4 | 2 | 4 | 437 | 49.6 | 8.12 |
| Q86TG7 | Retrotransposon-derived protein PEG10 OS=Homo sapiens GN=PEG10 PE=1 SV=2 - [PEG10_HUMAN] | 4 | 2 | 4 | 708 | 80.1 | 6.39 |
| Q8NCA5 | Protein FAM98A OS=Homo sapiens GN=FAM98A PE=1 SV=1 - [FA98A_HUMAN] | 4 | 2 | 4 | 519 | 55.4 | 9.03 |
| O96019 | Actin-like protein 6A OS=Homo sapiens GN=ACTL6A PE=1 SV=1 - [ACL6A_HUMAN] | 4 | 2 | 4 | 429 | 47.4 | 5.6 |
| Q9Y376 | Calcium-binding protein 39 OS=Homo sapiens GN=CAB39 PE=1 SV=1 - [CAB39_HUMAN] | 4 | 2 | 4 | 341 | 39.8 | 6.89 |
| P36543 | V-type proton ATPase subunit E 1 OS=Homo sapiens GN=ATP6V1E1 PE=1 SV=1 - [VATE1_HUMAN] | 4 | 2 | 4 | 226 | 26.1 | 8 |
| Q969X5 | Endoplasmic reticulum-Golgi intermediate compartment protein 1 OS=Homo sapiens GN=ERGIC1 PE=1 SV=1 - [ERGI1_HUMAN] | 4 | 2 | 4 | 290 | 32.6 | 7.06 |
| O43681 | ATPase ASNA1 OS=Homo sapiens GN=ASNA1 PE=1 SV=2 - [ASNA_HUMAN] | 4 | 2 | 4 | 348 | 38.8 | 4.91 |
| Q8ND56 | Protein LSM14 homolog A OS=Homo sapiens GN=LSM14A PE=1 SV=3 - [LS14A_HUMAN] | 4 | 2 | 4 | 463 | 50.5 | 9.52 |
| P13861 | cAMP-dependent protein kinase type II-alpha regulatory subunit OS=Homo sapiens GN=PRKAR2A PE=1 SV=2 - [KAP2_HUMAN] | 4 | 2 | 4 | 404 | 45.5 | 5.07 |
| P48637 | Glutathione synthetase OS=Homo sapiens GN=GSS PE=1 SV=1 - [GSHB_HUMAN] | 4 | 2 | 4 | 474 | 52.4 | 5.92 |
| Q9UQB8 | Brain-specific angiogenesis inhibitor 1-associated protein 2 OS=Homo sapiens GN=BAIAP2 PE=1 SV=1 - [BAIP2_HUMAN] | 4 | 2 | 4 | 552 | 60.8 | 8.9 |
| P62917 | 60S ribosomal protein L8 OS=Homo sapiens GN=RPL8 PE=1 SV=2 - [RL8_HUMAN] | 4 | 2 | 4 | 257 | 28 | 11.03 |
| P31937 | 3-hydroxyisobutyrate dehydrogenase, mitochondrial OS=Homo sapiens GN=HIBADH PE=1 SV=2 - [3HIDH_HUMAN] | 4 | 2 | 4 | 336 | 35.3 | 8.13 |
| P28072 | Proteasome subunit beta type-6 OS=Homo sapiens GN=PSMB6 PE=1 SV=4 - [PSB6_HUMAN] | 4 | 2 | 4 | 239 | 25.3 | 4.92 |
| Q9NR45 | Sialic acid synthase OS=Homo sapiens GN=NANS PE=1 SV=2 - [SIAS_HUMAN] | 4 | 2 | 4 | 359 | 40.3 | 6.74 |
| P23919 | Thymidylate kinase OS=Homo sapiens GN=DTYMK PE=1 SV=4 - [KTHY_HUMAN] | 4 | 2 | 4 | 212 | 23.8 | 8.27 |
| P40938 | Replication factor C subunit 3 OS=Homo sapiens GN=RFC3 PE=1 SV=2 - [RFC3_HUMAN] | 4 | 2 | 4 | 356 | 40.5 | 8.34 |
| Q9NPD3 | Exosome complex exonuclease RRP41 OS=Homo sapiens GN=EXOSC4 PE=1 SV=3 - [EXOS4_HUMAN] | 4 | 2 | 4 | 245 | 26.4 | 6.52 |
| O75494 | Splicing factor, arginine/serine-rich 13A OS=Homo sapiens GN=SFRS13A PE=1 SV=1 - [SF13A_HUMAN] | 4 | 2 | 4 | 262 | 31.3 | 11.27 |
| Q9H4A6 | Golgi phosphoprotein 3 OS=Homo sapiens GN=GOLPH3 PE=1 SV=1 - [GOLP3_HUMAN] | 4 | 2 | 4 | 298 | 33.8 | 6.44 |
| Q9NX63 | Coiled-coil-helix-coiled-coil-helix domain-containing protein 3, mitochondrial OS=Homo sapiens GN=CHCHD3 PE=1 SV=1 - [CHCH3_HUMAN] | 4 | 2 | 4 | 227 | 26.1 | 8.28 |
| Q96DG6 | Carboxymethylenebutenolidase homolog OS=Homo sapiens GN=CMBL PE=1 SV=1 - [CMBL_HUMAN] | 4 | 2 | 4 | 245 | 28 | 7.18 |
| O15173 | Membrane-associated progesterone receptor component 2 OS=Homo sapiens GN=PGRMC2 PE=1 SV=1 - [PGRC2_HUMAN] | 4 | 2 | 4 | 223 | 23.8 | 4.88 |
| Q15366 | Poly(rC)-binding protein 2 OS=Homo sapiens GN=PCBP2 PE=1 SV=1 - [PCBP2_HUMAN] | 4 | 6 | 11 | 365 | 38.6 | 6.79 |
| Q15637 | Splicing factor 1 OS=Homo sapiens GN=SF1 PE=1 SV=4 - [SF01_HUMAN] | 4 | 3 | 5 | 639 | 68.3 | 8.98 |
| Q15046 | Lysyl-tRNA synthetase OS=Homo sapiens GN=KARS PE=1 SV=3 - [SYK_HUMAN] | 4 | 3 | 5 | 597 | 68 | 6.35 |
| Q9BQ67 | Glutamate-rich WD repeat-containing protein 1 OS=Homo sapiens GN=GRWD1 PE=1 SV=1 - [GRWD1_HUMAN] | 4 | 3 | 5 | 446 | 49.4 | 4.92 |
| P50897 | Palmitoyl-protein thioesterase 1 OS=Homo sapiens GN=PPT1 PE=1 SV=1 - [PPT1_HUMAN] | 4 | 3 | 5 | 306 | 34.2 | 6.52 |
| P29083 | General transcription factor IIE subunit 1 OS=Homo sapiens GN=GTF2E1 PE=1 SV=2 - [T2EA_HUMAN] | 4 | 3 | 5 | 439 | 49.4 | 4.82 |
| P62249 | 40S ribosomal protein S16 OS=Homo sapiens GN=RPS16 PE=1 SV=2 - [RS16_HUMAN] | 4 | 3 | 5 | 146 | 16.4 | 10.21 |
| P30044 | Peroxiredoxin-5, mitochondrial OS=Homo sapiens GN=PRDX5 PE=1 SV=4 - [PRDX5_HUMAN] | 4 | 3 | 5 | 214 | 22.1 | 8.7 |
| P28161 | Glutathione S-transferase Mu 2 OS=Homo sapiens GN=GSTM2 PE=1 SV=2 - [GSTM2_HUMAN] | 4 | 3 | 5 | 218 | 25.7 | 6.37 |
| Q05519 | Splicing factor, arginine/serine-rich 11 OS=Homo sapiens GN=SFRS11 PE=1 SV=1 - [SFR11_HUMAN] | 4 | 4 | 6 | 484 | 53.5 | 10.52 |
| P49750 | YLP motif-containing protein 1 OS=Homo sapiens GN=YLPM1 PE=1 SV=3 - [YLPM1_HUMAN] | 4 | 2 | 3 | 1951 | 219.8 | 6.57 |
| P17480 | Nucleolar transcription factor 1 OS=Homo sapiens GN=UBTF PE=1 SV=1 - [UBF1_HUMAN] | 4 | 2 | 3 | 764 | 89.4 | 5.81 |
| P55884 | Eukaryotic translation initiation factor 3 subunit B OS=Homo sapiens GN=EIF3B PE=1 SV=3 - [EIF3B_HUMAN] | 4 | 2 | 3 | 814 | 92.4 | 5 |
| O60568 | Procollagen-lysine,2-oxoglutarate 5-dioxygenase 3 OS=Homo sapiens GN=PLOD3 PE=1 SV=1 - [PLOD3_HUMAN] | 4 | 2 | 3 | 738 | 84.7 | 6.05 |
| Q14203 | Dynactin subunit 1 OS=Homo sapiens GN=DCTN1 PE=1 SV=3 - [DCTN1_HUMAN] | 4 | 2 | 3 | 1278 | 141.6 | 5.81 |
| P49748 | Very long-chain specific acyl-CoA dehydrogenase, mitochondrial OS=Homo sapiens GN=ACADVL PE=1 SV=1 - [ACADV_HUMAN] | 4 | 2 | 3 | 655 | 70.3 | 8.75 |
| Q6P1J9 | Parafibromin OS=Homo sapiens GN=CDC73 PE=1 SV=1 - [CDC73_HUMAN] | 4 | 2 | 3 | 531 | 60.5 | 9.61 |
| Q9H4M9 | EH domain-containing protein 1 OS=Homo sapiens GN=EHD1 PE=1 SV=2 - [EHD1_HUMAN] | 4 | 2 | 3 | 534 | 60.6 | 6.83 |
| P07686 | Beta-hexosaminidase subunit beta OS=Homo sapiens GN=HEXB PE=1 SV=3 - [HEXB_HUMAN] | 4 | 2 | 3 | 556 | 63.1 | 6.76 |
| Q13409 | Cytoplasmic dynein 1 intermediate chain 2 OS=Homo sapiens GN=DYNC1I2 PE=1 SV=3 - [DC1I2_HUMAN] | 4 | 2 | 3 | 638 | 71.4 | 5.2 |
| Q01581 | Hydroxymethylglutaryl-CoA synthase, cytoplasmic OS=Homo sapiens GN=HMGCS1 PE=1 SV=2 - [HMCS1_HUMAN] | 4 | 2 | 3 | 520 | 57.3 | 5.41 |
| Q9Y639 | Neuroplastin OS=Homo sapiens GN=NPTN PE=1 SV=2 - [NPTN_HUMAN] | 4 | 2 | 3 | 398 | 44.4 | 7.99 |
| Q16537 | Serine/threonine-protein phosphatase 2A 56 kDa regulatory subunit epsilon isoform OS=Homo sapiens GN=PPP2R5E PE=1 SV=1 - [2A5E_HUMAN] | 4 | 2 | 3 | 467 | 54.7 | 6.95 |
| O43172 | U4/U6 small nuclear ribonucleoprotein Prp4 OS=Homo sapiens GN=PRPF4 PE=1 SV=2 - [PRP4_HUMAN] | 4 | 2 | 3 | 522 | 58.4 | 7.42 |
| Q96CX6 | Leucine-rich repeat-containing protein 58 OS=Homo sapiens GN=LRRC58 PE=1 SV=2 - [LRC58_HUMAN] | 4 | 2 | 3 | 371 | 40.6 | 6.84 |
| P30533 | Alpha-2-macroglobulin receptor-associated protein OS=Homo sapiens GN=LRPAP1 PE=1 SV=1 - [AMRP_HUMAN] | 4 | 2 | 3 | 357 | 41.4 | 8.78 |
| P06280 | Alpha-galactosidase A OS=Homo sapiens GN=GLA PE=1 SV=1 - [AGAL_HUMAN] | 4 | 2 | 3 | 429 | 48.7 | 5.6 |
| Q96GD0 | Pyridoxal phosphate phosphatase OS=Homo sapiens GN=PDXP PE=1 SV=2 - [PLPP_HUMAN] | 4 | 2 | 3 | 296 | 31.7 | 6.55 |
| Q9GZZ1 | N-acetyltransferase 13 OS=Homo sapiens GN=NAT13 PE=1 SV=1 - [NAT13_HUMAN] | 4 | 2 | 3 | 169 | 19.4 | 8.81 |
| Q92688 | Acidic leucine-rich nuclear phosphoprotein 32 family member B OS=Homo sapiens GN=ANP32B PE=1 SV=1 - [AN32B_HUMAN] | 4 | 9 | 13 | 251 | 28.8 | 4.06 |
| O75821 | Eukaryotic translation initiation factor 3 subunit G OS=Homo sapiens GN=EIF3G PE=1 SV=2 - [EIF3G_HUMAN] | 4 | 5 | 7 | 320 | 35.6 | 6.13 |
| P07814 | Bifunctional aminoacyl-tRNA synthetase OS=Homo sapiens GN=EPRS PE=1 SV=5 - [SYEP_HUMAN] | 4 | 3 | 4 | 1512 | 170.5 | 7.33 |
| Q5T8P6 | RNA-binding protein 26 OS=Homo sapiens GN=RBM26 PE=1 SV=3 - [RBM26_HUMAN] | 4 | 3 | 4 | 1007 | 113.5 | 9.16 |
| Q9H074 | Polyadenylate-binding protein-interacting protein 1 OS=Homo sapiens GN=PAIP1 PE=1 SV=1 - [PAIP1_HUMAN] | 4 | 3 | 4 | 479 | 53.5 | 4.81 |
| P23786 | Carnitine O-palmitoyltransferase 2, mitochondrial OS=Homo sapiens GN=CPT2 PE=1 SV=2 - [CPT2_HUMAN] | 4 | 3 | 4 | 658 | 73.7 | 8.18 |
| P48444 | Coatomer subunit delta OS=Homo sapiens GN=ARCN1 PE=1 SV=1 - [COPD_HUMAN] | 4 | 3 | 4 | 511 | 57.2 | 6.21 |
| P42785 | Lysosomal Pro-X carboxypeptidase OS=Homo sapiens GN=PRCP PE=1 SV=1 - [PCP_HUMAN] | 4 | 3 | 4 | 496 | 55.8 | 7.21 |
| P15586 | N-acetylglucosamine-6-sulfatase OS=Homo sapiens GN=GNS PE=1 SV=3 - [GNS_HUMAN] | 4 | 3 | 4 | 552 | 62 | 8.31 |
| Q9UBS4 | DnaJ homolog subfamily B member 11 OS=Homo sapiens GN=DNAJB11 PE=1 SV=1 - [DJB11_HUMAN] | 4 | 3 | 4 | 358 | 40.5 | 6.18 |
| Q9Y277 | Voltage-dependent anion-selective channel protein 3 OS=Homo sapiens GN=VDAC3 PE=1 SV=1 - [VDAC3_HUMAN] | 4 | 3 | 4 | 283 | 30.6 | 8.66 |
| Q52LJ0 | Protein FAM98B OS=Homo sapiens GN=FAM98B PE=1 SV=1 - [FA98B_HUMAN] | 4 | 3 | 4 | 330 | 37.2 | 6.29 |
| P35250 | Replication factor C subunit 2 OS=Homo sapiens GN=RFC2 PE=1 SV=3 - [RFC2_HUMAN] | 4 | 3 | 4 | 354 | 39.1 | 6.44 |
| P24752 | Acetyl-CoA acetyltransferase, mitochondrial OS=Homo sapiens GN=ACAT1 PE=1 SV=1 - [THIL_HUMAN] | 4 | 3 | 4 | 427 | 45.2 | 8.85 |
| Q6FI81 | Anamorsin OS=Homo sapiens GN=CIAPIN1 PE=1 SV=2 - [CPIN1_HUMAN] | 4 | 3 | 4 | 312 | 33.6 | 5.62 |
| Q96A49 | Synapse-associated protein 1 OS=Homo sapiens GN=SYAP1 PE=1 SV=1 - [SYAP1_HUMAN] | 4 | 3 | 4 | 352 | 39.9 | 4.53 |
| Q9NPD8 | Ubiquitin-conjugating enzyme E2 T OS=Homo sapiens GN=UBE2T PE=1 SV=1 - [UBE2T_HUMAN] | 4 | 3 | 4 | 197 | 22.5 | 7.99 |
| Q15056 | Eukaryotic translation initiation factor 4H OS=Homo sapiens GN=EIF4H PE=1 SV=5 - [IF4H_HUMAN] | 4 | 3 | 4 | 248 | 27.4 | 7.23 |
| P07305 | Histone H1.0 OS=Homo sapiens GN=H1F0 PE=1 SV=3 - [H10_HUMAN] | 4 | 3 | 4 | 194 | 20.9 | 10.84 |
| P61086 | Ubiquitin-conjugating enzyme E2 K OS=Homo sapiens GN=UBE2K PE=1 SV=3 - [UBE2K_HUMAN] | 4 | 3 | 4 | 200 | 22.4 | 5.44 |
| P63208 | S-phase kinase-associated protein 1 OS=Homo sapiens GN=SKP1 PE=1 SV=2 - [SKP1_HUMAN] | 4 | 3 | 4 | 163 | 18.6 | 4.54 |
| Q9Y5S9 | RNA-binding protein 8A OS=Homo sapiens GN=RBM8A PE=1 SV=1 - [RBM8A_HUMAN] | 4 | 3 | 4 | 174 | 19.9 | 5.72 |
| P09493 | Tropomyosin alpha-1 chain OS=Homo sapiens GN=TPM1 PE=1 SV=2 - [TPM1_HUMAN] | 4 | 10 | 13 | 284 | 32.7 | 4.74 |
| P06753 | Tropomyosin alpha-3 chain OS=Homo sapiens GN=TPM3 PE=1 SV=2 - [TPM3_HUMAN] | 4 | 10 | 13 | 285 | 32.9 | 4.72 |
| Q9Y2W2 | WW domain-binding protein 11 OS=Homo sapiens GN=WBP11 PE=1 SV=1 - [WBP11_HUMAN] | 4 | 4 | 5 | 641 | 70 | 8.38 |
| P50914 | 60S ribosomal protein L14 OS=Homo sapiens GN=RPL14 PE=1 SV=4 - [RL14_HUMAN] | 4 | 4 | 5 | 215 | 23.4 | 10.93 |
| Q9NUQ9 | Protein FAM49B OS=Homo sapiens GN=FAM49B PE=1 SV=1 - [FA49B_HUMAN] | 4 | 4 | 5 | 324 | 36.7 | 6.06 |
| Q01081 | Splicing factor U2AF 35 kDa subunit OS=Homo sapiens GN=U2AF1 PE=1 SV=3 - [U2AF1_HUMAN] | 4 | 4 | 5 | 240 | 27.9 | 8.81 |
| Q13011 | Delta(3,5)-Delta(2,4)-dienoyl-CoA isomerase, mitochondrial OS=Homo sapiens GN=ECH1 PE=1 SV=2 - [ECH1_HUMAN] | 4 | 4 | 5 | 328 | 35.8 | 8 |
| Q13155 | Aminoacyl tRNA synthetase complex-interacting multifunctional protein 2 OS=Homo sapiens GN=AIMP2 PE=1 SV=2 - [AIMP2_HUMAN] | 4 | 4 | 5 | 320 | 35.3 | 8.22 |
| Q13838 | Spliceosome RNA helicase BAT1 OS=Homo sapiens GN=BAT1 PE=1 SV=1 - [UAP56_HUMAN] | 4 | 5 | 6 | 428 | 49 | 5.67 |
| P68036 | Ubiquitin-conjugating enzyme E2 L3 OS=Homo sapiens GN=UBE2L3 PE=1 SV=1 - [UB2L3_HUMAN] | 4 | 5 | 6 | 154 | 17.9 | 8.51 |
| P04632 | Calpain small subunit 1 OS=Homo sapiens GN=CAPNS1 PE=1 SV=1 - [CPNS1_HUMAN] | 4 | 7 | 8 | 268 | 28.3 | 5.2 |
| P07437 | Tubulin beta chain OS=Homo sapiens GN=TUBB PE=1 SV=2 - [TBB5_HUMAN] | 4 | 79 | 89 | 444 | 49.6 | 4.89 |
| P52597 | Heterogeneous nuclear ribonucleoprotein F OS=Homo sapiens GN=HNRNPF PE=1 SV=3 - [HNRPF_HUMAN] | 4 | 10 | 11 | 415 | 45.6 | 5.58 |
| Q9BUF5 | Tubulin beta-6 chain OS=Homo sapiens GN=TUBB6 PE=1 SV=1 - [TBB6_HUMAN] | 4 | 28 | 28 | 446 | 49.8 | 4.88 |
| Q15717 | ELAV-like protein 1 OS=Homo sapiens GN=ELAVL1 PE=1 SV=2 - [ELAV1_HUMAN] | 4 | 5 | 5 | 326 | 36.1 | 9.17 |
| P62851 | 40S ribosomal protein S25 OS=Homo sapiens GN=RPS25 PE=1 SV=1 - [RS25_HUMAN] | 4 | 5 | 5 | 125 | 13.7 | 10.11 |
| P53999 | Activated RNA polymerase II transcriptional coactivator p15 OS=Homo sapiens GN=SUB1 PE=1 SV=3 - [TCP4_HUMAN] | 4 | 5 | 5 | 127 | 14.4 | 9.6 |
| O14639 | Actin-binding LIM protein 1 OS=Homo sapiens GN=ABLIM1 PE=1 SV=3 - [ABLM1_HUMAN] | 4 | 4 | 4 | 778 | 87.6 | 8.59 |
| Q08209 | Serine/threonine-protein phosphatase 2B catalytic subunit alpha isoform OS=Homo sapiens GN=PPP3CA PE=1 SV=1 - [PP2BA_HUMAN] | 4 | 4 | 4 | 521 | 58.7 | 5.86 |
| P52594 | Arf-GAP domain and FG repeats-containing protein 1 OS=Homo sapiens GN=AGFG1 PE=1 SV=2 - [AGFG1_HUMAN] | 4 | 4 | 4 | 562 | 58.2 | 8.63 |
| P46777 | 60S ribosomal protein L5 OS=Homo sapiens GN=RPL5 PE=1 SV=3 - [RL5_HUMAN] | 4 | 4 | 4 | 297 | 34.3 | 9.72 |
| P50402 | Emerin OS=Homo sapiens GN=EMD PE=1 SV=1 - [EMD_HUMAN] | 4 | 4 | 4 | 254 | 29 | 5.5 |
| P62750 | 60S ribosomal protein L23a OS=Homo sapiens GN=RPL23A PE=1 SV=1 - [RL23A_HUMAN] | 4 | 4 | 4 | 156 | 17.7 | 10.45 |
| O43399 | Tumor protein D54 OS=Homo sapiens GN=TPD52L2 PE=1 SV=2 - [TPD54_HUMAN] | 4 | 4 | 4 | 206 | 22.2 | 5.36 |
| P55786 | Puromycin-sensitive aminopeptidase OS=Homo sapiens GN=NPEPPS PE=1 SV=2 - [PSA_HUMAN] | 4 | 3 | 3 | 919 | 103.2 | 5.72 |
| P35659 | Protein DEK OS=Homo sapiens GN=DEK PE=1 SV=1 - [DEK_HUMAN] | 4 | 3 | 3 | 375 | 42.6 | 8.56 |
| P10155 | 60 kDa SS-A/Ro ribonucleoprotein OS=Homo sapiens GN=TROVE2 PE=1 SV=2 - [RO60_HUMAN] | 4 | 3 | 3 | 538 | 60.6 | 8.03 |
| Q8WX92 | Negative elongation factor B OS=Homo sapiens GN=COBRA1 PE=1 SV=1 - [NELFB_HUMAN] | 4 | 3 | 3 | 580 | 65.7 | 6.13 |
| P49257 | Protein ERGIC-53 OS=Homo sapiens GN=LMAN1 PE=1 SV=2 - [LMAN1_HUMAN] | 4 | 3 | 3 | 510 | 57.5 | 6.77 |
| Q13867 | Bleomycin hydrolase OS=Homo sapiens GN=BLMH PE=1 SV=1 - [BLMH_HUMAN] | 4 | 3 | 3 | 455 | 52.5 | 6.27 |
| P31483 | Nucleolysin TIA-1 isoform p40 OS=Homo sapiens GN=TIA1 PE=1 SV=3 - [TIA1_HUMAN] | 4 | 3 | 3 | 386 | 42.9 | 7.74 |
| P48556 | 26S proteasome non-ATPase regulatory subunit 8 OS=Homo sapiens GN=PSMD8 PE=1 SV=2 - [PSMD8_HUMAN] | 4 | 3 | 3 | 350 | 39.6 | 9.7 |
| Q9Y570 | Protein phosphatase methylesterase 1 OS=Homo sapiens GN=PPME1 PE=1 SV=3 - [PPME1_HUMAN] | 4 | 3 | 3 | 386 | 42.3 | 5.97 |
| Q15942 | Zyxin OS=Homo sapiens GN=ZYX PE=1 SV=1 - [ZYX_HUMAN] | 4 | 3 | 3 | 572 | 61.2 | 6.67 |
| Q13557 | Calcium/calmodulin-dependent protein kinase type II subunit delta OS=Homo sapiens GN=CAMK2D PE=1 SV=3 - [KCC2D_HUMAN] | 4 | 3 | 3 | 499 | 56.3 | 7.25 |
| Q13510 | Acid ceramidase OS=Homo sapiens GN=ASAH1 PE=1 SV=5 - [ASAH1_HUMAN] | 4 | 3 | 3 | 395 | 44.6 | 7.62 |
| Q96EP5 | DAZ-associated protein 1 OS=Homo sapiens GN=DAZAP1 PE=1 SV=1 - [DAZP1_HUMAN] | 4 | 3 | 3 | 407 | 43.4 | 8.56 |
| P51398 | 28S ribosomal protein S29, mitochondrial OS=Homo sapiens GN=DAP3 PE=1 SV=1 - [RT29_HUMAN] | 4 | 3 | 3 | 398 | 45.5 | 8.88 |
| P53004 | Biliverdin reductase A OS=Homo sapiens GN=BLVRA PE=1 SV=2 - [BIEA_HUMAN] | 4 | 3 | 3 | 296 | 33.4 | 6.44 |
| Q9Y3A5 | Ribosome maturation protein SBDS OS=Homo sapiens GN=SBDS PE=1 SV=4 - [SBDS_HUMAN] | 4 | 3 | 3 | 250 | 28.7 | 8.75 |
| Q96HY6 | DDRGK domain-containing protein 1 OS=Homo sapiens GN=DDRGK1 PE=1 SV=2 - [DDRGK_HUMAN] | 4 | 3 | 3 | 314 | 35.6 | 5.12 |
| Q6PJG6 | HEAT repeat-containing protein C7orf27 OS=Homo sapiens GN=C7orf27 PE=1 SV=2 - [CG027_HUMAN] | 4 | 2 | 2 | 821 | 88.1 | 5.27 |
| Q1KMD3 | Heterogeneous nuclear ribonucleoprotein U-like protein 2 OS=Homo sapiens GN=HNRNPUL2 PE=1 SV=1 - [HNRL2_HUMAN] | 4 | 2 | 2 | 747 | 85.1 | 4.91 |
| Q15007 | Pre-mRNA-splicing regulator WTAP OS=Homo sapiens GN=WTAP PE=1 SV=2 - [FL2D_HUMAN] | 4 | 2 | 2 | 396 | 44.2 | 5.19 |
| P49773 | Histidine triad nucleotide-binding protein 1 OS=Homo sapiens GN=HINT1 PE=1 SV=2 - [HINT1_HUMAN] | 4 | 2 | 2 | 126 | 13.8 | 6.95 |
| Q16186 | Proteasomal ubiquitin receptor ADRM1 OS=Homo sapiens GN=ADRM1 PE=1 SV=2 - [ADRM1_HUMAN] | 4 | 5 | 4 | 407 | 42.1 | 5.07 |
| P68104 | Elongation factor 1-alpha 1 OS=Homo sapiens GN=EEF1A1 PE=1 SV=1 - [EF1A1_HUMAN] | 4 | 18 | 14 | 462 | 50.1 | 9.01 |
| Q99460 | 26S proteasome non-ATPase regulatory subunit 1 OS=Homo sapiens GN=PSMD1 PE=1 SV=2 - [PSMD1_HUMAN] | 4 | 4 | 3 | 953 | 105.8 | 5.39 |
| Q8WXF1 | Paraspeckle component 1 OS=Homo sapiens GN=PSPC1 PE=1 SV=1 - [PSPC1_HUMAN] | 4 | 4 | 3 | 523 | 58.7 | 6.67 |
| Q9Y282 | Endoplasmic reticulum-Golgi intermediate compartment protein 3 OS=Homo sapiens GN=ERGIC3 PE=1 SV=1 - [ERGI3_HUMAN] | 4 | 4 | 3 | 383 | 43.2 | 6.06 |
| O43684 | Mitotic checkpoint protein BUB3 OS=Homo sapiens GN=BUB3 PE=1 SV=1 - [BUB3_HUMAN] | 4 | 4 | 3 | 328 | 37.1 | 6.84 |
| O60664 | Perilipin-3 OS=Homo sapiens GN=PLIN3 PE=1 SV=3 - [PLIN3_HUMAN] | 4 | 4 | 3 | 434 | 47 | 5.44 |
| Q969G3 | SWI/SNF-related matrix-associated actin-dependent regulator of chromatin subfamily E member 1 OS=Homo sapiens GN=SMARCE1 PE=1 SV=2 - [SMCE1_HUMAN] | 4 | 4 | 3 | 411 | 46.6 | 4.88 |
| Q5ZPR3 | CD276 antigen OS=Homo sapiens GN=CD276 PE=1 SV=1 - [CD276_HUMAN] | 4 | 4 | 3 | 534 | 57.2 | 4.91 |
| O00273 | DNA fragmentation factor subunit alpha OS=Homo sapiens GN=DFFA PE=1 SV=1 - [DFFA_HUMAN] | 4 | 4 | 3 | 331 | 36.5 | 4.79 |
| P55735 | Protein SEC13 homolog OS=Homo sapiens GN=SEC13 PE=1 SV=3 - [SEC13_HUMAN] | 4 | 4 | 3 | 322 | 35.5 | 5.48 |
| P69905 | Hemoglobin subunit alpha OS=Homo sapiens GN=HBA1 PE=1 SV=2 - [HBA_HUMAN] | 4 | 4 | 3 | 142 | 15.2 | 8.68 |
| O60282 | Kinesin heavy chain isoform 5C OS=Homo sapiens GN=KIF5C PE=1 SV=1 - [KIF5C_HUMAN] | 4 | 7 | 5 | 957 | 109.4 | 6.19 |
| P31942 | Heterogeneous nuclear ribonucleoprotein H3 OS=Homo sapiens GN=HNRNPH3 PE=1 SV=2 - [HNRH3_HUMAN] | 4 | 7 | 5 | 346 | 36.9 | 6.87 |
| Q09028 | Histone-binding protein RBBP4 OS=Homo sapiens GN=RBBP4 PE=1 SV=3 - [RBBP4_HUMAN] | 4 | 10 | 7 | 425 | 47.6 | 4.89 |
| P52701 | DNA mismatch repair protein Msh6 OS=Homo sapiens GN=MSH6 PE=1 SV=2 - [MSH6_HUMAN] | 4 | 3 | 2 | 1360 | 152.7 | 6.9 |
| O60763 | General vesicular transport factor p115 OS=Homo sapiens GN=USO1 PE=1 SV=2 - [USO1_HUMAN] | 4 | 3 | 2 | 962 | 107.8 | 4.91 |
| P47897 | Glutaminyl-tRNA synthetase OS=Homo sapiens GN=QARS PE=1 SV=1 - [SYQ_HUMAN] | 4 | 3 | 2 | 775 | 87.7 | 7.15 |
| Q92541 | RNA polymerase-associated protein RTF1 homolog OS=Homo sapiens GN=RTF1 PE=1 SV=4 - [RTF1_HUMAN] | 4 | 3 | 2 | 710 | 80.3 | 8.15 |
| Q9BZK7 | F-box-like/WD repeat-containing protein TBL1XR1 OS=Homo sapiens GN=TBL1XR1 PE=1 SV=1 - [TBL1R_HUMAN] | 4 | 3 | 2 | 514 | 55.6 | 5.55 |
| P78330 | Phosphoserine phosphatase OS=Homo sapiens GN=PSPH PE=1 SV=2 - [SERB_HUMAN] | 4 | 3 | 2 | 225 | 25 | 5.69 |
| Q53H12 | Acylglycerol kinase, mitochondrial OS=Homo sapiens GN=AGK PE=1 SV=2 - [AGK_HUMAN] | 4 | 3 | 2 | 422 | 47.1 | 8.09 |
| P48507 | Glutamate--cysteine ligase regulatory subunit OS=Homo sapiens GN=GCLM PE=1 SV=1 - [GSH0_HUMAN] | 4 | 3 | 2 | 274 | 30.7 | 6.02 |
| Q13404 | Ubiquitin-conjugating enzyme E2 variant 1 OS=Homo sapiens GN=UBE2V1 PE=1 SV=2 - [UB2V1_HUMAN] | 4 | 3 | 2 | 147 | 16.5 | 7.93 |
| P35580 | Myosin-10 OS=Homo sapiens GN=MYH10 PE=1 SV=3 - [MYH10_HUMAN] | 4 | 8 | 5 | 1976 | 228.9 | 5.54 |
| Q8TAQ2 | SWI/SNF complex subunit SMARCC2 OS=Homo sapiens GN=SMARCC2 PE=1 SV=1 - [SMRC2_HUMAN] | 4 | 8 | 5 | 1214 | 132.8 | 5.69 |
| Q14108 | Lysosome membrane protein 2 OS=Homo sapiens GN=SCARB2 PE=1 SV=2 - [SCRB2_HUMAN] | 4 | 5 | 3 | 478 | 54.3 | 5.14 |
| Q15370 | Transcription elongation factor B polypeptide 2 OS=Homo sapiens GN=TCEB2 PE=1 SV=1 - [ELOB_HUMAN] | 4 | 5 | 3 | 118 | 13.1 | 4.88 |
| O95299 | NADH dehydrogenase [ubiquinone] 1 alpha subcomplex subunit 10, mitochondrial OS=Homo sapiens GN=NDUFA10 PE=1 SV=1 - [NDUAA_HUMAN] | 4 | 4 | 2 | 355 | 40.7 | 8.48 |
| Q10471 | Polypeptide N-acetylgalactosaminyltransferase 2 OS=Homo sapiens GN=GALNT2 PE=1 SV=1 - [GALT2_HUMAN] | 4 | 3 | 1 | 571 | 64.7 | 8.35 |
| P19823 | Inter-alpha-trypsin inhibitor heavy chain H2 OS=Homo sapiens GN=ITIH2 PE=1 SV=2 - [ITIH2_HUMAN] | 4 | 4 | 1 | 946 | 106.4 | 6.86 |
| P42224 | Signal transducer and activator of transcription 1-alpha/beta OS=Homo sapiens GN=STAT1 PE=1 SV=2 - [STAT1_HUMAN] | 4 | 4 | 1 | 750 | 87.3 | 6.05 |
| O43242 | 26S proteasome non-ATPase regulatory subunit 3 OS=Homo sapiens GN=PSMD3 PE=1 SV=2 - [PSMD3_HUMAN] | 4 | 4 | 1 | 534 | 60.9 | 8.44 |
| P21397 | Amine oxidase [flavin-containing] A OS=Homo sapiens GN=MAOA PE=1 SV=1 - [AOFA_HUMAN] | 4 | 4 | 1 | 527 | 59.6 | 7.85 |
| P62316 | Small nuclear ribonucleoprotein Sm D2 OS=Homo sapiens GN=SNRPD2 PE=1 SV=1 - [SMD2_HUMAN] | 4 | 5 | 1 | 118 | 13.5 | 9.91 |
| P01111 | GTPase NRas OS=Homo sapiens GN=NRAS PE=1 SV=1 - [RASN_HUMAN] | 3 | 0 | 3 | 189 | 21.2 | 5.17 |
| Q9NP72 | Ras-related protein Rab-18 OS=Homo sapiens GN=RAB18 PE=1 SV=1 - [RAB18_HUMAN] | 3 | 0 | 3 | 206 | 23 | 5.24 |
| O94811 | Tubulin polymerization-promoting protein OS=Homo sapiens GN=TPPP PE=1 SV=1 - [TPPP_HUMAN] | 3 | 0 | 3 | 219 | 23.7 | 9.44 |
| Q9BUL8 | Programmed cell death protein 10 OS=Homo sapiens GN=PDCD10 PE=1 SV=1 - [PDC10_HUMAN] | 3 | 0 | 3 | 212 | 24.7 | 8.19 |
| P61019 | Ras-related protein Rab-2A OS=Homo sapiens GN=RAB2A PE=1 SV=1 - [RAB2A_HUMAN] | 3 | 0 | 3 | 212 | 23.5 | 6.54 |
| Q96C19 | EF-hand domain-containing protein D2 OS=Homo sapiens GN=EFHD2 PE=1 SV=1 - [EFHD2_HUMAN] | 3 | 0 | 3 | 240 | 26.7 | 5.2 |
| Q9BUR5 | MICOS complex subunit MIC26 OS=Homo sapiens GN=APOO PE=1 SV=1 - [MIC26_HUMAN] | 3 | 0 | 3 | 198 | 22.3 | 9.13 |
| Q9Y3B8 | Oligoribonuclease, mitochondrial OS=Homo sapiens GN=REXO2 PE=1 SV=3 - [ORN_HUMAN] | 3 | 0 | 3 | 237 | 26.8 | 6.87 |
| Q9BX40 | Protein LSM14 homolog B OS=Homo sapiens GN=LSM14B PE=1 SV=1 - [LS14B_HUMAN] | 3 | 0 | 3 | 385 | 42 | 9.69 |
| O75569 | Interferon-inducible double stranded RNA-dependent protein kinase activator A OS=Homo sapiens GN=PRKRA PE=1 SV=1 - [PRKRA_HUMAN] | 3 | 0 | 3 | 313 | 34.4 | 8.41 |
| P09543 | 2',3'-cyclic-nucleotide 3'-phosphodiesterase OS=Homo sapiens GN=CNP PE=1 SV=2 - [CN37_HUMAN] | 3 | 0 | 3 | 421 | 47.5 | 9.07 |
| Q12849 | G-rich sequence factor 1 OS=Homo sapiens GN=GRSF1 PE=1 SV=3 - [GRSF1_HUMAN] | 3 | 0 | 3 | 480 | 53.1 | 6.19 |
| Q9BXK5 | Bcl-2-like protein 13 OS=Homo sapiens GN=BCL2L13 PE=1 SV=1 - [B2L13_HUMAN] | 3 | 0 | 3 | 485 | 52.7 | 4.44 |
| P49821 | NADH dehydrogenase [ubiquinone] flavoprotein 1, mitochondrial OS=Homo sapiens GN=NDUFV1 PE=1 SV=4 - [NDUV1_HUMAN] | 3 | 0 | 3 | 464 | 50.8 | 8.21 |
| Q92791 | Synaptonemal complex protein SC65 OS=Homo sapiens GN=SC65 PE=1 SV=1 - [SC65_HUMAN] | 3 | 0 | 3 | 437 | 50.3 | 4.77 |
| Q96TC7 | Regulator of microtubule dynamics protein 3 OS=Homo sapiens GN=FAM82A2 PE=1 SV=2 - [RMD3_HUMAN] | 3 | 0 | 3 | 470 | 52.1 | 5.1 |
| O00566 | U3 small nucleolar ribonucleoprotein protein MPP10 OS=Homo sapiens GN=MPHOSPH10 PE=1 SV=2 - [MPP10_HUMAN] | 3 | 0 | 3 | 681 | 78.8 | 4.86 |
| Q14738 | Serine/threonine-protein phosphatase 2A 56 kDa regulatory subunit delta isoform OS=Homo sapiens GN=PPP2R5D PE=1 SV=1 - [2A5D_HUMAN] | 3 | 0 | 3 | 602 | 69.9 | 8.13 |
| P06865 | Beta-hexosaminidase subunit alpha OS=Homo sapiens GN=HEXA PE=1 SV=2 - [HEXA_HUMAN] | 3 | 0 | 3 | 529 | 60.7 | 5.16 |
| Q9NZT2 | Opioid growth factor receptor OS=Homo sapiens GN=OGFR PE=1 SV=3 - [OGFR_HUMAN] | 3 | 0 | 3 | 677 | 73.3 | 4.84 |
| Q9Y6A5 | Transforming acidic coiled-coil-containing protein 3 OS=Homo sapiens GN=TACC3 PE=1 SV=1 - [TACC3_HUMAN] | 3 | 0 | 3 | 838 | 90.3 | 5.05 |
| O14974 | Protein phosphatase 1 regulatory subunit 12A OS=Homo sapiens GN=PPP1R12A PE=1 SV=1 - [MYPT1_HUMAN] | 3 | 0 | 3 | 1030 | 115.2 | 5.4 |
| P49589 | Cysteinyl-tRNA synthetase, cytoplasmic OS=Homo sapiens GN=CARS PE=1 SV=3 - [SYCC_HUMAN] | 3 | 0 | 3 | 748 | 85.4 | 6.76 |
| Q8IX12 | Cell division cycle and apoptosis regulator protein 1 OS=Homo sapiens GN=CCAR1 PE=1 SV=2 - [CCAR1_HUMAN] | 3 | 0 | 3 | 1150 | 132.7 | 5.76 |
| Q9NRL2 | Bromodomain adjacent to zinc finger domain protein 1A OS=Homo sapiens GN=BAZ1A PE=1 SV=2 - [BAZ1A_HUMAN] | 3 | 0 | 3 | 1556 | 178.6 | 6.6 |
| Q9NTZ6 | RNA-binding protein 12 OS=Homo sapiens GN=RBM12 PE=1 SV=1 - [RBM12_HUMAN] | 3 | 0 | 3 | 932 | 97.3 | 8.63 |
| P08574 | Cytochrome c1, heme protein, mitochondrial OS=Homo sapiens GN=CYC1 PE=1 SV=3 - [CY1_HUMAN] | 3 | 1 | 6 | 325 | 35.4 | 9 |
| Q8NHH9 | Atlastin-2 OS=Homo sapiens GN=ATL2 PE=1 SV=2 - [ATLA2_HUMAN] | 3 | 1 | 5 | 583 | 66.2 | 5.48 |
| Q9BY32 | Inosine triphosphate pyrophosphatase OS=Homo sapiens GN=ITPA PE=1 SV=2 - [ITPA_HUMAN] | 3 | 1 | 5 | 194 | 21.4 | 5.66 |
| Q16850 | Lanosterol 14-alpha demethylase OS=Homo sapiens GN=CYP51A1 PE=1 SV=3 - [CP51A_HUMAN] | 3 | 1 | 4 | 503 | 56.8 | 8.53 |
| O75439 | Mitochondrial-processing peptidase subunit beta OS=Homo sapiens GN=PMPCB PE=1 SV=2 - [MPPB_HUMAN] | 3 | 1 | 4 | 489 | 54.3 | 6.83 |
| P82930 | 28S ribosomal protein S34, mitochondrial OS=Homo sapiens GN=MRPS34 PE=1 SV=2 - [RT34_HUMAN] | 3 | 1 | 4 | 218 | 25.6 | 9.98 |
| P04179 | Superoxide dismutase [Mn], mitochondrial OS=Homo sapiens GN=SOD2 PE=1 SV=2 - [SODM_HUMAN] | 3 | 1 | 4 | 222 | 24.7 | 8.25 |
| P51571 | Translocon-associated protein subunit delta OS=Homo sapiens GN=SSR4 PE=1 SV=1 - [SSRD_HUMAN] | 3 | 1 | 4 | 173 | 19 | 6.15 |
| P06730 | Eukaryotic translation initiation factor 4E OS=Homo sapiens GN=EIF4E PE=1 SV=2 - [IF4E_HUMAN] | 3 | 1 | 4 | 217 | 25.1 | 6.15 |
| P13693 | Translationally-controlled tumor protein OS=Homo sapiens GN=TPT1 PE=1 SV=1 - [TCTP_HUMAN] | 3 | 1 | 4 | 172 | 19.6 | 4.93 |
| P63279 | SUMO-conjugating enzyme UBC9 OS=Homo sapiens GN=UBE2I PE=1 SV=1 - [UBC9_HUMAN] | 3 | 1 | 4 | 158 | 18 | 8.66 |
| Q3V6T2 | Girdin OS=Homo sapiens GN=CCDC88A PE=1 SV=2 - [GRDN_HUMAN] | 3 | 1 | 3 | 1871 | 215.9 | 6.21 |
| Q7Z6Z7 | E3 ubiquitin-protein ligase HUWE1 OS=Homo sapiens GN=HUWE1 PE=1 SV=3 - [HUWE1_HUMAN] | 3 | 1 | 3 | 4374 | 481.6 | 5.22 |
| P35658 | Nuclear pore complex protein Nup214 OS=Homo sapiens GN=NUP214 PE=1 SV=2 - [NU214_HUMAN] | 3 | 1 | 3 | 2090 | 213.5 | 7.47 |
| P08069 | Insulin-like growth factor 1 receptor OS=Homo sapiens GN=IGF1R PE=1 SV=1 - [IGF1R_HUMAN] | 3 | 1 | 3 | 1367 | 154.7 | 5.8 |
| P52789 | Hexokinase-2 OS=Homo sapiens GN=HK2 PE=1 SV=2 - [HXK2_HUMAN] | 3 | 1 | 3 | 917 | 102.3 | 6.05 |
| Q9UMS6 | Synaptopodin-2 OS=Homo sapiens GN=SYNPO2 PE=1 SV=2 - [SYNP2_HUMAN] | 3 | 1 | 3 | 1093 | 117.4 | 8.57 |
| P51610 | Host cell factor 1 OS=Homo sapiens GN=HCFC1 PE=1 SV=2 - [HCFC1_HUMAN] | 3 | 1 | 3 | 2035 | 208.6 | 7.46 |
| P31040 | Succinate dehydrogenase [ubiquinone] flavoprotein subunit, mitochondrial OS=Homo sapiens GN=SDHA PE=1 SV=2 - [DHSA_HUMAN] | 3 | 1 | 3 | 664 | 72.6 | 7.39 |
| Q32MZ4 | Leucine-rich repeat flightless-interacting protein 1 OS=Homo sapiens GN=LRRFIP1 PE=1 SV=2 - [LRRF1_HUMAN] | 3 | 1 | 3 | 808 | 89.2 | 4.65 |
| Q96DV4 | 39S ribosomal protein L38, mitochondrial OS=Homo sapiens GN=MRPL38 PE=1 SV=2 - [RM38_HUMAN] | 3 | 1 | 3 | 380 | 44.6 | 7.53 |
| P06132 | Uroporphyrinogen decarboxylase OS=Homo sapiens GN=UROD PE=1 SV=2 - [DCUP_HUMAN] | 3 | 1 | 3 | 367 | 40.8 | 6.14 |
| P36551 | Coproporphyrinogen-III oxidase, mitochondrial OS=Homo sapiens GN=CPOX PE=1 SV=3 - [HEM6_HUMAN] | 3 | 1 | 3 | 454 | 50.1 | 8.25 |
| P14868 | Aspartyl-tRNA synthetase, cytoplasmic OS=Homo sapiens GN=DARS PE=1 SV=2 - [SYDC_HUMAN] | 3 | 1 | 3 | 501 | 57.1 | 6.55 |
| Q8N2K0 | Monoacylglycerol lipase ABHD12 OS=Homo sapiens GN=ABHD12 PE=2 SV=2 - [ABD12_HUMAN] | 3 | 1 | 3 | 398 | 45.1 | 8.65 |
| Q9BUJ2 | Heterogeneous nuclear ribonucleoprotein U-like protein 1 OS=Homo sapiens GN=HNRNPUL1 PE=1 SV=2 - [HNRL1_HUMAN] | 3 | 1 | 3 | 856 | 95.7 | 6.92 |
| Q8IZ40 | REST corepressor 2 OS=Homo sapiens GN=RCOR2 PE=2 SV=2 - [RCOR2_HUMAN] | 3 | 1 | 3 | 523 | 58 | 9.01 |
| P08559 | Pyruvate dehydrogenase E1 component subunit alpha, somatic form, mitochondrial OS=Homo sapiens GN=PDHA1 PE=1 SV=3 - [ODPA_HUMAN] | 3 | 1 | 3 | 390 | 43.3 | 8.06 |
| Q15404 | Ras suppressor protein 1 OS=Homo sapiens GN=RSU1 PE=1 SV=3 - [RSU1_HUMAN] | 3 | 1 | 3 | 277 | 31.5 | 8.65 |
| Q6UX04 | Peptidyl-prolyl cis-trans isomerase CWC27 homolog OS=Homo sapiens GN=CWC27 PE=1 SV=1 - [CWC27_HUMAN] | 3 | 1 | 3 | 472 | 53.8 | 5.8 |
| Q9BVP2 | Guanine nucleotide-binding protein-like 3 OS=Homo sapiens GN=GNL3 PE=1 SV=2 - [GNL3_HUMAN] | 3 | 1 | 3 | 549 | 62 | 9.16 |
| P49642 | DNA primase small subunit OS=Homo sapiens GN=PRIM1 PE=1 SV=1 - [PRI1_HUMAN] | 3 | 1 | 3 | 420 | 49.9 | 8.21 |
| Q9H3G5 | Probable serine carboxypeptidase CPVL OS=Homo sapiens GN=CPVL PE=1 SV=2 - [CPVL_HUMAN] | 3 | 1 | 3 | 476 | 54.1 | 5.62 |
| Q9NRY5 | Protein FAM114A2 OS=Homo sapiens GN=FAM114A2 PE=1 SV=4 - [F1142_HUMAN] | 3 | 1 | 3 | 505 | 55.4 | 4.88 |
| Q70UQ0 | Inhibitor of nuclear factor kappa-B kinase-interacting protein OS=Homo sapiens GN=IKIP PE=1 SV=1 - [IKIP_HUMAN] | 3 | 1 | 3 | 350 | 39.3 | 9.17 |
| O14773 | Tripeptidyl-peptidase 1 OS=Homo sapiens GN=TPP1 PE=1 SV=2 - [TPP1_HUMAN] | 3 | 1 | 3 | 563 | 61.2 | 6.48 |
| Q6NYC1 | Bifunctional arginine demethylase and lysyl-hydroxylase JMJD6 OS=Homo sapiens GN=JMJD6 PE=1 SV=1 - [JMJD6_HUMAN] | 3 | 1 | 3 | 403 | 46.4 | 8.69 |
| P49585 | Choline-phosphate cytidylyltransferase A OS=Homo sapiens GN=PCYT1A PE=1 SV=2 - [PCY1A_HUMAN] | 3 | 1 | 3 | 367 | 41.7 | 7.25 |
| Q8NBJ7 | Sulfatase-modifying factor 2 OS=Homo sapiens GN=SUMF2 PE=1 SV=2 - [SUMF2_HUMAN] | 3 | 1 | 3 | 301 | 33.8 | 8 |
| P61313 | 60S ribosomal protein L15 OS=Homo sapiens GN=RPL15 PE=1 SV=2 - [RL15_HUMAN] | 3 | 1 | 3 | 204 | 24.1 | 11.62 |
| Q9Y314 | Nitric oxide synthase-interacting protein OS=Homo sapiens GN=NOSIP PE=1 SV=1 - [NOSIP_HUMAN] | 3 | 1 | 3 | 301 | 33.2 | 8.82 |
| Q06203 | Amidophosphoribosyltransferase OS=Homo sapiens GN=PPAT PE=1 SV=1 - [PUR1_HUMAN] | 3 | 1 | 3 | 517 | 57.4 | 6.76 |
| P49406 | 39S ribosomal protein L19, mitochondrial OS=Homo sapiens GN=MRPL19 PE=1 SV=2 - [RM19_HUMAN] | 3 | 1 | 3 | 292 | 33.5 | 9.5 |
| P19404 | NADH dehydrogenase [ubiquinone] flavoprotein 2, mitochondrial OS=Homo sapiens GN=NDUFV2 PE=1 SV=2 - [NDUV2_HUMAN] | 3 | 1 | 3 | 249 | 27.4 | 8.06 |
| Q8WZA0 | Protein LZIC OS=Homo sapiens GN=LZIC PE=1 SV=1 - [LZIC_HUMAN] | 3 | 1 | 3 | 190 | 21.5 | 4.94 |
| P41227 | N-terminal acetyltransferase complex ARD1 subunit homolog A OS=Homo sapiens GN=ARD1A PE=1 SV=1 - [ARD1A_HUMAN] | 3 | 1 | 3 | 235 | 26.4 | 5.64 |
| Q9UHR5 | SAP30-binding protein OS=Homo sapiens GN=SAP30BP PE=1 SV=1 - [S30BP_HUMAN] | 3 | 1 | 3 | 308 | 33.8 | 4.84 |
| Q14847 | LIM and SH3 domain protein 1 OS=Homo sapiens GN=LASP1 PE=1 SV=2 - [LASP1_HUMAN] | 3 | 1 | 3 | 261 | 29.7 | 7.05 |
| P19387 | DNA-directed RNA polymerase II subunit RPB3 OS=Homo sapiens GN=POLR2C PE=1 SV=2 - [RPB3_HUMAN] | 3 | 1 | 3 | 275 | 31.4 | 4.92 |
| O95983 | Methyl-CpG-binding domain protein 3 OS=Homo sapiens GN=MBD3 PE=1 SV=1 - [MBD3_HUMAN] | 3 | 1 | 3 | 291 | 32.8 | 5.34 |
| Q9BZX2 | Uridine-cytidine kinase 2 OS=Homo sapiens GN=UCK2 PE=1 SV=1 - [UCK2_HUMAN] | 3 | 1 | 3 | 261 | 29.3 | 6.7 |
| O75608 | Acyl-protein thioesterase 1 OS=Homo sapiens GN=LYPLA1 PE=1 SV=1 - [LYPA1_HUMAN] | 3 | 1 | 3 | 230 | 24.7 | 6.77 |
| O75396 | Vesicle-trafficking protein SEC22b OS=Homo sapiens GN=SEC22B PE=1 SV=4 - [SC22B_HUMAN] | 3 | 1 | 3 | 215 | 24.6 | 6.92 |
| Q9UIJ7 | GTP:AMP phosphotransferase mitochondrial OS=Homo sapiens GN=AK3 PE=1 SV=4 - [KAD3_HUMAN] | 3 | 1 | 3 | 227 | 25.5 | 9.16 |
| Q9BRJ6 | Uncharacterized protein C7orf50 OS=Homo sapiens GN=C7orf50 PE=1 SV=1 - [CG050_HUMAN] | 3 | 1 | 3 | 194 | 22.1 | 9.64 |
| Q8WW12 | PEST proteolytic signal-containing nuclear protein OS=Homo sapiens GN=PCNP PE=1 SV=2 - [PCNP_HUMAN] | 3 | 1 | 3 | 178 | 18.9 | 7.49 |
| Q9BVG4 | UPF0368 protein Cxorf26 OS=Homo sapiens GN=CXorf26 PE=1 SV=1 - [CX026_HUMAN] | 3 | 1 | 3 | 233 | 26 | 4.79 |
| O60888 | Protein CutA OS=Homo sapiens GN=CUTA PE=1 SV=2 - [CUTA_HUMAN] | 3 | 1 | 3 | 179 | 19.1 | 5.5 |
| Q9BRA2 | Thioredoxin domain-containing protein 17 OS=Homo sapiens GN=TXNDC17 PE=1 SV=1 - [TXD17_HUMAN] | 3 | 1 | 3 | 123 | 13.9 | 5.52 |
| P52943 | Cysteine-rich protein 2 OS=Homo sapiens GN=CRIP2 PE=1 SV=1 - [CRIP2_HUMAN] | 3 | 1 | 3 | 208 | 22.5 | 8.72 |
| Q9H2U2 | Inorganic pyrophosphatase 2, mitochondrial OS=Homo sapiens GN=PPA2 PE=1 SV=2 - [IPYR2_HUMAN] | 3 | 2 | 5 | 334 | 37.9 | 7.39 |
| Q14166 | Tubulin--tyrosine ligase-like protein 12 OS=Homo sapiens GN=TTLL12 PE=1 SV=2 - [TTL12_HUMAN] | 3 | 2 | 5 | 644 | 74.4 | 5.53 |
| P11172 | Uridine 5'-monophosphate synthase OS=Homo sapiens GN=UMPS PE=1 SV=1 - [UMPS_HUMAN] | 3 | 2 | 5 | 480 | 52.2 | 7.24 |
| Q9NS69 | Mitochondrial import receptor subunit TOM22 homolog OS=Homo sapiens GN=TOMM22 PE=1 SV=3 - [TOM22_HUMAN] | 3 | 2 | 5 | 142 | 15.5 | 4.34 |
| P61204 | ADP-ribosylation factor 3 OS=Homo sapiens GN=ARF3 PE=1 SV=2 - [ARF3_HUMAN] | 3 | 3 | 6 | 181 | 20.6 | 7.43 |
| O00461 | Golgi integral membrane protein 4 OS=Homo sapiens GN=GOLIM4 PE=1 SV=1 - [GOLI4_HUMAN] | 3 | 2 | 4 | 696 | 81.8 | 4.77 |
| P36957 | Dihydrolipoyllysine-residue succinyltransferase component of 2-oxoglutarate dehydrogenase complex, mitochondrial OS=Homo sapiens GN=DLST PE=1 SV=4 - [ODO2_HUMAN] | 3 | 2 | 4 | 453 | 48.7 | 8.95 |
| P13473 | Lysosome-associated membrane glycoprotein 2 OS=Homo sapiens GN=LAMP2 PE=1 SV=2 - [LAMP2_HUMAN] | 3 | 2 | 4 | 410 | 44.9 | 5.63 |
| P14324 | Farnesyl pyrophosphate synthase OS=Homo sapiens GN=FDPS PE=1 SV=4 - [FPPS_HUMAN] | 3 | 2 | 4 | 419 | 48.2 | 6.15 |
| Q9UI10 | Translation initiation factor eIF-2B subunit delta OS=Homo sapiens GN=EIF2B4 PE=1 SV=2 - [EI2BD_HUMAN] | 3 | 2 | 4 | 523 | 57.5 | 9.38 |
| Q15427 | Splicing factor 3B subunit 4 OS=Homo sapiens GN=SF3B4 PE=1 SV=1 - [SF3B4_HUMAN] | 3 | 2 | 4 | 424 | 44.4 | 8.56 |
| Q9H9Q2 | COP9 signalosome complex subunit 7b OS=Homo sapiens GN=COPS7B PE=1 SV=1 - [CSN7B_HUMAN] | 3 | 2 | 4 | 264 | 29.6 | 6.15 |
| O15305 | Phosphomannomutase 2 OS=Homo sapiens GN=PMM2 PE=1 SV=1 - [PMM2_HUMAN] | 3 | 2 | 4 | 246 | 28.1 | 6.77 |
| P30042 | ES1 protein homolog, mitochondrial OS=Homo sapiens GN=C21orf33 PE=1 SV=3 - [ES1_HUMAN] | 3 | 2 | 4 | 268 | 28.2 | 8.27 |
| O15347 | High mobility group protein B3 OS=Homo sapiens GN=HMGB3 PE=1 SV=4 - [HMGB3_HUMAN] | 3 | 2 | 4 | 200 | 23 | 8.37 |
| Q9BRP8 | Partner of Y14 and mago OS=Homo sapiens GN=PYM1 PE=1 SV=1 - [PYM1_HUMAN] | 3 | 2 | 4 | 204 | 22.6 | 9.45 |
| Q99471 | Prefoldin subunit 5 OS=Homo sapiens GN=PFDN5 PE=1 SV=2 - [PFD5_HUMAN] | 3 | 2 | 4 | 154 | 17.3 | 6.33 |
| P52948 | Nuclear pore complex protein Nup98-Nup96 OS=Homo sapiens GN=NUP98 PE=1 SV=4 - [NUP98_HUMAN] | 3 | 1 | 2 | 1817 | 197.5 | 6.4 |
| P46013 | Antigen KI-67 OS=Homo sapiens GN=MKI67 PE=1 SV=2 - [KI67_HUMAN] | 3 | 1 | 2 | 3256 | 358.5 | 9.45 |
| O60264 | SWI/SNF-related matrix-associated actin-dependent regulator of chromatin subfamily A member 5 OS=Homo sapiens GN=SMARCA5 PE=1 SV=1 - [SMCA5_HUMAN] | 3 | 1 | 2 | 1052 | 121.8 | 8.09 |
| Q92896 | Golgi apparatus protein 1 OS=Homo sapiens GN=GLG1 PE=1 SV=2 - [GSLG1_HUMAN] | 3 | 1 | 2 | 1179 | 134.5 | 6.9 |
| Q9ULW0 | Targeting protein for Xklp2 OS=Homo sapiens GN=TPX2 PE=1 SV=2 - [TPX2_HUMAN] | 3 | 1 | 2 | 747 | 85.6 | 9.23 |
| Q10713 | Mitochondrial-processing peptidase subunit alpha OS=Homo sapiens GN=PMPCA PE=1 SV=2 - [MPPA_HUMAN] | 3 | 1 | 2 | 525 | 58.2 | 6.92 |
| Q9BPX3 | Condensin complex subunit 3 OS=Homo sapiens GN=NCAPG PE=1 SV=1 - [CND3_HUMAN] | 3 | 1 | 2 | 1015 | 114.3 | 5.59 |
| Q9Y6D9 | Mitotic spindle assembly checkpoint protein MAD1 OS=Homo sapiens GN=MAD1L1 PE=1 SV=2 - [MD1L1_HUMAN] | 3 | 1 | 2 | 718 | 83 | 5.92 |
| Q14671 | Pumilio homolog 1 OS=Homo sapiens GN=PUM1 PE=1 SV=3 - [PUM1_HUMAN] | 3 | 1 | 2 | 1186 | 126.4 | 6.84 |
| Q96QC0 | Serine/threonine-protein phosphatase 1 regulatory subunit 10 OS=Homo sapiens GN=PPP1R10 PE=1 SV=1 - [PP1RA_HUMAN] | 3 | 1 | 2 | 940 | 99 | 9.17 |
| Q9BTW9 | Tubulin-specific chaperone D OS=Homo sapiens GN=TBCD PE=1 SV=2 - [TBCD_HUMAN] | 3 | 1 | 2 | 1192 | 132.5 | 6.19 |
| P07948 | Tyrosine-protein kinase Lyn OS=Homo sapiens GN=LYN PE=1 SV=3 - [LYN_HUMAN] | 3 | 1 | 2 | 512 | 58.5 | 7.11 |
| P50552 | Vasodilator-stimulated phosphoprotein OS=Homo sapiens GN=VASP PE=1 SV=3 - [VASP_HUMAN] | 3 | 1 | 2 | 380 | 39.8 | 8.94 |
| O75312 | Zinc finger protein ZPR1 OS=Homo sapiens GN=ZNF259 PE=1 SV=1 - [ZPR1_HUMAN] | 3 | 1 | 2 | 459 | 50.9 | 4.73 |
| Q8NFF5 | FAD synthetase OS=Homo sapiens GN=FLAD1 PE=1 SV=1 - [FAD1_HUMAN] | 3 | 1 | 2 | 587 | 65.2 | 6.93 |
| P51911 | Calponin-1 OS=Homo sapiens GN=CNN1 PE=1 SV=2 - [CNN1_HUMAN] | 3 | 1 | 2 | 297 | 33.1 | 9.07 |
| Q9UJ70 | N-acetyl-D-glucosamine kinase OS=Homo sapiens GN=NAGK PE=1 SV=4 - [NAGK_HUMAN] | 3 | 1 | 2 | 344 | 37.4 | 6.24 |
| P15374 | Ubiquitin carboxyl-terminal hydrolase isozyme L3 OS=Homo sapiens GN=UCHL3 PE=1 SV=1 - [UCHL3_HUMAN] | 3 | 1 | 2 | 230 | 26.2 | 4.92 |
| Q9UBQ7 | Glyoxylate reductase/hydroxypyruvate reductase OS=Homo sapiens GN=GRHPR PE=1 SV=1 - [GRHPR_HUMAN] | 3 | 1 | 2 | 328 | 35.6 | 7.39 |
| Q9NVZ3 | Adaptin ear-binding coat-associated protein 2 OS=Homo sapiens GN=NECAP2 PE=1 SV=1 - [NECP2_HUMAN] | 3 | 1 | 2 | 263 | 28.3 | 8.38 |
| Q9Y383 | Putative RNA-binding protein Luc7-like 2 OS=Homo sapiens GN=LUC7L2 PE=1 SV=2 - [LC7L2_HUMAN] | 3 | 3 | 5 | 392 | 46.5 | 10.01 |
| Q9NQ29 | Putative RNA-binding protein Luc7-like 1 OS=Homo sapiens GN=LUC7L PE=1 SV=1 - [LUC7L_HUMAN] | 3 | 3 | 5 | 371 | 43.7 | 9.92 |
| Q9P0L0 | Vesicle-associated membrane protein-associated protein A OS=Homo sapiens GN=VAPA PE=1 SV=3 - [VAPA_HUMAN] | 3 | 3 | 5 | 249 | 27.9 | 8.62 |
| P84103 | Splicing factor, arginine/serine-rich 3 OS=Homo sapiens GN=SFRS3 PE=1 SV=1 - [SFRS3_HUMAN] | 3 | 3 | 5 | 164 | 19.3 | 11.65 |
| P05386 | 60S acidic ribosomal protein P1 OS=Homo sapiens GN=RPLP1 PE=1 SV=1 - [RLA1_HUMAN] | 3 | 3 | 5 | 114 | 11.5 | 4.32 |
| Q10567 | AP-1 complex subunit beta-1 OS=Homo sapiens GN=AP1B1 PE=1 SV=2 - [AP1B1_HUMAN] | 3 | 5 | 8 | 949 | 104.6 | 5.06 |
| Q9Y281 | Cofilin-2 OS=Homo sapiens GN=CFL2 PE=1 SV=1 - [COF2_HUMAN] | 3 | 5 | 8 | 166 | 18.7 | 7.88 |
| P22392 | Nucleoside diphosphate kinase B OS=Homo sapiens GN=NME2 PE=1 SV=1 - [NDKB_HUMAN] | 3 | 9 | 14 | 152 | 17.3 | 8.41 |
| O95292 | Vesicle-associated membrane protein-associated protein B/C OS=Homo sapiens GN=VAPB PE=1 SV=3 - [VAPB_HUMAN] | 3 | 4 | 6 | 243 | 27.2 | 7.3 |
| Q15393 | Splicing factor 3B subunit 3 OS=Homo sapiens GN=SF3B3 PE=1 SV=4 - [SF3B3_HUMAN] | 3 | 2 | 3 | 1217 | 135.5 | 5.26 |
| Q15020 | Squamous cell carcinoma antigen recognized by T-cells 3 OS=Homo sapiens GN=SART3 PE=1 SV=1 - [SART3_HUMAN] | 3 | 2 | 3 | 963 | 109.9 | 5.57 |
| Q9NYF8 | Bcl-2-associated transcription factor 1 OS=Homo sapiens GN=BCLAF1 PE=1 SV=2 - [BCLF1_HUMAN] | 3 | 2 | 3 | 920 | 106.1 | 9.98 |
| Q6Y7W6 | PERQ amino acid-rich with GYF domain-containing protein 2 OS=Homo sapiens GN=GIGYF2 PE=1 SV=1 - [PERQ2_HUMAN] | 3 | 2 | 3 | 1299 | 150 | 5.54 |
| O94842 | TOX high mobility group box family member 4 OS=Homo sapiens GN=TOX4 PE=1 SV=1 - [TOX4_HUMAN] | 3 | 2 | 3 | 621 | 66.2 | 5.06 |
| Q96JM3 | Zinc finger protein 828 OS=Homo sapiens GN=ZNF828 PE=1 SV=2 - [ZN828_HUMAN] | 3 | 2 | 3 | 812 | 89 | 8.44 |
| O43464 | Serine protease HTRA2, mitochondrial OS=Homo sapiens GN=HTRA2 PE=1 SV=2 - [HTRA2_HUMAN] | 3 | 2 | 3 | 458 | 48.8 | 10.07 |
| Q8IXH7 | Negative elongation factor C/D OS=Homo sapiens GN=TH1L PE=1 SV=2 - [NELFD_HUMAN] | 3 | 2 | 3 | 590 | 66.2 | 5.1 |
| Q9Y5A9 | YTH domain family protein 2 OS=Homo sapiens GN=YTHDF2 PE=1 SV=2 - [YTHD2_HUMAN] | 3 | 2 | 3 | 579 | 62.3 | 8.79 |
| P47985 | Cytochrome b-c1 complex subunit Rieske, mitochondrial OS=Homo sapiens GN=UQCRFS1 PE=1 SV=2 - [UCRI_HUMAN] | 3 | 2 | 3 | 274 | 29.6 | 8.32 |
| O75718 | Cartilage-associated protein OS=Homo sapiens GN=CRTAP PE=1 SV=1 - [CRTAP_HUMAN] | 3 | 2 | 3 | 401 | 46.5 | 5.73 |
| Q9HB07 | UPF0160 protein MYG1, mitochondrial OS=Homo sapiens GN=C12orf10 PE=1 SV=2 - [MYG1_HUMAN] | 3 | 2 | 3 | 376 | 42.4 | 6.67 |
| Q6NXE6 | Armadillo repeat-containing protein 6 OS=Homo sapiens GN=ARMC6 PE=1 SV=2 - [ARMC6_HUMAN] | 3 | 2 | 3 | 501 | 54.1 | 6.24 |
| Q9BVI4 | Nucleolar complex protein 4 homolog OS=Homo sapiens GN=NOC4L PE=1 SV=1 - [NOC4L_HUMAN] | 3 | 2 | 3 | 516 | 58.4 | 7.49 |
| Q9NY61 | Protein AATF OS=Homo sapiens GN=AATF PE=1 SV=1 - [AATF_HUMAN] | 3 | 2 | 3 | 560 | 63.1 | 4.94 |
| Q13505 | Metaxin-1 OS=Homo sapiens GN=MTX1 PE=1 SV=2 - [MTX1_HUMAN] | 3 | 2 | 3 | 466 | 51.4 | 9.79 |
| Q96M27 | Protein PRRC1 OS=Homo sapiens GN=PRRC1 PE=1 SV=1 - [PRRC1_HUMAN] | 3 | 2 | 3 | 445 | 46.7 | 5.83 |
| Q1ED39 | Protein C16orf88 OS=Homo sapiens GN=C16orf88 PE=1 SV=1 - [CP088_HUMAN] | 3 | 2 | 3 | 458 | 51.6 | 9.86 |
| P53597 | Succinate--CoA ligase [ADP/GDP-forming] subunit alpha, mitochondrial OS=Homo sapiens GN=SUCLG1 PE=1 SV=4 - [SUCA_HUMAN] | 3 | 2 | 3 | 346 | 36.2 | 8.79 |
| P00403 | Cytochrome c oxidase subunit 2 OS=Homo sapiens GN=MT-CO2 PE=1 SV=1 - [COX2_HUMAN] | 3 | 2 | 3 | 227 | 25.5 | 4.82 |
| Q9BTD8 | RNA-binding protein 42 OS=Homo sapiens GN=RBM42 PE=1 SV=1 - [RBM42_HUMAN] | 3 | 2 | 3 | 480 | 50.4 | 9.63 |
| Q92785 | Zinc finger protein ubi-d4 OS=Homo sapiens GN=DPF2 PE=1 SV=2 - [REQU_HUMAN] | 3 | 2 | 3 | 391 | 44.1 | 6.33 |
| P35237 | Serpin B6 OS=Homo sapiens GN=SERPINB6 PE=1 SV=3 - [SPB6_HUMAN] | 3 | 2 | 3 | 376 | 42.6 | 5.27 |
| P26373 | 60S ribosomal protein L13 OS=Homo sapiens GN=RPL13 PE=1 SV=4 - [RL13_HUMAN] | 3 | 2 | 3 | 211 | 24.2 | 11.65 |
| P21912 | Succinate dehydrogenase [ubiquinone] iron-sulfur subunit, mitochondrial OS=Homo sapiens GN=SDHB PE=1 SV=3 - [DHSB_HUMAN] | 3 | 2 | 3 | 280 | 31.6 | 8.76 |
| P84098 | 60S ribosomal protein L19 OS=Homo sapiens GN=RPL19 PE=1 SV=1 - [RL19_HUMAN] | 3 | 2 | 3 | 196 | 23.5 | 11.47 |
| Q9H444 | Charged multivesicular body protein 4b OS=Homo sapiens GN=CHMP4B PE=1 SV=1 - [CHM4B_HUMAN] | 3 | 2 | 3 | 224 | 24.9 | 4.82 |
| P62280 | 40S ribosomal protein S11 OS=Homo sapiens GN=RPS11 PE=1 SV=3 - [RS11_HUMAN] | 3 | 2 | 3 | 158 | 18.4 | 10.3 |
| P62753 | 40S ribosomal protein S6 OS=Homo sapiens GN=RPS6 PE=1 SV=1 - [RS6_HUMAN] | 3 | 2 | 3 | 249 | 28.7 | 10.84 |
| P68402 | Platelet-activating factor acetylhydrolase IB subunit beta OS=Homo sapiens GN=PAFAH1B2 PE=1 SV=1 - [PA1B2_HUMAN] | 3 | 2 | 3 | 229 | 25.6 | 5.92 |
| Q8NCW5 | Apolipoprotein A-I-binding protein OS=Homo sapiens GN=APOA1BP PE=1 SV=2 - [AIBP_HUMAN] | 3 | 2 | 3 | 288 | 31.7 | 7.66 |
| Q9NRV9 | Heme-binding protein 1 OS=Homo sapiens GN=HEBP1 PE=1 SV=1 - [HEBP1_HUMAN] | 3 | 2 | 3 | 189 | 21.1 | 5.8 |
| P42771 | Cyclin-dependent kinase inhibitor 2A, isoforms 1/2/3 OS=Homo sapiens GN=CDKN2A PE=1 SV=2 - [CD2A1_HUMAN] | 3 | 2 | 3 | 156 | 16.5 | 5.81 |
| Q8N5K1 | CDGSH iron sulfur domain-containing protein 2 OS=Homo sapiens GN=CISD2 PE=1 SV=1 - [CISD2_HUMAN] | 3 | 2 | 3 | 135 | 15.3 | 9.61 |
| P09132 | Signal recognition particle 19 kDa protein OS=Homo sapiens GN=SRP19 PE=1 SV=3 - [SRP19_HUMAN] | 3 | 2 | 3 | 144 | 16.1 | 9.85 |
| Q15424 | Scaffold attachment factor B1 OS=Homo sapiens GN=SAFB PE=1 SV=4 - [SAFB1_HUMAN] | 3 | 5 | 7 | 915 | 102.6 | 5.47 |
| Q9H0U4 | Ras-related protein Rab-1B OS=Homo sapiens GN=RAB1B PE=1 SV=1 - [RAB1B_HUMAN] | 3 | 5 | 7 | 201 | 22.2 | 5.73 |
| O14787 | Transportin-2 OS=Homo sapiens GN=TNPO2 PE=1 SV=3 - [TNPO2_HUMAN] | 3 | 3 | 4 | 897 | 101.3 | 5.01 |
| Q8N8S7 | Protein enabled homolog OS=Homo sapiens GN=ENAH PE=1 SV=2 - [ENAH_HUMAN] | 3 | 3 | 4 | 591 | 66.5 | 6.93 |
| Q8IYB3 | Serine/arginine repetitive matrix protein 1 OS=Homo sapiens GN=SRRM1 PE=1 SV=2 - [SRRM1_HUMAN] | 3 | 3 | 4 | 904 | 102.3 | 11.84 |
| Q9Y371 | Endophilin-B1 OS=Homo sapiens GN=SH3GLB1 PE=1 SV=1 - [SHLB1_HUMAN] | 3 | 3 | 4 | 365 | 40.8 | 6.04 |
| P61758 | Prefoldin subunit 3 OS=Homo sapiens GN=VBP1 PE=1 SV=3 - [PFD3_HUMAN] | 3 | 3 | 4 | 197 | 22.6 | 7.11 |
| Q01130 | Splicing factor, arginine/serine-rich 2 OS=Homo sapiens GN=SFRS2 PE=1 SV=4 - [SFRS2_HUMAN] | 3 | 3 | 4 | 221 | 25.5 | 11.85 |
| P60866 | 40S ribosomal protein S20 OS=Homo sapiens GN=RPS20 PE=1 SV=1 - [RS20_HUMAN] | 3 | 3 | 4 | 119 | 13.4 | 9.94 |
| Q9BV86 | Methyltransferase-like protein 11A OS=Homo sapiens GN=METTL11A PE=1 SV=3 - [ME11A_HUMAN] | 3 | 3 | 4 | 223 | 25.4 | 5.52 |
| Q9UHV9 | Prefoldin subunit 2 OS=Homo sapiens GN=PFDN2 PE=1 SV=1 - [PFD2_HUMAN] | 3 | 3 | 4 | 154 | 16.6 | 6.58 |
| P30085 | UMP-CMP kinase OS=Homo sapiens GN=CMPK1 PE=1 SV=3 - [KCY_HUMAN] | 3 | 3 | 4 | 196 | 22.2 | 5.57 |
| P61106 | Ras-related protein Rab-14 OS=Homo sapiens GN=RAB14 PE=1 SV=4 - [RAB14_HUMAN] | 3 | 3 | 4 | 215 | 23.9 | 6.21 |
| P62847 | 40S ribosomal protein S24 OS=Homo sapiens GN=RPS24 PE=1 SV=1 - [RS24_HUMAN] | 3 | 3 | 4 | 133 | 15.4 | 10.78 |
| P62158 | Calmodulin OS=Homo sapiens GN=CALM1 PE=1 SV=2 - [CALM_HUMAN] | 3 | 3 | 4 | 149 | 16.8 | 4.22 |
| Q9UN86 | Ras GTPase-activating protein-binding protein 2 OS=Homo sapiens GN=G3BP2 PE=1 SV=2 - [G3BP2_HUMAN] | 3 | 4 | 5 | 482 | 54.1 | 5.55 |
| P42167 | Lamina-associated polypeptide 2, isoforms beta/gamma OS=Homo sapiens GN=TMPO PE=1 SV=2 - [LAP2B_HUMAN] | 3 | 9 | 11 | 454 | 50.6 | 9.38 |
| P15531 | Nucleoside diphosphate kinase A OS=Homo sapiens GN=NME1 PE=1 SV=1 - [NDKA_HUMAN] | 3 | 12 | 14 | 152 | 17.1 | 6.19 |
| P31150 | Rab GDP dissociation inhibitor alpha OS=Homo sapiens GN=GDI1 PE=1 SV=2 - [GDIA_HUMAN] | 3 | 7 | 8 | 447 | 50.6 | 5.14 |
| Q92599 | Septin-8 OS=Homo sapiens GN=SEPT8 PE=1 SV=4 - [SEPT8_HUMAN] | 3 | 5 | 5 | 483 | 55.7 | 6.28 |
| P60953 | Cell division control protein 42 homolog OS=Homo sapiens GN=CDC42 PE=1 SV=2 - [CDC42_HUMAN] | 3 | 5 | 5 | 191 | 21.2 | 6.55 |
| Q68EM7 | Rho GTPase-activating protein 17 OS=Homo sapiens GN=ARHGAP17 PE=1 SV=1 - [RHG17_HUMAN] | 3 | 4 | 4 | 881 | 95.4 | 7.62 |
| Q9UJS0 | Calcium-binding mitochondrial carrier protein Aralar2 OS=Homo sapiens GN=SLC25A13 PE=1 SV=2 - [CMC2_HUMAN] | 3 | 4 | 4 | 675 | 74.1 | 8.62 |
| Q15738 | Sterol-4-alpha-carboxylate 3-dehydrogenase, decarboxylating OS=Homo sapiens GN=NSDHL PE=1 SV=2 - [NSDHL_HUMAN] | 3 | 4 | 4 | 373 | 41.9 | 8.06 |
| Q96GG9 | DCN1-like protein 1 OS=Homo sapiens GN=DCUN1D1 PE=1 SV=1 - [DCNL1_HUMAN] | 3 | 4 | 4 | 259 | 30.1 | 5.34 |
| P21964 | Catechol O-methyltransferase OS=Homo sapiens GN=COMT PE=1 SV=2 - [COMT_HUMAN] | 3 | 4 | 4 | 271 | 30 | 5.47 |
| P60981 | Destrin OS=Homo sapiens GN=DSTN PE=1 SV=3 - [DEST_HUMAN] | 3 | 4 | 4 | 165 | 18.5 | 7.85 |
| P37108 | Signal recognition particle 14 kDa protein OS=Homo sapiens GN=SRP14 PE=1 SV=2 - [SRP14_HUMAN] | 3 | 4 | 4 | 136 | 14.6 | 10.04 |
| P11279 | Lysosome-associated membrane glycoprotein 1 OS=Homo sapiens GN=LAMP1 PE=1 SV=3 - [LAMP1_HUMAN] | 3 | 3 | 3 | 417 | 44.9 | 8.75 |
| Q49AR2 | UPF0489 protein C5orf22 OS=Homo sapiens GN=C5orf22 PE=1 SV=2 - [CE022_HUMAN] | 3 | 3 | 3 | 442 | 49.9 | 4.78 |
| Q9NUJ1 | Mycophenolic acid acyl-glucuronide esterase, mitochondrial OS=Homo sapiens GN=ABHD10 PE=1 SV=1 - [ABHDA_HUMAN] | 3 | 3 | 3 | 306 | 33.9 | 8.57 |
| P51572 | B-cell receptor-associated protein 31 OS=Homo sapiens GN=BCAP31 PE=1 SV=3 - [BAP31_HUMAN] | 3 | 3 | 3 | 246 | 28 | 8.44 |
| O00625 | Pirin OS=Homo sapiens GN=PIR PE=1 SV=1 - [PIR_HUMAN] | 3 | 3 | 3 | 290 | 32.1 | 6.92 |
| P28676 | Grancalcin OS=Homo sapiens GN=GCA PE=1 SV=2 - [GRAN_HUMAN] | 3 | 3 | 3 | 217 | 24 | 5.21 |
| Q96P16 | Regulation of nuclear pre-mRNA domain-containing protein 1A OS=Homo sapiens GN=RPRD1A PE=1 SV=1 - [RPR1A_HUMAN] | 3 | 3 | 3 | 312 | 35.7 | 7.55 |
| Q96CT7 | Coiled-coil domain-containing protein 124 OS=Homo sapiens GN=CCDC124 PE=1 SV=1 - [CC124_HUMAN] | 3 | 3 | 3 | 223 | 25.8 | 9.54 |
| P48739 | Phosphatidylinositol transfer protein beta isoform OS=Homo sapiens GN=PITPNB PE=1 SV=2 - [PIPNB_HUMAN] | 3 | 3 | 3 | 271 | 31.5 | 6.87 |
| P67870 | Casein kinase II subunit beta OS=Homo sapiens GN=CSNK2B PE=1 SV=1 - [CSK2B_HUMAN] | 3 | 3 | 3 | 215 | 24.9 | 5.55 |
| O96000 | NADH dehydrogenase [ubiquinone] 1 beta subcomplex subunit 10 OS=Homo sapiens GN=NDUFB10 PE=1 SV=3 - [NDUBA_HUMAN] | 3 | 3 | 3 | 172 | 20.8 | 8.48 |
| O60869 | Endothelial differentiation-related factor 1 OS=Homo sapiens GN=EDF1 PE=1 SV=1 - [EDF1_HUMAN] | 3 | 3 | 3 | 148 | 16.4 | 9.95 |
| P62318 | Small nuclear ribonucleoprotein Sm D3 OS=Homo sapiens GN=SNRPD3 PE=1 SV=1 - [SMD3_HUMAN] | 3 | 3 | 3 | 126 | 13.9 | 10.32 |
| P63165 | Small ubiquitin-related modifier 1 OS=Homo sapiens GN=SUMO1 PE=1 SV=1 - [SUMO1_HUMAN] | 3 | 3 | 3 | 101 | 11.5 | 5.52 |
| Q8NI27 | THO complex subunit 2 OS=Homo sapiens GN=THOC2 PE=1 SV=2 - [THOC2_HUMAN] | 3 | 2 | 2 | 1593 | 182.7 | 8.44 |
| Q08AD1 | Calmodulin-regulated spectrin-associated protein 2 OS=Homo sapiens GN=CAMSAP2 PE=1 SV=3 - [CAMP2_HUMAN] | 3 | 2 | 2 | 1489 | 168 | 6.8 |
| O95239 | Chromosome-associated kinesin KIF4A OS=Homo sapiens GN=KIF4A PE=1 SV=3 - [KIF4A_HUMAN] | 3 | 2 | 2 | 1232 | 139.8 | 6.27 |
| Q53GS9 | U4/U6.U5 tri-snRNP-associated protein 2 OS=Homo sapiens GN=USP39 PE=1 SV=2 - [SNUT2_HUMAN] | 3 | 2 | 2 | 565 | 65.3 | 8.91 |
| Q9P258 | Protein RCC2 OS=Homo sapiens GN=RCC2 PE=1 SV=2 - [RCC2_HUMAN] | 3 | 2 | 2 | 522 | 56 | 8.78 |
| Q09161 | Nuclear cap-binding protein subunit 1 OS=Homo sapiens GN=NCBP1 PE=1 SV=1 - [NCBP1_HUMAN] | 3 | 2 | 2 | 790 | 91.8 | 6.43 |
| Q7Z2W4 | Zinc finger CCCH-type antiviral protein 1 OS=Homo sapiens GN=ZC3HAV1 PE=1 SV=3 - [ZCCHV_HUMAN] | 3 | 2 | 2 | 902 | 101.4 | 8.4 |
| P50995 | Annexin A11 OS=Homo sapiens GN=ANXA11 PE=1 SV=1 - [ANX11_HUMAN] | 3 | 2 | 2 | 505 | 54.4 | 7.65 |
| Q5BKZ1 | DBIRD complex subunit ZNF326 OS=Homo sapiens GN=ZNF326 PE=1 SV=2 - [ZN326_HUMAN] | 3 | 2 | 2 | 582 | 65.6 | 5.15 |
| Q0VDF9 | Heat shock 70 kDa protein 14 OS=Homo sapiens GN=HSPA14 PE=1 SV=1 - [HSP7E_HUMAN] | 3 | 2 | 2 | 509 | 54.8 | 5.59 |
| P39023 | 60S ribosomal protein L3 OS=Homo sapiens GN=RPL3 PE=1 SV=2 - [RL3_HUMAN] | 3 | 2 | 2 | 403 | 46.1 | 10.18 |
| Q92734 | Protein TFG OS=Homo sapiens GN=TFG PE=1 SV=2 - [TFG_HUMAN] | 3 | 2 | 2 | 400 | 43.4 | 5.1 |
| Q16775 | Hydroxyacylglutathione hydrolase, mitochondrial OS=Homo sapiens GN=HAGH PE=1 SV=2 - [GLO2_HUMAN] | 3 | 2 | 2 | 308 | 33.8 | 8.12 |
| P28070 | Proteasome subunit beta type-4 OS=Homo sapiens GN=PSMB4 PE=1 SV=4 - [PSB4_HUMAN] | 3 | 2 | 2 | 264 | 29.2 | 5.97 |
| O43488 | Aflatoxin B1 aldehyde reductase member 2 OS=Homo sapiens GN=AKR7A2 PE=1 SV=3 - [ARK72_HUMAN] | 3 | 2 | 2 | 359 | 39.6 | 7.17 |
| Q9NYK5 | 39S ribosomal protein L39, mitochondrial OS=Homo sapiens GN=MRPL39 PE=1 SV=3 - [RM39_HUMAN] | 3 | 2 | 2 | 338 | 38.7 | 7.65 |
| P53582 | Methionine aminopeptidase 1 OS=Homo sapiens GN=METAP1 PE=1 SV=2 - [AMPM1_HUMAN] | 3 | 2 | 2 | 386 | 43.2 | 7.17 |
| Q9Y512 | Sorting and assembly machinery component 50 homolog OS=Homo sapiens GN=SAMM50 PE=1 SV=3 - [SAM50_HUMAN] | 3 | 2 | 2 | 469 | 51.9 | 6.9 |
| P15927 | Replication protein A 32 kDa subunit OS=Homo sapiens GN=RPA2 PE=1 SV=1 - [RFA2_HUMAN] | 3 | 2 | 2 | 270 | 29.2 | 6.15 |
| O95816 | BAG family molecular chaperone regulator 2 OS=Homo sapiens GN=BAG2 PE=1 SV=1 - [BAG2_HUMAN] | 3 | 2 | 2 | 211 | 23.8 | 6.7 |
| P09417 | Dihydropteridine reductase OS=Homo sapiens GN=QDPR PE=1 SV=2 - [DHPR_HUMAN] | 3 | 2 | 2 | 244 | 25.8 | 7.37 |
| O95861 | 3'(2'),5'-bisphosphate nucleotidase 1 OS=Homo sapiens GN=BPNT1 PE=1 SV=1 - [BPNT1_HUMAN] | 3 | 2 | 2 | 308 | 33.4 | 5.69 |
| O00764 | Pyridoxal kinase OS=Homo sapiens GN=PDXK PE=1 SV=1 - [PDXK_HUMAN] | 3 | 2 | 2 | 312 | 35.1 | 6.13 |
| Q9Y3D6 | Mitochondrial fission 1 protein OS=Homo sapiens GN=FIS1 PE=1 SV=2 - [FIS1_HUMAN] | 3 | 2 | 2 | 152 | 16.9 | 8.79 |
| P62263 | 40S ribosomal protein S14 OS=Homo sapiens GN=RPS14 PE=1 SV=3 - [RS14_HUMAN] | 3 | 2 | 2 | 151 | 16.3 | 10.05 |
| Q9Y2B0 | Protein canopy homolog 2 OS=Homo sapiens GN=CNPY2 PE=1 SV=1 - [CNPY2_HUMAN] | 3 | 2 | 2 | 182 | 20.6 | 4.92 |
| P30046 | D-dopachrome decarboxylase OS=Homo sapiens GN=DDT PE=1 SV=3 - [DOPD_HUMAN] | 3 | 2 | 2 | 118 | 12.7 | 7.3 |
| P04080 | Cystatin-B OS=Homo sapiens GN=CSTB PE=1 SV=2 - [CYTB_HUMAN] | 3 | 2 | 2 | 98 | 11.1 | 7.56 |
| Q9UKY7 | Protein CDV3 homolog OS=Homo sapiens GN=CDV3 PE=1 SV=1 - [CDV3_HUMAN] | 3 | 2 | 2 | 258 | 27.3 | 6.4 |
| P60842 | Eukaryotic initiation factor 4A-I OS=Homo sapiens GN=EIF4A1 PE=1 SV=1 - [IF4A1_HUMAN] | 3 | 9 | 8 | 406 | 46.1 | 5.48 |
| Q05639 | Elongation factor 1-alpha 2 OS=Homo sapiens GN=EEF1A2 PE=1 SV=1 - [EF1A2_HUMAN] | 3 | 12 | 9 | 463 | 50.4 | 9.03 |
| P62888 | 60S ribosomal protein L30 OS=Homo sapiens GN=RPL30 PE=1 SV=2 - [RL30_HUMAN] | 3 | 4 | 3 | 115 | 12.8 | 9.63 |
| P63220 | 40S ribosomal protein S21 OS=Homo sapiens GN=RPS21 PE=1 SV=1 - [RS21_HUMAN] | 3 | 4 | 3 | 83 | 9.1 | 8.5 |
| Q14240 | Eukaryotic initiation factor 4A-II OS=Homo sapiens GN=EIF4A2 PE=1 SV=2 - [IF4A2_HUMAN] | 3 | 10 | 7 | 407 | 46.4 | 5.48 |
| P18583 | Protein SON OS=Homo sapiens GN=SON PE=1 SV=4 - [SON_HUMAN] | 3 | 3 | 2 | 2426 | 263.7 | 5.64 |
| O60716 | Catenin delta-1 OS=Homo sapiens GN=CTNND1 PE=1 SV=1 - [CTND1_HUMAN] | 3 | 3 | 2 | 968 | 108.1 | 6.23 |
| Q32P28 | Prolyl 3-hydroxylase 1 OS=Homo sapiens GN=LEPRE1 PE=1 SV=2 - [P3H1_HUMAN] | 3 | 3 | 2 | 736 | 83.3 | 5.14 |
| O43719 | HIV Tat-specific factor 1 OS=Homo sapiens GN=HTATSF1 PE=1 SV=1 - [HTSF1_HUMAN] | 3 | 3 | 2 | 755 | 85.8 | 4.4 |
| Q92769 | Histone deacetylase 2 OS=Homo sapiens GN=HDAC2 PE=1 SV=2 - [HDAC2_HUMAN] | 3 | 3 | 2 | 488 | 55.3 | 5.91 |
| Q96I99 | Succinyl-CoA ligase [GDP-forming] subunit beta, mitochondrial OS=Homo sapiens GN=SUCLG2 PE=1 SV=2 - [SUCB2_HUMAN] | 3 | 3 | 2 | 432 | 46.5 | 6.39 |
| Q9H3P2 | Negative elongation factor A OS=Homo sapiens GN=WHSC2 PE=1 SV=3 - [NELFA_HUMAN] | 3 | 3 | 2 | 528 | 57.2 | 9.03 |
| Q8NFH3 | Nucleoporin Nup43 OS=Homo sapiens GN=NUP43 PE=1 SV=1 - [NUP43_HUMAN] | 3 | 3 | 2 | 380 | 42.1 | 5.63 |
| Q99496 | E3 ubiquitin-protein ligase RING2 OS=Homo sapiens GN=RNF2 PE=1 SV=1 - [RING2_HUMAN] | 3 | 3 | 2 | 336 | 37.6 | 6.84 |
| Q8WXX5 | DnaJ homolog subfamily C member 9 OS=Homo sapiens GN=DNAJC9 PE=1 SV=1 - [DNJC9_HUMAN] | 3 | 3 | 2 | 260 | 29.9 | 5.73 |
| Q9H9H4 | Vacuolar protein sorting-associated protein 37B OS=Homo sapiens GN=VPS37B PE=1 SV=1 - [VP37B_HUMAN] | 3 | 3 | 2 | 285 | 31.3 | 7.34 |
| P61353 | 60S ribosomal protein L27 OS=Homo sapiens GN=RPL27 PE=1 SV=2 - [RL27_HUMAN] | 3 | 3 | 2 | 136 | 15.8 | 10.56 |
| P19367 | Hexokinase-1 OS=Homo sapiens GN=HK1 PE=1 SV=3 - [HXK1_HUMAN] | 3 | 4 | 2 | 917 | 102.4 | 6.8 |
| Q9P2E9 | Ribosome-binding protein 1 OS=Homo sapiens GN=RRBP1 PE=1 SV=4 - [RRBP1_HUMAN] | 3 | 2 | 1 | 1410 | 152.4 | 8.6 |
| Q8ND24 | RING finger protein 214 OS=Homo sapiens GN=RNF214 PE=1 SV=2 - [RN214_HUMAN] | 3 | 2 | 1 | 703 | 77.6 | 6.95 |
| Q96SB3 | Neurabin-2 OS=Homo sapiens GN=PPP1R9B PE=1 SV=2 - [NEB2_HUMAN] | 3 | 2 | 1 | 815 | 89.1 | 4.97 |
| Q9UNF0 | Protein kinase C and casein kinase substrate in neurons protein 2 OS=Homo sapiens GN=PACSIN2 PE=1 SV=2 - [PACN2_HUMAN] | 3 | 2 | 1 | 486 | 55.7 | 5.2 |
| O43615 | Mitochondrial import inner membrane translocase subunit TIM44 OS=Homo sapiens GN=TIMM44 PE=1 SV=2 - [TIM44_HUMAN] | 3 | 2 | 1 | 452 | 51.3 | 8.32 |
| Q12907 | Vesicular integral-membrane protein VIP36 OS=Homo sapiens GN=LMAN2 PE=1 SV=1 - [LMAN2_HUMAN] | 3 | 2 | 1 | 356 | 40.2 | 6.95 |
| O60256 | Phosphoribosyl pyrophosphate synthase-associated protein 2 OS=Homo sapiens GN=PRPSAP2 PE=1 SV=1 - [KPRB_HUMAN] | 3 | 2 | 1 | 369 | 40.9 | 7.44 |
| O75663 | TIP41-like protein OS=Homo sapiens GN=TIPRL PE=1 SV=2 - [TIPRL_HUMAN] | 3 | 2 | 1 | 272 | 31.4 | 5.91 |
| P02765 | Alpha-2-HS-glycoprotein OS=Homo sapiens GN=AHSG PE=1 SV=1 - [FETUA_HUMAN] | 3 | 6 | 2 | 367 | 39.3 | 5.72 |
| O75643 | U5 small nuclear ribonucleoprotein 200 kDa helicase OS=Homo sapiens GN=SNRNP200 PE=1 SV=2 - [U520_HUMAN] | 3 | 3 | 1 | 2136 | 244.4 | 6.06 |
| Q7Z3B4 | Nucleoporin p54 OS=Homo sapiens GN=NUP54 PE=1 SV=2 - [NUP54_HUMAN] | 3 | 3 | 1 | 507 | 55.4 | 7.02 |
| P55263 | Adenosine kinase OS=Homo sapiens GN=ADK PE=1 SV=2 - [ADK_HUMAN] | 3 | 3 | 1 | 362 | 40.5 | 6.7 |
| P33240 | Cleavage stimulation factor subunit 2 OS=Homo sapiens GN=CSTF2 PE=1 SV=1 - [CSTF2_HUMAN] | 3 | 3 | 1 | 577 | 60.9 | 6.83 |
| O00154 | Cytosolic acyl coenzyme A thioester hydrolase OS=Homo sapiens GN=ACOT7 PE=1 SV=3 - [BACH_HUMAN] | 3 | 3 | 1 | 380 | 41.8 | 8.54 |
| Q92665 | 28S ribosomal protein S31, mitochondrial OS=Homo sapiens GN=MRPS31 PE=1 SV=3 - [RT31_HUMAN] | 3 | 3 | 1 | 395 | 45.3 | 9.29 |
| O43657 | Tetraspanin-6 OS=Homo sapiens GN=TSPAN6 PE=1 SV=1 - [TSN6_HUMAN] | 3 | 3 | 1 | 245 | 27.5 | 8.1 |
| Q9Y5Z4 | Heme-binding protein 2 OS=Homo sapiens GN=HEBP2 PE=1 SV=1 - [HEBP2_HUMAN] | 3 | 3 | 1 | 205 | 22.9 | 4.63 |
| O14737 | Programmed cell death protein 5 OS=Homo sapiens GN=PDCD5 PE=1 SV=3 - [PDCD5_HUMAN] | 3 | 3 | 1 | 125 | 14.3 | 6.04 |
| P68431 | Histone H3.1 OS=Homo sapiens GN=HIST1H3A PE=1 SV=2 - [H31_HUMAN] | 3 | 4 | 1 | 136 | 15.4 | 11.12 |
| P06396 | Gelsolin OS=Homo sapiens GN=GSN PE=1 SV=1 - [GELS_HUMAN] | 3 | 3 | 0 | 782 | 85.6 | 6.28 |
| P11802 | Cell division protein kinase 4 OS=Homo sapiens GN=CDK4 PE=1 SV=2 - [CDK4_HUMAN] | 2 | 0 | 4 | 303 | 33.7 | 7.01 |
| O43169 | Cytochrome b5 type B OS=Homo sapiens GN=CYB5B PE=1 SV=2 - [CYB5B_HUMAN] | 2 | 0 | 3 | 146 | 16.3 | 4.97 |
| Q9NRX4 | 14 kDa phosphohistidine phosphatase OS=Homo sapiens GN=PHPT1 PE=1 SV=1 - [PHP14_HUMAN] | 2 | 0 | 3 | 125 | 13.8 | 6.07 |
| Q14011 | Cold-inducible RNA-binding protein OS=Homo sapiens GN=CIRBP PE=1 SV=1 - [CIRBP_HUMAN] | 2 | 0 | 3 | 172 | 18.6 | 9.51 |
| O14602 | Eukaryotic translation initiation factor 1A, Y-chromosomal OS=Homo sapiens GN=EIF1AY PE=1 SV=4 - [IF1AY_HUMAN] | 2 | 0 | 3 | 144 | 16.4 | 5.24 |
| Q9NX40 | OCIA domain-containing protein 1 OS=Homo sapiens GN=OCIAD1 PE=1 SV=1 - [OCAD1_HUMAN] | 2 | 0 | 3 | 245 | 27.6 | 7.49 |
| O15160 | DNA-directed RNA polymerases I and III subunit RPAC1 OS=Homo sapiens GN=POLR1C PE=1 SV=1 - [RPAC1_HUMAN] | 2 | 0 | 3 | 346 | 39.2 | 5.5 |
| Q9HD33 | 39S ribosomal protein L47, mitochondrial OS=Homo sapiens GN=MRPL47 PE=1 SV=2 - [RM47_HUMAN] | 2 | 0 | 3 | 250 | 29.4 | 10.37 |
| Q9C0H2 | Protein tweety homolog 3 OS=Homo sapiens GN=TTYH3 PE=1 SV=3 - [TTYH3_HUMAN] | 2 | 0 | 3 | 523 | 57.5 | 5.39 |
| Q92783 | Signal transducing adapter molecule 1 OS=Homo sapiens GN=STAM PE=1 SV=3 - [STAM1_HUMAN] | 2 | 0 | 3 | 540 | 59.1 | 4.82 |
| Q9UK76 | Hematological and neurological expressed 1 protein OS=Homo sapiens GN=HN1 PE=1 SV=3 - [HN1_HUMAN] | 2 | 0 | 2 | 154 | 16 | 5.6 |
| O00483 | NADH dehydrogenase [ubiquinone] 1 alpha subcomplex subunit 4 OS=Homo sapiens GN=NDUFA4 PE=1 SV=1 - [NDUA4_HUMAN] | 2 | 0 | 2 | 81 | 9.4 | 9.38 |
| Q9NWV4 | UPF0587 protein C1orf123 OS=Homo sapiens GN=C1orf123 PE=1 SV=1 - [CA123_HUMAN] | 2 | 0 | 2 | 160 | 18 | 5.01 |
| Q9UL45 | Biogenesis of lysosome-related organelles complex 1 subunit 6 OS=Homo sapiens GN=BLOC1S6 PE=1 SV=1 - [BL1S6_HUMAN] | 2 | 0 | 2 | 172 | 19.7 | 6.4 |
| P61970 | Nuclear transport factor 2 OS=Homo sapiens GN=NUTF2 PE=1 SV=1 - [NTF2_HUMAN] | 2 | 0 | 2 | 127 | 14.5 | 5.38 |
| Q8N183 | Mimitin, mitochondrial OS=Homo sapiens GN=NDUFAF2 PE=1 SV=1 - [MIMIT_HUMAN] | 2 | 0 | 2 | 169 | 19.8 | 8.97 |
| Q9Y3B4 | Pre-mRNA branch site protein p14 OS=Homo sapiens GN=SF3B14 PE=1 SV=1 - [PM14_HUMAN] | 2 | 0 | 2 | 125 | 14.6 | 9.38 |
| Q9NX14 | NADH dehydrogenase [ubiquinone] 1 beta subcomplex subunit 11, mitochondrial OS=Homo sapiens GN=NDUFB11 PE=1 SV=1 - [NDUBB_HUMAN] | 2 | 0 | 2 | 153 | 17.3 | 5.22 |
| Q9UI09 | NADH dehydrogenase [ubiquinone] 1 alpha subcomplex subunit 12 OS=Homo sapiens GN=NDUFA12 PE=1 SV=1 - [NDUAC_HUMAN] | 2 | 0 | 2 | 145 | 17.1 | 9.63 |
| Q8N5M4 | Tetratricopeptide repeat protein 9C OS=Homo sapiens GN=TTC9C PE=1 SV=1 - [TTC9C_HUMAN] | 2 | 0 | 2 | 171 | 20 | 8.92 |
| P20674 | Cytochrome c oxidase subunit 5A, mitochondrial OS=Homo sapiens GN=COX5A PE=1 SV=2 - [COX5A_HUMAN] | 2 | 0 | 2 | 150 | 16.8 | 6.79 |
| Q96B26 | Exosome complex component RRP43 OS=Homo sapiens GN=EXOSC8 PE=1 SV=1 - [EXOS8_HUMAN] | 2 | 0 | 2 | 276 | 30 | 5.3 |
| P62256 | Ubiquitin-conjugating enzyme E2 H OS=Homo sapiens GN=UBE2H PE=1 SV=1 - [UBE2H_HUMAN] | 2 | 0 | 2 | 183 | 20.6 | 4.67 |
| Q8TAE8 | Growth arrest and DNA damage-inducible proteins-interacting protein 1 OS=Homo sapiens GN=GADD45GIP1 PE=1 SV=1 - [G45IP_HUMAN] | 2 | 0 | 2 | 222 | 25.4 | 10.02 |
| Q9NPA0 | UPF0480 protein C15orf24 OS=Homo sapiens GN=C15orf24 PE=1 SV=1 - [CO024_HUMAN] | 2 | 0 | 2 | 242 | 26.5 | 9.25 |
| Q9Y3E1 | Hepatoma-derived growth factor-related protein 3 OS=Homo sapiens GN=HDGFRP3 PE=1 SV=1 - [HDGR3_HUMAN] | 2 | 0 | 2 | 203 | 22.6 | 7.99 |
| Q9NV31 | U3 small nucleolar ribonucleoprotein protein IMP3 OS=Homo sapiens GN=IMP3 PE=1 SV=1 - [IMP3_HUMAN] | 2 | 0 | 2 | 184 | 21.8 | 9.5 |
| P13984 | General transcription factor IIF subunit 2 OS=Homo sapiens GN=GTF2F2 PE=1 SV=2 - [T2FB_HUMAN] | 2 | 0 | 2 | 249 | 28.4 | 9.23 |
| Q86X83 | COMM domain-containing protein 2 OS=Homo sapiens GN=COMMD2 PE=1 SV=2 - [COMD2_HUMAN] | 2 | 0 | 2 | 199 | 22.7 | 6.73 |
| Q9Y3A3 | MOB-like protein phocein OS=Homo sapiens GN=MOB4 PE=1 SV=1 - [PHOCN_HUMAN] | 2 | 0 | 2 | 225 | 26 | 5.78 |
| Q9NRX1 | RNA-binding protein PNO1 OS=Homo sapiens GN=PNO1 PE=1 SV=1 - [PNO1_HUMAN] | 2 | 0 | 2 | 252 | 27.9 | 9.73 |
| P52815 | 39S ribosomal protein L12, mitochondrial OS=Homo sapiens GN=MRPL12 PE=1 SV=2 - [RM12_HUMAN] | 2 | 0 | 2 | 198 | 21.3 | 8.87 |
| O95169 | NADH dehydrogenase [ubiquinone] 1 beta subcomplex subunit 8, mitochondrial OS=Homo sapiens GN=NDUFB8 PE=1 SV=1 - [NDUB8_HUMAN] | 2 | 0 | 2 | 186 | 21.8 | 6.8 |
| O95571 | Persulfide dioxygenase ETHE1, mitochondrial OS=Homo sapiens GN=ETHE1 PE=1 SV=2 - [ETHE1_HUMAN] | 2 | 0 | 2 | 254 | 27.9 | 6.83 |
| Q92520 | Protein FAM3C OS=Homo sapiens GN=FAM3C PE=1 SV=1 - [FAM3C_HUMAN] | 2 | 0 | 2 | 227 | 24.7 | 8.29 |
| Q9GZZ9 | Ubiquitin-like modifier-activating enzyme 5 OS=Homo sapiens GN=UBA5 PE=1 SV=1 - [UBA5_HUMAN] | 2 | 0 | 2 | 404 | 44.8 | 4.84 |
| P11766 | Alcohol dehydrogenase class-3 OS=Homo sapiens GN=ADH5 PE=1 SV=4 - [ADHX_HUMAN] | 2 | 0 | 2 | 374 | 39.7 | 7.49 |
| Q96DI7 | U5 small nuclear ribonucleoprotein 40 kDa protein OS=Homo sapiens GN=SNRNP40 PE=1 SV=1 - [SNR40_HUMAN] | 2 | 0 | 2 | 357 | 39.3 | 8.1 |
| P55081 | Microfibrillar-associated protein 1 OS=Homo sapiens GN=MFAP1 PE=1 SV=2 - [MFAP1_HUMAN] | 2 | 0 | 2 | 439 | 51.9 | 4.98 |
| Q92600 | Cell differentiation protein RCD1 homolog OS=Homo sapiens GN=RQCD1 PE=1 SV=1 - [RCD1_HUMAN] | 2 | 0 | 2 | 299 | 33.6 | 8.03 |
| Q9BYD3 | 39S ribosomal protein L4, mitochondrial OS=Homo sapiens GN=MRPL4 PE=1 SV=1 - [RM04_HUMAN] | 2 | 0 | 2 | 311 | 34.9 | 9.72 |
| Q7Z4H3 | HD domain-containing protein 2 OS=Homo sapiens GN=HDDC2 PE=1 SV=1 - [HDDC2_HUMAN] | 2 | 0 | 2 | 204 | 23.4 | 5.49 |
| Q9C0F1 | Centrosomal protein of 44 kDa OS=Homo sapiens GN=CEP44 PE=1 SV=2 - [CEP44_HUMAN] | 2 | 0 | 2 | 390 | 44.1 | 5.21 |
| Q8N9N7 | Leucine-rich repeat-containing protein 57 OS=Homo sapiens GN=LRRC57 PE=1 SV=1 - [LRC57_HUMAN] | 2 | 0 | 2 | 239 | 26.7 | 8.43 |
| Q13445 | Transmembrane emp24 domain-containing protein 1 OS=Homo sapiens GN=TMED1 PE=1 SV=1 - [TMED1_HUMAN] | 2 | 0 | 2 | 227 | 25.2 | 4.48 |
| Q8NBN7 | Retinol dehydrogenase 13 OS=Homo sapiens GN=RDH13 PE=1 SV=2 - [RDH13_HUMAN] | 2 | 0 | 2 | 331 | 35.9 | 8.1 |
| Q9NVS9 | Pyridoxine-5'-phosphate oxidase OS=Homo sapiens GN=PNPO PE=1 SV=1 - [PNPO_HUMAN] | 2 | 0 | 2 | 261 | 30 | 7.06 |
| Q9Y316 | Protein MEMO1 OS=Homo sapiens GN=MEMO1 PE=1 SV=1 - [MEMO1_HUMAN] | 2 | 0 | 2 | 297 | 33.7 | 7.14 |
| O00233 | 26S proteasome non-ATPase regulatory subunit 9 OS=Homo sapiens GN=PSMD9 PE=1 SV=3 - [PSMD9_HUMAN] | 2 | 0 | 2 | 223 | 24.7 | 6.95 |
| P55145 | Mesencephalic astrocyte-derived neurotrophic factor OS=Homo sapiens GN=MANF PE=1 SV=3 - [MANF_HUMAN] | 2 | 0 | 2 | 182 | 20.7 | 8.69 |
| Q92733 | Proline-rich protein PRCC OS=Homo sapiens GN=PRCC PE=1 SV=1 - [PRCC_HUMAN] | 2 | 0 | 2 | 491 | 52.4 | 5.1 |
| P61962 | DDB1- and CUL4-associated factor 7 OS=Homo sapiens GN=DCAF7 PE=1 SV=1 - [DCAF7_HUMAN] | 2 | 0 | 2 | 342 | 38.9 | 5.52 |
| Q00059 | Transcription factor A, mitochondrial OS=Homo sapiens GN=TFAM PE=1 SV=1 - [TFAM_HUMAN] | 2 | 0 | 2 | 246 | 29.1 | 9.72 |
| Q07960 | Rho GTPase-activating protein 1 OS=Homo sapiens GN=ARHGAP1 PE=1 SV=1 - [RHG01_HUMAN] | 2 | 0 | 2 | 439 | 50.4 | 6.29 |
| Q9H6V9 | UPF0554 protein C2orf43 OS=Homo sapiens GN=C2orf43 PE=1 SV=1 - [CB043_HUMAN] | 2 | 0 | 2 | 325 | 37.3 | 6.54 |
| P29084 | Transcription initiation factor IIE subunit beta OS=Homo sapiens GN=GTF2E2 PE=1 SV=1 - [T2EB_HUMAN] | 2 | 0 | 2 | 291 | 33 | 9.66 |
| O75063 | Protein FAM20B OS=Homo sapiens GN=FAM20B PE=2 SV=1 - [FA20B_HUMAN] | 2 | 0 | 2 | 409 | 46.4 | 6.87 |
| Q99436 | Proteasome subunit beta type-7 OS=Homo sapiens GN=PSMB7 PE=1 SV=1 - [PSB7_HUMAN] | 2 | 0 | 2 | 277 | 29.9 | 7.68 |
| Q13642 | Four and a half LIM domains protein 1 OS=Homo sapiens GN=FHL1 PE=1 SV=4 - [FHL1_HUMAN] | 2 | 0 | 2 | 323 | 36.2 | 8.97 |
| Q6NVY1 | 3-hydroxyisobutyryl-CoA hydrolase, mitochondrial OS=Homo sapiens GN=HIBCH PE=1 SV=2 - [HIBCH_HUMAN] | 2 | 0 | 2 | 386 | 43.5 | 8.19 |
| Q05048 | Cleavage stimulation factor subunit 1 OS=Homo sapiens GN=CSTF1 PE=1 SV=1 - [CSTF1_HUMAN] | 2 | 0 | 2 | 431 | 48.3 | 6.58 |
| Q9Y6W5 | Wiskott-Aldrich syndrome protein family member 2 OS=Homo sapiens GN=WASF2 PE=1 SV=3 - [WASF2_HUMAN] | 2 | 0 | 2 | 498 | 54.3 | 5.53 |
| O43148 | mRNA cap guanine-N7 methyltransferase OS=Homo sapiens GN=RNMT PE=1 SV=1 - [MCES_HUMAN] | 2 | 0 | 2 | 476 | 54.8 | 6.61 |
| Q7Z417 | Nuclear fragile X mental retardation-interacting protein 2 OS=Homo sapiens GN=NUFIP2 PE=1 SV=1 - [NUFP2_HUMAN] | 2 | 0 | 2 | 695 | 76.1 | 8.7 |
| Q5F1R6 | DnaJ homolog subfamily C member 21 OS=Homo sapiens GN=DNAJC21 PE=1 SV=2 - [DJC21_HUMAN] | 2 | 0 | 2 | 531 | 62 | 5.47 |
| Q06546 | GA-binding protein alpha chain OS=Homo sapiens GN=GABPA PE=1 SV=1 - [GABPA_HUMAN] | 2 | 0 | 2 | 454 | 51.3 | 4.97 |
| Q9UBL3 | Set1/Ash2 histone methyltransferase complex subunit ASH2 OS=Homo sapiens GN=ASH2L PE=1 SV=1 - [ASH2L_HUMAN] | 2 | 0 | 2 | 628 | 68.7 | 5.69 |
| Q9H0C8 | Integrin-linked kinase-associated serine/threonine phosphatase 2C OS=Homo sapiens GN=ILKAP PE=1 SV=1 - [ILKAP_HUMAN] | 2 | 0 | 2 | 392 | 42.9 | 7.09 |
| Q8IUF8 | Bifunctional lysine-specific demethylase and histidyl-hydroxylase MINA OS=Homo sapiens GN=MINA PE=1 SV=1 - [MINA_HUMAN] | 2 | 0 | 2 | 465 | 52.8 | 6.7 |
| Q9GZT8 | NIF3-like protein 1 OS=Homo sapiens GN=NIF3L1 PE=1 SV=2 - [NIF3L_HUMAN] | 2 | 0 | 2 | 377 | 41.9 | 6.65 |
| Q13685 | Angio-associated migratory cell protein OS=Homo sapiens GN=AAMP PE=1 SV=2 - [AAMP_HUMAN] | 2 | 0 | 2 | 434 | 46.7 | 4.42 |
| P10619 | Lysosomal protective protein OS=Homo sapiens GN=CTSA PE=1 SV=2 - [PPGB_HUMAN] | 2 | 0 | 2 | 480 | 54.4 | 6.61 |
| Q8N6H7 | ADP-ribosylation factor GTPase-activating protein 2 OS=Homo sapiens GN=ARFGAP2 PE=1 SV=1 - [ARFG2_HUMAN] | 2 | 0 | 2 | 521 | 56.7 | 7.99 |
| Q3KQV9 | UDP-N-acetylhexosamine pyrophosphorylase-like protein 1 OS=Homo sapiens GN=UAP1L1 PE=2 SV=2 - [UAP1L_HUMAN] | 2 | 0 | 2 | 507 | 57 | 6.32 |
| Q9UH03 | Neuronal-specific septin-3 OS=Homo sapiens GN=SEPT3 PE=1 SV=3 - [SEPT3_HUMAN] | 2 | 0 | 2 | 358 | 40.7 | 7.2 |
| Q96NU1 | Sterile alpha motif domain-containing protein 11 OS=Homo sapiens GN=SAMD11 PE=1 SV=3 - [SAM11_HUMAN] | 2 | 0 | 2 | 681 | 72.7 | 7.59 |
| P49643 | DNA primase large subunit OS=Homo sapiens GN=PRIM2 PE=1 SV=2 - [PRI2_HUMAN] | 2 | 0 | 2 | 509 | 58.8 | 7.91 |
| Q9UHY1 | Nuclear receptor-binding protein OS=Homo sapiens GN=NRBP1 PE=1 SV=1 - [NRBP_HUMAN] | 2 | 0 | 2 | 535 | 59.8 | 5.08 |
| Q9BVL2 | Nucleoporin p58/p45 OS=Homo sapiens GN=NUP58 PE=1 SV=1 - [NUP58_HUMAN] | 2 | 0 | 2 | 599 | 60.9 | 9.33 |
| Q96G46 | tRNA-dihydrouridine synthase 3-like OS=Homo sapiens GN=DUS3L PE=1 SV=2 - [DUS3L_HUMAN] | 2 | 0 | 2 | 650 | 72.5 | 8.05 |
| Q6NUQ4 | Transmembrane protein 214 OS=Homo sapiens GN=TMEM214 PE=1 SV=2 - [TM214_HUMAN] | 2 | 0 | 2 | 689 | 77.1 | 9.14 |
| Q5T0N5 | Formin-binding protein 1-like OS=Homo sapiens GN=FNBP1L PE=1 SV=3 - [FBP1L_HUMAN] | 2 | 0 | 2 | 605 | 70 | 6.64 |
| Q7Z2K8 | G protein-regulated inducer of neurite outgrowth 1 OS=Homo sapiens GN=GPRIN1 PE=1 SV=2 - [GRIN1_HUMAN] | 2 | 0 | 2 | 1008 | 102.3 | 8.06 |
| Q9H501 | ESF1 homolog OS=Homo sapiens GN=ESF1 PE=1 SV=1 - [ESF1_HUMAN] | 2 | 0 | 2 | 851 | 98.7 | 5.11 |
| O00469 | Procollagen-lysine,2-oxoglutarate 5-dioxygenase 2 OS=Homo sapiens GN=PLOD2 PE=1 SV=2 - [PLOD2_HUMAN] | 2 | 0 | 2 | 737 | 84.6 | 6.71 |
| Q6IN85 | Serine/threonine-protein phosphatase 4 regulatory subunit 3A OS=Homo sapiens GN=SMEK1 PE=1 SV=1 - [P4R3A_HUMAN] | 2 | 0 | 2 | 833 | 95.3 | 4.94 |
| Q13618 | Cullin-3 OS=Homo sapiens GN=CUL3 PE=1 SV=2 - [CUL3_HUMAN] | 2 | 0 | 2 | 768 | 88.9 | 8.48 |
| Q9NR30 | Nucleolar RNA helicase 2 OS=Homo sapiens GN=DDX21 PE=1 SV=5 - [DDX21_HUMAN] | 2 | 0 | 2 | 783 | 87.3 | 9.28 |
| Q8IXK0 | Polyhomeotic-like protein 2 OS=Homo sapiens GN=PHC2 PE=1 SV=1 - [PHC2_HUMAN] | 2 | 0 | 2 | 858 | 90.7 | 8.69 |
| Q9BSJ8 | Extended synaptotagmin-1 OS=Homo sapiens GN=ESYT1 PE=1 SV=1 - [ESYT1_HUMAN] | 2 | 0 | 2 | 1104 | 122.8 | 5.83 |
| Q9UJ41 | Rab5 GDP/GTP exchange factor OS=Homo sapiens GN=RABGEF1 PE=1 SV=2 - [RABX5_HUMAN] | 2 | 0 | 2 | 708 | 79.3 | 6.81 |
| Q5VT52 | Regulation of nuclear pre-mRNA domain-containing protein 2 OS=Homo sapiens GN=RPRD2 PE=1 SV=1 - [RPRD2_HUMAN] | 2 | 0 | 2 | 1461 | 155.9 | 7.42 |
| O60502 | Bifunctional protein NCOAT OS=Homo sapiens GN=MGEA5 PE=1 SV=2 - [NCOAT_HUMAN] | 2 | 0 | 2 | 916 | 102.8 | 4.91 |
| Q9UDY2 | Tight junction protein ZO-2 OS=Homo sapiens GN=TJP2 PE=1 SV=2 - [ZO2_HUMAN] | 2 | 0 | 2 | 1190 | 133.9 | 7.4 |
| Q9NYU2 | UDP-glucose:glycoprotein glucosyltransferase 1 OS=Homo sapiens GN=UGGT1 PE=1 SV=3 - [UGGG1_HUMAN] | 2 | 0 | 2 | 1555 | 177.1 | 5.63 |
| P78527 | DNA-dependent protein kinase catalytic subunit OS=Homo sapiens GN=PRKDC PE=1 SV=3 - [PRKDC_HUMAN] | 2 | 0 | 2 | 4128 | 468.8 | 7.12 |
| O43663 | Protein regulator of cytokinesis 1 OS=Homo sapiens GN=PRC1 PE=1 SV=2 - [PRC1_HUMAN] | 2 | 1 | 4 | 620 | 71.6 | 6.68 |
| Q99627 | COP9 signalosome complex subunit 8 OS=Homo sapiens GN=COPS8 PE=1 SV=1 - [CSN8_HUMAN] | 2 | 1 | 4 | 209 | 23.2 | 5.38 |
| Q9UMX0 | Ubiquilin-1 OS=Homo sapiens GN=UBQLN1 PE=1 SV=2 - [UBQL1_HUMAN] | 2 | 2 | 6 | 589 | 62.5 | 5.11 |
| P31323 | cAMP-dependent protein kinase type II-beta regulatory subunit OS=Homo sapiens GN=PRKAR2B PE=1 SV=3 - [KAP3_HUMAN] | 2 | 1 | 3 | 418 | 46.3 | 4.92 |
| Q9NR31 | GTP-binding protein SAR1a OS=Homo sapiens GN=SAR1A PE=1 SV=1 - [SAR1A_HUMAN] | 2 | 1 | 3 | 198 | 22.4 | 6.68 |
| Q9BWD1 | Acetyl-CoA acetyltransferase, cytosolic OS=Homo sapiens GN=ACAT2 PE=1 SV=2 - [THIC_HUMAN] | 2 | 1 | 3 | 397 | 41.3 | 6.92 |
| P61956 | Small ubiquitin-related modifier 2 OS=Homo sapiens GN=SUMO2 PE=1 SV=1 - [SUMO2_HUMAN] | 2 | 1 | 3 | 95 | 10.9 | 5.5 |
| P62314 | Small nuclear ribonucleoprotein Sm D1 OS=Homo sapiens GN=SNRPD1 PE=1 SV=1 - [SMD1_HUMAN] | 2 | 1 | 3 | 119 | 13.3 | 11.56 |
| O43432 | Eukaryotic translation initiation factor 4 gamma 3 OS=Homo sapiens GN=EIF4G3 PE=1 SV=2 - [IF4G3_HUMAN] | 2 | 2 | 5 | 1585 | 176.5 | 5.38 |
| Q9UKL0 | REST corepressor 1 OS=Homo sapiens GN=RCOR1 PE=1 SV=1 - [RCOR1_HUMAN] | 2 | 2 | 5 | 482 | 53 | 7.03 |
| Q14151 | Scaffold attachment factor B2 OS=Homo sapiens GN=SAFB2 PE=1 SV=1 - [SAFB2_HUMAN] | 2 | 3 | 6 | 953 | 107.4 | 6.16 |
| P01023 | Alpha-2-macroglobulin OS=Homo sapiens GN=A2M PE=1 SV=3 - [A2MG_HUMAN] | 2 | 2 | 4 | 1474 | 163.2 | 6.46 |
| P47755 | F-actin-capping protein subunit alpha-2 OS=Homo sapiens GN=CAPZA2 PE=1 SV=3 - [CAZA2_HUMAN] | 2 | 2 | 4 | 286 | 32.9 | 5.85 |
| P54725 | UV excision repair protein RAD23 homolog A OS=Homo sapiens GN=RAD23A PE=1 SV=1 - [RD23A_HUMAN] | 2 | 2 | 4 | 363 | 39.6 | 4.58 |
| P20340 | Ras-related protein Rab-6A OS=Homo sapiens GN=RAB6A PE=1 SV=3 - [RAB6A_HUMAN] | 2 | 2 | 4 | 208 | 23.6 | 5.54 |
| Q8TF05 | Serine/threonine-protein phosphatase 4 regulatory subunit 1 OS=Homo sapiens GN=PPP4R1 PE=1 SV=1 - [PP4R1_HUMAN] | 2 | 1 | 2 | 950 | 106.9 | 4.77 |
| Q13428 | Treacle protein OS=Homo sapiens GN=TCOF1 PE=1 SV=3 - [TCOF_HUMAN] | 2 | 1 | 2 | 1488 | 152 | 9.04 |
| P26640 | Valyl-tRNA synthetase OS=Homo sapiens GN=VARS PE=1 SV=4 - [SYVC_HUMAN] | 2 | 1 | 2 | 1264 | 140.4 | 7.59 |
| Q9UKG1 | DCC-interacting protein 13-alpha OS=Homo sapiens GN=APPL1 PE=1 SV=1 - [DP13A_HUMAN] | 2 | 1 | 2 | 709 | 79.6 | 5.41 |
| Q96PY6 | Serine/threonine-protein kinase Nek1 OS=Homo sapiens GN=NEK1 PE=1 SV=2 - [NEK1_HUMAN] | 2 | 1 | 2 | 1258 | 142.7 | 5.94 |
| Q96FV9 | THO complex subunit 1 OS=Homo sapiens GN=THOC1 PE=1 SV=1 - [THOC1_HUMAN] | 2 | 1 | 2 | 657 | 75.6 | 4.98 |
| P16435 | NADPH--cytochrome P450 reductase OS=Homo sapiens GN=POR PE=1 SV=2 - [NCPR_HUMAN] | 2 | 1 | 2 | 677 | 76.6 | 5.58 |
| Q12959 | Disks large homolog 1 OS=Homo sapiens GN=DLG1 PE=1 SV=2 - [DLG1_HUMAN] | 2 | 1 | 2 | 904 | 100.4 | 5.76 |
| P23368 | NAD-dependent malic enzyme, mitochondrial OS=Homo sapiens GN=ME2 PE=1 SV=1 - [MAOM_HUMAN] | 2 | 1 | 2 | 584 | 65.4 | 7.61 |
| Q9NUQ8 | ATP-binding cassette sub-family F member 3 OS=Homo sapiens GN=ABCF3 PE=1 SV=2 - [ABCF3_HUMAN] | 2 | 1 | 2 | 709 | 79.7 | 6.34 |
| Q13619 | Cullin-4A OS=Homo sapiens GN=CUL4A PE=1 SV=3 - [CUL4A_HUMAN] | 2 | 1 | 2 | 759 | 87.6 | 8.13 |
| Q05D32 | CTD small phosphatase-like protein 2 OS=Homo sapiens GN=CTDSPL2 PE=1 SV=2 - [CTSL2_HUMAN] | 2 | 1 | 2 | 466 | 53 | 6.4 |
| Q2TAL8 | Glutamine-rich protein 1 OS=Homo sapiens GN=QRICH1 PE=1 SV=1 - [QRIC1_HUMAN] | 2 | 1 | 2 | 776 | 86.4 | 5.87 |
| Q13427 | Peptidyl-prolyl cis-trans isomerase G OS=Homo sapiens GN=PPIG PE=1 SV=2 - [PPIG_HUMAN] | 2 | 1 | 2 | 754 | 88.6 | 10.29 |
| Q13033 | Striatin-3 OS=Homo sapiens GN=STRN3 PE=1 SV=3 - [STRN3_HUMAN] | 2 | 1 | 2 | 797 | 87.2 | 5.36 |
| P05067 | Amyloid beta A4 protein OS=Homo sapiens GN=APP PE=1 SV=3 - [A4_HUMAN] | 2 | 1 | 2 | 770 | 86.9 | 4.82 |
| P14859 | POU domain, class 2, transcription factor 1 OS=Homo sapiens GN=POU2F1 PE=1 SV=2 - [PO2F1_HUMAN] | 2 | 1 | 2 | 743 | 76.4 | 6.81 |
| Q96T37 | Putative RNA-binding protein 15 OS=Homo sapiens GN=RBM15 PE=1 SV=2 - [RBM15_HUMAN] | 2 | 1 | 2 | 977 | 107.1 | 10.08 |
| P07199 | Major centromere autoantigen B OS=Homo sapiens GN=CENPB PE=1 SV=2 - [CENPB_HUMAN] | 2 | 1 | 2 | 599 | 65.1 | 4.55 |
| Q96RP9 | Elongation factor G, mitochondrial OS=Homo sapiens GN=GFM1 PE=1 SV=2 - [EFGM_HUMAN] | 2 | 1 | 2 | 751 | 83.4 | 7.01 |
| Q9BUI4 | DNA-directed RNA polymerase III subunit RPC3 OS=Homo sapiens GN=POLR3C PE=1 SV=1 - [RPC3_HUMAN] | 2 | 1 | 2 | 534 | 60.6 | 7.31 |
| P21579 | Synaptotagmin-1 OS=Homo sapiens GN=SYT1 PE=1 SV=1 - [SYT1_HUMAN] | 2 | 1 | 2 | 422 | 47.5 | 8.12 |
| Q16539 | Mitogen-activated protein kinase 14 OS=Homo sapiens GN=MAPK14 PE=1 SV=3 - [MK14_HUMAN] | 2 | 1 | 2 | 360 | 41.3 | 5.78 |
| Q15645 | Thyroid receptor-interacting protein 13 OS=Homo sapiens GN=TRIP13 PE=1 SV=2 - [TRP13_HUMAN] | 2 | 1 | 2 | 432 | 48.5 | 6.09 |
| Q02818 | Nucleobindin-1 OS=Homo sapiens GN=NUCB1 PE=1 SV=4 - [NUCB1_HUMAN] | 2 | 1 | 2 | 461 | 53.8 | 5.25 |
| Q9UGV2 | Protein NDRG3 OS=Homo sapiens GN=NDRG3 PE=1 SV=2 - [NDRG3_HUMAN] | 2 | 1 | 2 | 375 | 41.4 | 5.31 |
| Q92905 | COP9 signalosome complex subunit 5 OS=Homo sapiens GN=COPS5 PE=1 SV=4 - [CSN5_HUMAN] | 2 | 1 | 2 | 334 | 37.6 | 6.54 |
| Q9NXV6 | CDKN2A-interacting protein OS=Homo sapiens GN=CDKN2AIP PE=1 SV=3 - [CARF_HUMAN] | 2 | 1 | 2 | 580 | 61.1 | 9.01 |
| Q9HB40 | Retinoid-inducible serine carboxypeptidase OS=Homo sapiens GN=SCPEP1 PE=1 SV=1 - [RISC_HUMAN] | 2 | 1 | 2 | 452 | 50.8 | 5.81 |
| Q9NUQ3 | Gamma-taxilin OS=Homo sapiens GN=TXLNG PE=1 SV=2 - [TXLNG_HUMAN] | 2 | 1 | 2 | 528 | 60.5 | 7.52 |
| Q9Y3B9 | RRP15-like protein OS=Homo sapiens GN=RRP15 PE=1 SV=2 - [RRP15_HUMAN] | 2 | 1 | 2 | 282 | 31.5 | 5.52 |
| Q9NY93 | Probable ATP-dependent RNA helicase DDX56 OS=Homo sapiens GN=DDX56 PE=1 SV=1 - [DDX56_HUMAN] | 2 | 1 | 2 | 547 | 61.6 | 9.26 |
| Q9UPQ0 | LIM and calponin homology domains-containing protein 1 OS=Homo sapiens GN=LIMCH1 PE=1 SV=4 - [LIMC1_HUMAN] | 2 | 1 | 2 | 1083 | 121.8 | 6.47 |
| P53041 | Serine/threonine-protein phosphatase 5 OS=Homo sapiens GN=PPP5C PE=1 SV=1 - [PPP5_HUMAN] | 2 | 1 | 2 | 499 | 56.8 | 6.28 |
| Q92552 | 28S ribosomal protein S27, mitochondrial OS=Homo sapiens GN=MRPS27 PE=1 SV=3 - [RT27_HUMAN] | 2 | 1 | 2 | 414 | 47.6 | 6.18 |
| O14662 | Syntaxin-16 OS=Homo sapiens GN=STX16 PE=1 SV=3 - [STX16_HUMAN] | 2 | 1 | 2 | 325 | 37 | 6.11 |
| Q9UJU6 | Drebrin-like protein OS=Homo sapiens GN=DBNL PE=1 SV=1 - [DBNL_HUMAN] | 2 | 1 | 2 | 430 | 48.2 | 5.05 |
| P56182 | Ribosomal RNA processing protein 1 homolog A OS=Homo sapiens GN=RRP1 PE=1 SV=1 - [RRP1_HUMAN] | 2 | 1 | 2 | 461 | 52.8 | 9.33 |
| Q9UNS2 | COP9 signalosome complex subunit 3 OS=Homo sapiens GN=COPS3 PE=1 SV=3 - [CSN3_HUMAN] | 2 | 1 | 2 | 423 | 47.8 | 6.65 |
| O14498 | Immunoglobulin superfamily containing leucine-rich repeat protein OS=Homo sapiens GN=ISLR PE=2 SV=1 - [ISLR_HUMAN] | 2 | 1 | 2 | 428 | 46 | 5.15 |
| Q96AT9 | Ribulose-phosphate 3-epimerase OS=Homo sapiens GN=RPE PE=1 SV=1 - [RPE_HUMAN] | 2 | 1 | 2 | 228 | 24.9 | 5.58 |
| O00232 | 26S proteasome non-ATPase regulatory subunit 12 OS=Homo sapiens GN=PSMD12 PE=1 SV=3 - [PSD12_HUMAN] | 2 | 1 | 2 | 456 | 52.9 | 7.65 |
| Q96S66 | Chloride channel CLIC-like protein 1 OS=Homo sapiens GN=CLCC1 PE=1 SV=1 - [CLCC1_HUMAN] | 2 | 1 | 2 | 551 | 62 | 5.55 |
| P10644 | cAMP-dependent protein kinase type I-alpha regulatory subunit OS=Homo sapiens GN=PRKAR1A PE=1 SV=1 - [KAP0_HUMAN] | 2 | 1 | 2 | 381 | 43 | 5.35 |
| Q00403 | Transcription initiation factor IIB OS=Homo sapiens GN=GTF2B PE=1 SV=1 - [TF2B_HUMAN] | 2 | 1 | 2 | 316 | 34.8 | 8.35 |
| P50750 | Cell division protein kinase 9 OS=Homo sapiens GN=CDK9 PE=1 SV=3 - [CDK9_HUMAN] | 2 | 1 | 2 | 372 | 42.8 | 8.79 |
| P20073 | Annexin A7 OS=Homo sapiens GN=ANXA7 PE=1 SV=3 - [ANXA7_HUMAN] | 2 | 1 | 2 | 488 | 52.7 | 5.68 |
| O00560 | Syntenin-1 OS=Homo sapiens GN=SDCBP PE=1 SV=1 - [SDCB1_HUMAN] | 2 | 1 | 2 | 298 | 32.4 | 7.53 |
| Q12800 | Alpha-globin transcription factor CP2 OS=Homo sapiens GN=TFCP2 PE=1 SV=2 - [TFCP2_HUMAN] | 2 | 1 | 2 | 502 | 57.2 | 5.8 |
| P18615 | Negative elongation factor E OS=Homo sapiens GN=RDBP PE=1 SV=3 - [NELFE_HUMAN] | 2 | 1 | 2 | 380 | 43.2 | 9.33 |
| Q8WWC4 | Uncharacterized protein C2orf47, mitochondrial OS=Homo sapiens GN=C2orf47 PE=1 SV=1 - [CB047_HUMAN] | 2 | 1 | 2 | 291 | 32.5 | 9.17 |
| O94905 | Erlin-2 OS=Homo sapiens GN=ERLIN2 PE=1 SV=1 - [ERLN2_HUMAN] | 2 | 1 | 2 | 339 | 37.8 | 5.62 |
| Q99873 | Protein arginine N-methyltransferase 1 OS=Homo sapiens GN=PRMT1 PE=1 SV=2 - [ANM1_HUMAN] | 2 | 1 | 2 | 361 | 41.5 | 5.43 |
| P61011 | Signal recognition particle 54 kDa protein OS=Homo sapiens GN=SRP54 PE=1 SV=1 - [SRP54_HUMAN] | 2 | 1 | 2 | 504 | 55.7 | 8.75 |
| P10909 | Clusterin OS=Homo sapiens GN=CLU PE=1 SV=1 - [CLUS_HUMAN] | 2 | 1 | 2 | 449 | 52.5 | 6.27 |
| O43251 | RNA binding protein fox-1 homolog 2 OS=Homo sapiens GN=RBFOX2 PE=1 SV=3 - [RFOX2_HUMAN] | 2 | 1 | 2 | 390 | 41.3 | 7.27 |
| P21281 | V-type proton ATPase subunit B, brain isoform OS=Homo sapiens GN=ATP6V1B2 PE=1 SV=3 - [VATB2_HUMAN] | 2 | 1 | 2 | 511 | 56.5 | 5.81 |
| P36542 | ATP synthase subunit gamma, mitochondrial OS=Homo sapiens GN=ATP5C1 PE=1 SV=1 - [ATPG_HUMAN] | 2 | 1 | 2 | 298 | 33 | 9.22 |
| P51970 | NADH dehydrogenase [ubiquinone] 1 alpha subcomplex subunit 8 OS=Homo sapiens GN=NDUFA8 PE=1 SV=3 - [NDUA8_HUMAN] | 2 | 1 | 2 | 172 | 20.1 | 7.65 |
| P05026 | Sodium/potassium-transporting ATPase subunit beta-1 OS=Homo sapiens GN=ATP1B1 PE=1 SV=1 - [AT1B1_HUMAN] | 2 | 1 | 2 | 303 | 35 | 8.53 |
| P42126 | 3,2-trans-enoyl-CoA isomerase, mitochondrial OS=Homo sapiens GN=DCI PE=1 SV=1 - [D3D2_HUMAN] | 2 | 1 | 2 | 302 | 32.8 | 8.54 |
| Q8TC12 | Retinol dehydrogenase 11 OS=Homo sapiens GN=RDH11 PE=1 SV=2 - [RDH11_HUMAN] | 2 | 1 | 2 | 318 | 35.4 | 8.82 |
| O14828 | Secretory carrier-associated membrane protein 3 OS=Homo sapiens GN=SCAMP3 PE=1 SV=3 - [SCAM3_HUMAN] | 2 | 1 | 2 | 347 | 38.3 | 7.64 |
| Q9BTE7 | DCN1-like protein 5 OS=Homo sapiens GN=DCUN1D5 PE=1 SV=1 - [DCNL5_HUMAN] | 2 | 1 | 2 | 237 | 27.5 | 5.58 |
| Q5HYI8 | Rab-like protein 3 OS=Homo sapiens GN=RABL3 PE=1 SV=1 - [RABL3_HUMAN] | 2 | 1 | 2 | 236 | 26.4 | 7.11 |
| P24539 | ATP synthase subunit b, mitochondrial OS=Homo sapiens GN=ATP5F1 PE=1 SV=2 - [AT5F1_HUMAN] | 2 | 1 | 2 | 256 | 28.9 | 9.36 |
| Q9NT62 | Autophagy-related protein 3 OS=Homo sapiens GN=ATG3 PE=1 SV=1 - [ATG3_HUMAN] | 2 | 1 | 2 | 314 | 35.8 | 4.74 |
| O95297 | Myelin protein zero-like protein 1 OS=Homo sapiens GN=MPZL1 PE=1 SV=1 - [MPZL1_HUMAN] | 2 | 1 | 2 | 269 | 29.1 | 8.72 |
| Q92890 | Ubiquitin fusion degradation protein 1 homolog OS=Homo sapiens GN=UFD1L PE=1 SV=3 - [UFD1_HUMAN] | 2 | 1 | 2 | 307 | 34.5 | 6.7 |
| Q9UMS0 | NFU1 iron-sulfur cluster scaffold homolog, mitochondrial OS=Homo sapiens GN=NFU1 PE=1 SV=2 - [NFU1_HUMAN] | 2 | 1 | 2 | 254 | 28.4 | 5.07 |
| Q8N983 | 39S ribosomal protein L43, mitochondrial OS=Homo sapiens GN=MRPL43 PE=1 SV=1 - [RM43_HUMAN] | 2 | 1 | 2 | 215 | 23.4 | 8.65 |
| Q8NFH4 | Nucleoporin Nup37 OS=Homo sapiens GN=NUP37 PE=1 SV=1 - [NUP37_HUMAN] | 2 | 1 | 2 | 326 | 36.7 | 5.92 |
| Q9UMY4 | Sorting nexin-12 OS=Homo sapiens GN=SNX12 PE=1 SV=3 - [SNX12_HUMAN] | 2 | 1 | 2 | 172 | 19.7 | 7.87 |
| Q9H6E4 | Coiled-coil domain-containing protein 134 OS=Homo sapiens GN=CCDC134 PE=1 SV=1 - [CC134_HUMAN] | 2 | 1 | 2 | 229 | 26.5 | 8.85 |
| O15116 | U6 snRNA-associated Sm-like protein LSm1 OS=Homo sapiens GN=LSM1 PE=1 SV=1 - [LSM1_HUMAN] | 2 | 1 | 2 | 133 | 15.2 | 5.22 |
| Q14919 | Dr1-associated corepressor OS=Homo sapiens GN=DRAP1 PE=1 SV=3 - [NC2A_HUMAN] | 2 | 1 | 2 | 205 | 22.3 | 5.17 |
| Q13242 | Splicing factor, arginine/serine-rich 9 OS=Homo sapiens GN=SFRS9 PE=1 SV=1 - [SFRS9_HUMAN] | 2 | 1 | 2 | 221 | 25.5 | 8.65 |
| O43447 | Peptidyl-prolyl cis-trans isomerase H OS=Homo sapiens GN=PPIH PE=1 SV=1 - [PPIH_HUMAN] | 2 | 1 | 2 | 177 | 19.2 | 8.07 |
| Q9UPY8 | Microtubule-associated protein RP/EB family member 3 OS=Homo sapiens GN=MAPRE3 PE=1 SV=1 - [MARE3_HUMAN] | 2 | 1 | 2 | 281 | 32 | 5.54 |
| Q9UHQ9 | NADH-cytochrome b5 reductase 1 OS=Homo sapiens GN=CYB5R1 PE=1 SV=1 - [NB5R1_HUMAN] | 2 | 1 | 2 | 305 | 34.1 | 9.38 |
| Q9GZL7 | Ribosome biogenesis protein WDR12 OS=Homo sapiens GN=WDR12 PE=1 SV=2 - [WDR12_HUMAN] | 2 | 1 | 2 | 423 | 47.7 | 5.9 |
| O15212 | Prefoldin subunit 6 OS=Homo sapiens GN=PFDN6 PE=1 SV=1 - [PFD6_HUMAN] | 2 | 1 | 2 | 129 | 14.6 | 8.88 |
| Q9BY42 | Protein RTF2 homolog OS=Homo sapiens GN=RTFDC1 PE=1 SV=3 - [RTF2_HUMAN] | 2 | 1 | 2 | 306 | 33.9 | 8.59 |
| O43583 | Density-regulated protein OS=Homo sapiens GN=DENR PE=1 SV=2 - [DENR_HUMAN] | 2 | 1 | 2 | 198 | 22.1 | 5.3 |
| Q53FT3 | Uncharacterized protein C11orf73 OS=Homo sapiens GN=C11orf73 PE=1 SV=2 - [CK073_HUMAN] | 2 | 1 | 2 | 197 | 21.6 | 5.45 |
| O75431 | Metaxin-2 OS=Homo sapiens GN=MTX2 PE=1 SV=1 - [MTX2_HUMAN] | 2 | 1 | 2 | 263 | 29.7 | 6.29 |
| Q9NQ50 | 39S ribosomal protein L40, mitochondrial OS=Homo sapiens GN=MRPL40 PE=1 SV=1 - [RM40_HUMAN] | 2 | 1 | 2 | 206 | 24.5 | 9.63 |
| Q9NX62 | Inositol monophosphatase 3 OS=Homo sapiens GN=IMPAD1 PE=1 SV=1 - [IMPA3_HUMAN] | 2 | 1 | 2 | 359 | 38.7 | 6.86 |
| Q9UEU0 | Vesicle transport through interaction with t-SNAREs homolog 1B OS=Homo sapiens GN=VTI1B PE=1 SV=3 - [VTI1B_HUMAN] | 2 | 1 | 2 | 232 | 26.7 | 9.04 |
| Q8TDP1 | Ribonuclease H2 subunit C OS=Homo sapiens GN=RNASEH2C PE=1 SV=1 - [RNH2C_HUMAN] | 2 | 1 | 2 | 164 | 17.8 | 5.03 |
| O00762 | Ubiquitin-conjugating enzyme E2 C OS=Homo sapiens GN=UBE2C PE=1 SV=1 - [UBE2C_HUMAN] | 2 | 1 | 2 | 179 | 19.6 | 7.37 |
| Q04323 | UBX domain-containing protein 1 OS=Homo sapiens GN=UBXN1 PE=1 SV=2 - [UBXN1_HUMAN] | 2 | 1 | 2 | 297 | 33.3 | 5.25 |
| P84090 | Enhancer of rudimentary homolog OS=Homo sapiens GN=ERH PE=1 SV=1 - [ERH_HUMAN] | 2 | 1 | 2 | 104 | 12.3 | 5.92 |
| Q9Y2V2 | Calcium-regulated heat stable protein 1 OS=Homo sapiens GN=CARHSP1 PE=1 SV=2 - [CHSP1_HUMAN] | 2 | 1 | 2 | 147 | 15.9 | 8.21 |
| Q9NQP4 | Prefoldin subunit 4 OS=Homo sapiens GN=PFDN4 PE=1 SV=1 - [PFD4_HUMAN] | 2 | 1 | 2 | 134 | 15.3 | 4.53 |
| Q9UI30 | tRNA methyltransferase 112 homolog OS=Homo sapiens GN=TRMT112 PE=1 SV=1 - [TR112_HUMAN] | 2 | 1 | 2 | 125 | 14.2 | 5.26 |
| P60983 | Glia maturation factor beta OS=Homo sapiens GN=GMFB PE=1 SV=2 - [GMFB_HUMAN] | 2 | 1 | 2 | 142 | 16.7 | 5.29 |
| P10599 | Thioredoxin OS=Homo sapiens GN=TXN PE=1 SV=3 - [THIO_HUMAN] | 2 | 1 | 2 | 105 | 11.7 | 4.92 |
| Q9H3Z4 | DnaJ homolog subfamily C member 5 OS=Homo sapiens GN=DNAJC5 PE=1 SV=1 - [DNJC5_HUMAN] | 2 | 1 | 2 | 198 | 22.1 | 5.07 |
| Q4VC31 | Coiled-coil domain-containing protein 58 OS=Homo sapiens GN=CCDC58 PE=1 SV=1 - [CCD58_HUMAN] | 2 | 1 | 2 | 144 | 16.6 | 7.81 |
| P51161 | Gastrotropin OS=Homo sapiens GN=FABP6 PE=1 SV=2 - [FABP6_HUMAN] | 2 | 1 | 2 | 128 | 14.4 | 6.8 |
| P61960 | Ubiquitin-fold modifier 1 OS=Homo sapiens GN=UFM1 PE=1 SV=1 - [UFM1_HUMAN] | 2 | 1 | 2 | 85 | 9.1 | 9.31 |
| P09972 | Fructose-bisphosphate aldolase C OS=Homo sapiens GN=ALDOC PE=1 SV=2 - [ALDOC_HUMAN] | 2 | 6 | 10 | 364 | 39.4 | 6.87 |
| Q6S8J3 | POTE ankyrin domain family member E OS=Homo sapiens GN=POTEE PE=1 SV=3 - [POTEE_HUMAN] | 2 | 30 | 49 | 1075 | 121.3 | 6.2 |
| Q8IUE6 | Histone H2A type 2-B OS=Homo sapiens GN=HIST2H2AB PE=1 SV=3 - [H2A2B_HUMAN] | 2 | 5 | 8 | 130 | 14 | 10.89 |
| P26378 | ELAV-like protein 4 OS=Homo sapiens GN=ELAVL4 PE=1 SV=2 - [ELAV4_HUMAN] | 2 | 7 | 11 | 380 | 41.7 | 9.39 |
| P13929 | Beta-enolase OS=Homo sapiens GN=ENO3 PE=1 SV=5 - [ENOB_HUMAN] | 2 | 14 | 21 | 434 | 47 | 7.71 |
| Q96S52 | GPI transamidase component PIG-S OS=Homo sapiens GN=PIGS PE=1 SV=3 - [PIGS_HUMAN] | 2 | 2 | 3 | 555 | 61.6 | 6.49 |
| P09471 | Guanine nucleotide-binding protein G(o) subunit alpha OS=Homo sapiens GN=GNAO1 PE=1 SV=4 - [GNAO_HUMAN] | 2 | 2 | 3 | 354 | 40 | 5.53 |
| P43307 | Translocon-associated protein subunit alpha OS=Homo sapiens GN=SSR1 PE=1 SV=3 - [SSRA_HUMAN] | 2 | 2 | 3 | 286 | 32.2 | 4.49 |
| Q9P000 | COMM domain-containing protein 9 OS=Homo sapiens GN=COMMD9 PE=1 SV=2 - [COMD9_HUMAN] | 2 | 2 | 3 | 198 | 21.8 | 5.88 |
| P20645 | Cation-dependent mannose-6-phosphate receptor OS=Homo sapiens GN=M6PR PE=1 SV=1 - [MPRD_HUMAN] | 2 | 2 | 3 | 277 | 31 | 5.83 |
| P47914 | 60S ribosomal protein L29 OS=Homo sapiens GN=RPL29 PE=1 SV=2 - [RL29_HUMAN] | 2 | 2 | 3 | 159 | 17.7 | 11.66 |
| P42766 | 60S ribosomal protein L35 OS=Homo sapiens GN=RPL35 PE=1 SV=2 - [RL35_HUMAN] | 2 | 2 | 3 | 123 | 14.5 | 11.05 |
| Q9NVI7 | ATPase family AAA domain-containing protein 3A OS=Homo sapiens GN=ATAD3A PE=1 SV=2 - [ATD3A_HUMAN] | 2 | 4 | 5 | 634 | 71.3 | 8.98 |
| Q9UHD9 | Ubiquilin-2 OS=Homo sapiens GN=UBQLN2 PE=1 SV=2 - [UBQL2_HUMAN] | 2 | 4 | 5 | 624 | 65.7 | 5.22 |
| P30154 | Serine/threonine-protein phosphatase 2A 65 kDa regulatory subunit A beta isoform OS=Homo sapiens GN=PPP2R1B PE=1 SV=3 - [2AAB_HUMAN] | 2 | 11 | 13 | 601 | 66.2 | 4.94 |
| P68366 | Tubulin alpha-4A chain OS=Homo sapiens GN=TUBA4A PE=1 SV=1 - [TBA4A_HUMAN] | 2 | 47 | 49 | 448 | 49.9 | 5.06 |
| O60814 | Histone H2B type 1-K OS=Homo sapiens GN=HIST1H2BK PE=1 SV=3 - [H2B1K_HUMAN] | 2 | 10 | 10 | 126 | 13.9 | 10.32 |
| P06899 | Histone H2B type 1-J OS=Homo sapiens GN=HIST1H2BJ PE=1 SV=3 - [H2B1J_HUMAN] | 2 | 9 | 9 | 126 | 13.9 | 10.32 |
| Q16576 | Histone-binding protein RBBP7 OS=Homo sapiens GN=RBBP7 PE=1 SV=1 - [RBBP7_HUMAN] | 2 | 7 | 7 | 425 | 47.8 | 5.05 |
| P60891 | Ribose-phosphate pyrophosphokinase 1 OS=Homo sapiens GN=PRPS1 PE=1 SV=2 - [PRPS1_HUMAN] | 2 | 4 | 4 | 318 | 34.8 | 6.98 |
| P14678 | Small nuclear ribonucleoprotein-associated proteins B and B' OS=Homo sapiens GN=SNRPB PE=1 SV=2 - [RSMB_HUMAN] | 2 | 3 | 3 | 240 | 24.6 | 11.19 |
| Q9H8Y8 | Golgi reassembly-stacking protein 2 OS=Homo sapiens GN=GORASP2 PE=1 SV=3 - [GORS2_HUMAN] | 2 | 3 | 3 | 452 | 47.1 | 4.82 |
| Q9NP79 | Vacuolar protein sorting-associated protein VTA1 homolog OS=Homo sapiens GN=VTA1 PE=1 SV=1 - [VTA1_HUMAN] | 2 | 3 | 3 | 307 | 33.9 | 6.29 |
| P62829 | 60S ribosomal protein L23 OS=Homo sapiens GN=RPL23 PE=1 SV=1 - [RL23_HUMAN] | 2 | 3 | 3 | 140 | 14.9 | 10.51 |
| Q04837 | Single-stranded DNA-binding protein, mitochondrial OS=Homo sapiens GN=SSBP1 PE=1 SV=1 - [SSBP_HUMAN] | 2 | 3 | 3 | 148 | 17.2 | 9.6 |
| P62942 | Peptidyl-prolyl cis-trans isomerase FKBP1A OS=Homo sapiens GN=FKBP1A PE=1 SV=2 - [FKB1A_HUMAN] | 2 | 3 | 3 | 108 | 11.9 | 8.16 |
| P02788 | Lactotransferrin OS=Homo sapiens GN=LTF PE=1 SV=6 - [TRFL_HUMAN] | 2 | 2 | 2 | 710 | 78.1 | 8.12 |
| Q7L2E3 | Putative ATP-dependent RNA helicase DHX30 OS=Homo sapiens GN=DHX30 PE=1 SV=1 - [DHX30_HUMAN] | 2 | 2 | 2 | 1194 | 133.9 | 8.78 |
| Q4V328 | GRIP1-associated protein 1 OS=Homo sapiens GN=GRIPAP1 PE=1 SV=1 - [GRAP1_HUMAN] | 2 | 2 | 2 | 841 | 95.9 | 5.11 |
| Q92990 | Glomulin OS=Homo sapiens GN=GLMN PE=1 SV=2 - [GLMN_HUMAN] | 2 | 2 | 2 | 594 | 68.2 | 5.33 |
| Q9Y262 | Eukaryotic translation initiation factor 3 subunit L OS=Homo sapiens GN=EIF3L PE=1 SV=1 - [EIF3L_HUMAN] | 2 | 2 | 2 | 564 | 66.7 | 6.34 |
| Q9ULX6 | A-kinase anchor protein 8-like OS=Homo sapiens GN=AKAP8L PE=1 SV=3 - [AKP8L_HUMAN] | 2 | 2 | 2 | 646 | 71.6 | 5.07 |
| P22307 | Non-specific lipid-transfer protein OS=Homo sapiens GN=SCP2 PE=1 SV=2 - [NLTP_HUMAN] | 2 | 2 | 2 | 547 | 59 | 6.89 |
| Q7Z739 | YTH domain family protein 3 OS=Homo sapiens GN=YTHDF3 PE=1 SV=1 - [YTHD3_HUMAN] | 2 | 2 | 2 | 585 | 63.8 | 9.04 |
| P56545 | C-terminal-binding protein 2 OS=Homo sapiens GN=CTBP2 PE=1 SV=1 - [CTBP2_HUMAN] | 2 | 2 | 2 | 445 | 48.9 | 6.95 |
| P43694 | Transcription factor GATA-4 OS=Homo sapiens GN=GATA4 PE=1 SV=2 - [GATA4_HUMAN] | 2 | 2 | 2 | 442 | 44.5 | 9.26 |
| Q06124 | Tyrosine-protein phosphatase non-receptor type 11 OS=Homo sapiens GN=PTPN11 PE=1 SV=2 - [PTN11_HUMAN] | 2 | 2 | 2 | 597 | 68.4 | 7.3 |
| Q8N7H5 | RNA polymerase II-associated factor 1 homolog OS=Homo sapiens GN=PAF1 PE=1 SV=2 - [PAF1_HUMAN] | 2 | 2 | 2 | 531 | 59.9 | 4.63 |
| Q6UN15 | Pre-mRNA 3'-end-processing factor FIP1 OS=Homo sapiens GN=FIP1L1 PE=1 SV=1 - [FIP1_HUMAN] | 2 | 2 | 2 | 594 | 66.5 | 5.59 |
| Q99961 | Endophilin-A2 OS=Homo sapiens GN=SH3GL1 PE=1 SV=1 - [SH3G1_HUMAN] | 2 | 2 | 2 | 368 | 41.5 | 5.43 |
| O43670 | BUB3-interacting and GLEBS motif-containing protein ZNF207 OS=Homo sapiens GN=ZNF207 PE=1 SV=1 - [ZN207_HUMAN] | 2 | 2 | 2 | 478 | 50.7 | 9.1 |
| Q9H3P7 | Golgi resident protein GCP60 OS=Homo sapiens GN=ACBD3 PE=1 SV=4 - [GCP60_HUMAN] | 2 | 2 | 2 | 528 | 60.6 | 5.06 |
| P15104 | Glutamine synthetase OS=Homo sapiens GN=GLUL PE=1 SV=4 - [GLNA_HUMAN] | 2 | 2 | 2 | 373 | 42 | 6.89 |
| P49903 | Selenide, water dikinase 1 OS=Homo sapiens GN=SEPHS1 PE=1 SV=2 - [SPS1_HUMAN] | 2 | 2 | 2 | 392 | 42.9 | 5.97 |
| P35270 | Sepiapterin reductase OS=Homo sapiens GN=SPR PE=1 SV=1 - [SPRE_HUMAN] | 2 | 2 | 2 | 261 | 28 | 8.05 |
| Q7LGA3 | Heparan sulfate 2-O-sulfotransferase 1 OS=Homo sapiens GN=HS2ST1 PE=1 SV=1 - [HS2ST_HUMAN] | 2 | 2 | 2 | 356 | 41.9 | 8.69 |
| Q9UI12 | V-type proton ATPase subunit H OS=Homo sapiens GN=ATP6V1H PE=1 SV=1 - [VATH_HUMAN] | 2 | 2 | 2 | 483 | 55.8 | 6.48 |
| Q8WVY7 | Ubiquitin-like domain-containing CTD phosphatase 1 OS=Homo sapiens GN=UBLCP1 PE=1 SV=2 - [UBCP1_HUMAN] | 2 | 2 | 2 | 318 | 36.8 | 6.46 |
| Q9H0R4 | Haloacid dehalogenase-like hydrolase domain-containing protein 2 OS=Homo sapiens GN=HDHD2 PE=1 SV=1 - [HDHD2_HUMAN] | 2 | 2 | 2 | 259 | 28.5 | 6.24 |
| P00387 | NADH-cytochrome b5 reductase 3 OS=Homo sapiens GN=CYB5R3 PE=1 SV=3 - [NB5R3_HUMAN] | 2 | 2 | 2 | 301 | 34.2 | 7.59 |
| Q9BVC6 | Transmembrane protein 109 OS=Homo sapiens GN=TMEM109 PE=1 SV=1 - [TM109_HUMAN] | 2 | 2 | 2 | 243 | 26.2 | 10.48 |
| P62910 | 60S ribosomal protein L32 OS=Homo sapiens GN=RPL32 PE=1 SV=2 - [RL32_HUMAN] | 2 | 2 | 2 | 135 | 15.8 | 11.33 |
| A6NDG6 | Phosphoglycolate phosphatase OS=Homo sapiens GN=PGP PE=1 SV=1 - [PGP_HUMAN] | 2 | 2 | 2 | 321 | 34 | 6.14 |
| Q9P287 | BRCA2 and CDKN1A-interacting protein OS=Homo sapiens GN=BCCIP PE=1 SV=1 - [BCCIP_HUMAN] | 2 | 2 | 2 | 314 | 36 | 4.61 |
| P54709 | Sodium/potassium-transporting ATPase subunit beta-3 OS=Homo sapiens GN=ATP1B3 PE=1 SV=1 - [AT1B3_HUMAN] | 2 | 2 | 2 | 279 | 31.5 | 8.35 |
| O00161 | Synaptosomal-associated protein 23 OS=Homo sapiens GN=SNAP23 PE=1 SV=1 - [SNP23_HUMAN] | 2 | 2 | 2 | 211 | 23.3 | 5.01 |
| P36639 | 7,8-dihydro-8-oxoguanine triphosphatase OS=Homo sapiens GN=NUDT1 PE=1 SV=3 - [8ODP_HUMAN] | 2 | 2 | 2 | 197 | 22.5 | 5.27 |
| P02042 | Hemoglobin subunit delta OS=Homo sapiens GN=HBD PE=1 SV=2 - [HBD_HUMAN] | 2 | 2 | 2 | 147 | 16 | 8.05 |
| Q99598 | Translin-associated protein X OS=Homo sapiens GN=TSNAX PE=1 SV=1 - [TSNAX_HUMAN] | 2 | 2 | 2 | 290 | 33.1 | 6.55 |
| P49006 | MARCKS-related protein OS=Homo sapiens GN=MARCKSL1 PE=1 SV=2 - [MRP_HUMAN] | 2 | 2 | 2 | 195 | 19.5 | 4.67 |
| P04216 | Thy-1 membrane glycoprotein OS=Homo sapiens GN=THY1 PE=1 SV=2 - [THY1_HUMAN] | 2 | 2 | 2 | 161 | 17.9 | 8.73 |
| P46776 | 60S ribosomal protein L27a OS=Homo sapiens GN=RPL27A PE=1 SV=2 - [RL27A_HUMAN] | 2 | 2 | 2 | 148 | 16.6 | 11 |
| P54105 | Methylosome subunit pICln OS=Homo sapiens GN=CLNS1A PE=1 SV=1 - [ICLN_HUMAN] | 2 | 2 | 2 | 237 | 26.2 | 4.11 |
| P14174 | Macrophage migration inhibitory factor OS=Homo sapiens GN=MIF PE=1 SV=4 - [MIF_HUMAN] | 2 | 2 | 2 | 115 | 12.5 | 7.88 |
| P35268 | 60S ribosomal protein L22 OS=Homo sapiens GN=RPL22 PE=1 SV=2 - [RL22_HUMAN] | 2 | 2 | 2 | 128 | 14.8 | 9.19 |
| P55769 | NHP2-like protein 1 OS=Homo sapiens GN=NHP2L1 PE=1 SV=3 - [NH2L1_HUMAN] | 2 | 2 | 2 | 128 | 14.2 | 8.46 |
| Q9Y3U8 | 60S ribosomal protein L36 OS=Homo sapiens GN=RPL36 PE=1 SV=3 - [RL36_HUMAN] | 2 | 2 | 2 | 105 | 12.2 | 11.59 |
| P62310 | U6 snRNA-associated Sm-like protein LSm3 OS=Homo sapiens GN=LSM3 PE=1 SV=2 - [LSM3_HUMAN] | 2 | 2 | 2 | 102 | 11.8 | 4.7 |
| O75964 | ATP synthase subunit g, mitochondrial OS=Homo sapiens GN=ATP5L PE=1 SV=3 - [ATP5L_HUMAN] | 2 | 2 | 2 | 103 | 11.4 | 9.64 |
| P49458 | Signal recognition particle 9 kDa protein OS=Homo sapiens GN=SRP9 PE=1 SV=2 - [SRP09_HUMAN] | 2 | 2 | 2 | 86 | 10.1 | 7.97 |
| P61513 | 60S ribosomal protein L37a OS=Homo sapiens GN=RPL37A PE=1 SV=2 - [RL37A_HUMAN] | 2 | 2 | 2 | 92 | 10.3 | 10.43 |
| Q5VYK3 | Proteasome-associated protein ECM29 homolog OS=Homo sapiens GN=ECM29 PE=1 SV=2 - [ECM29_HUMAN] | 2 | 1 | 1 | 1845 | 204.2 | 7.12 |
| Q5JSZ5 | Protein PRRC2B OS=Homo sapiens GN=PRRC2B PE=1 SV=2 - [PRC2B_HUMAN] | 2 | 1 | 1 | 2229 | 242.8 | 8.34 |
| Q27J81 | Inverted formin-2 OS=Homo sapiens GN=INF2 PE=1 SV=2 - [INF2_HUMAN] | 2 | 1 | 1 | 1249 | 135.5 | 5.38 |
| Q6P2E9 | Enhancer of mRNA-decapping protein 4 OS=Homo sapiens GN=EDC4 PE=1 SV=1 - [EDC4_HUMAN] | 2 | 1 | 1 | 1401 | 151.6 | 5.86 |
| Q9NSE4 | Isoleucyl-tRNA synthetase, mitochondrial OS=Homo sapiens GN=IARS2 PE=1 SV=2 - [SYIM_HUMAN] | 2 | 1 | 1 | 1012 | 113.7 | 7.2 |
| O43264 | Centromere/kinetochore protein zw10 homolog OS=Homo sapiens GN=ZW10 PE=1 SV=3 - [ZW10_HUMAN] | 2 | 1 | 1 | 779 | 88.8 | 6.27 |
| P69849 | Nodal modulator 3 OS=Homo sapiens GN=NOMO3 PE=3 SV=2 - [NOMO3_HUMAN] | 2 | 1 | 1 | 1222 | 134 | 5.67 |
| Q5H9R7 | Serine/threonine-protein phosphatase 6 regulatory subunit 3 OS=Homo sapiens GN=SAPS3 PE=1 SV=2 - [SAPS3_HUMAN] | 2 | 1 | 1 | 873 | 97.6 | 4.6 |
| Q01650 | Large neutral amino acids transporter small subunit 1 OS=Homo sapiens GN=SLC7A5 PE=1 SV=2 - [LAT1_HUMAN] | 2 | 1 | 1 | 507 | 55 | 7.72 |
| Q5VTR2 | E3 ubiquitin-protein ligase BRE1A OS=Homo sapiens GN=RNF20 PE=1 SV=2 - [BRE1A_HUMAN] | 2 | 1 | 1 | 975 | 113.6 | 5.94 |
| Q8N1F7 | Nuclear pore complex protein Nup93 OS=Homo sapiens GN=NUP93 PE=1 SV=2 - [NUP93_HUMAN] | 2 | 1 | 1 | 819 | 93.4 | 5.72 |
| Q13330 | Metastasis-associated protein MTA1 OS=Homo sapiens GN=MTA1 PE=1 SV=2 - [MTA1_HUMAN] | 2 | 1 | 1 | 715 | 80.7 | 9.26 |
| P54289 | Voltage-dependent calcium channel subunit alpha-2/delta-1 OS=Homo sapiens GN=CACNA2D1 PE=1 SV=3 - [CA2D1_HUMAN] | 2 | 1 | 1 | 1103 | 124.5 | 5.27 |
| O43395 | U4/U6 small nuclear ribonucleoprotein Prp3 OS=Homo sapiens GN=PRPF3 PE=1 SV=2 - [PRPF3_HUMAN] | 2 | 1 | 1 | 683 | 77.5 | 9.5 |
| Q9BZI7 | Regulator of nonsense transcripts 3B OS=Homo sapiens GN=UPF3B PE=1 SV=1 - [REN3B_HUMAN] | 2 | 1 | 1 | 483 | 57.7 | 9.48 |
| P23921 | Ribonucleoside-diphosphate reductase large subunit OS=Homo sapiens GN=RRM1 PE=1 SV=1 - [RIR1_HUMAN] | 2 | 1 | 1 | 792 | 90 | 7.15 |
| Q9P2R7 | Succinyl-CoA ligase [ADP-forming] subunit beta, mitochondrial OS=Homo sapiens GN=SUCLA2 PE=1 SV=3 - [SUCB1_HUMAN] | 2 | 1 | 1 | 463 | 50.3 | 7.42 |
| Q9Y5J1 | U3 small nucleolar RNA-associated protein 18 homolog OS=Homo sapiens GN=UTP18 PE=1 SV=3 - [UTP18_HUMAN] | 2 | 1 | 1 | 556 | 62 | 8.76 |
| O00116 | Alkyldihydroxyacetonephosphate synthase, peroxisomal OS=Homo sapiens GN=AGPS PE=1 SV=1 - [ADAS_HUMAN] | 2 | 1 | 1 | 658 | 72.9 | 7.34 |
| P33992 | DNA replication licensing factor MCM5 OS=Homo sapiens GN=MCM5 PE=1 SV=5 - [MCM5_HUMAN] | 2 | 1 | 1 | 734 | 82.2 | 8.37 |
| Q9UPN7 | Serine/threonine-protein phosphatase 6 regulatory subunit 1 OS=Homo sapiens GN=PPP6R1 PE=1 SV=5 - [PP6R1_HUMAN] | 2 | 1 | 1 | 881 | 96.7 | 4.55 |
| Q96ER9 | Coiled-coil domain-containing protein 51 OS=Homo sapiens GN=CCDC51 PE=1 SV=2 - [CCD51_HUMAN] | 2 | 1 | 1 | 411 | 45.8 | 8.19 |
| O94763 | Unconventional prefoldin RPB5 interactor OS=Homo sapiens GN=RMP PE=1 SV=3 - [RMP_HUMAN] | 2 | 1 | 1 | 535 | 59.8 | 5.05 |
| Q96MG7 | Non-structural maintenance of chromosomes element 3 homolog OS=Homo sapiens GN=NSMCE3 PE=1 SV=1 - [NSE3_HUMAN] | 2 | 1 | 1 | 304 | 34.3 | 9.28 |
| Q15438 | Cytohesin-1 OS=Homo sapiens GN=CYTH1 PE=1 SV=1 - [CYH1_HUMAN] | 2 | 1 | 1 | 398 | 46.4 | 5.54 |
| Q9H078 | Caseinolytic peptidase B protein homolog OS=Homo sapiens GN=CLPB PE=1 SV=1 - [CLPB_HUMAN] | 2 | 1 | 1 | 707 | 78.7 | 9.01 |
| Q9P0I2 | Transmembrane protein 111 OS=Homo sapiens GN=TMEM111 PE=1 SV=3 - [TM111_HUMAN] | 2 | 1 | 1 | 261 | 29.9 | 6.81 |
| Q9GZR7 | ATP-dependent RNA helicase DDX24 OS=Homo sapiens GN=DDX24 PE=1 SV=1 - [DDX24_HUMAN] | 2 | 1 | 1 | 859 | 96.3 | 9.06 |
| Q13325 | Interferon-induced protein with tetratricopeptide repeats 5 OS=Homo sapiens GN=IFIT5 PE=1 SV=1 - [IFIT5_HUMAN] | 2 | 1 | 1 | 482 | 55.8 | 7.4 |
| Q9NX58 | Cell growth-regulating nucleolar protein OS=Homo sapiens GN=LYAR PE=1 SV=2 - [LYAR_HUMAN] | 2 | 1 | 1 | 379 | 43.6 | 9.54 |
| Q96HS1 | Serine/threonine-protein phosphatase PGAM5, mitochondrial OS=Homo sapiens GN=PGAM5 PE=1 SV=2 - [PGAM5_HUMAN] | 2 | 1 | 1 | 289 | 32 | 8.68 |
| Q96Q11 | CCA tRNA nucleotidyltransferase 1, mitochondrial OS=Homo sapiens GN=TRNT1 PE=1 SV=2 - [TRNT1_HUMAN] | 2 | 1 | 1 | 434 | 50.1 | 8.1 |
| O00170 | AH receptor-interacting protein OS=Homo sapiens GN=AIP PE=1 SV=2 - [AIP_HUMAN] | 2 | 1 | 1 | 330 | 37.6 | 6.29 |
| Q8NFH5 | Nucleoporin NUP53 OS=Homo sapiens GN=NUP35 PE=1 SV=1 - [NUP53_HUMAN] | 2 | 1 | 1 | 326 | 34.8 | 9.09 |
| Q9BU76 | Multiple myeloma tumor-associated protein 2 OS=Homo sapiens GN=MMTAG2 PE=1 SV=1 - [MMTA2_HUMAN] | 2 | 1 | 1 | 263 | 29.4 | 10.02 |
| Q9UL15 | BAG family molecular chaperone regulator 5 OS=Homo sapiens GN=BAG5 PE=1 SV=1 - [BAG5_HUMAN] | 2 | 1 | 1 | 447 | 51.2 | 6.05 |
| Q9H3Q1 | Cdc42 effector protein 4 OS=Homo sapiens GN=CDC42EP4 PE=1 SV=1 - [BORG4_HUMAN] | 2 | 1 | 1 | 356 | 38 | 5.19 |
| Q96QR8 | Transcriptional activator protein Pur-beta OS=Homo sapiens GN=PURB PE=1 SV=3 - [PURB_HUMAN] | 2 | 1 | 1 | 312 | 33.2 | 5.43 |
| O60762 | Dolichol-phosphate mannosyltransferase OS=Homo sapiens GN=DPM1 PE=1 SV=1 - [DPM1_HUMAN] | 2 | 1 | 1 | 260 | 29.6 | 9.57 |
| O00217 | NADH dehydrogenase [ubiquinone] iron-sulfur protein 8, mitochondrial OS=Homo sapiens GN=NDUFS8 PE=1 SV=1 - [NDUS8_HUMAN] | 2 | 1 | 1 | 210 | 23.7 | 6.34 |
| O43402 | Neighbor of COX4 OS=Homo sapiens GN=COX4NB PE=1 SV=1 - [CX4NB_HUMAN] | 2 | 1 | 1 | 210 | 23.8 | 6.4 |
| Q6P1N9 | Putative deoxyribonuclease TATDN1 OS=Homo sapiens GN=TATDN1 PE=1 SV=2 - [TATD1_HUMAN] | 2 | 1 | 1 | 297 | 33.6 | 6.96 |
| Q9UKD2 | mRNA turnover protein 4 homolog OS=Homo sapiens GN=MRTO4 PE=1 SV=2 - [MRT4_HUMAN] | 2 | 1 | 1 | 239 | 27.5 | 8.29 |
| Q9Y680 | Peptidyl-prolyl cis-trans isomerase FKBP7 OS=Homo sapiens GN=FKBP7 PE=1 SV=1 - [FKBP7_HUMAN] | 2 | 1 | 1 | 259 | 30 | 6.54 |
| Q13630 | GDP-L-fucose synthase OS=Homo sapiens GN=TSTA3 PE=1 SV=1 - [FCL_HUMAN] | 2 | 1 | 1 | 321 | 35.9 | 6.6 |
| A6NDU8 | UPF0600 protein C5orf51 OS=Homo sapiens GN=C5orf51 PE=1 SV=1 - [CE051_HUMAN] | 2 | 1 | 1 | 294 | 33.6 | 5.26 |
| O75446 | Histone deacetylase complex subunit SAP30 OS=Homo sapiens GN=SAP30 PE=1 SV=1 - [SAP30_HUMAN] | 2 | 1 | 1 | 220 | 23.3 | 9.17 |
| Q9Y3D8 | Adenylate kinase isoenzyme 6 OS=Homo sapiens GN=AK6 PE=1 SV=1 - [KAD6_HUMAN] | 2 | 1 | 1 | 172 | 20 | 4.58 |
| Q9Y3C8 | Ubiquitin-fold modifier-conjugating enzyme 1 OS=Homo sapiens GN=UFC1 PE=1 SV=3 - [UFC1_HUMAN] | 2 | 1 | 1 | 167 | 19.4 | 7.4 |
| Q86W42 | THO complex subunit 6 homolog OS=Homo sapiens GN=THOC6 PE=1 SV=1 - [THOC6_HUMAN] | 2 | 1 | 1 | 341 | 37.5 | 7.43 |
| Q9BRG1 | Vacuolar protein-sorting-associated protein 25 OS=Homo sapiens GN=VPS25 PE=1 SV=1 - [VPS25_HUMAN] | 2 | 1 | 1 | 176 | 20.7 | 6.34 |
| O15400 | Syntaxin-7 OS=Homo sapiens GN=STX7 PE=1 SV=4 - [STX7_HUMAN] | 2 | 1 | 1 | 261 | 29.8 | 5.55 |
| Q9NQR4 | Omega-amidase NIT2 OS=Homo sapiens GN=NIT2 PE=1 SV=1 - [NIT2_HUMAN] | 2 | 1 | 1 | 276 | 30.6 | 7.21 |
| Q99614 | Tetratricopeptide repeat protein 1 OS=Homo sapiens GN=TTC1 PE=1 SV=1 - [TTC1_HUMAN] | 2 | 1 | 1 | 292 | 33.5 | 4.84 |
| P00390 | Glutathione reductase, mitochondrial OS=Homo sapiens GN=GSR PE=1 SV=2 - [GSHR_HUMAN] | 2 | 1 | 1 | 522 | 56.2 | 8.5 |
| Q9NV56 | MRG/MORF4L-binding protein OS=Homo sapiens GN=MRGBP PE=1 SV=1 - [MRGBP_HUMAN] | 2 | 1 | 1 | 204 | 22.4 | 5.83 |
| P52434 | DNA-directed RNA polymerases I, II, and III subunit RPABC3 OS=Homo sapiens GN=POLR2H PE=1 SV=4 - [RPAB3_HUMAN] | 2 | 1 | 1 | 150 | 17.1 | 4.68 |
| Q15369 | Transcription elongation factor B polypeptide 1 OS=Homo sapiens GN=TCEB1 PE=1 SV=1 - [ELOC_HUMAN] | 2 | 1 | 1 | 112 | 12.5 | 4.78 |
| O95777 | U6 snRNA-associated Sm-like protein LSm8 OS=Homo sapiens GN=LSM8 PE=1 SV=3 - [LSM8_HUMAN] | 2 | 1 | 1 | 96 | 10.4 | 4.48 |
| O15514 | DNA-directed RNA polymerase II subunit RPB4 OS=Homo sapiens GN=POLR2D PE=1 SV=1 - [RPB4_HUMAN] | 2 | 1 | 1 | 142 | 16.3 | 4.79 |
| Q9Y333 | U6 snRNA-associated Sm-like protein LSm2 OS=Homo sapiens GN=LSM2 PE=1 SV=1 - [LSM2_HUMAN] | 2 | 1 | 1 | 95 | 10.8 | 6.52 |
| P36873 | Serine/threonine-protein phosphatase PP1-gamma catalytic subunit OS=Homo sapiens GN=PPP1CC PE=1 SV=1 - [PP1G_HUMAN] | 2 | 16 | 14 | 323 | 37 | 6.54 |
| P62140 | Serine/threonine-protein phosphatase PP1-beta catalytic subunit OS=Homo sapiens GN=PPP1CB PE=1 SV=3 - [PP1B_HUMAN] | 2 | 14 | 12 | 327 | 37.2 | 6.19 |
| P62136 | Serine/threonine-protein phosphatase PP1-alpha catalytic subunit OS=Homo sapiens GN=PPP1CA PE=1 SV=1 - [PP1A_HUMAN] | 2 | 15 | 12 | 330 | 37.5 | 6.33 |
| P62899 | 60S ribosomal protein L31 OS=Homo sapiens GN=RPL31 PE=1 SV=1 - [RL31_HUMAN] | 2 | 4 | 3 | 125 | 14.5 | 10.54 |
| P18085 | ADP-ribosylation factor 4 OS=Homo sapiens GN=ARF4 PE=1 SV=3 - [ARF4_HUMAN] | 2 | 4 | 3 | 180 | 20.5 | 7.14 |
| P0C0S5 | Histone H2A.Z OS=Homo sapiens GN=H2AFZ PE=1 SV=2 - [H2AZ_HUMAN] | 2 | 6 | 4 | 128 | 13.5 | 10.58 |
| P10253 | Lysosomal alpha-glucosidase OS=Homo sapiens GN=GAA PE=1 SV=4 - [LYAG_HUMAN] | 2 | 3 | 2 | 952 | 105.3 | 6 |
| P09012 | U1 small nuclear ribonucleoprotein A OS=Homo sapiens GN=SNRPA PE=1 SV=3 - [SNRPA_HUMAN] | 2 | 3 | 2 | 282 | 31.3 | 9.83 |
| O14579 | Coatomer subunit epsilon OS=Homo sapiens GN=COPE PE=1 SV=3 - [COPE_HUMAN] | 2 | 3 | 2 | 308 | 34.5 | 5.12 |
| P15153 | Ras-related C3 botulinum toxin substrate 2 OS=Homo sapiens GN=RAC2 PE=1 SV=1 - [RAC2_HUMAN] | 2 | 3 | 2 | 192 | 21.4 | 7.61 |
| P08579 | U2 small nuclear ribonucleoprotein B'' OS=Homo sapiens GN=SNRPB2 PE=1 SV=1 - [RU2B_HUMAN] | 2 | 3 | 2 | 225 | 25.5 | 9.72 |
| Q13243 | Splicing factor, arginine/serine-rich 5 OS=Homo sapiens GN=SFRS5 PE=1 SV=1 - [SFRS5_HUMAN] | 2 | 4 | 2 | 272 | 31.2 | 11.59 |
| P08754 | Guanine nucleotide-binding protein G(k) subunit alpha OS=Homo sapiens GN=GNAI3 PE=1 SV=3 - [GNAI3_HUMAN] | 2 | 4 | 2 | 354 | 40.5 | 5.69 |
| Q5UIP0 | Telomere-associated protein RIF1 OS=Homo sapiens GN=RIF1 PE=1 SV=2 - [RIF1_HUMAN] | 2 | 2 | 1 | 2472 | 274.3 | 5.52 |
| P55265 | Double-stranded RNA-specific adenosine deaminase OS=Homo sapiens GN=ADAR PE=1 SV=4 - [DSRAD_HUMAN] | 2 | 2 | 1 | 1226 | 136 | 8.65 |
| Q9UPT8 | Zinc finger CCCH domain-containing protein 4 OS=Homo sapiens GN=ZC3H4 PE=1 SV=3 - [ZC3H4_HUMAN] | 2 | 2 | 1 | 1303 | 140.2 | 6.27 |
| Q13823 | Nucleolar GTP-binding protein 2 OS=Homo sapiens GN=GNL2 PE=1 SV=1 - [NOG2_HUMAN] | 2 | 2 | 1 | 731 | 83.6 | 9.25 |
| Q9Y3L3 | SH3 domain-binding protein 1 OS=Homo sapiens GN=SH3BP1 PE=1 SV=3 - [3BP1_HUMAN] | 2 | 2 | 1 | 701 | 75.7 | 6.77 |
| P01008 | Antithrombin-III OS=Homo sapiens GN=SERPINC1 PE=1 SV=1 - [ANT3_HUMAN] | 2 | 2 | 1 | 464 | 52.6 | 6.71 |
| Q8WWK9 | Cytoskeleton-associated protein 2 OS=Homo sapiens GN=CKAP2 PE=1 SV=1 - [CKAP2_HUMAN] | 2 | 2 | 1 | 683 | 76.9 | 9.41 |
| Q8WVC0 | RNA polymerase-associated protein LEO1 OS=Homo sapiens GN=LEO1 PE=1 SV=1 - [LEO1_HUMAN] | 2 | 2 | 1 | 666 | 75.4 | 4.51 |
| Q16401 | 26S proteasome non-ATPase regulatory subunit 5 OS=Homo sapiens GN=PSMD5 PE=1 SV=3 - [PSMD5_HUMAN] | 2 | 2 | 1 | 504 | 56.2 | 5.48 |
| Q9BPW8 | Protein NipSnap homolog 1 OS=Homo sapiens GN=NIPSNAP1 PE=1 SV=1 - [NIPS1_HUMAN] | 2 | 2 | 1 | 284 | 33.3 | 9.31 |
| P09960 | Leukotriene A-4 hydrolase OS=Homo sapiens GN=LTA4H PE=1 SV=2 - [LKHA4_HUMAN] | 2 | 2 | 1 | 611 | 69.2 | 6.18 |
| P78406 | mRNA export factor OS=Homo sapiens GN=RAE1 PE=1 SV=1 - [RAE1L_HUMAN] | 2 | 2 | 1 | 368 | 40.9 | 7.83 |
| O14618 | Copper chaperone for superoxide dismutase OS=Homo sapiens GN=CCS PE=1 SV=1 - [CCS_HUMAN] | 2 | 2 | 1 | 274 | 29 | 5.58 |
| Q00535 | Cell division protein kinase 5 OS=Homo sapiens GN=CDK5 PE=1 SV=3 - [CDK5_HUMAN] | 2 | 2 | 1 | 292 | 33.3 | 7.66 |
| Q86XZ4 | Spermatogenesis-associated serine-rich protein 2 OS=Homo sapiens GN=SPATS2 PE=1 SV=1 - [SPAS2_HUMAN] | 2 | 2 | 1 | 545 | 59.5 | 8.9 |
| P19784 | Casein kinase II subunit alpha' OS=Homo sapiens GN=CSNK2A2 PE=1 SV=1 - [CSK22_HUMAN] | 2 | 2 | 1 | 350 | 41.2 | 8.56 |
| P04818 | Thymidylate synthase OS=Homo sapiens GN=TYMS PE=1 SV=3 - [TYSY_HUMAN] | 2 | 2 | 1 | 313 | 35.7 | 7.01 |
| O00487 | 26S proteasome non-ATPase regulatory subunit 14 OS=Homo sapiens GN=PSMD14 PE=1 SV=1 - [PSDE_HUMAN] | 2 | 2 | 1 | 310 | 34.6 | 6.52 |
| P55212 | Caspase-6 OS=Homo sapiens GN=CASP6 PE=1 SV=2 - [CASP6_HUMAN] | 2 | 2 | 1 | 293 | 33.3 | 6.93 |
| P09496 | Clathrin light chain A OS=Homo sapiens GN=CLTA PE=1 SV=1 - [CLCA_HUMAN] | 2 | 2 | 1 | 248 | 27.1 | 4.51 |
| Q15165 | Serum paraoxonase/arylesterase 2 OS=Homo sapiens GN=PON2 PE=1 SV=3 - [PON2_HUMAN] | 2 | 2 | 1 | 354 | 39.4 | 5.6 |
| P11926 | Ornithine decarboxylase OS=Homo sapiens GN=ODC1 PE=1 SV=2 - [DCOR_HUMAN] | 2 | 2 | 1 | 461 | 51.1 | 5.26 |
| O14929 | Histone acetyltransferase type B catalytic subunit OS=Homo sapiens GN=HAT1 PE=1 SV=1 - [HAT1_HUMAN] | 2 | 2 | 1 | 419 | 49.5 | 5.69 |
| P02774 | Vitamin D-binding protein OS=Homo sapiens GN=GC PE=1 SV=1 - [VTDB_HUMAN] | 2 | 2 | 1 | 474 | 52.9 | 5.54 |
| O75367 | Core histone macro-H2A.1 OS=Homo sapiens GN=H2AFY PE=1 SV=4 - [H2AY_HUMAN] | 2 | 2 | 1 | 372 | 39.6 | 9.79 |
| Q9UIV1 | CCR4-NOT transcription complex subunit 7 OS=Homo sapiens GN=CNOT7 PE=1 SV=3 - [CNOT7_HUMAN] | 2 | 2 | 1 | 285 | 32.7 | 4.84 |
| O95721 | Synaptosomal-associated protein 29 OS=Homo sapiens GN=SNAP29 PE=1 SV=1 - [SNP29_HUMAN] | 2 | 2 | 1 | 258 | 29 | 5.81 |
| P52564 | Dual specificity mitogen-activated protein kinase kinase 6 OS=Homo sapiens GN=MAP2K6 PE=1 SV=1 - [MP2K6_HUMAN] | 2 | 2 | 1 | 334 | 37.5 | 7.39 |
| P43897 | Elongation factor Ts, mitochondrial OS=Homo sapiens GN=TSFM PE=1 SV=2 - [EFTS_HUMAN] | 2 | 2 | 1 | 325 | 35.4 | 8.38 |
| P22087 | rRNA 2'-O-methyltransferase fibrillarin OS=Homo sapiens GN=FBL PE=1 SV=2 - [FBRL_HUMAN] | 2 | 2 | 1 | 321 | 33.8 | 10.18 |
| Q16543 | Hsp90 co-chaperone Cdc37 OS=Homo sapiens GN=CDC37 PE=1 SV=1 - [CDC37_HUMAN] | 2 | 2 | 1 | 378 | 44.4 | 5.25 |
| O75792 | Ribonuclease H2 subunit A OS=Homo sapiens GN=RNASEH2A PE=1 SV=2 - [RNH2A_HUMAN] | 2 | 2 | 1 | 299 | 33.4 | 5.25 |
| P61009 | Signal peptidase complex subunit 3 OS=Homo sapiens GN=SPCS3 PE=1 SV=1 - [SPCS3_HUMAN] | 2 | 2 | 1 | 180 | 20.3 | 8.62 |
| P13073 | Cytochrome c oxidase subunit 4 isoform 1, mitochondrial OS=Homo sapiens GN=COX4I1 PE=1 SV=1 - [COX41_HUMAN] | 2 | 2 | 1 | 169 | 19.6 | 9.51 |
| P11233 | Ras-related protein Ral-A OS=Homo sapiens GN=RALA PE=1 SV=1 - [RALA_HUMAN] | 2 | 2 | 1 | 206 | 23.6 | 7.11 |
| P46778 | 60S ribosomal protein L21 OS=Homo sapiens GN=RPL21 PE=1 SV=2 - [RL21_HUMAN] | 2 | 2 | 1 | 160 | 18.6 | 10.49 |
| P18077 | 60S ribosomal protein L35a OS=Homo sapiens GN=RPL35A PE=1 SV=2 - [RL35A_HUMAN] | 2 | 2 | 1 | 110 | 12.5 | 11.06 |
| Q8TEA8 | D-tyrosyl-tRNA(Tyr) deacylase 1 OS=Homo sapiens GN=DTD1 PE=1 SV=2 - [DTD1_HUMAN] | 2 | 2 | 1 | 209 | 23.4 | 8.24 |
| Q96EY4 | Translation machinery-associated protein 16 OS=Homo sapiens GN=TMA16 PE=1 SV=2 - [TMA16_HUMAN] | 2 | 2 | 1 | 203 | 23.8 | 9.26 |
| Q9BZL1 | Ubiquitin-like protein 5 OS=Homo sapiens GN=UBL5 PE=1 SV=1 - [UBL5_HUMAN] | 2 | 2 | 1 | 73 | 8.5 | 8.44 |
| P83881 | 60S ribosomal protein L36a OS=Homo sapiens GN=RPL36A PE=1 SV=2 - [RL36A_HUMAN] | 2 | 2 | 1 | 106 | 12.4 | 10.58 |
| P63167 | Dynein light chain 1, cytoplasmic OS=Homo sapiens GN=DYNLL1 PE=1 SV=1 - [DYL1_HUMAN] | 2 | 2 | 1 | 89 | 10.4 | 7.4 |
| Q9UBI6 | Guanine nucleotide-binding protein G(I)/G(S)/G(O) subunit gamma-12 OS=Homo sapiens GN=GNG12 PE=1 SV=3 - [GBG12_HUMAN] | 2 | 2 | 1 | 72 | 8 | 8.97 |
| P57740 | Nuclear pore complex protein Nup107 OS=Homo sapiens GN=NUP107 PE=1 SV=1 - [NU107_HUMAN] | 2 | 3 | 1 | 925 | 106.3 | 5.43 |
| P62244 | 40S ribosomal protein S15a OS=Homo sapiens GN=RPS15A PE=1 SV=2 - [RS15A_HUMAN] | 2 | 3 | 0 | 130 | 14.8 | 10.13 |
| P01024 | Complement C3 OS=Homo sapiens GN=C3 PE=1 SV=2 - [CO3_HUMAN] | 2 | 2 | 0 | 1663 | 187 | 6.4 |
| P51532 | Transcription activator BRG1 OS=Homo sapiens GN=SMARCA4 PE=1 SV=2 - [SMCA4_HUMAN] | 2 | 2 | 0 | 1647 | 184.5 | 7.88 |
| Q86YZ3 | Hornerin OS=Homo sapiens GN=HRNR PE=1 SV=2 - [HORN_HUMAN] | 2 | 2 | 0 | 2850 | 282.2 | 10.04 |
| O95197 | Reticulon-3 OS=Homo sapiens GN=RTN3 PE=1 SV=2 - [RTN3_HUMAN] | 2 | 2 | 0 | 1032 | 112.5 | 4.96 |
| P48634 | Protein PRRC2A OS=Homo sapiens GN=PRRC2A PE=1 SV=3 - [PRC2A_HUMAN] | 2 | 2 | 0 | 2157 | 228.7 | 9.45 |
| P53618 | Coatomer subunit beta OS=Homo sapiens GN=COPB1 PE=1 SV=3 - [COPB_HUMAN] | 2 | 2 | 0 | 953 | 107.1 | 6.05 |
| Q8WTV0 | Scavenger receptor class B member 1 OS=Homo sapiens GN=SCARB1 PE=1 SV=1 - [SCRB1_HUMAN] | 2 | 2 | 0 | 552 | 60.8 | 8.24 |
| O95219 | Sorting nexin-4 OS=Homo sapiens GN=SNX4 PE=1 SV=1 - [SNX4_HUMAN] | 2 | 2 | 0 | 450 | 51.9 | 5.99 |
| O15460 | Prolyl 4-hydroxylase subunit alpha-2 OS=Homo sapiens GN=P4HA2 PE=1 SV=1 - [P4HA2_HUMAN] | 2 | 2 | 0 | 535 | 60.9 | 5.71 |
| P82650 | 28S ribosomal protein S22, mitochondrial OS=Homo sapiens GN=MRPS22 PE=1 SV=1 - [RT22_HUMAN] | 2 | 2 | 0 | 360 | 41.3 | 7.9 |
| O15372 | Eukaryotic translation initiation factor 3 subunit H OS=Homo sapiens GN=EIF3H PE=1 SV=1 - [EIF3H_HUMAN] | 2 | 2 | 0 | 352 | 39.9 | 6.54 |
| O96008 | Mitochondrial import receptor subunit TOM40 homolog OS=Homo sapiens GN=TOMM40 PE=1 SV=1 - [TOM40_HUMAN] | 2 | 2 | 0 | 361 | 37.9 | 7.25 |
| O43617 | Trafficking protein particle complex subunit 3 OS=Homo sapiens GN=TRAPPC3 PE=1 SV=1 - [TPPC3_HUMAN] | 2 | 2 | 0 | 180 | 20.3 | 4.96 |

**Supplementary Table 2:** A full list of proteins common detected in *mock* and *hMT3* overexpressed in UKF-NB-4 cell line. Out of total 1481 common protein in two datasets, 438 were up-regulated (fold ratio > 1.5) and 32 were downregulated (fold ratio < 0.5).

| Accession | Description | ΣUnique Peptides | PSM | PSM | Fold Ratio |
| --- | --- | --- | --- | --- | --- |
|  |  |  | UKF-NB-4 | UKF-NB-4 |  |
|  |  |  | *Mock* | *hMT3* |  |
| Q7L0Y3 | Mitochondrial ribonuclease P protein 1 OS=Homo sapiens GN=RG9MTD1 PE=1 SV=2 - [MRRP1_HUMAN] | 7 | 1 | 7 | 7 |
| Q13907 | Isopentenyl-diphosphate Delta-isomerase 1 OS=Homo sapiens GN=IDI1 PE=1 SV=2 - [IDI1_HUMAN] | 5 | 1 | 7 | 7 |
| P25713 | Metallothionein-3, Growth inhibitory factor, OS=Homo sapiens GN=MT3 PE=1 SV=2 - [MT3_HUMAN] | 5 | 1 | 7 | 7 |
| Q9UBQ5 | Eukaryotic translation initiation factor 3 subunit K OS=Homo sapiens GN=EIF3K PE=1 SV=1 - [EIF3K_HUMAN] | 5 | 1 | 7 | 7 |
| Q9Y520 | Protein PRRC2C OS=Homo sapiens GN=PRRC2C PE=1 SV=4 - [PRC2C_HUMAN] | 6 | 1 | 6 | 6 |
| O00451 | GDNF family receptor alpha-2 OS=Homo sapiens GN=GFRA2 PE=2 SV=2 - [GFRA2_HUMAN] | 6 | 1 | 6 | 6 |
| Q9BVJ6 | U3 small nucleolar RNA-associated protein 14 homolog A OS=Homo sapiens GN=UTP14A PE=1 SV=1 - [UT14A_HUMAN] | 6 | 1 | 6 | 6 |
| Q8IWX8 | Calcium homeostasis endoplasmic reticulum protein OS=Homo sapiens GN=CHERP PE=1 SV=3 - [CHERP_HUMAN] | 6 | 1 | 6 | 6 |
| P21283 | V-type proton ATPase subunit C 1 OS=Homo sapiens GN=ATP6V1C1 PE=1 SV=4 - [VATC1_HUMAN] | 6 | 1 | 6 | 6 |
| Q96C86 | m7GpppX diphosphatase OS=Homo sapiens GN=DCPS PE=1 SV=2 - [DCPS_HUMAN] | 5 | 1 | 6 | 6 |
| O94903 | Proline synthetase co-transcribed bacterial homolog protein OS=Homo sapiens GN=PROSC PE=1 SV=1 - [PROSC_HUMAN] | 5 | 1 | 6 | 6 |
| Q96TA2 | ATP-dependent metalloprotease YME1L1 OS=Homo sapiens GN=YME1L1 PE=1 SV=2 - [YMEL1_HUMAN] | 4 | 1 | 6 | 6 |
| P49755 | Transmembrane emp24 domain-containing protein 10 OS=Homo sapiens GN=TMED10 PE=1 SV=2 - [TMEDA_HUMAN] | 4 | 1 | 6 | 6 |
| P08574 | Cytochrome c1, heme protein, mitochondrial OS=Homo sapiens GN=CYC1 PE=1 SV=3 - [CY1_HUMAN] | 3 | 1 | 6 | 6 |
| P51665 | 26S proteasome non-ATPase regulatory subunit 7 OS=Homo sapiens GN=PSMD7 PE=1 SV=2 - [PSD7_HUMAN] | 8 | 2 | 10 | 5 |
| P27635 | 60S ribosomal protein L10 OS=Homo sapiens GN=RPL10 PE=1 SV=4 - [RL10_HUMAN] | 6 | 1 | 5 | 5 |
| Q5QJE6 | Deoxynucleotidyltransferase terminal-interacting protein 2 OS=Homo sapiens GN=DNTTIP2 PE=1 SV=2 - [TDIF2_HUMAN] | 5 | 1 | 5 | 5 |
| Q9BYT8 | Neurolysin, mitochondrial OS=Homo sapiens GN=NLN PE=1 SV=1 - [NEUL_HUMAN] | 5 | 1 | 5 | 5 |
| Q13451 | Peptidyl-prolyl cis-trans isomerase FKBP5 OS=Homo sapiens GN=FKBP5 PE=1 SV=2 - [FKBP5_HUMAN] | 5 | 1 | 5 | 5 |
| P55010 | Eukaryotic translation initiation factor 5 OS=Homo sapiens GN=EIF5 PE=1 SV=2 - [IF5_HUMAN] | 5 | 1 | 5 | 5 |
| Q9H2W6 | 39S ribosomal protein L46, mitochondrial OS=Homo sapiens GN=MRPL46 PE=1 SV=1 - [RM46_HUMAN] | 5 | 1 | 5 | 5 |
| P35269 | General transcription factor IIF subunit 1 OS=Homo sapiens GN=GTF2F1 PE=1 SV=2 - [T2FA_HUMAN] | 5 | 1 | 5 | 5 |
| Q8WZA9 | Immunity-related GTPase family Q protein OS=Homo sapiens GN=IRGQ PE=1 SV=1 - [IRGQ_HUMAN] | 5 | 1 | 5 | 5 |
| P49721 | Proteasome subunit beta type-2 OS=Homo sapiens GN=PSMB2 PE=1 SV=1 - [PSB2_HUMAN] | 5 | 1 | 5 | 5 |
| Q9BSD7 | Nucleoside-triphosphatase C1orf57 OS=Homo sapiens GN=C1orf57 PE=1 SV=1 - [CA057_HUMAN] | 5 | 1 | 5 | 5 |
| Q6IAA8 | RhoA activator C11orf59 OS=Homo sapiens GN=C11orf59 PE=1 SV=2 - [CK059_HUMAN] | 5 | 1 | 5 | 5 |
| Q14978 | Nucleolar and coiled-body phosphoprotein 1 OS=Homo sapiens GN=NOLC1 PE=1 SV=2 - [NOLC1_HUMAN] | 4 | 1 | 5 | 5 |
| O75475 | PC4 and SFRS1-interacting protein OS=Homo sapiens GN=PSIP1 PE=1 SV=1 - [PSIP1_HUMAN] | 4 | 1 | 5 | 5 |
| Q9Y295 | Developmentally-regulated GTP-binding protein 1 OS=Homo sapiens GN=DRG1 PE=1 SV=1 - [DRG1_HUMAN] | 4 | 1 | 5 | 5 |
| Q15435 | Protein phosphatase 1 regulatory subunit 7 OS=Homo sapiens GN=PPP1R7 PE=1 SV=1 - [PP1R7_HUMAN] | 4 | 1 | 5 | 5 |
| Q99986 | Serine/threonine-protein kinase VRK1 OS=Homo sapiens GN=VRK1 PE=1 SV=1 - [VRK1_HUMAN] | 4 | 1 | 5 | 5 |
| Q9BVK6 | Transmembrane emp24 domain-containing protein 9 OS=Homo sapiens GN=TMED9 PE=1 SV=2 - [TMED9_HUMAN] | 4 | 1 | 5 | 5 |
| P45973 | Chromobox protein homolog 5 OS=Homo sapiens GN=CBX5 PE=1 SV=1 - [CBX5_HUMAN] | 4 | 1 | 5 | 5 |
| P16455 | Methylated-DNA--protein-cysteine methyltransferase OS=Homo sapiens GN=MGMT PE=1 SV=1 - [MGMT_HUMAN] | 4 | 1 | 5 | 5 |
| Q8NHH9 | Atlastin-2 OS=Homo sapiens GN=ATL2 PE=1 SV=2 - [ATLA2_HUMAN] | 3 | 1 | 5 | 5 |
| Q9BY32 | Inosine triphosphate pyrophosphatase OS=Homo sapiens GN=ITPA PE=1 SV=2 - [ITPA_HUMAN] | 3 | 1 | 5 | 5 |
| O15042 | U2-associated protein SR140 OS=Homo sapiens GN=SR140 PE=1 SV=2 - [SR140_HUMAN] | 9 | 2 | 8 | 4 |
| P43487 | Ran-specific GTPase-activating protein OS=Homo sapiens GN=RANBP1 PE=1 SV=1 - [RANG_HUMAN] | 5 | 2 | 8 | 4 |
| Q15021 | Condensin complex subunit 1 OS=Homo sapiens GN=NCAPD2 PE=1 SV=3 - [CND1_HUMAN] | 5 | 1 | 4 | 4 |
| O95793 | Double-stranded RNA-binding protein Staufen homolog 1 OS=Homo sapiens GN=STAU1 PE=1 SV=2 - [STAU1_HUMAN] | 5 | 1 | 4 | 4 |
| P12109 | Collagen alpha-1(VI) chain OS=Homo sapiens GN=COL6A1 PE=1 SV=3 - [CO6A1_HUMAN] | 5 | 1 | 4 | 4 |
| Q9Y5X3 | Sorting nexin-5 OS=Homo sapiens GN=SNX5 PE=1 SV=1 - [SNX5_HUMAN] | 5 | 1 | 4 | 4 |
| Q15813 | Tubulin-specific chaperone E OS=Homo sapiens GN=TBCE PE=1 SV=1 - [TBCE_HUMAN] | 4 | 1 | 4 | 4 |
| Q8WYA6 | Beta-catenin-like protein 1 OS=Homo sapiens GN=CTNNBL1 PE=1 SV=1 - [CTBL1_HUMAN] | 4 | 1 | 4 | 4 |
| Q96JB5 | CDK5 regulatory subunit-associated protein 3 OS=Homo sapiens GN=CDK5RAP3 PE=1 SV=2 - [CK5P3_HUMAN] | 4 | 1 | 4 | 4 |
| O00567 | Nucleolar protein 56 OS=Homo sapiens GN=NOP56 PE=1 SV=4 - [NOP56_HUMAN] | 4 | 1 | 4 | 4 |
| Q01844 | RNA-binding protein EWS OS=Homo sapiens GN=EWSR1 PE=1 SV=1 - [EWS_HUMAN] | 4 | 1 | 4 | 4 |
| Q14677 | Clathrin interactor 1 OS=Homo sapiens GN=CLINT1 PE=1 SV=1 - [EPN4_HUMAN] | 4 | 1 | 4 | 4 |
| Q16836 | Hydroxyacyl-coenzyme A dehydrogenase, mitochondrial OS=Homo sapiens GN=HADH PE=1 SV=3 - [HCDH_HUMAN] | 4 | 1 | 4 | 4 |
| O95218 | Zinc finger Ran-binding domain-containing protein 2 OS=Homo sapiens GN=ZRANB2 PE=1 SV=2 - [ZRAB2_HUMAN] | 4 | 1 | 4 | 4 |
| Q96GM5 | SWI/SNF-related matrix-associated actin-dependent regulator of chromatin subfamily D member 1 OS=Homo sapiens GN=SMARCD1 PE=1 SV=2 - [SMRD1_HUMAN] | 4 | 1 | 4 | 4 |
| Q7Z5L9 | Interferon regulatory factor 2-binding protein 2 OS=Homo sapiens GN=IRF2BP2 PE=1 SV=2 - [I2BP2_HUMAN] | 4 | 1 | 4 | 4 |
| P40222 | Alpha-taxilin OS=Homo sapiens GN=TXLNA PE=1 SV=3 - [TXLNA_HUMAN] | 4 | 1 | 4 | 4 |
| P23258 | Tubulin gamma-1 chain OS=Homo sapiens GN=TUBG1 PE=1 SV=2 - [TBG1_HUMAN] | 4 | 1 | 4 | 4 |
| Q96DH6 | RNA-binding protein Musashi homolog 2 OS=Homo sapiens GN=MSI2 PE=1 SV=1 - [MSI2H_HUMAN] | 4 | 1 | 4 | 4 |
| P54920 | Alpha-soluble NSF attachment protein OS=Homo sapiens GN=NAPA PE=1 SV=3 - [SNAA_HUMAN] | 4 | 1 | 4 | 4 |
| P28074 | Proteasome subunit beta type-5 OS=Homo sapiens GN=PSMB5 PE=1 SV=3 - [PSB5_HUMAN] | 4 | 1 | 4 | 4 |
| P53701 | Cytochrome c-type heme lyase OS=Homo sapiens GN=HCCS PE=1 SV=1 - [CCHL_HUMAN] | 4 | 1 | 4 | 4 |
| P55957 | BH3-interacting domain death agonist OS=Homo sapiens GN=BID PE=1 SV=1 - [BID_HUMAN] | 4 | 1 | 4 | 4 |
| Q07020 | 60S ribosomal protein L18 OS=Homo sapiens GN=RPL18 PE=1 SV=2 - [RL18_HUMAN] | 4 | 1 | 4 | 4 |
| Q3MHD2 | Protein LSM12 homolog OS=Homo sapiens GN=LSM12 PE=1 SV=2 - [LSM12_HUMAN] | 4 | 1 | 4 | 4 |
| Q13442 | 28 kDa heat- and acid-stable phosphoprotein OS=Homo sapiens GN=PDAP1 PE=1 SV=1 - [HAP28_HUMAN] | 4 | 1 | 4 | 4 |
| Q16850 | Lanosterol 14-alpha demethylase OS=Homo sapiens GN=CYP51A1 PE=1 SV=3 - [CP51A_HUMAN] | 3 | 1 | 4 | 4 |
| O75439 | Mitochondrial-processing peptidase subunit beta OS=Homo sapiens GN=PMPCB PE=1 SV=2 - [MPPB_HUMAN] | 3 | 1 | 4 | 4 |
| P82930 | 28S ribosomal protein S34, mitochondrial OS=Homo sapiens GN=MRPS34 PE=1 SV=2 - [RT34_HUMAN] | 3 | 1 | 4 | 4 |
| P04179 | Superoxide dismutase [Mn], mitochondrial OS=Homo sapiens GN=SOD2 PE=1 SV=2 - [SODM_HUMAN] | 3 | 1 | 4 | 4 |
| P51571 | Translocon-associated protein subunit delta OS=Homo sapiens GN=SSR4 PE=1 SV=1 - [SSRD_HUMAN] | 3 | 1 | 4 | 4 |
| P06730 | Eukaryotic translation initiation factor 4E OS=Homo sapiens GN=EIF4E PE=1 SV=2 - [IF4E_HUMAN] | 3 | 1 | 4 | 4 |
| P13693 | Translationally-controlled tumor protein OS=Homo sapiens GN=TPT1 PE=1 SV=1 - [TCTP_HUMAN] | 3 | 1 | 4 | 4 |
| P63279 | SUMO-conjugating enzyme UBC9 OS=Homo sapiens GN=UBE2I PE=1 SV=1 - [UBC9_HUMAN] | 3 | 1 | 4 | 4 |
| O43663 | Protein regulator of cytokinesis 1 OS=Homo sapiens GN=PRC1 PE=1 SV=2 - [PRC1_HUMAN] | 2 | 1 | 4 | 4 |
| Q99627 | COP9 signalosome complex subunit 8 OS=Homo sapiens GN=COPS8 PE=1 SV=1 - [CSN8_HUMAN] | 2 | 1 | 4 | 4 |
| Q9UIG0 | Tyrosine-protein kinase BAZ1B OS=Homo sapiens GN=BAZ1B PE=1 SV=2 - [BAZ1B_HUMAN] | 12 | 3 | 11 | 3.6666667 |
| Q16352 | Alpha-internexin OS=Homo sapiens GN=INA PE=1 SV=2 - [AINX_HUMAN] | 10 | 3 | 11 | 3.6666667 |
| P52788 | Spermine synthase OS=Homo sapiens GN=SMS PE=1 SV=2 - [SPSY_HUMAN] | 10 | 3 | 11 | 3.6666667 |
| O76003 | Glutaredoxin-3 OS=Homo sapiens GN=GLRX3 PE=1 SV=2 - [GLRX3_HUMAN] | 9 | 3 | 11 | 3.6666667 |
| Q9Y224 | UPF0568 protein C14orf166 OS=Homo sapiens GN=C14orf166 PE=1 SV=1 - [CN166_HUMAN] | 8 | 3 | 11 | 3.6666667 |
| O60610 | Protein diaphanous homolog 1 OS=Homo sapiens GN=DIAPH1 PE=1 SV=2 - [DIAP1_HUMAN] | 8 | 2 | 7 | 3.5 |
| O76094 | Signal recognition particle 72 kDa protein OS=Homo sapiens GN=SRP72 PE=1 SV=3 - [SRP72_HUMAN] | 7 | 2 | 7 | 3.5 |
| Q8WU90 | Zinc finger CCCH domain-containing protein 15 OS=Homo sapiens GN=ZC3H15 PE=1 SV=1 - [ZC3HF_HUMAN] | 7 | 2 | 7 | 3.5 |
| Q9BY77 | Polymerase delta-interacting protein 3 OS=Homo sapiens GN=POLDIP3 PE=1 SV=2 - [PDIP3_HUMAN] | 7 | 2 | 7 | 3.5 |
| Q9UQ35 | Serine/arginine repetitive matrix protein 2 OS=Homo sapiens GN=SRRM2 PE=1 SV=2 - [SRRM2_HUMAN] | 6 | 2 | 7 | 3.5 |
| Q9UKV3 | Apoptotic chromatin condensation inducer in the nucleus OS=Homo sapiens GN=ACIN1 PE=1 SV=2 - [ACINU_HUMAN] | 6 | 2 | 7 | 3.5 |
| P30520 | Adenylosuccinate synthetase isozyme 2 OS=Homo sapiens GN=ADSS PE=1 SV=3 - [PURA2_HUMAN] | 6 | 2 | 7 | 3.5 |
| P62633 | Cellular nucleic acid-binding protein OS=Homo sapiens GN=CNBP PE=1 SV=1 - [CNBP_HUMAN] | 5 | 2 | 7 | 3.5 |
| P54819 | Adenylate kinase 2, mitochondrial OS=Homo sapiens GN=AK2 PE=1 SV=2 - [KAD2_HUMAN] | 8 | 3 | 10 | 3.3333333 |
| P28331 | NADH-ubiquinone oxidoreductase 75 kDa subunit, mitochondrial OS=Homo sapiens GN=NDUFS1 PE=1 SV=3 - [NDUS1_HUMAN] | 11 | 4 | 13 | 3.25 |
| P33991 | DNA replication licensing factor MCM4 OS=Homo sapiens GN=MCM4 PE=1 SV=5 - [MCM4_HUMAN] | 18 | 6 | 19 | 3.1666667 |
| P48047 | ATP synthase subunit O, mitochondrial OS=Homo sapiens GN=ATP5O PE=1 SV=1 - [ATPO_HUMAN] | 7 | 3 | 9 | 3 |
| P20290 | Transcription factor BTF3 OS=Homo sapiens GN=BTF3 PE=1 SV=1 - [BTF3_HUMAN] | 5 | 3 | 9 | 3 |
| O75533 | Splicing factor 3B subunit 1 OS=Homo sapiens GN=SF3B1 PE=1 SV=3 - [SF3B1_HUMAN] | 8 | 2 | 6 | 3 |
| O14497 | AT-rich interactive domain-containing protein 1A OS=Homo sapiens GN=ARID1A PE=1 SV=3 - [ARI1A_HUMAN] | 7 | 2 | 6 | 3 |
| Q9NWY4 | Histone PARylation factor 1 OS=Homo sapiens GN=HPF1 PE=1 SV=2 - [HPF1_HUMAN] | 7 | 2 | 6 | 3 |
| Q8NE71 | ATP-binding cassette sub-family F member 1 OS=Homo sapiens GN=ABCF1 PE=1 SV=2 - [ABCF1_HUMAN] | 6 | 2 | 6 | 3 |
| P53634 | Dipeptidyl peptidase 1 OS=Homo sapiens GN=CTSC PE=1 SV=2 - [CATC_HUMAN] | 6 | 2 | 6 | 3 |
| O00264 | Membrane-associated progesterone receptor component 1 OS=Homo sapiens GN=PGRMC1 PE=1 SV=3 - [PGRC1_HUMAN] | 6 | 2 | 6 | 3 |
| Q13596 | Sorting nexin-1 OS=Homo sapiens GN=SNX1 PE=1 SV=3 - [SNX1_HUMAN] | 5 | 2 | 6 | 3 |
| Q92522 | Histone H1x OS=Homo sapiens GN=H1FX PE=1 SV=1 - [H1X_HUMAN] | 5 | 2 | 6 | 3 |
| Q8IY81 | pre-rRNA processing protein FTSJ3 OS=Homo sapiens GN=FTSJ3 PE=1 SV=2 - [SPB1_HUMAN] | 4 | 2 | 6 | 3 |
| P30837 | Aldehyde dehydrogenase X, mitochondrial OS=Homo sapiens GN=ALDH1B1 PE=1 SV=3 - [AL1B1_HUMAN] | 4 | 2 | 6 | 3 |
| Q9UMX0 | Ubiquilin-1 OS=Homo sapiens GN=UBQLN1 PE=1 SV=2 - [UBQL1_HUMAN] | 2 | 2 | 6 | 3 |
| Q9Y4W6 | AFG3-like protein 2 OS=Homo sapiens GN=AFG3L2 PE=1 SV=2 - [AFG32_HUMAN] | 4 | 1 | 3 | 3 |
| P35221 | Catenin alpha-1 OS=Homo sapiens GN=CTNNA1 PE=1 SV=1 - [CTNA1_HUMAN] | 4 | 1 | 3 | 3 |
| Q6PJT7 | Zinc finger CCCH domain-containing protein 14 OS=Homo sapiens GN=ZC3H14 PE=1 SV=1 - [ZC3HE_HUMAN] | 4 | 1 | 3 | 3 |
| Q86VS8 | Protein Hook homolog 3 OS=Homo sapiens GN=HOOK3 PE=1 SV=2 - [HOOK3_HUMAN] | 4 | 1 | 3 | 3 |
| Q99816 | Tumor susceptibility gene 101 protein OS=Homo sapiens GN=TSG101 PE=1 SV=2 - [TS101_HUMAN] | 4 | 1 | 3 | 3 |
| Q16795 | NADH dehydrogenase [ubiquinone] 1 alpha subcomplex subunit 9, mitochondrial OS=Homo sapiens GN=NDUFA9 PE=1 SV=2 - [NDUA9_HUMAN] | 4 | 1 | 3 | 3 |
| O95400 | CD2 antigen cytoplasmic tail-binding protein 2 OS=Homo sapiens GN=CD2BP2 PE=1 SV=1 - [CD2B2_HUMAN] | 4 | 1 | 3 | 3 |
| Q15014 | Mortality factor 4-like protein 2 OS=Homo sapiens GN=MORF4L2 PE=1 SV=1 - [MO4L2_HUMAN] | 4 | 1 | 3 | 3 |
| Q9BRX5 | DNA replication complex GINS protein PSF3 OS=Homo sapiens GN=GINS3 PE=1 SV=1 - [PSF3_HUMAN] | 4 | 1 | 3 | 3 |
| Q3V6T2 | Girdin OS=Homo sapiens GN=CCDC88A PE=1 SV=2 - [GRDN_HUMAN] | 3 | 1 | 3 | 3 |
| Q7Z6Z7 | E3 ubiquitin-protein ligase HUWE1 OS=Homo sapiens GN=HUWE1 PE=1 SV=3 - [HUWE1_HUMAN] | 3 | 1 | 3 | 3 |
| P35658 | Nuclear pore complex protein Nup214 OS=Homo sapiens GN=NUP214 PE=1 SV=2 - [NU214_HUMAN] | 3 | 1 | 3 | 3 |
| P08069 | Insulin-like growth factor 1 receptor OS=Homo sapiens GN=IGF1R PE=1 SV=1 - [IGF1R_HUMAN] | 3 | 1 | 3 | 3 |
| P52789 | Hexokinase-2 OS=Homo sapiens GN=HK2 PE=1 SV=2 - [HXK2_HUMAN] | 3 | 1 | 3 | 3 |
| Q9UMS6 | Synaptopodin-2 OS=Homo sapiens GN=SYNPO2 PE=1 SV=2 - [SYNP2_HUMAN] | 3 | 1 | 3 | 3 |
| P51610 | Host cell factor 1 OS=Homo sapiens GN=HCFC1 PE=1 SV=2 - [HCFC1_HUMAN] | 3 | 1 | 3 | 3 |
| P31040 | Succinate dehydrogenase [ubiquinone] flavoprotein subunit, mitochondrial OS=Homo sapiens GN=SDHA PE=1 SV=2 - [DHSA_HUMAN] | 3 | 1 | 3 | 3 |
| Q32MZ4 | Leucine-rich repeat flightless-interacting protein 1 OS=Homo sapiens GN=LRRFIP1 PE=1 SV=2 - [LRRF1_HUMAN] | 3 | 1 | 3 | 3 |
| Q96DV4 | 39S ribosomal protein L38, mitochondrial OS=Homo sapiens GN=MRPL38 PE=1 SV=2 - [RM38_HUMAN] | 3 | 1 | 3 | 3 |
| P06132 | Uroporphyrinogen decarboxylase OS=Homo sapiens GN=UROD PE=1 SV=2 - [DCUP_HUMAN] | 3 | 1 | 3 | 3 |
| P36551 | Coproporphyrinogen-III oxidase, mitochondrial OS=Homo sapiens GN=CPOX PE=1 SV=3 - [HEM6_HUMAN] | 3 | 1 | 3 | 3 |
| P14868 | Aspartyl-tRNA synthetase, cytoplasmic OS=Homo sapiens GN=DARS PE=1 SV=2 - [SYDC_HUMAN] | 3 | 1 | 3 | 3 |
| Q8N2K0 | Monoacylglycerol lipase ABHD12 OS=Homo sapiens GN=ABHD12 PE=2 SV=2 - [ABD12_HUMAN] | 3 | 1 | 3 | 3 |
| Q9BUJ2 | Heterogeneous nuclear ribonucleoprotein U-like protein 1 OS=Homo sapiens GN=HNRNPUL1 PE=1 SV=2 - [HNRL1_HUMAN] | 3 | 1 | 3 | 3 |
| Q8IZ40 | REST corepressor 2 OS=Homo sapiens GN=RCOR2 PE=2 SV=2 - [RCOR2_HUMAN] | 3 | 1 | 3 | 3 |
| P08559 | Pyruvate dehydrogenase E1 component subunit alpha, somatic form, mitochondrial OS=Homo sapiens GN=PDHA1 PE=1 SV=3 - [ODPA_HUMAN] | 3 | 1 | 3 | 3 |
| Q15404 | Ras suppressor protein 1 OS=Homo sapiens GN=RSU1 PE=1 SV=3 - [RSU1_HUMAN] | 3 | 1 | 3 | 3 |
| Q6UX04 | Peptidyl-prolyl cis-trans isomerase CWC27 homolog OS=Homo sapiens GN=CWC27 PE=1 SV=1 - [CWC27_HUMAN] | 3 | 1 | 3 | 3 |
| Q9BVP2 | Guanine nucleotide-binding protein-like 3 OS=Homo sapiens GN=GNL3 PE=1 SV=2 - [GNL3_HUMAN] | 3 | 1 | 3 | 3 |
| P49642 | DNA primase small subunit OS=Homo sapiens GN=PRIM1 PE=1 SV=1 - [PRI1_HUMAN] | 3 | 1 | 3 | 3 |
| Q9H3G5 | Probable serine carboxypeptidase CPVL OS=Homo sapiens GN=CPVL PE=1 SV=2 - [CPVL_HUMAN] | 3 | 1 | 3 | 3 |
| Q9NRY5 | Protein FAM114A2 OS=Homo sapiens GN=FAM114A2 PE=1 SV=4 - [F1142_HUMAN] | 3 | 1 | 3 | 3 |
| Q70UQ0 | Inhibitor of nuclear factor kappa-B kinase-interacting protein OS=Homo sapiens GN=IKIP PE=1 SV=1 - [IKIP_HUMAN] | 3 | 1 | 3 | 3 |
| O14773 | Tripeptidyl-peptidase 1 OS=Homo sapiens GN=TPP1 PE=1 SV=2 - [TPP1_HUMAN] | 3 | 1 | 3 | 3 |
| Q6NYC1 | Bifunctional arginine demethylase and lysyl-hydroxylase JMJD6 OS=Homo sapiens GN=JMJD6 PE=1 SV=1 - [JMJD6_HUMAN] | 3 | 1 | 3 | 3 |
| P49585 | Choline-phosphate cytidylyltransferase A OS=Homo sapiens GN=PCYT1A PE=1 SV=2 - [PCY1A_HUMAN] | 3 | 1 | 3 | 3 |
| Q8NBJ7 | Sulfatase-modifying factor 2 OS=Homo sapiens GN=SUMF2 PE=1 SV=2 - [SUMF2_HUMAN] | 3 | 1 | 3 | 3 |
| P61313 | 60S ribosomal protein L15 OS=Homo sapiens GN=RPL15 PE=1 SV=2 - [RL15_HUMAN] | 3 | 1 | 3 | 3 |
| Q9Y314 | Nitric oxide synthase-interacting protein OS=Homo sapiens GN=NOSIP PE=1 SV=1 - [NOSIP_HUMAN] | 3 | 1 | 3 | 3 |
| Q06203 | Amidophosphoribosyltransferase OS=Homo sapiens GN=PPAT PE=1 SV=1 - [PUR1_HUMAN] | 3 | 1 | 3 | 3 |
| P49406 | 39S ribosomal protein L19, mitochondrial OS=Homo sapiens GN=MRPL19 PE=1 SV=2 - [RM19_HUMAN] | 3 | 1 | 3 | 3 |
| P19404 | NADH dehydrogenase [ubiquinone] flavoprotein 2, mitochondrial OS=Homo sapiens GN=NDUFV2 PE=1 SV=2 - [NDUV2_HUMAN] | 3 | 1 | 3 | 3 |
| Q8WZA0 | Protein LZIC OS=Homo sapiens GN=LZIC PE=1 SV=1 - [LZIC_HUMAN] | 3 | 1 | 3 | 3 |
| P41227 | N-terminal acetyltransferase complex ARD1 subunit homolog A OS=Homo sapiens GN=ARD1A PE=1 SV=1 - [ARD1A_HUMAN] | 3 | 1 | 3 | 3 |
| Q9UHR5 | SAP30-binding protein OS=Homo sapiens GN=SAP30BP PE=1 SV=1 - [S30BP_HUMAN] | 3 | 1 | 3 | 3 |
| Q14847 | LIM and SH3 domain protein 1 OS=Homo sapiens GN=LASP1 PE=1 SV=2 - [LASP1_HUMAN] | 3 | 1 | 3 | 3 |
| P19387 | DNA-directed RNA polymerase II subunit RPB3 OS=Homo sapiens GN=POLR2C PE=1 SV=2 - [RPB3_HUMAN] | 3 | 1 | 3 | 3 |
| O95983 | Methyl-CpG-binding domain protein 3 OS=Homo sapiens GN=MBD3 PE=1 SV=1 - [MBD3_HUMAN] | 3 | 1 | 3 | 3 |
| Q9BZX2 | Uridine-cytidine kinase 2 OS=Homo sapiens GN=UCK2 PE=1 SV=1 - [UCK2_HUMAN] | 3 | 1 | 3 | 3 |
| O75608 | Acyl-protein thioesterase 1 OS=Homo sapiens GN=LYPLA1 PE=1 SV=1 - [LYPA1_HUMAN] | 3 | 1 | 3 | 3 |
| O75396 | Vesicle-trafficking protein SEC22b OS=Homo sapiens GN=SEC22B PE=1 SV=4 - [SC22B_HUMAN] | 3 | 1 | 3 | 3 |
| Q9UIJ7 | GTP:AMP phosphotransferase mitochondrial OS=Homo sapiens GN=AK3 PE=1 SV=4 - [KAD3_HUMAN] | 3 | 1 | 3 | 3 |
| Q9BRJ6 | Uncharacterized protein C7orf50 OS=Homo sapiens GN=C7orf50 PE=1 SV=1 - [CG050_HUMAN] | 3 | 1 | 3 | 3 |
| Q8WW12 | PEST proteolytic signal-containing nuclear protein OS=Homo sapiens GN=PCNP PE=1 SV=2 - [PCNP_HUMAN] | 3 | 1 | 3 | 3 |
| Q9BVG4 | UPF0368 protein Cxorf26 OS=Homo sapiens GN=CXorf26 PE=1 SV=1 - [CX026_HUMAN] | 3 | 1 | 3 | 3 |
| O60888 | Protein CutA OS=Homo sapiens GN=CUTA PE=1 SV=2 - [CUTA_HUMAN] | 3 | 1 | 3 | 3 |
| Q9BRA2 | Thioredoxin domain-containing protein 17 OS=Homo sapiens GN=TXNDC17 PE=1 SV=1 - [TXD17_HUMAN] | 3 | 1 | 3 | 3 |
| P52943 | Cysteine-rich protein 2 OS=Homo sapiens GN=CRIP2 PE=1 SV=1 - [CRIP2_HUMAN] | 3 | 1 | 3 | 3 |
| P31323 | cAMP-dependent protein kinase type II-beta regulatory subunit OS=Homo sapiens GN=PRKAR2B PE=1 SV=3 - [KAP3_HUMAN] | 2 | 1 | 3 | 3 |
| Q9NR31 | GTP-binding protein SAR1a OS=Homo sapiens GN=SAR1A PE=1 SV=1 - [SAR1A_HUMAN] | 2 | 1 | 3 | 3 |
| Q9BWD1 | Acetyl-CoA acetyltransferase, cytosolic OS=Homo sapiens GN=ACAT2 PE=1 SV=2 - [THIC_HUMAN] | 2 | 1 | 3 | 3 |
| P61956 | Small ubiquitin-related modifier 2 OS=Homo sapiens GN=SUMO2 PE=1 SV=1 - [SUMO2_HUMAN] | 2 | 1 | 3 | 3 |
| P62314 | Small nuclear ribonucleoprotein Sm D1 OS=Homo sapiens GN=SNRPD1 PE=1 SV=1 - [SMD1_HUMAN] | 2 | 1 | 3 | 3 |
| Q93009 | Ubiquitin carboxyl-terminal hydrolase 7 OS=Homo sapiens GN=USP7 PE=1 SV=2 - [UBP7_HUMAN] | 12 | 4 | 11 | 2.75 |
| Q9UQE7 | Structural maintenance of chromosomes protein 3 OS=Homo sapiens GN=SMC3 PE=1 SV=2 - [SMC3_HUMAN] | 9 | 3 | 8 | 2.6666667 |
| P98175 | RNA-binding protein 10 OS=Homo sapiens GN=RBM10 PE=1 SV=3 - [RBM10_HUMAN] | 9 | 3 | 8 | 2.6666667 |
| Q05682 | Caldesmon OS=Homo sapiens GN=CALD1 PE=1 SV=3 - [CALD1_HUMAN] | 9 | 3 | 8 | 2.6666667 |
| O43290 | U4/U6.U5 tri-snRNP-associated protein 1 OS=Homo sapiens GN=SART1 PE=1 SV=1 - [SNUT1_HUMAN] | 8 | 3 | 8 | 2.6666667 |
| Q14693 | Phosphatidate phosphatase LPIN1 OS=Homo sapiens GN=LPIN1 PE=2 SV=2 - [LPIN1_HUMAN] | 8 | 3 | 8 | 2.6666667 |
| Q9UL46 | Proteasome activator complex subunit 2 OS=Homo sapiens GN=PSME2 PE=1 SV=4 - [PSME2_HUMAN] | 8 | 3 | 8 | 2.6666667 |
| Q12904 | Aminoacyl tRNA synthetase complex-interacting multifunctional protein 1 OS=Homo sapiens GN=AIMP1 PE=1 SV=2 - [AIMP1_HUMAN] | 7 | 3 | 8 | 2.6666667 |
| P06493 | Cyclin-dependent kinase 1 OS=Homo sapiens GN=CDK1 PE=1 SV=3 - [CDK1_HUMAN] | 7 | 3 | 8 | 2.6666667 |
| Q8NC51 | Plasminogen activator inhibitor 1 RNA-binding protein OS=Homo sapiens GN=SERBP1 PE=1 SV=2 - [PAIRB_HUMAN] | 6 | 3 | 8 | 2.6666667 |
| O43809 | Cleavage and polyadenylation specificity factor subunit 5 OS=Homo sapiens GN=NUDT21 PE=1 SV=1 - [CPSF5_HUMAN] | 6 | 3 | 8 | 2.6666667 |
| Q9BTT0 | Acidic leucine-rich nuclear phosphoprotein 32 family member E OS=Homo sapiens GN=ANP32E PE=1 SV=1 - [AN32E_HUMAN] | 5 | 3 | 8 | 2.6666667 |
| Q96QK1 | Vacuolar protein sorting-associated protein 35 OS=Homo sapiens GN=VPS35 PE=1 SV=2 - [VPS35_HUMAN] | 12 | 5 | 13 | 2.6 |
| Q99497 | Protein DJ-1 OS=Homo sapiens GN=PARK7 PE=1 SV=2 - [PARK7_HUMAN] | 9 | 6 | 15 | 2.5 |
| Q9UNM6 | 26S proteasome non-ATPase regulatory subunit 13 OS=Homo sapiens GN=PSMD13 PE=1 SV=2 - [PSD13_HUMAN] | 8 | 4 | 10 | 2.5 |
| P21266 | Glutathione S-transferase Mu 3 OS=Homo sapiens GN=GSTM3 PE=1 SV=3 - [GSTM3_HUMAN] | 8 | 4 | 10 | 2.5 |
| P09661 | U2 small nuclear ribonucleoprotein A' OS=Homo sapiens GN=SNRPA1 PE=1 SV=2 - [RU2A_HUMAN] | 8 | 4 | 10 | 2.5 |
| P25787 | Proteasome subunit alpha type-2 OS=Homo sapiens GN=PSMA2 PE=1 SV=2 - [PSA2_HUMAN] | 6 | 4 | 10 | 2.5 |
| P49792 | E3 SUMO-protein ligase RanBP2 OS=Homo sapiens GN=RANBP2 PE=1 SV=2 - [RBP2_HUMAN] | 6 | 2 | 5 | 2.5 |
| Q99829 | Copine-1 OS=Homo sapiens GN=CPNE1 PE=1 SV=1 - [CPNE1_HUMAN] | 6 | 2 | 5 | 2.5 |
| P13591 | Neural cell adhesion molecule 1 OS=Homo sapiens GN=NCAM1 PE=1 SV=3 - [NCAM1_HUMAN] | 6 | 2 | 5 | 2.5 |
| Q9NQX3 | Gephyrin OS=Homo sapiens GN=GPHN PE=1 SV=1 - [GEPH_HUMAN] | 5 | 2 | 5 | 2.5 |
| P39656 | Dolichyl-diphosphooligosaccharide--protein glycosyltransferase 48 kDa subunit OS=Homo sapiens GN=DDOST PE=1 SV=4 - [OST48_HUMAN] | 5 | 2 | 5 | 2.5 |
| Q9C0B1 | Protein fto OS=Homo sapiens GN=FTO PE=1 SV=3 - [FTO_HUMAN] | 5 | 2 | 5 | 2.5 |
| P37268 | Squalene synthase OS=Homo sapiens GN=FDFT1 PE=1 SV=1 - [FDFT_HUMAN] | 5 | 2 | 5 | 2.5 |
| P12532 | Creatine kinase U-type, mitochondrial OS=Homo sapiens GN=CKMT1A PE=1 SV=1 - [KCRU_HUMAN] | 5 | 2 | 5 | 2.5 |
| P25788 | Proteasome subunit alpha type-3 OS=Homo sapiens GN=PSMA3 PE=1 SV=2 - [PSA3_HUMAN] | 5 | 2 | 5 | 2.5 |
| P60660 | Myosin light polypeptide 6 OS=Homo sapiens GN=MYL6 PE=1 SV=2 - [MYL6_HUMAN] | 5 | 2 | 5 | 2.5 |
| P53990 | IST1 homolog OS=Homo sapiens GN=KIAA0174 PE=1 SV=1 - [IST1_HUMAN] | 4 | 2 | 5 | 2.5 |
| Q15050 | Ribosome biogenesis regulatory protein homolog OS=Homo sapiens GN=RRS1 PE=1 SV=2 - [RRS1_HUMAN] | 4 | 2 | 5 | 2.5 |
| Q9BTE6 | Alanyl-tRNA editing protein Aarsd1 OS=Homo sapiens GN=AARSD1 PE=1 SV=2 - [AASD1_HUMAN] | 4 | 2 | 5 | 2.5 |
| P07858 | Cathepsin B OS=Homo sapiens GN=CTSB PE=1 SV=3 - [CATB_HUMAN] | 4 | 2 | 5 | 2.5 |
| P46108 | Adapter molecule crk OS=Homo sapiens GN=CRK PE=1 SV=2 - [CRK_HUMAN] | 4 | 2 | 5 | 2.5 |
| Q99653 | Calcium-binding protein p22 OS=Homo sapiens GN=CHP PE=1 SV=3 - [CHP1_HUMAN] | 4 | 2 | 5 | 2.5 |
| Q15126 | Phosphomevalonate kinase OS=Homo sapiens GN=PMVK PE=1 SV=3 - [PMVK_HUMAN] | 4 | 2 | 5 | 2.5 |
| P62913 | 60S ribosomal protein L11 OS=Homo sapiens GN=RPL11 PE=1 SV=2 - [RL11_HUMAN] | 4 | 2 | 5 | 2.5 |
| Q9GZT3 | SRA stem-loop-interacting RNA-binding protein, mitochondrial OS=Homo sapiens GN=SLIRP PE=1 SV=1 - [SLIRP_HUMAN] | 4 | 2 | 5 | 2.5 |
| Q9H2U2 | Inorganic pyrophosphatase 2, mitochondrial OS=Homo sapiens GN=PPA2 PE=1 SV=2 - [IPYR2_HUMAN] | 3 | 2 | 5 | 2.5 |
| Q14166 | Tubulin--tyrosine ligase-like protein 12 OS=Homo sapiens GN=TTLL12 PE=1 SV=2 - [TTL12_HUMAN] | 3 | 2 | 5 | 2.5 |
| P11172 | Uridine 5'-monophosphate synthase OS=Homo sapiens GN=UMPS PE=1 SV=1 - [UMPS_HUMAN] | 3 | 2 | 5 | 2.5 |
| Q9NS69 | Mitochondrial import receptor subunit TOM22 homolog OS=Homo sapiens GN=TOMM22 PE=1 SV=3 - [TOM22_HUMAN] | 3 | 2 | 5 | 2.5 |
| O43432 | Eukaryotic translation initiation factor 4 gamma 3 OS=Homo sapiens GN=EIF4G3 PE=1 SV=2 - [IF4G3_HUMAN] | 2 | 2 | 5 | 2.5 |
| Q9UKL0 | REST corepressor 1 OS=Homo sapiens GN=RCOR1 PE=1 SV=1 - [RCOR1_HUMAN] | 2 | 2 | 5 | 2.5 |
| P13489 | Ribonuclease inhibitor OS=Homo sapiens GN=RNH1 PE=1 SV=2 - [RINI_HUMAN] | 11 | 5 | 12 | 2.4 |
| P35637 | RNA-binding protein FUS OS=Homo sapiens GN=FUS PE=1 SV=1 - [FUS_HUMAN] | 7 | 5 | 12 | 2.4 |
| P49959 | Double-strand break repair protein MRE11A OS=Homo sapiens GN=MRE11A PE=1 SV=3 - [MRE11_HUMAN] | 8 | 3 | 7 | 2.3333333 |
| P15121 | Aldose reductase OS=Homo sapiens GN=AKR1B1 PE=1 SV=3 - [ALDR_HUMAN] | 7 | 3 | 7 | 2.3333333 |
| Q9Y5K5 | Ubiquitin carboxyl-terminal hydrolase isozyme L5 OS=Homo sapiens GN=UCHL5 PE=1 SV=3 - [UCHL5_HUMAN] | 7 | 3 | 7 | 2.3333333 |
| Q13148 | TAR DNA-binding protein 43 OS=Homo sapiens GN=TARDBP PE=1 SV=1 - [TADBP_HUMAN] | 6 | 3 | 7 | 2.3333333 |
| Q8NEJ9 | Neuroguidin OS=Homo sapiens GN=NGDN PE=1 SV=1 - [NGDN_HUMAN] | 6 | 3 | 7 | 2.3333333 |
| Q96KB5 | Lymphokine-activated killer T-cell-originated protein kinase OS=Homo sapiens GN=PBK PE=1 SV=3 - [TOPK_HUMAN] | 6 | 3 | 7 | 2.3333333 |
| O75340 | Programmed cell death protein 6 OS=Homo sapiens GN=PDCD6 PE=1 SV=1 - [PDCD6_HUMAN] | 6 | 3 | 7 | 2.3333333 |
| P17677 | Neuromodulin OS=Homo sapiens GN=GAP43 PE=1 SV=1 - [NEUM_HUMAN] | 6 | 3 | 7 | 2.3333333 |
| P49756 | RNA-binding protein 25 OS=Homo sapiens GN=RBM25 PE=1 SV=3 - [RBM25_HUMAN] | 5 | 3 | 7 | 2.3333333 |
| Q96I25 | Splicing factor 45 OS=Homo sapiens GN=RBM17 PE=1 SV=1 - [SPF45_HUMAN] | 5 | 3 | 7 | 2.3333333 |
| Q13573 | SNW domain-containing protein 1 OS=Homo sapiens GN=SNW1 PE=1 SV=1 - [SNW1_HUMAN] | 5 | 3 | 7 | 2.3333333 |
| P28066 | Proteasome subunit alpha type-5 OS=Homo sapiens GN=PSMA5 PE=1 SV=3 - [PSA5_HUMAN] | 5 | 3 | 7 | 2.3333333 |
| P25685 | DnaJ homolog subfamily B member 1 OS=Homo sapiens GN=DNAJB1 PE=1 SV=4 - [DNJB1_HUMAN] | 8 | 4 | 9 | 2.25 |
| P68400 | Casein kinase II subunit alpha OS=Homo sapiens GN=CSNK2A1 PE=1 SV=1 - [CSK21_HUMAN] | 8 | 4 | 9 | 2.25 |
| P00492 | Hypoxanthine-guanine phosphoribosyltransferase OS=Homo sapiens GN=HPRT1 PE=1 SV=2 - [HPRT_HUMAN] | 8 | 4 | 9 | 2.25 |
| P61586 | Transforming protein RhoA OS=Homo sapiens GN=RHOA PE=1 SV=1 - [RHOA_HUMAN] | 7 | 4 | 9 | 2.25 |
| P78344 | Eukaryotic translation initiation factor 4 gamma 2 OS=Homo sapiens GN=EIF4G2 PE=1 SV=1 - [IF4G2_HUMAN] | 11 | 5 | 11 | 2.2 |
| Q14697 | Neutral alpha-glucosidase AB OS=Homo sapiens GN=GANAB PE=1 SV=3 - [GANAB_HUMAN] | 11 | 5 | 11 | 2.2 |
| P35249 | Replication factor C subunit 4 OS=Homo sapiens GN=RFC4 PE=1 SV=2 - [RFC4_HUMAN] | 11 | 5 | 11 | 2.2 |
| Q6PKG0 | La-related protein 1 OS=Homo sapiens GN=LARP1 PE=1 SV=2 - [LARP1_HUMAN] | 10 | 5 | 11 | 2.2 |
| Q14157 | Ubiquitin-associated protein 2-like OS=Homo sapiens GN=UBAP2L PE=1 SV=2 - [UBP2L_HUMAN] | 11 | 7 | 15 | 2.1428571 |
| P33316 | Deoxyuridine 5'-triphosphate nucleotidohydrolase, mitochondrial OS=Homo sapiens GN=DUT PE=1 SV=4 - [DUT_HUMAN] | 8 | 7 | 15 | 2.1428571 |
| Q01082 | Spectrin beta chain, brain 1 OS=Homo sapiens GN=SPTBN1 PE=1 SV=2 - [SPTB2_HUMAN] | 25 | 14 | 29 | 2.0714286 |
| Q16891 | Mitochondrial inner membrane protein OS=Homo sapiens GN=IMMT PE=1 SV=1 - [IMMT_HUMAN] | 19 | 10 | 20 | 2 |
| Q03252 | Lamin-B2 OS=Homo sapiens GN=LMNB2 PE=1 SV=4 - [LMNB2_HUMAN] | 15 | 9 | 18 | 2 |
| Q15365 | Poly(rC)-binding protein 1 OS=Homo sapiens GN=PCBP1 PE=1 SV=2 - [PCBP1_HUMAN] | 7 | 8 | 16 | 2 |
| Q99623 | Prohibitin-2 OS=Homo sapiens GN=PHB2 PE=1 SV=2 - [PHB2_HUMAN] | 11 | 7 | 14 | 2 |
| Q9Y696 | Chloride intracellular channel protein 4 OS=Homo sapiens GN=CLIC4 PE=1 SV=4 - [CLIC4_HUMAN] | 10 | 7 | 14 | 2 |
| Q9NQG5 | Regulation of nuclear pre-mRNA domain-containing protein 1B OS=Homo sapiens GN=RPRD1B PE=1 SV=1 - [RPR1B_HUMAN] | 9 | 7 | 14 | 2 |
| P37802 | Transgelin-2 OS=Homo sapiens GN=TAGLN2 PE=1 SV=3 - [TAGL2_HUMAN] | 9 | 7 | 14 | 2 |
| P29692 | Elongation factor 1-delta OS=Homo sapiens GN=EEF1D PE=1 SV=5 - [EF1D_HUMAN] | 9 | 6 | 12 | 2 |
| O14776 | Transcription elongation regulator 1 OS=Homo sapiens GN=TCERG1 PE=1 SV=2 - [TCRG1_HUMAN] | 10 | 5 | 10 | 2 |
| Q7Z460 | CLIP-associating protein 1 OS=Homo sapiens GN=CLASP1 PE=1 SV=1 - [CLAP1_HUMAN] | 8 | 5 | 10 | 2 |
| P50502 | Hsc70-interacting protein OS=Homo sapiens GN=ST13 PE=1 SV=2 - [F10A1_HUMAN] | 7 | 5 | 10 | 2 |
| O14979 | Heterogeneous nuclear ribonucleoprotein D-like OS=Homo sapiens GN=HNRPDL PE=1 SV=3 - [HNRDL_HUMAN] | 6 | 5 | 10 | 2 |
| P52306 | Rap1 GTPase-GDP dissociation stimulator 1 OS=Homo sapiens GN=RAP1GDS1 PE=1 SV=3 - [GDS1_HUMAN] | 9 | 4 | 8 | 2 |
| P17980 | 26S protease regulatory subunit 6A OS=Homo sapiens GN=PSMC3 PE=1 SV=3 - [PRS6A_HUMAN] | 7 | 4 | 8 | 2 |
| P31689 | DnaJ homolog subfamily A member 1 OS=Homo sapiens GN=DNAJA1 PE=1 SV=2 - [DNJA1_HUMAN] | 7 | 4 | 8 | 2 |
| P00568 | Adenylate kinase isoenzyme 1 OS=Homo sapiens GN=AK1 PE=1 SV=3 - [KAD1_HUMAN] | 7 | 4 | 8 | 2 |
| P05387 | 60S acidic ribosomal protein P2 OS=Homo sapiens GN=RPLP2 PE=1 SV=1 - [RLA2_HUMAN] | 5 | 4 | 8 | 2 |
| P11216 | Glycogen phosphorylase, brain form OS=Homo sapiens GN=PYGB PE=1 SV=5 - [PYGB_HUMAN] | 8 | 3 | 6 | 2 |
| Q14444 | Caprin-1 OS=Homo sapiens GN=CAPRIN1 PE=1 SV=2 - [CAPR1_HUMAN] | 7 | 3 | 6 | 2 |
| Q9UKM9 | RNA-binding protein Raly OS=Homo sapiens GN=RALY PE=1 SV=1 - [RALY_HUMAN] | 7 | 3 | 6 | 2 |
| Q5SW79 | Centrosomal protein of 170 kDa OS=Homo sapiens GN=CEP170 PE=1 SV=1 - [CE170_HUMAN] | 6 | 3 | 6 | 2 |
| Q02809 | Procollagen-lysine,2-oxoglutarate 5-dioxygenase 1 OS=Homo sapiens GN=PLOD1 PE=1 SV=2 - [PLOD1_HUMAN] | 6 | 3 | 6 | 2 |
| Q7L2H7 | Eukaryotic translation initiation factor 3 subunit M OS=Homo sapiens GN=EIF3M PE=1 SV=1 - [EIF3M_HUMAN] | 6 | 3 | 6 | 2 |
| Q96PZ0 | Pseudouridylate synthase 7 homolog OS=Homo sapiens GN=PUS7 PE=1 SV=2 - [PUS7_HUMAN] | 6 | 3 | 6 | 2 |
| Q96KP4 | Cytosolic non-specific dipeptidase OS=Homo sapiens GN=CNDP2 PE=1 SV=2 - [CNDP2_HUMAN] | 6 | 3 | 6 | 2 |
| P49720 | Proteasome subunit beta type-3 OS=Homo sapiens GN=PSMB3 PE=1 SV=2 - [PSB3_HUMAN] | 6 | 3 | 6 | 2 |
| Q15631 | Translin OS=Homo sapiens GN=TSN PE=1 SV=1 - [TSN_HUMAN] | 6 | 3 | 6 | 2 |
| Q8TEX9 | Importin-4 OS=Homo sapiens GN=IPO4 PE=1 SV=2 - [IPO4_HUMAN] | 5 | 3 | 6 | 2 |
| Q8WXI9 | Transcriptional repressor p66-beta OS=Homo sapiens GN=GATAD2B PE=1 SV=1 - [P66B_HUMAN] | 5 | 3 | 6 | 2 |
| Q7L1Q6 | Basic leucine zipper and W2 domain-containing protein 1 OS=Homo sapiens GN=BZW1 PE=1 SV=1 - [BZW1_HUMAN] | 5 | 3 | 6 | 2 |
| Q16630 | Cleavage and polyadenylation specificity factor subunit 6 OS=Homo sapiens GN=CPSF6 PE=1 SV=2 - [CPSF6_HUMAN] | 5 | 3 | 6 | 2 |
| P18621 | 60S ribosomal protein L17 OS=Homo sapiens GN=RPL17 PE=1 SV=3 - [RL17_HUMAN] | 5 | 3 | 6 | 2 |
| P32322 | Pyrroline-5-carboxylate reductase 1, mitochondrial OS=Homo sapiens GN=PYCR1 PE=1 SV=2 - [P5CR1_HUMAN] | 5 | 3 | 6 | 2 |
| P19105 | Myosin regulatory light chain 12A OS=Homo sapiens GN=MYL12A PE=1 SV=2 - [ML12A_HUMAN] | 5 | 3 | 6 | 2 |
| P63173 | 60S ribosomal protein L38 OS=Homo sapiens GN=RPL38 PE=1 SV=2 - [RL38_HUMAN] | 4 | 3 | 6 | 2 |
| P61204 | ADP-ribosylation factor 3 OS=Homo sapiens GN=ARF3 PE=1 SV=2 - [ARF3_HUMAN] | 3 | 3 | 6 | 2 |
| Q14151 | Scaffold attachment factor B2 OS=Homo sapiens GN=SAFB2 PE=1 SV=1 - [SAFB2_HUMAN] | 2 | 3 | 6 | 2 |
| Q16881 | Thioredoxin reductase 1, cytoplasmic OS=Homo sapiens GN=TXNRD1 PE=1 SV=3 - [TRXR1_HUMAN] | 6 | 2 | 4 | 2 |
| Q15075 | Early endosome antigen 1 OS=Homo sapiens GN=EEA1 PE=1 SV=2 - [EEA1_HUMAN] | 5 | 2 | 4 | 2 |
| Q14160 | Protein scribble homolog OS=Homo sapiens GN=SCRIB PE=1 SV=4 - [SCRIB_HUMAN] | 5 | 2 | 4 | 2 |
| P49790 | Nuclear pore complex protein Nup153 OS=Homo sapiens GN=NUP153 PE=1 SV=2 - [NU153_HUMAN] | 5 | 2 | 4 | 2 |
| O15371 | Eukaryotic translation initiation factor 3 subunit D OS=Homo sapiens GN=EIF3D PE=1 SV=1 - [EIF3D_HUMAN] | 5 | 2 | 4 | 2 |
| P51858 | Hepatoma-derived growth factor OS=Homo sapiens GN=HDGF PE=1 SV=1 - [HDGF_HUMAN] | 5 | 2 | 4 | 2 |
| P30626 | Sorcin OS=Homo sapiens GN=SRI PE=1 SV=1 - [SORCN_HUMAN] | 5 | 2 | 4 | 2 |
| P07942 | Laminin subunit beta-1 OS=Homo sapiens GN=LAMB1 PE=1 SV=2 - [LAMB1_HUMAN] | 4 | 2 | 4 | 2 |
| Q8NBJ5 | Procollagen galactosyltransferase 1 OS=Homo sapiens GN=GLT25D1 PE=1 SV=1 - [GT251_HUMAN] | 4 | 2 | 4 | 2 |
| Q9NW13 | RNA-binding protein 28 OS=Homo sapiens GN=RBM28 PE=1 SV=3 - [RBM28_HUMAN] | 4 | 2 | 4 | 2 |
| Q9BWU0 | Kanadaptin OS=Homo sapiens GN=SLC4A1AP PE=1 SV=1 - [NADAP_HUMAN] | 4 | 2 | 4 | 2 |
| Q9UQ88 | Cyclin-dependent kinase 11A OS=Homo sapiens GN=CDK11A PE=1 SV=4 - [CD11A_HUMAN] | 4 | 2 | 4 | 2 |
| Q13895 | Bystin OS=Homo sapiens GN=BYSL PE=1 SV=3 - [BYST_HUMAN] | 4 | 2 | 4 | 2 |
| Q86TG7 | Retrotransposon-derived protein PEG10 OS=Homo sapiens GN=PEG10 PE=1 SV=2 - [PEG10_HUMAN] | 4 | 2 | 4 | 2 |
| Q8NCA5 | Protein FAM98A OS=Homo sapiens GN=FAM98A PE=1 SV=1 - [FA98A_HUMAN] | 4 | 2 | 4 | 2 |
| O96019 | Actin-like protein 6A OS=Homo sapiens GN=ACTL6A PE=1 SV=1 - [ACL6A_HUMAN] | 4 | 2 | 4 | 2 |
| Q9Y376 | Calcium-binding protein 39 OS=Homo sapiens GN=CAB39 PE=1 SV=1 - [CAB39_HUMAN] | 4 | 2 | 4 | 2 |
| P36543 | V-type proton ATPase subunit E 1 OS=Homo sapiens GN=ATP6V1E1 PE=1 SV=1 - [VATE1_HUMAN] | 4 | 2 | 4 | 2 |
| Q969X5 | Endoplasmic reticulum-Golgi intermediate compartment protein 1 OS=Homo sapiens GN=ERGIC1 PE=1 SV=1 - [ERGI1_HUMAN] | 4 | 2 | 4 | 2 |
| O43681 | ATPase ASNA1 OS=Homo sapiens GN=ASNA1 PE=1 SV=2 - [ASNA_HUMAN] | 4 | 2 | 4 | 2 |
| Q8ND56 | Protein LSM14 homolog A OS=Homo sapiens GN=LSM14A PE=1 SV=3 - [LS14A_HUMAN] | 4 | 2 | 4 | 2 |
| P13861 | cAMP-dependent protein kinase type II-alpha regulatory subunit OS=Homo sapiens GN=PRKAR2A PE=1 SV=2 - [KAP2_HUMAN] | 4 | 2 | 4 | 2 |
| P48637 | Glutathione synthetase OS=Homo sapiens GN=GSS PE=1 SV=1 - [GSHB_HUMAN] | 4 | 2 | 4 | 2 |
| Q9UQB8 | Brain-specific angiogenesis inhibitor 1-associated protein 2 OS=Homo sapiens GN=BAIAP2 PE=1 SV=1 - [BAIP2_HUMAN] | 4 | 2 | 4 | 2 |
| P62917 | 60S ribosomal protein L8 OS=Homo sapiens GN=RPL8 PE=1 SV=2 - [RL8_HUMAN] | 4 | 2 | 4 | 2 |
| P31937 | 3-hydroxyisobutyrate dehydrogenase, mitochondrial OS=Homo sapiens GN=HIBADH PE=1 SV=2 - [3HIDH_HUMAN] | 4 | 2 | 4 | 2 |
| P28072 | Proteasome subunit beta type-6 OS=Homo sapiens GN=PSMB6 PE=1 SV=4 - [PSB6_HUMAN] | 4 | 2 | 4 | 2 |
| Q9NR45 | Sialic acid synthase OS=Homo sapiens GN=NANS PE=1 SV=2 - [SIAS_HUMAN] | 4 | 2 | 4 | 2 |
| P23919 | Thymidylate kinase OS=Homo sapiens GN=DTYMK PE=1 SV=4 - [KTHY_HUMAN] | 4 | 2 | 4 | 2 |
| P40938 | Replication factor C subunit 3 OS=Homo sapiens GN=RFC3 PE=1 SV=2 - [RFC3_HUMAN] | 4 | 2 | 4 | 2 |
| Q9NPD3 | Exosome complex exonuclease RRP41 OS=Homo sapiens GN=EXOSC4 PE=1 SV=3 - [EXOS4_HUMAN] | 4 | 2 | 4 | 2 |
| O75494 | Splicing factor, arginine/serine-rich 13A OS=Homo sapiens GN=SFRS13A PE=1 SV=1 - [SF13A_HUMAN] | 4 | 2 | 4 | 2 |
| Q9H4A6 | Golgi phosphoprotein 3 OS=Homo sapiens GN=GOLPH3 PE=1 SV=1 - [GOLP3_HUMAN] | 4 | 2 | 4 | 2 |
| Q9NX63 | Coiled-coil-helix-coiled-coil-helix domain-containing protein 3, mitochondrial OS=Homo sapiens GN=CHCHD3 PE=1 SV=1 - [CHCH3_HUMAN] | 4 | 2 | 4 | 2 |
| Q96DG6 | Carboxymethylenebutenolidase homolog OS=Homo sapiens GN=CMBL PE=1 SV=1 - [CMBL_HUMAN] | 4 | 2 | 4 | 2 |
| O15173 | Membrane-associated progesterone receptor component 2 OS=Homo sapiens GN=PGRMC2 PE=1 SV=1 - [PGRC2_HUMAN] | 4 | 2 | 4 | 2 |
| O00461 | Golgi integral membrane protein 4 OS=Homo sapiens GN=GOLIM4 PE=1 SV=1 - [GOLI4_HUMAN] | 3 | 2 | 4 | 2 |
| P36957 | Dihydrolipoyllysine-residue succinyltransferase component of 2-oxoglutarate dehydrogenase complex, mitochondrial OS=Homo sapiens GN=DLST PE=1 SV=4 - [ODO2_HUMAN] | 3 | 2 | 4 | 2 |
| P13473 | Lysosome-associated membrane glycoprotein 2 OS=Homo sapiens GN=LAMP2 PE=1 SV=2 - [LAMP2_HUMAN] | 3 | 2 | 4 | 2 |
| P14324 | Farnesyl pyrophosphate synthase OS=Homo sapiens GN=FDPS PE=1 SV=4 - [FPPS_HUMAN] | 3 | 2 | 4 | 2 |
| Q9UI10 | Translation initiation factor eIF-2B subunit delta OS=Homo sapiens GN=EIF2B4 PE=1 SV=2 - [EI2BD_HUMAN] | 3 | 2 | 4 | 2 |
| Q15427 | Splicing factor 3B subunit 4 OS=Homo sapiens GN=SF3B4 PE=1 SV=1 - [SF3B4_HUMAN] | 3 | 2 | 4 | 2 |
| Q9H9Q2 | COP9 signalosome complex subunit 7b OS=Homo sapiens GN=COPS7B PE=1 SV=1 - [CSN7B_HUMAN] | 3 | 2 | 4 | 2 |
| O15305 | Phosphomannomutase 2 OS=Homo sapiens GN=PMM2 PE=1 SV=1 - [PMM2_HUMAN] | 3 | 2 | 4 | 2 |
| P30042 | ES1 protein homolog, mitochondrial OS=Homo sapiens GN=C21orf33 PE=1 SV=3 - [ES1_HUMAN] | 3 | 2 | 4 | 2 |
| O15347 | High mobility group protein B3 OS=Homo sapiens GN=HMGB3 PE=1 SV=4 - [HMGB3_HUMAN] | 3 | 2 | 4 | 2 |
| Q9BRP8 | Partner of Y14 and mago OS=Homo sapiens GN=PYM1 PE=1 SV=1 - [PYM1_HUMAN] | 3 | 2 | 4 | 2 |
| Q99471 | Prefoldin subunit 5 OS=Homo sapiens GN=PFDN5 PE=1 SV=2 - [PFD5_HUMAN] | 3 | 2 | 4 | 2 |
| P01023 | Alpha-2-macroglobulin OS=Homo sapiens GN=A2M PE=1 SV=3 - [A2MG_HUMAN] | 2 | 2 | 4 | 2 |
| P47755 | F-actin-capping protein subunit alpha-2 OS=Homo sapiens GN=CAPZA2 PE=1 SV=3 - [CAZA2_HUMAN] | 2 | 2 | 4 | 2 |
| P54725 | UV excision repair protein RAD23 homolog A OS=Homo sapiens GN=RAD23A PE=1 SV=1 - [RD23A_HUMAN] | 2 | 2 | 4 | 2 |
| P20340 | Ras-related protein Rab-6A OS=Homo sapiens GN=RAB6A PE=1 SV=3 - [RAB6A_HUMAN] | 2 | 2 | 4 | 2 |
| P22626 | Heterogeneous nuclear ribonucleoproteins A2/B1 OS=Homo sapiens GN=HNRNPA2B1 PE=1 SV=2 - [ROA2_HUMAN] | 13 | 15 | 29 | 1.9333333 |
| P09874 | Poly [ADP-ribose] polymerase 1 OS=Homo sapiens GN=PARP1 PE=1 SV=4 - [PARP1_HUMAN] | 17 | 9 | 17 | 1.8888889 |
| P25705 | ATP synthase subunit alpha, mitochondrial OS=Homo sapiens GN=ATP5A1 PE=1 SV=1 - [ATPA_HUMAN] | 13 | 9 | 17 | 1.8888889 |
| P29590 | Probable transcription factor PML OS=Homo sapiens GN=PML PE=1 SV=3 - [PML_HUMAN] | 14 | 8 | 15 | 1.875 |
| Q13813 | Spectrin alpha chain, brain OS=Homo sapiens GN=SPTAN1 PE=1 SV=3 - [SPTA2_HUMAN] | 45 | 23 | 43 | 1.8695652 |
| P46060 | Ran GTPase-activating protein 1 OS=Homo sapiens GN=RANGAP1 PE=1 SV=1 - [RAGP1_HUMAN] | 13 | 7 | 13 | 1.8571429 |
| P02545 | Lamin-A/C OS=Homo sapiens GN=LMNA PE=1 SV=1 - [LMNA_HUMAN] | 20 | 12 | 22 | 1.8333333 |
| P35998 | 26S protease regulatory subunit 7 OS=Homo sapiens GN=PSMC2 PE=1 SV=3 - [PRS7_HUMAN] | 14 | 6 | 11 | 1.8333333 |
| P36776 | Lon protease homolog, mitochondrial OS=Homo sapiens GN=LONP1 PE=1 SV=2 - [LONM_HUMAN] | 10 | 6 | 11 | 1.8333333 |
| Q13177 | Serine/threonine-protein kinase PAK 2 OS=Homo sapiens GN=PAK2 PE=1 SV=3 - [PAK2_HUMAN] | 10 | 6 | 11 | 1.8333333 |
| P17174 | Aspartate aminotransferase, cytoplasmic OS=Homo sapiens GN=GOT1 PE=1 SV=3 - [AATC_HUMAN] | 10 | 6 | 11 | 1.8333333 |
| Q15691 | Microtubule-associated protein RP/EB family member 1 OS=Homo sapiens GN=MAPRE1 PE=1 SV=3 - [MARE1_HUMAN] | 8 | 6 | 11 | 1.8333333 |
| Q15366 | Poly(rC)-binding protein 2 OS=Homo sapiens GN=PCBP2 PE=1 SV=1 - [PCBP2_HUMAN] | 4 | 6 | 11 | 1.8333333 |
| O00429 | Dynamin-1-like protein OS=Homo sapiens GN=DNM1L PE=1 SV=2 - [DNM1L_HUMAN] | 16 | 11 | 20 | 1.8181818 |
| P48643 | T-complex protein 1 subunit epsilon OS=Homo sapiens GN=CCT5 PE=1 SV=1 - [TCPE_HUMAN] | 13 | 10 | 18 | 1.8 |
| Q9UKX7 | Nuclear pore complex protein Nup50 OS=Homo sapiens GN=NUP50 PE=1 SV=2 - [NUP50_HUMAN] | 10 | 5 | 9 | 1.8 |
| Q9UNF1 | Melanoma-associated antigen D2 OS=Homo sapiens GN=MAGED2 PE=1 SV=2 - [MAGD2_HUMAN] | 9 | 5 | 9 | 1.8 |
| P16615 | Sarcoplasmic/endoplasmic reticulum calcium ATPase 2 OS=Homo sapiens GN=ATP2A2 PE=1 SV=1 - [AT2A2_HUMAN] | 9 | 5 | 9 | 1.8 |
| P46781 | 40S ribosomal protein S9 OS=Homo sapiens GN=RPS9 PE=1 SV=3 - [RS9_HUMAN] | 9 | 5 | 9 | 1.8 |
| Q9BWF3 | RNA-binding protein 4 OS=Homo sapiens GN=RBM4 PE=1 SV=1 - [RBM4_HUMAN] | 6 | 5 | 9 | 1.8 |
| Q15185 | Prostaglandin E synthase 3 OS=Homo sapiens GN=PTGES3 PE=1 SV=1 - [TEBP_HUMAN] | 6 | 5 | 9 | 1.8 |
| Q13151 | Heterogeneous nuclear ribonucleoprotein A0 OS=Homo sapiens GN=HNRNPA0 PE=1 SV=1 - [ROA0_HUMAN] | 5 | 5 | 9 | 1.8 |
| P62701 | 40S ribosomal protein S4, X isoform OS=Homo sapiens GN=RPS4X PE=1 SV=2 - [RS4X_HUMAN] | 12 | 9 | 16 | 1.7777778 |
| P39687 | Acidic leucine-rich nuclear phosphoprotein 32 family member A OS=Homo sapiens GN=ANP32A PE=1 SV=1 - [AN32A_HUMAN] | 6 | 9 | 16 | 1.7777778 |
| P55060 | Exportin-2 OS=Homo sapiens GN=CSE1L PE=1 SV=3 - [XPO2_HUMAN] | 20 | 12 | 21 | 1.75 |
| P31948 | Stress-induced-phosphoprotein 1 OS=Homo sapiens GN=STIP1 PE=1 SV=1 - [STIP1_HUMAN] | 18 | 12 | 21 | 1.75 |
| P46783 | 40S ribosomal protein S10 OS=Homo sapiens GN=RPS10 PE=1 SV=1 - [RS10_HUMAN] | 5 | 8 | 14 | 1.75 |
| O75534 | Cold shock domain-containing protein E1 OS=Homo sapiens GN=CSDE1 PE=1 SV=2 - [CSDE1_HUMAN] | 8 | 4 | 7 | 1.75 |
| Q9Y3I0 | UPF0027 protein C22orf28 OS=Homo sapiens GN=C22orf28 PE=1 SV=1 - [CV028_HUMAN] | 7 | 4 | 7 | 1.75 |
| P22695 | Cytochrome b-c1 complex subunit 2, mitochondrial OS=Homo sapiens GN=UQCRC2 PE=1 SV=3 - [QCR2_HUMAN] | 7 | 4 | 7 | 1.75 |
| Q9H3N1 | Thioredoxin-related transmembrane protein 1 OS=Homo sapiens GN=TMX1 PE=1 SV=1 - [TMX1_HUMAN] | 7 | 4 | 7 | 1.75 |
| Q9UKK9 | ADP-sugar pyrophosphatase OS=Homo sapiens GN=NUDT5 PE=1 SV=1 - [NUDT5_HUMAN] | 7 | 4 | 7 | 1.75 |
| Q9P2K5 | Myelin expression factor 2 OS=Homo sapiens GN=MYEF2 PE=1 SV=3 - [MYEF2_HUMAN] | 6 | 4 | 7 | 1.75 |
| Q04760 | Lactoylglutathione lyase OS=Homo sapiens GN=GLO1 PE=1 SV=4 - [LGUL_HUMAN] | 6 | 4 | 7 | 1.75 |
| Q9GZS3 | WD repeat-containing protein 61 OS=Homo sapiens GN=WDR61 PE=1 SV=1 - [WDR61_HUMAN] | 5 | 4 | 7 | 1.75 |
| P04075 | Fructose-bisphosphate aldolase A OS=Homo sapiens GN=ALDOA PE=1 SV=2 - [ALDOA_HUMAN] | 18 | 28 | 48 | 1.7142857 |
| Q04637 | Eukaryotic translation initiation factor 4 gamma 1 OS=Homo sapiens GN=EIF4G1 PE=1 SV=4 - [IF4G1_HUMAN] | 19 | 14 | 24 | 1.7142857 |
| P33993 | DNA replication licensing factor MCM7 OS=Homo sapiens GN=MCM7 PE=1 SV=4 - [MCM7_HUMAN] | 12 | 7 | 12 | 1.7142857 |
| Q15417 | Calponin-3 OS=Homo sapiens GN=CNN3 PE=1 SV=1 - [CNN3_HUMAN] | 8 | 7 | 12 | 1.7142857 |
| P07737 | Profilin-1 OS=Homo sapiens GN=PFN1 PE=1 SV=2 - [PROF1_HUMAN] | 8 | 7 | 12 | 1.7142857 |
| P12814 | Alpha-actinin-1 OS=Homo sapiens GN=ACTN1 PE=1 SV=2 - [ACTN1_HUMAN] | 11 | 17 | 29 | 1.7058824 |
| P50454 | Serpin H1 OS=Homo sapiens GN=SERPINH1 PE=1 SV=2 - [SERPH_HUMAN] | 14 | 10 | 17 | 1.7 |
| P61247 | 40S ribosomal protein S3a OS=Homo sapiens GN=RPS3A PE=1 SV=2 - [RS3A_HUMAN] | 13 | 10 | 17 | 1.7 |
| P09104 | Gamma-enolase OS=Homo sapiens GN=ENO2 PE=1 SV=3 - [ENOG_HUMAN] | 7 | 13 | 22 | 1.6923077 |
| P49321 | Nuclear autoantigenic sperm protein OS=Homo sapiens GN=NASP PE=1 SV=2 - [NASP_HUMAN] | 21 | 16 | 27 | 1.6875 |
| Q06830 | Peroxiredoxin-1 OS=Homo sapiens GN=PRDX1 PE=1 SV=1 - [PRDX1_HUMAN] | 13 | 15 | 25 | 1.6666667 |
| P30086 | Phosphatidylethanolamine-binding protein 1 OS=Homo sapiens GN=PEBP1 PE=1 SV=3 - [PEBP1_HUMAN] | 9 | 9 | 15 | 1.6666667 |
| P27816 | Microtubule-associated protein 4 OS=Homo sapiens GN=MAP4 PE=1 SV=3 - [MAP4_HUMAN] | 12 | 6 | 10 | 1.6666667 |
| Q7KZF4 | Staphylococcal nuclease domain-containing protein 1 OS=Homo sapiens GN=SND1 PE=1 SV=1 - [SND1_HUMAN] | 11 | 6 | 10 | 1.6666667 |
| Q8N1G4 | Leucine-rich repeat-containing protein 47 OS=Homo sapiens GN=LRRC47 PE=1 SV=1 - [LRC47_HUMAN] | 9 | 6 | 10 | 1.6666667 |
| O94826 | Mitochondrial import receptor subunit TOM70 OS=Homo sapiens GN=TOMM70A PE=1 SV=1 - [TOM70_HUMAN] | 9 | 6 | 10 | 1.6666667 |
| Q9Y285 | Phenylalanyl-tRNA synthetase alpha chain OS=Homo sapiens GN=FARSA PE=1 SV=3 - [SYFA_HUMAN] | 7 | 6 | 10 | 1.6666667 |
| P61604 | 10 kDa heat shock protein, mitochondrial OS=Homo sapiens GN=HSPE1 PE=1 SV=2 - [CH10_HUMAN] | 7 | 6 | 10 | 1.6666667 |
| O75489 | NADH dehydrogenase [ubiquinone] iron-sulfur protein 3, mitochondrial OS=Homo sapiens GN=NDUFS3 PE=1 SV=1 - [NDUS3_HUMAN] | 6 | 6 | 10 | 1.6666667 |
| P25398 | 40S ribosomal protein S12 OS=Homo sapiens GN=RPS12 PE=1 SV=3 - [RS12_HUMAN] | 6 | 6 | 10 | 1.6666667 |
| O75369 | Filamin-B OS=Homo sapiens GN=FLNB PE=1 SV=2 - [FLNB_HUMAN] | 5 | 6 | 10 | 1.6666667 |
| P09972 | Fructose-bisphosphate aldolase C OS=Homo sapiens GN=ALDOC PE=1 SV=2 - [ALDOC_HUMAN] | 2 | 6 | 10 | 1.6666667 |
| Q15758 | Neutral amino acid transporter B(0) OS=Homo sapiens GN=SLC1A5 PE=1 SV=2 - [AAAT_HUMAN] | 7 | 3 | 5 | 1.6666667 |
| P63151 | Serine/threonine-protein phosphatase 2A 55 kDa regulatory subunit B alpha isoform OS=Homo sapiens GN=PPP2R2A PE=1 SV=1 - [2ABA_HUMAN] | 7 | 3 | 5 | 1.6666667 |
| P23193 | Transcription elongation factor A protein 1 OS=Homo sapiens GN=TCEA1 PE=1 SV=2 - [TCEA1_HUMAN] | 7 | 3 | 5 | 1.6666667 |
| Q96A33 | Coiled-coil domain-containing protein 47 OS=Homo sapiens GN=CCDC47 PE=1 SV=1 - [CCD47_HUMAN] | 6 | 3 | 5 | 1.6666667 |
| Q9UHX1 | Poly(U)-binding-splicing factor PUF60 OS=Homo sapiens GN=PUF60 PE=1 SV=1 - [PUF60_HUMAN] | 6 | 3 | 5 | 1.6666667 |
| P51784 | Ubiquitin carboxyl-terminal hydrolase 11 OS=Homo sapiens GN=USP11 PE=1 SV=3 - [UBP11_HUMAN] | 5 | 3 | 5 | 1.6666667 |
| Q92542 | Nicastrin OS=Homo sapiens GN=NCSTN PE=1 SV=2 - [NICA_HUMAN] | 5 | 3 | 5 | 1.6666667 |
| O75152 | Zinc finger CCCH domain-containing protein 11A OS=Homo sapiens GN=ZC3H11A PE=1 SV=3 - [ZC11A_HUMAN] | 5 | 3 | 5 | 1.6666667 |
| Q6NUK1 | Calcium-binding mitochondrial carrier protein SCaMC-1 OS=Homo sapiens GN=SLC25A24 PE=1 SV=2 - [SCMC1_HUMAN] | 5 | 3 | 5 | 1.6666667 |
| P18754 | Regulator of chromosome condensation OS=Homo sapiens GN=RCC1 PE=1 SV=1 - [RCC1_HUMAN] | 5 | 3 | 5 | 1.6666667 |
| O94888 | UBX domain-containing protein 7 OS=Homo sapiens GN=UBXN7 PE=1 SV=2 - [UBXN7_HUMAN] | 5 | 3 | 5 | 1.6666667 |
| Q92820 | Gamma-glutamyl hydrolase OS=Homo sapiens GN=GGH PE=1 SV=2 - [GGH_HUMAN] | 5 | 3 | 5 | 1.6666667 |
| Q7Z434 | Mitochondrial antiviral-signaling protein OS=Homo sapiens GN=MAVS PE=1 SV=2 - [MAVS_HUMAN] | 5 | 3 | 5 | 1.6666667 |
| Q9NRN7 | L-aminoadipate-semialdehyde dehydrogenase-phosphopantetheinyl transferase OS=Homo sapiens GN=AASDHPPT PE=1 SV=2 - [ADPPT_HUMAN] | 5 | 3 | 5 | 1.6666667 |
| Q15006 | Tetratricopeptide repeat protein 35 OS=Homo sapiens GN=TTC35 PE=1 SV=1 - [TTC35_HUMAN] | 5 | 3 | 5 | 1.6666667 |
| O60884 | DnaJ homolog subfamily A member 2 OS=Homo sapiens GN=DNAJA2 PE=1 SV=1 - [DNJA2_HUMAN] | 5 | 3 | 5 | 1.6666667 |
| P19623 | Spermidine synthase OS=Homo sapiens GN=SRM PE=1 SV=1 - [SPEE_HUMAN] | 5 | 3 | 5 | 1.6666667 |
| P18124 | 60S ribosomal protein L7 OS=Homo sapiens GN=RPL7 PE=1 SV=1 - [RL7_HUMAN] | 5 | 3 | 5 | 1.6666667 |
| P25325 | 3-mercaptopyruvate sulfurtransferase OS=Homo sapiens GN=MPST PE=1 SV=3 - [THTM_HUMAN] | 5 | 3 | 5 | 1.6666667 |
| P83731 | 60S ribosomal protein L24 OS=Homo sapiens GN=RPL24 PE=1 SV=1 - [RL24_HUMAN] | 5 | 3 | 5 | 1.6666667 |
| Q15637 | Splicing factor 1 OS=Homo sapiens GN=SF1 PE=1 SV=4 - [SF01_HUMAN] | 4 | 3 | 5 | 1.6666667 |
| Q15046 | Lysyl-tRNA synthetase OS=Homo sapiens GN=KARS PE=1 SV=3 - [SYK_HUMAN] | 4 | 3 | 5 | 1.6666667 |
| Q9BQ67 | Glutamate-rich WD repeat-containing protein 1 OS=Homo sapiens GN=GRWD1 PE=1 SV=1 - [GRWD1_HUMAN] | 4 | 3 | 5 | 1.6666667 |
| P50897 | Palmitoyl-protein thioesterase 1 OS=Homo sapiens GN=PPT1 PE=1 SV=1 - [PPT1_HUMAN] | 4 | 3 | 5 | 1.6666667 |
| P29083 | General transcription factor IIE subunit 1 OS=Homo sapiens GN=GTF2E1 PE=1 SV=2 - [T2EA_HUMAN] | 4 | 3 | 5 | 1.6666667 |
| P62249 | 40S ribosomal protein S16 OS=Homo sapiens GN=RPS16 PE=1 SV=2 - [RS16_HUMAN] | 4 | 3 | 5 | 1.6666667 |
| P30044 | Peroxiredoxin-5, mitochondrial OS=Homo sapiens GN=PRDX5 PE=1 SV=4 - [PRDX5_HUMAN] | 4 | 3 | 5 | 1.6666667 |
| P28161 | Glutathione S-transferase Mu 2 OS=Homo sapiens GN=GSTM2 PE=1 SV=2 - [GSTM2_HUMAN] | 4 | 3 | 5 | 1.6666667 |
| Q9Y383 | Putative RNA-binding protein Luc7-like 2 OS=Homo sapiens GN=LUC7L2 PE=1 SV=2 - [LC7L2_HUMAN] | 3 | 3 | 5 | 1.6666667 |
| Q9NQ29 | Putative RNA-binding protein Luc7-like 1 OS=Homo sapiens GN=LUC7L PE=1 SV=1 - [LUC7L_HUMAN] | 3 | 3 | 5 | 1.6666667 |
| Q9P0L0 | Vesicle-associated membrane protein-associated protein A OS=Homo sapiens GN=VAPA PE=1 SV=3 - [VAPA_HUMAN] | 3 | 3 | 5 | 1.6666667 |
| P84103 | Splicing factor, arginine/serine-rich 3 OS=Homo sapiens GN=SFRS3 PE=1 SV=1 - [SFRS3_HUMAN] | 3 | 3 | 5 | 1.6666667 |
| P05386 | 60S acidic ribosomal protein P1 OS=Homo sapiens GN=RPLP1 PE=1 SV=1 - [RLA1_HUMAN] | 3 | 3 | 5 | 1.6666667 |
| P20700 | Lamin-B1 OS=Homo sapiens GN=LMNB1 PE=1 SV=2 - [LMNB1_HUMAN] | 18 | 17 | 28 | 1.6470588 |
| Q6S8J3 | POTE ankyrin domain family member E OS=Homo sapiens GN=POTEE PE=1 SV=3 - [POTEE_HUMAN] | 2 | 30 | 49 | 1.6333333 |
| P18669 | Phosphoglycerate mutase 1 OS=Homo sapiens GN=PGAM1 PE=1 SV=2 - [PGAM1_HUMAN] | 8 | 8 | 13 | 1.625 |
| P43246 | DNA mismatch repair protein Msh2 OS=Homo sapiens GN=MSH2 PE=1 SV=1 - [MSH2_HUMAN] | 8 | 5 | 8 | 1.6 |
| O00499 | Myc box-dependent-interacting protein 1 OS=Homo sapiens GN=BIN1 PE=1 SV=1 - [BIN1_HUMAN] | 8 | 5 | 8 | 1.6 |
| P40939 | Trifunctional enzyme subunit alpha, mitochondrial OS=Homo sapiens GN=HADHA PE=1 SV=2 - [ECHA_HUMAN] | 8 | 5 | 8 | 1.6 |
| P07954 | Fumarate hydratase, mitochondrial OS=Homo sapiens GN=FH PE=1 SV=3 - [FUMH_HUMAN] | 7 | 5 | 8 | 1.6 |
| O43837 | Isocitrate dehydrogenase [NAD] subunit beta, mitochondrial OS=Homo sapiens GN=IDH3B PE=1 SV=2 - [IDH3B_HUMAN] | 7 | 5 | 8 | 1.6 |
| P31930 | Cytochrome b-c1 complex subunit 1, mitochondrial OS=Homo sapiens GN=UQCRC1 PE=1 SV=3 - [QCR1_HUMAN] | 7 | 5 | 8 | 1.6 |
| Q86V81 | THO complex subunit 4 OS=Homo sapiens GN=THOC4 PE=1 SV=3 - [THOC4_HUMAN] | 6 | 5 | 8 | 1.6 |
| Q10567 | AP-1 complex subunit beta-1 OS=Homo sapiens GN=AP1B1 PE=1 SV=2 - [AP1B1_HUMAN] | 3 | 5 | 8 | 1.6 |
| Q9Y281 | Cofilin-2 OS=Homo sapiens GN=CFL2 PE=1 SV=1 - [COF2_HUMAN] | 3 | 5 | 8 | 1.6 |
| Q8IUE6 | Histone H2A type 2-B OS=Homo sapiens GN=HIST2H2AB PE=1 SV=3 - [H2A2B_HUMAN] | 2 | 5 | 8 | 1.6 |
| P04406 | Glyceraldehyde-3-phosphate dehydrogenase OS=Homo sapiens GN=GAPDH PE=1 SV=3 - [G3P_HUMAN] | 12 | 24 | 38 | 1.5833333 |
| P49419 | Alpha-aminoadipic semialdehyde dehydrogenase OS=Homo sapiens GN=ALDH7A1 PE=1 SV=5 - [AL7A1_HUMAN] | 11 | 7 | 11 | 1.5714286 |
| Q9NTK5 | Obg-like ATPase 1 OS=Homo sapiens GN=OLA1 PE=1 SV=2 - [OLA1_HUMAN] | 10 | 7 | 11 | 1.5714286 |
| P36578 | 60S ribosomal protein L4 OS=Homo sapiens GN=RPL4 PE=1 SV=5 - [RL4_HUMAN] | 10 | 7 | 11 | 1.5714286 |
| P13804 | Electron transfer flavoprotein subunit alpha, mitochondrial OS=Homo sapiens GN=ETFA PE=1 SV=1 - [ETFA_HUMAN] | 9 | 7 | 11 | 1.5714286 |
| P62081 | 40S ribosomal protein S7 OS=Homo sapiens GN=RPS7 PE=1 SV=1 - [RS7_HUMAN] | 8 | 7 | 11 | 1.5714286 |
| P09429 | High mobility group protein B1 OS=Homo sapiens GN=HMGB1 PE=1 SV=3 - [HMGB1_HUMAN] | 7 | 7 | 11 | 1.5714286 |
| P63010 | AP-2 complex subunit beta OS=Homo sapiens GN=AP2B1 PE=1 SV=1 - [AP2B1_HUMAN] | 6 | 7 | 11 | 1.5714286 |
| P00441 | Superoxide dismutase [Cu-Zn] OS=Homo sapiens GN=SOD1 PE=1 SV=2 - [SODC_HUMAN] | 5 | 7 | 11 | 1.5714286 |
| P26378 | ELAV-like protein 4 OS=Homo sapiens GN=ELAVL4 PE=1 SV=2 - [ELAV4_HUMAN] | 2 | 7 | 11 | 1.5714286 |
| P26583 | High mobility group protein B2 OS=Homo sapiens GN=HMGB2 PE=1 SV=2 - [HMGB2_HUMAN] | 7 | 9 | 14 | 1.5555556 |
| P22392 | Nucleoside diphosphate kinase B OS=Homo sapiens GN=NME2 PE=1 SV=1 - [NDKB_HUMAN] | 3 | 9 | 14 | 1.5555556 |
| P41219 | Peripherin OS=Homo sapiens GN=PRPH PE=1 SV=2 - [PERI_HUMAN] | 21 | 17 | 26 | 1.5294118 |
| P09651 | Heterogeneous nuclear ribonucleoprotein A1 OS=Homo sapiens GN=HNRNPA1 PE=1 SV=5 - [ROA1_HUMAN] | 12 | 19 | 29 | 1.5263158 |
| P23526 | Adenosylhomocysteinase OS=Homo sapiens GN=AHCY PE=1 SV=4 - [SAHH_HUMAN] | 7 | 8 | 4 | 0.5 |
| Q9P2J5 | Leucyl-tRNA synthetase, cytoplasmic OS=Homo sapiens GN=LARS PE=1 SV=2 - [SYLC_HUMAN] | 6 | 6 | 3 | 0.5 |
| Q96T88 | E3 ubiquitin-protein ligase UHRF1 OS=Homo sapiens GN=UHRF1 PE=1 SV=1 - [UHRF1_HUMAN] | 6 | 6 | 3 | 0.5 |
| Q13098 | COP9 signalosome complex subunit 1 OS=Homo sapiens GN=GPS1 PE=1 SV=4 - [CSN1_HUMAN] | 6 | 6 | 3 | 0.5 |
| Q14683 | Structural maintenance of chromosomes protein 1A OS=Homo sapiens GN=SMC1A PE=1 SV=2 - [SMC1A_HUMAN] | 6 | 4 | 2 | 0.5 |
| P17655 | Calpain-2 catalytic subunit OS=Homo sapiens GN=CAPN2 PE=1 SV=6 - [CAN2_HUMAN] | 6 | 4 | 2 | 0.5 |
| P02461 | Collagen alpha-1(III) chain OS=Homo sapiens GN=COL3A1 PE=1 SV=4 - [CO3A1_HUMAN] | 5 | 4 | 2 | 0.5 |
| Q9Y2X3 | Nucleolar protein 58 OS=Homo sapiens GN=NOP58 PE=1 SV=1 - [NOP58_HUMAN] | 5 | 4 | 2 | 0.5 |
| O95299 | NADH dehydrogenase [ubiquinone] 1 alpha subcomplex subunit 10, mitochondrial OS=Homo sapiens GN=NDUFA10 PE=1 SV=1 - [NDUAA_HUMAN] | 4 | 4 | 2 | 0.5 |
| P19367 | Hexokinase-1 OS=Homo sapiens GN=HK1 PE=1 SV=3 - [HXK1_HUMAN] | 3 | 4 | 2 | 0.5 |
| Q13243 | Splicing factor, arginine/serine-rich 5 OS=Homo sapiens GN=SFRS5 PE=1 SV=1 - [SFRS5_HUMAN] | 2 | 4 | 2 | 0.5 |
| P08754 | Guanine nucleotide-binding protein G(k) subunit alpha OS=Homo sapiens GN=GNAI3 PE=1 SV=3 - [GNAI3_HUMAN] | 2 | 4 | 2 | 0.5 |
| Q99613 | Eukaryotic translation initiation factor 3 subunit C OS=Homo sapiens GN=EIF3C PE=1 SV=1 - [EIF3C_HUMAN] | 9 | 8 | 3 | 0.375 |
| P02765 | Alpha-2-HS-glycoprotein OS=Homo sapiens GN=AHSG PE=1 SV=1 - [FETUA_HUMAN] | 3 | 6 | 2 | 0.3333333 |
| Q10471 | Polypeptide N-acetylgalactosaminyltransferase 2 OS=Homo sapiens GN=GALNT2 PE=1 SV=1 - [GALT2_HUMAN] | 4 | 3 | 1 | 0.3333333 |
| O75643 | U5 small nuclear ribonucleoprotein 200 kDa helicase OS=Homo sapiens GN=SNRNP200 PE=1 SV=2 - [U520_HUMAN] | 3 | 3 | 1 | 0.3333333 |
| Q7Z3B4 | Nucleoporin p54 OS=Homo sapiens GN=NUP54 PE=1 SV=2 - [NUP54_HUMAN] | 3 | 3 | 1 | 0.3333333 |
| P55263 | Adenosine kinase OS=Homo sapiens GN=ADK PE=1 SV=2 - [ADK_HUMAN] | 3 | 3 | 1 | 0.3333333 |
| P33240 | Cleavage stimulation factor subunit 2 OS=Homo sapiens GN=CSTF2 PE=1 SV=1 - [CSTF2_HUMAN] | 3 | 3 | 1 | 0.3333333 |
| O00154 | Cytosolic acyl coenzyme A thioester hydrolase OS=Homo sapiens GN=ACOT7 PE=1 SV=3 - [BACH_HUMAN] | 3 | 3 | 1 | 0.3333333 |
| Q92665 | 28S ribosomal protein S31, mitochondrial OS=Homo sapiens GN=MRPS31 PE=1 SV=3 - [RT31_HUMAN] | 3 | 3 | 1 | 0.3333333 |
| O43657 | Tetraspanin-6 OS=Homo sapiens GN=TSPAN6 PE=1 SV=1 - [TSN6_HUMAN] | 3 | 3 | 1 | 0.3333333 |
| Q9Y5Z4 | Heme-binding protein 2 OS=Homo sapiens GN=HEBP2 PE=1 SV=1 - [HEBP2_HUMAN] | 3 | 3 | 1 | 0.3333333 |
| O14737 | Programmed cell death protein 5 OS=Homo sapiens GN=PDCD5 PE=1 SV=3 - [PDCD5_HUMAN] | 3 | 3 | 1 | 0.3333333 |
| P57740 | Nuclear pore complex protein Nup107 OS=Homo sapiens GN=NUP107 PE=1 SV=1 - [NU107_HUMAN] | 2 | 3 | 1 | 0.3333333 |
| P37837 | Transaldolase OS=Homo sapiens GN=TALDO1 PE=1 SV=2 - [TALDO_HUMAN] | 5 | 4 | 1 | 0.25 |
| P19823 | Inter-alpha-trypsin inhibitor heavy chain H2 OS=Homo sapiens GN=ITIH2 PE=1 SV=2 - [ITIH2_HUMAN] | 4 | 4 | 1 | 0.25 |
| P42224 | Signal transducer and activator of transcription 1-alpha/beta OS=Homo sapiens GN=STAT1 PE=1 SV=2 - [STAT1_HUMAN] | 4 | 4 | 1 | 0.25 |
| O43242 | 26S proteasome non-ATPase regulatory subunit 3 OS=Homo sapiens GN=PSMD3 PE=1 SV=2 - [PSMD3_HUMAN] | 4 | 4 | 1 | 0.25 |
| P21397 | Amine oxidase [flavin-containing] A OS=Homo sapiens GN=MAOA PE=1 SV=1 - [AOFA_HUMAN] | 4 | 4 | 1 | 0.25 |
| P68431 | Histone H3.1 OS=Homo sapiens GN=HIST1H3A PE=1 SV=2 - [H31_HUMAN] | 3 | 4 | 1 | 0.25 |
| P62316 | Small nuclear ribonucleoprotein Sm D2 OS=Homo sapiens GN=SNRPD2 PE=1 SV=1 - [SMD2_HUMAN] | 4 | 5 | 1 | 0.2 |

**Supplementary Table 3:** A full list of proteins exclusively identified in *hMT3* overexpressed in UKF-NB-4 cell line

| Accession | Description | ΣUnique | PSM | PSM | AAs | MW [kDa] | calc. pI |
| --- | --- | --- | --- | --- | --- | --- | --- |
|  |  | Peptides | UKF-NB-4 | UKF-NB-4 |  |  |  |
|  |  |  | *Mock* | *hMT3* |  |  |  |
| Q99543 | DnaJ homolog subfamily C member 2 OS=Homo sapiens GN=DNAJC2 PE=1 SV=4 - [DNJC2_HUMAN] | 6 | 0 | 6 | 621 | 72 | 8.7 |
| P41223 | Protein BUD31 homolog OS=Homo sapiens GN=BUD31 PE=1 SV=2 - [BUD31_HUMAN] | 5 | 0 | 5 | 144 | 17 | 8.82 |
| Q53EL6 | Programmed cell death protein 4 OS=Homo sapiens GN=PDCD4 PE=1 SV=2 - [PDCD4_HUMAN] | 5 | 0 | 5 | 469 | 51.7 | 5.21 |
| P02792 | Ferritin light chain OS=Homo sapiens GN=FTL PE=1 SV=2 - [FRIL_HUMAN] | 4 | 0 | 5 | 175 | 20 | 5.78 |
| P61923 | Coatomer subunit zeta-1 OS=Homo sapiens GN=COPZ1 PE=1 SV=1 - [COPZ1_HUMAN] | 4 | 0 | 4 | 177 | 20.2 | 4.81 |
| P20618 | Proteasome subunit beta type-1 OS=Homo sapiens GN=PSMB1 PE=1 SV=2 - [PSB1_HUMAN] | 4 | 0 | 4 | 241 | 26.5 | 8.13 |
| Q9Y3D9 | 28S ribosomal protein S23, mitochondrial OS=Homo sapiens GN=MRPS23 PE=1 SV=2 - [RT23_HUMAN] | 4 | 0 | 4 | 190 | 21.8 | 8.9 |
| P30519 | Heme oxygenase 2 OS=Homo sapiens GN=HMOX2 PE=1 SV=2 - [HMOX2_HUMAN] | 4 | 0 | 4 | 316 | 36 | 5.41 |
| P00374 | Dihydrofolate reductase OS=Homo sapiens GN=DHFR PE=1 SV=2 - [DYR_HUMAN] | 4 | 0 | 4 | 187 | 21.4 | 7.42 |
| P15170 | Eukaryotic peptide chain release factor GTP-binding subunit ERF3A OS=Homo sapiens GN=GSPT1 PE=1 SV=1 - [ERF3A_HUMAN] | 4 | 0 | 4 | 499 | 55.7 | 5.62 |
| Q8IVD9 | NudC domain-containing protein 3 OS=Homo sapiens GN=NUDCD3 PE=1 SV=3 - [NUDC3_HUMAN] | 4 | 0 | 4 | 361 | 40.8 | 5.25 |
| Q15904 | V-type proton ATPase subunit S1 OS=Homo sapiens GN=ATP6AP1 PE=1 SV=2 - [VAS1_HUMAN] | 4 | 0 | 4 | 470 | 52 | 6.14 |
| Q9Y3Z3 | SAM domain and HD domain-containing protein 1 OS=Homo sapiens GN=SAMHD1 PE=1 SV=2 - [SAMH1_HUMAN] | 4 | 0 | 4 | 626 | 72.2 | 7.14 |
| Q9Y6G9 | Cytoplasmic dynein 1 light intermediate chain 1 OS=Homo sapiens GN=DYNC1LI1 PE=1 SV=3 - [DC1L1_HUMAN] | 4 | 0 | 4 | 523 | 56.5 | 6.42 |
| Q8IVL6 | Prolyl 3-hydroxylase 3 OS=Homo sapiens GN=LEPREL2 PE=2 SV=1 - [P3H3_HUMAN] | 4 | 0 | 4 | 736 | 81.8 | 6.32 |
| Q68E01 | Integrator complex subunit 3 OS=Homo sapiens GN=INTS3 PE=1 SV=1 - [INT3_HUMAN] | 4 | 0 | 4 | 1043 | 118 | 5.8 |
| Q9H6T3 | RNA polymerase II-associated protein 3 OS=Homo sapiens GN=RPAP3 PE=1 SV=2 - [RPAP3_HUMAN] | 4 | 0 | 4 | 665 | 75.7 | 6.84 |
| Q9H307 | Pinin OS=Homo sapiens GN=PNN PE=1 SV=4 - [PININ_HUMAN] | 4 | 0 | 4 | 717 | 81.6 | 7.14 |
| P11802 | Cell division protein kinase 4 OS=Homo sapiens GN=CDK4 PE=1 SV=2 - [CDK4_HUMAN] | 2 | 0 | 4 | 303 | 33.7 | 7.01 |
| P01111 | GTPase NRas OS=Homo sapiens GN=NRAS PE=1 SV=1 - [RASN_HUMAN] | 3 | 0 | 3 | 189 | 21.2 | 5.17 |
| Q9NP72 | Ras-related protein Rab-18 OS=Homo sapiens GN=RAB18 PE=1 SV=1 - [RAB18_HUMAN] | 3 | 0 | 3 | 206 | 23 | 5.24 |
| O94811 | Tubulin polymerization-promoting protein OS=Homo sapiens GN=TPPP PE=1 SV=1 - [TPPP_HUMAN] | 3 | 0 | 3 | 219 | 23.7 | 9.44 |
| Q9BUL8 | Programmed cell death protein 10 OS=Homo sapiens GN=PDCD10 PE=1 SV=1 - [PDC10_HUMAN] | 3 | 0 | 3 | 212 | 24.7 | 8.19 |
| P61019 | Ras-related protein Rab-2A OS=Homo sapiens GN=RAB2A PE=1 SV=1 - [RAB2A_HUMAN] | 3 | 0 | 3 | 212 | 23.5 | 6.54 |
| Q96C19 | EF-hand domain-containing protein D2 OS=Homo sapiens GN=EFHD2 PE=1 SV=1 - [EFHD2_HUMAN] | 3 | 0 | 3 | 240 | 26.7 | 5.2 |
| Q9BUR5 | MICOS complex subunit MIC26 OS=Homo sapiens GN=APOO PE=1 SV=1 - [MIC26_HUMAN] | 3 | 0 | 3 | 198 | 22.3 | 9.13 |
| Q9Y3B8 | Oligoribonuclease, mitochondrial OS=Homo sapiens GN=REXO2 PE=1 SV=3 - [ORN_HUMAN] | 3 | 0 | 3 | 237 | 26.8 | 6.87 |
| Q9BX40 | Protein LSM14 homolog B OS=Homo sapiens GN=LSM14B PE=1 SV=1 - [LS14B_HUMAN] | 3 | 0 | 3 | 385 | 42 | 9.69 |
| O75569 | Interferon-inducible double stranded RNA-dependent protein kinase activator A OS=Homo sapiens GN=PRKRA PE=1 SV=1 - [PRKRA_HUMAN] | 3 | 0 | 3 | 313 | 34.4 | 8.41 |
| P09543 | 2',3'-cyclic-nucleotide 3'-phosphodiesterase OS=Homo sapiens GN=CNP PE=1 SV=2 - [CN37_HUMAN] | 3 | 0 | 3 | 421 | 47.5 | 9.07 |
| Q12849 | G-rich sequence factor 1 OS=Homo sapiens GN=GRSF1 PE=1 SV=3 - [GRSF1_HUMAN] | 3 | 0 | 3 | 480 | 53.1 | 6.19 |
| Q9BXK5 | Bcl-2-like protein 13 OS=Homo sapiens GN=BCL2L13 PE=1 SV=1 - [B2L13_HUMAN] | 3 | 0 | 3 | 485 | 52.7 | 4.44 |
| P49821 | NADH dehydrogenase [ubiquinone] flavoprotein 1, mitochondrial OS=Homo sapiens GN=NDUFV1 PE=1 SV=4 - [NDUV1_HUMAN] | 3 | 0 | 3 | 464 | 50.8 | 8.21 |
| Q92791 | Synaptonemal complex protein SC65 OS=Homo sapiens GN=SC65 PE=1 SV=1 - [SC65_HUMAN] | 3 | 0 | 3 | 437 | 50.3 | 4.77 |
| Q96TC7 | Regulator of microtubule dynamics protein 3 OS=Homo sapiens GN=FAM82A2 PE=1 SV=2 - [RMD3_HUMAN] | 3 | 0 | 3 | 470 | 52.1 | 5.1 |
| O00566 | U3 small nucleolar ribonucleoprotein protein MPP10 OS=Homo sapiens GN=MPHOSPH10 PE=1 SV=2 - [MPP10_HUMAN] | 3 | 0 | 3 | 681 | 78.8 | 4.86 |
| Q14738 | Serine/threonine-protein phosphatase 2A 56 kDa regulatory subunit delta isoform OS=Homo sapiens GN=PPP2R5D PE=1 SV=1 - [2A5D_HUMAN] | 3 | 0 | 3 | 602 | 69.9 | 8.13 |
| P06865 | Beta-hexosaminidase subunit alpha OS=Homo sapiens GN=HEXA PE=1 SV=2 - [HEXA_HUMAN] | 3 | 0 | 3 | 529 | 60.7 | 5.16 |
| Q9NZT2 | Opioid growth factor receptor OS=Homo sapiens GN=OGFR PE=1 SV=3 - [OGFR_HUMAN] | 3 | 0 | 3 | 677 | 73.3 | 4.84 |
| Q9Y6A5 | Transforming acidic coiled-coil-containing protein 3 OS=Homo sapiens GN=TACC3 PE=1 SV=1 - [TACC3_HUMAN] | 3 | 0 | 3 | 838 | 90.3 | 5.05 |
| O14974 | Protein phosphatase 1 regulatory subunit 12A OS=Homo sapiens GN=PPP1R12A PE=1 SV=1 - [MYPT1_HUMAN] | 3 | 0 | 3 | 1030 | 115.2 | 5.4 |
| P49589 | Cysteinyl-tRNA synthetase, cytoplasmic OS=Homo sapiens GN=CARS PE=1 SV=3 - [SYCC_HUMAN] | 3 | 0 | 3 | 748 | 85.4 | 6.76 |
| Q8IX12 | Cell division cycle and apoptosis regulator protein 1 OS=Homo sapiens GN=CCAR1 PE=1 SV=2 - [CCAR1_HUMAN] | 3 | 0 | 3 | 1150 | 132.7 | 5.76 |
| Q9NRL2 | Bromodomain adjacent to zinc finger domain protein 1A OS=Homo sapiens GN=BAZ1A PE=1 SV=2 - [BAZ1A_HUMAN] | 3 | 0 | 3 | 1556 | 178.6 | 6.6 |
| Q9NTZ6 | RNA-binding protein 12 OS=Homo sapiens GN=RBM12 PE=1 SV=1 - [RBM12_HUMAN] | 3 | 0 | 3 | 932 | 97.3 | 8.63 |
| O43169 | Cytochrome b5 type B OS=Homo sapiens GN=CYB5B PE=1 SV=2 - [CYB5B_HUMAN] | 2 | 0 | 3 | 146 | 16.3 | 4.97 |
| Q9NRX4 | 14 kDa phosphohistidine phosphatase OS=Homo sapiens GN=PHPT1 PE=1 SV=1 - [PHP14_HUMAN] | 2 | 0 | 3 | 125 | 13.8 | 6.07 |
| Q14011 | Cold-inducible RNA-binding protein OS=Homo sapiens GN=CIRBP PE=1 SV=1 - [CIRBP_HUMAN] | 2 | 0 | 3 | 172 | 18.6 | 9.51 |
| O14602 | Eukaryotic translation initiation factor 1A, Y-chromosomal OS=Homo sapiens GN=EIF1AY PE=1 SV=4 - [IF1AY_HUMAN] | 2 | 0 | 3 | 144 | 16.4 | 5.24 |
| Q9NX40 | OCIA domain-containing protein 1 OS=Homo sapiens GN=OCIAD1 PE=1 SV=1 - [OCAD1_HUMAN] | 2 | 0 | 3 | 245 | 27.6 | 7.49 |
| O15160 | DNA-directed RNA polymerases I and III subunit RPAC1 OS=Homo sapiens GN=POLR1C PE=1 SV=1 - [RPAC1_HUMAN] | 2 | 0 | 3 | 346 | 39.2 | 5.5 |
| Q9HD33 | 39S ribosomal protein L47, mitochondrial OS=Homo sapiens GN=MRPL47 PE=1 SV=2 - [RM47_HUMAN] | 2 | 0 | 3 | 250 | 29.4 | 10.37 |
| Q9C0H2 | Protein tweety homolog 3 OS=Homo sapiens GN=TTYH3 PE=1 SV=3 - [TTYH3_HUMAN] | 2 | 0 | 3 | 523 | 57.5 | 5.39 |
| Q92783 | Signal transducing adapter molecule 1 OS=Homo sapiens GN=STAM PE=1 SV=3 - [STAM1_HUMAN] | 2 | 0 | 3 | 540 | 59.1 | 4.82 |
| Q9UK76 | Hematological and neurological expressed 1 protein OS=Homo sapiens GN=HN1 PE=1 SV=3 - [HN1_HUMAN] | 2 | 0 | 2 | 154 | 16 | 5.6 |
| O00483 | NADH dehydrogenase [ubiquinone] 1 alpha subcomplex subunit 4 OS=Homo sapiens GN=NDUFA4 PE=1 SV=1 - [NDUA4_HUMAN] | 2 | 0 | 2 | 81 | 9.4 | 9.38 |
| Q9NWV4 | UPF0587 protein C1orf123 OS=Homo sapiens GN=C1orf123 PE=1 SV=1 - [CA123_HUMAN] | 2 | 0 | 2 | 160 | 18 | 5.01 |
| Q9UL45 | Biogenesis of lysosome-related organelles complex 1 subunit 6 OS=Homo sapiens GN=BLOC1S6 PE=1 SV=1 - [BL1S6_HUMAN] | 2 | 0 | 2 | 172 | 19.7 | 6.4 |
| P61970 | Nuclear transport factor 2 OS=Homo sapiens GN=NUTF2 PE=1 SV=1 - [NTF2_HUMAN] | 2 | 0 | 2 | 127 | 14.5 | 5.38 |
| Q8N183 | Mimitin, mitochondrial OS=Homo sapiens GN=NDUFAF2 PE=1 SV=1 - [MIMIT_HUMAN] | 2 | 0 | 2 | 169 | 19.8 | 8.97 |
| Q9Y3B4 | Pre-mRNA branch site protein p14 OS=Homo sapiens GN=SF3B14 PE=1 SV=1 - [PM14_HUMAN] | 2 | 0 | 2 | 125 | 14.6 | 9.38 |
| Q9NX14 | NADH dehydrogenase [ubiquinone] 1 beta subcomplex subunit 11, mitochondrial OS=Homo sapiens GN=NDUFB11 PE=1 SV=1 - [NDUBB_HUMAN] | 2 | 0 | 2 | 153 | 17.3 | 5.22 |
| Q9UI09 | NADH dehydrogenase [ubiquinone] 1 alpha subcomplex subunit 12 OS=Homo sapiens GN=NDUFA12 PE=1 SV=1 - [NDUAC_HUMAN] | 2 | 0 | 2 | 145 | 17.1 | 9.63 |
| Q8N5M4 | Tetratricopeptide repeat protein 9C OS=Homo sapiens GN=TTC9C PE=1 SV=1 - [TTC9C_HUMAN] | 2 | 0 | 2 | 171 | 20 | 8.92 |
| P20674 | Cytochrome c oxidase subunit 5A, mitochondrial OS=Homo sapiens GN=COX5A PE=1 SV=2 - [COX5A_HUMAN] | 2 | 0 | 2 | 150 | 16.8 | 6.79 |
| Q96B26 | Exosome complex component RRP43 OS=Homo sapiens GN=EXOSC8 PE=1 SV=1 - [EXOS8_HUMAN] | 2 | 0 | 2 | 276 | 30 | 5.3 |
| P62256 | Ubiquitin-conjugating enzyme E2 H OS=Homo sapiens GN=UBE2H PE=1 SV=1 - [UBE2H_HUMAN] | 2 | 0 | 2 | 183 | 20.6 | 4.67 |
| Q8TAE8 | Growth arrest and DNA damage-inducible proteins-interacting protein 1 OS=Homo sapiens GN=GADD45GIP1 PE=1 SV=1 - [G45IP_HUMAN] | 2 | 0 | 2 | 222 | 25.4 | 10.02 |
| Q9NPA0 | UPF0480 protein C15orf24 OS=Homo sapiens GN=C15orf24 PE=1 SV=1 - [CO024_HUMAN] | 2 | 0 | 2 | 242 | 26.5 | 9.25 |
| Q9Y3E1 | Hepatoma-derived growth factor-related protein 3 OS=Homo sapiens GN=HDGFRP3 PE=1 SV=1 - [HDGR3_HUMAN] | 2 | 0 | 2 | 203 | 22.6 | 7.99 |
| Q9NV31 | U3 small nucleolar ribonucleoprotein protein IMP3 OS=Homo sapiens GN=IMP3 PE=1 SV=1 - [IMP3_HUMAN] | 2 | 0 | 2 | 184 | 21.8 | 9.5 |
| P13984 | General transcription factor IIF subunit 2 OS=Homo sapiens GN=GTF2F2 PE=1 SV=2 - [T2FB_HUMAN] | 2 | 0 | 2 | 249 | 28.4 | 9.23 |
| Q86X83 | COMM domain-containing protein 2 OS=Homo sapiens GN=COMMD2 PE=1 SV=2 - [COMD2_HUMAN] | 2 | 0 | 2 | 199 | 22.7 | 6.73 |
| Q9Y3A3 | MOB-like protein phocein OS=Homo sapiens GN=MOB4 PE=1 SV=1 - [PHOCN_HUMAN] | 2 | 0 | 2 | 225 | 26 | 5.78 |
| Q9NRX1 | RNA-binding protein PNO1 OS=Homo sapiens GN=PNO1 PE=1 SV=1 - [PNO1_HUMAN] | 2 | 0 | 2 | 252 | 27.9 | 9.73 |
| P52815 | 39S ribosomal protein L12, mitochondrial OS=Homo sapiens GN=MRPL12 PE=1 SV=2 - [RM12_HUMAN] | 2 | 0 | 2 | 198 | 21.3 | 8.87 |
| O95169 | NADH dehydrogenase [ubiquinone] 1 beta subcomplex subunit 8, mitochondrial OS=Homo sapiens GN=NDUFB8 PE=1 SV=1 - [NDUB8_HUMAN] | 2 | 0 | 2 | 186 | 21.8 | 6.8 |
| O95571 | Persulfide dioxygenase ETHE1, mitochondrial OS=Homo sapiens GN=ETHE1 PE=1 SV=2 - [ETHE1_HUMAN] | 2 | 0 | 2 | 254 | 27.9 | 6.83 |
| Q92520 | Protein FAM3C OS=Homo sapiens GN=FAM3C PE=1 SV=1 - [FAM3C_HUMAN] | 2 | 0 | 2 | 227 | 24.7 | 8.29 |
| Q9GZZ9 | Ubiquitin-like modifier-activating enzyme 5 OS=Homo sapiens GN=UBA5 PE=1 SV=1 - [UBA5_HUMAN] | 2 | 0 | 2 | 404 | 44.8 | 4.84 |
| P11766 | Alcohol dehydrogenase class-3 OS=Homo sapiens GN=ADH5 PE=1 SV=4 - [ADHX_HUMAN] | 2 | 0 | 2 | 374 | 39.7 | 7.49 |
| Q96DI7 | U5 small nuclear ribonucleoprotein 40 kDa protein OS=Homo sapiens GN=SNRNP40 PE=1 SV=1 - [SNR40_HUMAN] | 2 | 0 | 2 | 357 | 39.3 | 8.1 |
| P55081 | Microfibrillar-associated protein 1 OS=Homo sapiens GN=MFAP1 PE=1 SV=2 - [MFAP1_HUMAN] | 2 | 0 | 2 | 439 | 51.9 | 4.98 |
| Q92600 | Cell differentiation protein RCD1 homolog OS=Homo sapiens GN=RQCD1 PE=1 SV=1 - [RCD1_HUMAN] | 2 | 0 | 2 | 299 | 33.6 | 8.03 |
| Q9BYD3 | 39S ribosomal protein L4, mitochondrial OS=Homo sapiens GN=MRPL4 PE=1 SV=1 - [RM04_HUMAN] | 2 | 0 | 2 | 311 | 34.9 | 9.72 |
| Q7Z4H3 | HD domain-containing protein 2 OS=Homo sapiens GN=HDDC2 PE=1 SV=1 - [HDDC2_HUMAN] | 2 | 0 | 2 | 204 | 23.4 | 5.49 |
| Q9C0F1 | Centrosomal protein of 44 kDa OS=Homo sapiens GN=CEP44 PE=1 SV=2 - [CEP44_HUMAN] | 2 | 0 | 2 | 390 | 44.1 | 5.21 |
| Q8N9N7 | Leucine-rich repeat-containing protein 57 OS=Homo sapiens GN=LRRC57 PE=1 SV=1 - [LRC57_HUMAN] | 2 | 0 | 2 | 239 | 26.7 | 8.43 |
| Q13445 | Transmembrane emp24 domain-containing protein 1 OS=Homo sapiens GN=TMED1 PE=1 SV=1 - [TMED1_HUMAN] | 2 | 0 | 2 | 227 | 25.2 | 4.48 |
| Q8NBN7 | Retinol dehydrogenase 13 OS=Homo sapiens GN=RDH13 PE=1 SV=2 - [RDH13_HUMAN] | 2 | 0 | 2 | 331 | 35.9 | 8.1 |
| Q9NVS9 | Pyridoxine-5'-phosphate oxidase OS=Homo sapiens GN=PNPO PE=1 SV=1 - [PNPO_HUMAN] | 2 | 0 | 2 | 261 | 30 | 7.06 |
| Q9Y316 | Protein MEMO1 OS=Homo sapiens GN=MEMO1 PE=1 SV=1 - [MEMO1_HUMAN] | 2 | 0 | 2 | 297 | 33.7 | 7.14 |
| O00233 | 26S proteasome non-ATPase regulatory subunit 9 OS=Homo sapiens GN=PSMD9 PE=1 SV=3 - [PSMD9_HUMAN] | 2 | 0 | 2 | 223 | 24.7 | 6.95 |
| P55145 | Mesencephalic astrocyte-derived neurotrophic factor OS=Homo sapiens GN=MANF PE=1 SV=3 - [MANF_HUMAN] | 2 | 0 | 2 | 182 | 20.7 | 8.69 |
| Q92733 | Proline-rich protein PRCC OS=Homo sapiens GN=PRCC PE=1 SV=1 - [PRCC_HUMAN] | 2 | 0 | 2 | 491 | 52.4 | 5.1 |
| P61962 | DDB1- and CUL4-associated factor 7 OS=Homo sapiens GN=DCAF7 PE=1 SV=1 - [DCAF7_HUMAN] | 2 | 0 | 2 | 342 | 38.9 | 5.52 |
| Q00059 | Transcription factor A, mitochondrial OS=Homo sapiens GN=TFAM PE=1 SV=1 - [TFAM_HUMAN] | 2 | 0 | 2 | 246 | 29.1 | 9.72 |
| Q07960 | Rho GTPase-activating protein 1 OS=Homo sapiens GN=ARHGAP1 PE=1 SV=1 - [RHG01_HUMAN] | 2 | 0 | 2 | 439 | 50.4 | 6.29 |
| Q9H6V9 | UPF0554 protein C2orf43 OS=Homo sapiens GN=C2orf43 PE=1 SV=1 - [CB043_HUMAN] | 2 | 0 | 2 | 325 | 37.3 | 6.54 |
| P29084 | Transcription initiation factor IIE subunit beta OS=Homo sapiens GN=GTF2E2 PE=1 SV=1 - [T2EB_HUMAN] | 2 | 0 | 2 | 291 | 33 | 9.66 |
| O75063 | Protein FAM20B OS=Homo sapiens GN=FAM20B PE=2 SV=1 - [FA20B_HUMAN] | 2 | 0 | 2 | 409 | 46.4 | 6.87 |
| Q99436 | Proteasome subunit beta type-7 OS=Homo sapiens GN=PSMB7 PE=1 SV=1 - [PSB7_HUMAN] | 2 | 0 | 2 | 277 | 29.9 | 7.68 |
| Q13642 | Four and a half LIM domains protein 1 OS=Homo sapiens GN=FHL1 PE=1 SV=4 - [FHL1_HUMAN] | 2 | 0 | 2 | 323 | 36.2 | 8.97 |
| Q6NVY1 | 3-hydroxyisobutyryl-CoA hydrolase, mitochondrial OS=Homo sapiens GN=HIBCH PE=1 SV=2 - [HIBCH_HUMAN] | 2 | 0 | 2 | 386 | 43.5 | 8.19 |
| Q05048 | Cleavage stimulation factor subunit 1 OS=Homo sapiens GN=CSTF1 PE=1 SV=1 - [CSTF1_HUMAN] | 2 | 0 | 2 | 431 | 48.3 | 6.58 |
| Q9Y6W5 | Wiskott-Aldrich syndrome protein family member 2 OS=Homo sapiens GN=WASF2 PE=1 SV=3 - [WASF2_HUMAN] | 2 | 0 | 2 | 498 | 54.3 | 5.53 |
| O43148 | mRNA cap guanine-N7 methyltransferase OS=Homo sapiens GN=RNMT PE=1 SV=1 - [MCES_HUMAN] | 2 | 0 | 2 | 476 | 54.8 | 6.61 |
| Q7Z417 | Nuclear fragile X mental retardation-interacting protein 2 OS=Homo sapiens GN=NUFIP2 PE=1 SV=1 - [NUFP2_HUMAN] | 2 | 0 | 2 | 695 | 76.1 | 8.7 |
| Q5F1R6 | DnaJ homolog subfamily C member 21 OS=Homo sapiens GN=DNAJC21 PE=1 SV=2 - [DJC21_HUMAN] | 2 | 0 | 2 | 531 | 62 | 5.47 |
| Q06546 | GA-binding protein alpha chain OS=Homo sapiens GN=GABPA PE=1 SV=1 - [GABPA_HUMAN] | 2 | 0 | 2 | 454 | 51.3 | 4.97 |
| Q9UBL3 | Set1/Ash2 histone methyltransferase complex subunit ASH2 OS=Homo sapiens GN=ASH2L PE=1 SV=1 - [ASH2L_HUMAN] | 2 | 0 | 2 | 628 | 68.7 | 5.69 |
| Q9H0C8 | Integrin-linked kinase-associated serine/threonine phosphatase 2C OS=Homo sapiens GN=ILKAP PE=1 SV=1 - [ILKAP_HUMAN] | 2 | 0 | 2 | 392 | 42.9 | 7.09 |
| Q8IUF8 | Bifunctional lysine-specific demethylase and histidyl-hydroxylase MINA OS=Homo sapiens GN=MINA PE=1 SV=1 - [MINA_HUMAN] | 2 | 0 | 2 | 465 | 52.8 | 6.7 |
| Q9GZT8 | NIF3-like protein 1 OS=Homo sapiens GN=NIF3L1 PE=1 SV=2 - [NIF3L_HUMAN] | 2 | 0 | 2 | 377 | 41.9 | 6.65 |
| Q13685 | Angio-associated migratory cell protein OS=Homo sapiens GN=AAMP PE=1 SV=2 - [AAMP_HUMAN] | 2 | 0 | 2 | 434 | 46.7 | 4.42 |
| P10619 | Lysosomal protective protein OS=Homo sapiens GN=CTSA PE=1 SV=2 - [PPGB_HUMAN] | 2 | 0 | 2 | 480 | 54.4 | 6.61 |
| Q8N6H7 | ADP-ribosylation factor GTPase-activating protein 2 OS=Homo sapiens GN=ARFGAP2 PE=1 SV=1 - [ARFG2_HUMAN] | 2 | 0 | 2 | 521 | 56.7 | 7.99 |
| Q3KQV9 | UDP-N-acetylhexosamine pyrophosphorylase-like protein 1 OS=Homo sapiens GN=UAP1L1 PE=2 SV=2 - [UAP1L_HUMAN] | 2 | 0 | 2 | 507 | 57 | 6.32 |
| Q9UH03 | Neuronal-specific septin-3 OS=Homo sapiens GN=SEPT3 PE=1 SV=3 - [SEPT3_HUMAN] | 2 | 0 | 2 | 358 | 40.7 | 7.2 |
| Q96NU1 | Sterile alpha motif domain-containing protein 11 OS=Homo sapiens GN=SAMD11 PE=1 SV=3 - [SAM11_HUMAN] | 2 | 0 | 2 | 681 | 72.7 | 7.59 |
| P49643 | DNA primase large subunit OS=Homo sapiens GN=PRIM2 PE=1 SV=2 - [PRI2_HUMAN] | 2 | 0 | 2 | 509 | 58.8 | 7.91 |
| Q9UHY1 | Nuclear receptor-binding protein OS=Homo sapiens GN=NRBP1 PE=1 SV=1 - [NRBP_HUMAN] | 2 | 0 | 2 | 535 | 59.8 | 5.08 |
| Q9BVL2 | Nucleoporin p58/p45 OS=Homo sapiens GN=NUP58 PE=1 SV=1 - [NUP58_HUMAN] | 2 | 0 | 2 | 599 | 60.9 | 9.33 |
| Q96G46 | tRNA-dihydrouridine synthase 3-like OS=Homo sapiens GN=DUS3L PE=1 SV=2 - [DUS3L_HUMAN] | 2 | 0 | 2 | 650 | 72.5 | 8.05 |
| Q6NUQ4 | Transmembrane protein 214 OS=Homo sapiens GN=TMEM214 PE=1 SV=2 - [TM214_HUMAN] | 2 | 0 | 2 | 689 | 77.1 | 9.14 |
| Q5T0N5 | Formin-binding protein 1-like OS=Homo sapiens GN=FNBP1L PE=1 SV=3 - [FBP1L_HUMAN] | 2 | 0 | 2 | 605 | 70 | 6.64 |
| Q7Z2K8 | G protein-regulated inducer of neurite outgrowth 1 OS=Homo sapiens GN=GPRIN1 PE=1 SV=2 - [GRIN1_HUMAN] | 2 | 0 | 2 | 1008 | 102.3 | 8.06 |
| Q9H501 | ESF1 homolog OS=Homo sapiens GN=ESF1 PE=1 SV=1 - [ESF1_HUMAN] | 2 | 0 | 2 | 851 | 98.7 | 5.11 |
| O00469 | Procollagen-lysine,2-oxoglutarate 5-dioxygenase 2 OS=Homo sapiens GN=PLOD2 PE=1 SV=2 - [PLOD2_HUMAN] | 2 | 0 | 2 | 737 | 84.6 | 6.71 |
| Q6IN85 | Serine/threonine-protein phosphatase 4 regulatory subunit 3A OS=Homo sapiens GN=SMEK1 PE=1 SV=1 - [P4R3A_HUMAN] | 2 | 0 | 2 | 833 | 95.3 | 4.94 |
| Q13618 | Cullin-3 OS=Homo sapiens GN=CUL3 PE=1 SV=2 - [CUL3_HUMAN] | 2 | 0 | 2 | 768 | 88.9 | 8.48 |
| Q9NR30 | Nucleolar RNA helicase 2 OS=Homo sapiens GN=DDX21 PE=1 SV=5 - [DDX21_HUMAN] | 2 | 0 | 2 | 783 | 87.3 | 9.28 |
| Q8IXK0 | Polyhomeotic-like protein 2 OS=Homo sapiens GN=PHC2 PE=1 SV=1 - [PHC2_HUMAN] | 2 | 0 | 2 | 858 | 90.7 | 8.69 |
| Q9BSJ8 | Extended synaptotagmin-1 OS=Homo sapiens GN=ESYT1 PE=1 SV=1 - [ESYT1_HUMAN] | 2 | 0 | 2 | 1104 | 122.8 | 5.83 |
| Q9UJ41 | Rab5 GDP/GTP exchange factor OS=Homo sapiens GN=RABGEF1 PE=1 SV=2 - [RABX5_HUMAN] | 2 | 0 | 2 | 708 | 79.3 | 6.81 |
| Q5VT52 | Regulation of nuclear pre-mRNA domain-containing protein 2 OS=Homo sapiens GN=RPRD2 PE=1 SV=1 - [RPRD2_HUMAN] | 2 | 0 | 2 | 1461 | 155.9 | 7.42 |
| O60502 | Bifunctional protein NCOAT OS=Homo sapiens GN=MGEA5 PE=1 SV=2 - [NCOAT_HUMAN] | 2 | 0 | 2 | 916 | 102.8 | 4.91 |
| Q9UDY2 | Tight junction protein ZO-2 OS=Homo sapiens GN=TJP2 PE=1 SV=2 - [ZO2_HUMAN] | 2 | 0 | 2 | 1190 | 133.9 | 7.4 |
| Q9NYU2 | UDP-glucose:glycoprotein glucosyltransferase 1 OS=Homo sapiens GN=UGGT1 PE=1 SV=3 - [UGGG1_HUMAN] | 2 | 0 | 2 | 1555 | 177.1 | 5.63 |
| P78527 | DNA-dependent protein kinase catalytic subunit OS=Homo sapiens GN=PRKDC PE=1 SV=3 - [PRKDC_HUMAN] | 2 | 0 | 2 | 4128 | 468.8 | 7.12 |

**Supplementary Table 4:** The list of processes and/or pathways involved in proteins regulation in UKF-NB-4 cells (*hMT3* vs. mock) using Gene Ontology (GO) annotations and KEGG 10 software.

| **pathway ID** | **pathway description** | **Observed proteins count** | **False discovery rate** |
| --- | --- | --- | --- |
| GO:0048519 | negative regulation of biological process | 184 | 0.004 |
| GO:0006810 | transport | 176 | 5.33E-06 |
| GO:0016070 | RNA metabolic process | 169 | 5.15E-10 |
| GO:0010033 | response to organic substance | 116 | 0.003 |
| GO:0022607 | cellular component assembly | 107 | 0.0001 |
| GO:0046907 | intracellular transport | 98 | 7.24E-13 |
| GO:0016192 | vesicle-mediated transport | 87 | 2.19E-05 |
| GO:0033554 | cellular response to stress | 77 | 0.0002 |
| GO:0006508 | proteolysis | 58 | 0.005 |
| GO:0008380 | RNA splicing | 52 | 1.58E-15 |
| GO:0006887 | exocytosis | 52 | 4.22E-06 |
| GO:0017144 | drug metabolic process | 49 | 1.17E-07 |
| GO:0061024 | membrane organization | 45 | 0.0001 |
| GO:0009894 | regulation of catabolic process | 45 | 0.003 |
| GO:0000278 | mitotic cell cycle | 44 | 1.24E-05 |
| GO:0010243 | response to organonitrogen compound | 44 | 0.001 |
| GO:0000398 | mRNA splicing, via spliceosome | 41 | 2.64E-13 |
| GO:0022613 | ribonucleoprotein complex biogenesis | 41 | 4.74E-09 |
| GO:0009161 | ribonucleoside monophosphate metabolic process | 37 | 1.09E-12 |
| GO:0007005 | mitochondrion organization | 35 | 6.73E-06 |
| GO:0006605 | protein targeting | 29 | 1.15E-05 |
| GO:0046034 | ATP metabolic process | 28 | 2.32E-09 |
| GO:0006979 | response to oxidative stress | 24 | 0.009 |
| GO:0048193 | Golgi vesicle transport | 22 | 0.008 |
| GO:0006119 | oxidative phosphorylation | 18 | 3.56E-07 |
| GO:0050658 | RNA transport | 16 | 0.005 |
| GO:0022904 | respiratory electron transport chain | 15 | 2.32E-05 |
| GO:2001242 | regulation of intrinsic apoptotic signaling pathway | 12 | 0.004 |
| GO:0048024 | regulation of mRNA splicing, via spliceosome | 11 | 0.001 |
| GO:0006900 | vesicle budding from membrane | 11 | 0.002 |
| GO:1990748 | cellular detoxification | 11 | 0.003 |
| GO:0009060 | aerobic respiration | 9 | 0.008 |
| GO:0070125 | mitochondrial translational | 9 | 0.0028 |
| GO:0006749 | glutathione metabolic process | 8 | 0.006 |
| GO:0006120 | mitochondrial electron transport, NADH to ubiquinone | 7 | 0.01 |
| GO:0061077 | chaperone-mediated protein folding | 7 | 0.004 |
| GO:0006735 | NADH regeneration | 6 | 0.003 |
| GO:0061718 | glucose catabolic process to pyruvate | 6 | 0.001 |
| GO:0042866 | pyruvate biosynthetic process | 6 | 0.003 |
| GO:0006094 | gluconeogenesis | 6 | 0.004 |
| GO:0000305 | response to oxygen radical | 5 | 0.01 |
| GO:0034982 | mitochondrial protein processing | 4 | 0.0096 |
| GO:0019430 | removal of superoxide radicals | 4 | 0.001 |

**Supplementary Table 5:** A full list of proteins detected for *mock* and *hMT3* overexpressed in UKF-NB-4 cell line in comparison with UKF-NB-4^CDDP^ cell line.

| Accession | Description | PSM | PSM | PSM |
| --- | --- | --- | --- | --- |
|  |  | UKF-NB-4^CDDP^ | UKF-NB-4 | UKF-NB-4 |
|  |  |  | *Mock* | *hMT3* |
| Q15084 | Protein disulfide-isomerase A6 OS=Homo sapiens GN=PDIA6 PE=1 SV=1 - [PDIA6_HUMAN] | 121 | 70 | 98 |
| P07900 | Heat shock protein HSP 90-alpha OS=Homo sapiens GN=HSP90AA1 PE=1 SV=5 - [HS90A_HUMAN] | 95 | 74 | 93 |
| P07437 | Tubulin beta chain OS=Homo sapiens GN=TUBB PE=1 SV=2 - [TBB5_HUMAN] | 108 | 79 | 89 |
| P11142 | Heat shock cognate 71 kDa protein OS=Homo sapiens GN=HSPA8 PE=1 SV=1 - [HSP7C_HUMAN] | 66 | 75 | 82 |
| P21333 | Filamin-A OS=Homo sapiens GN=FLNA PE=1 SV=4 - [FLNA_HUMAN] | 19 | 57 | 82 |
| P10809 | 60 kDa heat shock protein, mitochondrial OS=Homo sapiens GN=HSPD1 PE=1 SV=2 - [CH60_HUMAN] | 210 | 55 | 82 |
| P08238 | Heat shock protein HSP 90-beta OS=Homo sapiens GN=HSP90AB1 PE=1 SV=4 - [HS90B_HUMAN] | 85 | 64 | 79 |
| P06733 | Alpha-enolase OS=Homo sapiens GN=ENO1 PE=1 SV=2 - [ENOA_HUMAN] | 60 | 50 | 66 |
| P46821 | Microtubule-associated protein 1B OS=Homo sapiens GN=MAP1B PE=1 SV=2 - [MAP1B_HUMAN] | 35 | 43 | 59 |
| P11021 | 78 kDa glucose-regulated protein OS=Homo sapiens GN=HSPA5 PE=1 SV=2 - [GRP78_HUMAN] | 51 | 43 | 58 |
| P22314 | Ubiquitin-like modifier-activating enzyme 1 OS=Homo sapiens GN=UBA1 PE=1 SV=3 - [UBA1_HUMAN] | 37 | 35 | 52 |
| P14625 | Endoplasmin OS=Homo sapiens GN=HSP90B1 PE=1 SV=1 - [ENPL_HUMAN] | 50 | 36 | 51 |
| P68366 | Tubulin alpha-4A chain OS=Homo sapiens GN=TUBA4A PE=1 SV=1 - [TBA4A_HUMAN] | 54 | 47 | 49 |
| P42704 | Leucine-rich PPR motif-containing protein, mitochondrial OS=Homo sapiens GN=LRPPRC PE=1 SV=3 - [LPPRC_HUMAN] | 44 | 40 | 49 |
| Q6S8J3 | POTE ankyrin domain family member E OS=Homo sapiens GN=POTEE PE=1 SV=3 - [POTEE_HUMAN] | 32 | 30 | 49 |
| P04075 | Fructose-bisphosphate aldolase A OS=Homo sapiens GN=ALDOA PE=1 SV=2 - [ALDOA_HUMAN] | 27 | 28 | 48 |
| Q13509 | Tubulin beta-3 chain OS=Homo sapiens GN=TUBB3 PE=1 SV=2 - [TBB3_HUMAN] | 77 | 42 | 46 |
| P38646 | Stress-70 protein, mitochondrial OS=Homo sapiens GN=HSPA9 PE=1 SV=2 - [GRP75_HUMAN] | 44 | 34 | 43 |
| Q13813 | Spectrin alpha chain, brain OS=Homo sapiens GN=SPTAN1 PE=1 SV=3 - [SPTA2_HUMAN] | 25 | 23 | 43 |
| Q09666 | Neuroblast differentiation-associated protein AHNAK OS=Homo sapiens GN=AHNAK PE=1 SV=2 - [AHNK_HUMAN] | 1 | 37 | 42 |
| P19338 | Nucleolin OS=Homo sapiens GN=NCL PE=1 SV=3 - [NUCL_HUMAN] | 29 | 29 | 40 |
| P04406 | Glyceraldehyde-3-phosphate dehydrogenase OS=Homo sapiens GN=GAPDH PE=1 SV=3 - [G3P_HUMAN] | 36 | 24 | 38 |
| P60174 | Triosephosphate isomerase OS=Homo sapiens GN=TPI1 PE=1 SV=3 - [TPIS_HUMAN] | 30 | 27 | 37 |
| P30101 | Protein disulfide-isomerase A3 OS=Homo sapiens GN=PDIA3 PE=1 SV=4 - [PDIA3_HUMAN] | 40 | 26 | 37 |
| P78371 | T-complex protein 1 subunit beta OS=Homo sapiens GN=CCT2 PE=1 SV=4 - [TCPB_HUMAN] | 47 | 28 | 36 |
| P13639 | Elongation factor 2 OS=Homo sapiens GN=EEF2 PE=1 SV=4 - [EF2_HUMAN] | 47 | 26 | 36 |
| Q13263 | Transcription intermediary factor 1-beta OS=Homo sapiens GN=TRIM28 PE=1 SV=5 - [TIF1B_HUMAN] | 12 | 24 | 36 |
| P08670 | Vimentin OS=Homo sapiens GN=VIM PE=1 SV=4 - [VIME_HUMAN] | 34 | 31 | 35 |
| Q9Y4L1 | Hypoxia up-regulated protein 1 OS=Homo sapiens GN=HYOU1 PE=1 SV=1 - [HYOU1_HUMAN] | 21 | 25 | 34 |
| Q14974 | Importin subunit beta-1 OS=Homo sapiens GN=KPNB1 PE=1 SV=2 - [IMB1_HUMAN] | 37 | 33 | 33 |
| P30153 | Serine/threonine-protein phosphatase 2A 65 kDa regulatory subunit A alpha isoform OS=Homo sapiens GN=PPP2R1A PE=1 SV=4 - [2AAA_HUMAN] | 28 | 23 | 33 |
| P00558 | Phosphoglycerate kinase 1 OS=Homo sapiens GN=PGK1 PE=1 SV=3 - [PGK1_HUMAN] | 27 | 29 | 32 |
| P06576 | ATP synthase subunit beta, mitochondrial OS=Homo sapiens GN=ATP5B PE=1 SV=3 - [ATPB_HUMAN] | 38 | 26 | 31 |
| O43707 | Alpha-actinin-4 OS=Homo sapiens GN=ACTN4 PE=1 SV=2 - [ACTN4_HUMAN] | 21 | 26 | 31 |
| P55072 | Transitional endoplasmic reticulum ATPase OS=Homo sapiens GN=VCP PE=1 SV=4 - [TERA_HUMAN] | 31 | 27 | 30 |
| P48681 | Nestin OS=Homo sapiens GN=NES PE=1 SV=2 - [NEST_HUMAN] | 15 | 27 | 30 |
| P07237 | Protein disulfide-isomerase OS=Homo sapiens GN=P4HB PE=1 SV=3 - [PDIA1_HUMAN] | 26 | 24 | 30 |
| P61978 | Heterogeneous nuclear ribonucleoprotein K OS=Homo sapiens GN=HNRNPK PE=1 SV=1 - [HNRPK_HUMAN] | 33 | 23 | 30 |
| O43175 | D-3-phosphoglycerate dehydrogenase OS=Homo sapiens GN=PHGDH PE=1 SV=4 - [SERA_HUMAN] | 25 | 21 | 30 |
| P63104 | 14-3-3 protein zeta/delta OS=Homo sapiens GN=YWHAZ PE=1 SV=1 - [1433Z_HUMAN] | 27 | 29 | 29 |
| Q14315 | Filamin-C OS=Homo sapiens GN=FLNC PE=1 SV=3 - [FLNC_HUMAN] | 11 | 21 | 29 |
| P09651 | Heterogeneous nuclear ribonucleoprotein A1 OS=Homo sapiens GN=HNRNPA1 PE=1 SV=5 - [ROA1_HUMAN] | 31 | 19 | 29 |
| P12814 | Alpha-actinin-1 OS=Homo sapiens GN=ACTN1 PE=1 SV=2 - [ACTN1_HUMAN] | 24 | 17 | 29 |
| P22626 | Heterogeneous nuclear ribonucleoproteins A2/B1 OS=Homo sapiens GN=HNRNPA2B1 PE=1 SV=2 - [ROA2_HUMAN] | 36 | 15 | 29 |
| Q01082 | Spectrin beta chain, brain 1 OS=Homo sapiens GN=SPTBN1 PE=1 SV=2 - [SPTB2_HUMAN] | 5 | 14 | 29 |
| Q9BUF5 | Tubulin beta-6 chain OS=Homo sapiens GN=TUBB6 PE=1 SV=1 - [TBB6_HUMAN] | 51 | 28 | 28 |
| P08107 | Heat shock 70 kDa protein 1A/1B OS=Homo sapiens GN=HSPA1A PE=1 SV=5 - [HSP71_HUMAN] | 31 | 27 | 28 |
| Q16555 | Dihydropyrimidinase-related protein 2 OS=Homo sapiens GN=DPYSL2 PE=1 SV=1 - [DPYL2_HUMAN] | 22 | 26 | 28 |
| P31939 | Bifunctional purine biosynthesis protein PURH OS=Homo sapiens GN=ATIC PE=1 SV=3 - [PUR9_HUMAN] | 27 | 22 | 28 |
| P07195 | L-lactate dehydrogenase B chain OS=Homo sapiens GN=LDHB PE=1 SV=2 - [LDHB_HUMAN] | 25 | 22 | 28 |
| Q92499 | ATP-dependent RNA helicase DDX1 OS=Homo sapiens GN=DDX1 PE=1 SV=2 - [DDX1_HUMAN] | 28 | 20 | 28 |
| Q14980 | Nuclear mitotic apparatus protein 1 OS=Homo sapiens GN=NUMA1 PE=1 SV=2 - [NUMA1_HUMAN] | 24 | 19 | 28 |
| P20700 | Lamin-B1 OS=Homo sapiens GN=LMNB1 PE=1 SV=2 - [LMNB1_HUMAN] | 18 | 17 | 28 |
| P07355 | Annexin A2 OS=Homo sapiens GN=ANXA2 PE=1 SV=2 - [ANXA2_HUMAN] | 24 | 32 | 27 |
| P08133 | Annexin A6 OS=Homo sapiens GN=ANXA6 PE=1 SV=3 - [ANXA6_HUMAN] | 5 | 21 | 27 |
| P52292 | Importin subunit alpha-2 OS=Homo sapiens GN=KPNA2 PE=1 SV=1 - [IMA2_HUMAN] | 28 | 18 | 27 |
| P49321 | Nuclear autoantigenic sperm protein OS=Homo sapiens GN=NASP PE=1 SV=2 - [NASP_HUMAN] | 8 | 16 | 27 |
| P14618 | Pyruvate kinase isozymes M1/M2 OS=Homo sapiens GN=PKM2 PE=1 SV=4 - [KPYM_HUMAN] | 28 | 28 | 26 |
| P27348 | 14-3-3 protein theta OS=Homo sapiens GN=YWHAQ PE=1 SV=1 - [1433T_HUMAN] | 24 | 23 | 26 |
| P04792 | Heat shock protein beta-1 OS=Homo sapiens GN=HSPB1 PE=1 SV=2 - [HSPB1_HUMAN] | 12 | 21 | 26 |
| P31943 | Heterogeneous nuclear ribonucleoprotein H OS=Homo sapiens GN=HNRNPH1 PE=1 SV=4 - [HNRH1_HUMAN] | 18 | 19 | 26 |
| P41219 | Peripherin OS=Homo sapiens GN=PRPH PE=1 SV=2 - [PERI_HUMAN] | 77 | 17 | 26 |
| P13667 | Protein disulfide-isomerase A4 OS=Homo sapiens GN=PDIA4 PE=1 SV=2 - [PDIA4_HUMAN] | 12 | 31 | 25 |
| P06748 | Nucleophosmin OS=Homo sapiens GN=NPM1 PE=1 SV=2 - [NPM_HUMAN] | 19 | 26 | 25 |
| Q07065 | Cytoskeleton-associated protein 4 OS=Homo sapiens GN=CKAP4 PE=1 SV=2 - [CKAP4_HUMAN] | 24 | 24 | 25 |
| P05455 | Lupus La protein OS=Homo sapiens GN=SSB PE=1 SV=2 - [LA_HUMAN] | 31 | 22 | 25 |
| P40926 | Malate dehydrogenase, mitochondrial OS=Homo sapiens GN=MDH2 PE=1 SV=3 - [MDHM_HUMAN] | 21 | 21 | 25 |
| O00410 | Importin-5 OS=Homo sapiens GN=IPO5 PE=1 SV=4 - [IPO5_HUMAN] | 25 | 18 | 25 |
| Q06830 | Peroxiredoxin-1 OS=Homo sapiens GN=PRDX1 PE=1 SV=1 - [PRDX1_HUMAN] | 16 | 15 | 25 |
| P12956 | ATP-dependent DNA helicase 2 subunit 1 OS=Homo sapiens GN=XRCC6 PE=1 SV=2 - [KU70_HUMAN] | 23 | 19 | 24 |
| Q04637 | Eukaryotic translation initiation factor 4 gamma 1 OS=Homo sapiens GN=EIF4G1 PE=1 SV=4 - [IF4G1_HUMAN] | 5 | 14 | 24 |
| P62258 | 14-3-3 protein epsilon OS=Homo sapiens GN=YWHAE PE=1 SV=1 - [1433E_HUMAN] | 22 | 22 | 23 |
| O00571 | ATP-dependent RNA helicase DDX3X OS=Homo sapiens GN=DDX3X PE=1 SV=3 - [DDX3X_HUMAN] | 17 | 18 | 23 |
| P49368 | T-complex protein 1 subunit gamma OS=Homo sapiens GN=CCT3 PE=1 SV=4 - [TCPG_HUMAN] | 20 | 16 | 23 |
| P43243 | Matrin-3 OS=Homo sapiens GN=MATR3 PE=1 SV=2 - [MATR3_HUMAN] | 22 | 18 | 22 |
| P62937 | Peptidyl-prolyl cis-trans isomerase A OS=Homo sapiens GN=PPIA PE=1 SV=2 - [PPIA_HUMAN] | 34 | 17 | 22 |
| P50990 | T-complex protein 1 subunit theta OS=Homo sapiens GN=CCT8 PE=1 SV=4 - [TCPQ_HUMAN] | 23 | 17 | 22 |
| P63244 | Guanine nucleotide-binding protein subunit beta-2-like 1 OS=Homo sapiens GN=GNB2L1 PE=1 SV=3 - [GBLP_HUMAN] | 16 | 16 | 22 |
| P26599 | Polypyrimidine tract-binding protein 1 OS=Homo sapiens GN=PTBP1 PE=1 SV=1 - [PTBP1_HUMAN] | 16 | 16 | 22 |
| Q13283 | Ras GTPase-activating protein-binding protein 1 OS=Homo sapiens GN=G3BP1 PE=1 SV=1 - [G3BP1_HUMAN] | 14 | 15 | 22 |
| P09104 | Gamma-enolase OS=Homo sapiens GN=ENO2 PE=1 SV=3 - [ENOG_HUMAN] | 30 | 13 | 22 |
| P02545 | Lamin-A/C OS=Homo sapiens GN=LMNA PE=1 SV=1 - [LMNA_HUMAN] | 20 | 12 | 22 |
| Q9NZI8 | Insulin-like growth factor 2 mRNA-binding protein 1 OS=Homo sapiens GN=IGF2BP1 PE=1 SV=2 - [IF2B1_HUMAN] | 15 | 23 | 21 |
| Q92598 | Heat shock protein 105 kDa OS=Homo sapiens GN=HSPH1 PE=1 SV=1 - [HS105_HUMAN] | 18 | 19 | 21 |
| Q12906 | Interleukin enhancer-binding factor 3 OS=Homo sapiens GN=ILF3 PE=1 SV=3 - [ILF3_HUMAN] | 14 | 19 | 21 |
| P13929 | Beta-enolase OS=Homo sapiens GN=ENO3 PE=1 SV=5 - [ENOB_HUMAN] | 0 | 14 | 21 |
| P55060 | Exportin-2 OS=Homo sapiens GN=CSE1L PE=1 SV=3 - [XPO2_HUMAN] | 30 | 12 | 21 |
| P31948 | Stress-induced-phosphoprotein 1 OS=Homo sapiens GN=STIP1 PE=1 SV=1 - [STIP1_HUMAN] | 23 | 12 | 21 |
| P49411 | Elongation factor Tu, mitochondrial OS=Homo sapiens GN=TUFM PE=1 SV=2 - [EFTU_HUMAN] | 14 | 17 | 20 |
| P50991 | T-complex protein 1 subunit delta OS=Homo sapiens GN=CCT4 PE=1 SV=4 - [TCPD_HUMAN] | 16 | 16 | 20 |
| P27797 | Calreticulin OS=Homo sapiens GN=CALR PE=1 SV=1 - [CALR_HUMAN] | 29 | 14 | 20 |
| O00429 | Dynamin-1-like protein OS=Homo sapiens GN=DNM1L PE=1 SV=2 - [DNM1L_HUMAN] | 16 | 11 | 20 |
| Q16891 | Mitochondrial inner membrane protein OS=Homo sapiens GN=IMMT PE=1 SV=1 - [IMMT_HUMAN] | 8 | 10 | 20 |
| P11940 | Polyadenylate-binding protein 1 OS=Homo sapiens GN=PABPC1 PE=1 SV=2 - [PABP1_HUMAN] | 16 | 17 | 19 |
| P08758 | Annexin A5 OS=Homo sapiens GN=ANXA5 PE=1 SV=2 - [ANXA5_HUMAN] | 15 | 17 | 19 |
| P23528 | Cofilin-1 OS=Homo sapiens GN=CFL1 PE=1 SV=3 - [COF1_HUMAN] | 25 | 16 | 19 |
| Q9Y490 | Talin-1 OS=Homo sapiens GN=TLN1 PE=1 SV=3 - [TLN1_HUMAN] | 12 | 16 | 19 |
| P32119 | Peroxiredoxin-2 OS=Homo sapiens GN=PRDX2 PE=1 SV=5 - [PRDX2_HUMAN] | 22 | 14 | 19 |
| P12268 | Inosine-5'-monophosphate dehydrogenase 2 OS=Homo sapiens GN=IMPDH2 PE=1 SV=2 - [IMDH2_HUMAN] | 14 | 13 | 19 |
| P17844 | Probable ATP-dependent RNA helicase DDX5 OS=Homo sapiens GN=DDX5 PE=1 SV=1 - [DDX5_HUMAN] | 13 | 13 | 19 |
| P33991 | DNA replication licensing factor MCM4 OS=Homo sapiens GN=MCM4 PE=1 SV=5 - [MCM4_HUMAN] | 7 | 6 | 19 |
| P31350 | Ribonucleoside-diphosphate reductase subunit M2 OS=Homo sapiens GN=RRM2 PE=1 SV=1 - [RIR2_HUMAN] | 13 | 17 | 18 |
| P30041 | Peroxiredoxin-6 OS=Homo sapiens GN=PRDX6 PE=1 SV=3 - [PRDX6_HUMAN] | 17 | 16 | 18 |
| Q14152 | Eukaryotic translation initiation factor 3 subunit A OS=Homo sapiens GN=EIF3A PE=1 SV=1 - [EIF3A_HUMAN] | 12 | 16 | 18 |
| P26641 | Elongation factor 1-gamma OS=Homo sapiens GN=EEF1G PE=1 SV=3 - [EF1G_HUMAN] | 24 | 15 | 18 |
| P06744 | Glucose-6-phosphate isomerase OS=Homo sapiens GN=GPI PE=1 SV=4 - [G6PI_HUMAN] | 16 | 14 | 18 |
| O15061 | Synemin OS=Homo sapiens GN=SYNM PE=1 SV=2 - [SYNEM_HUMAN] | 1 | 14 | 18 |
| Q15181 | Inorganic pyrophosphatase OS=Homo sapiens GN=PPA1 PE=1 SV=2 - [IPYR_HUMAN] | 24 | 13 | 18 |
| P09211 | Glutathione S-transferase P OS=Homo sapiens GN=GSTP1 PE=1 SV=2 - [GSTP1_HUMAN] | 18 | 13 | 18 |
| Q00839 | Heterogeneous nuclear ribonucleoprotein U OS=Homo sapiens GN=HNRNPU PE=1 SV=6 - [HNRPU_HUMAN] | 12 | 13 | 18 |
| Q07955 | Splicing factor, arginine/serine-rich 1 OS=Homo sapiens GN=SFRS1 PE=1 SV=2 - [SFRS1_HUMAN] | 13 | 12 | 18 |
| P48643 | T-complex protein 1 subunit epsilon OS=Homo sapiens GN=CCT5 PE=1 SV=1 - [TCPE_HUMAN] | 20 | 10 | 18 |
| Q03252 | Lamin-B2 OS=Homo sapiens GN=LMNB2 PE=1 SV=4 - [LMNB2_HUMAN] | 13 | 9 | 18 |
| P31946 | 14-3-3 protein beta/alpha OS=Homo sapiens GN=YWHAB PE=1 SV=3 - [1433B_HUMAN] | 24 | 19 | 17 |
| P61981 | 14-3-3 protein gamma OS=Homo sapiens GN=YWHAG PE=1 SV=2 - [1433G_HUMAN] | 23 | 18 | 17 |
| Q86VP6 | Cullin-associated NEDD8-dissociated protein 1 OS=Homo sapiens GN=CAND1 PE=1 SV=2 - [CAND1_HUMAN] | 23 | 16 | 17 |
| P49915 | GMP synthase [glutamine-hydrolyzing] OS=Homo sapiens GN=GMPS PE=1 SV=1 - [GUAA_HUMAN] | 3 | 16 | 17 |
| Q92973 | Transportin-1 OS=Homo sapiens GN=TNPO1 PE=1 SV=2 - [TNPO1_HUMAN] | 13 | 15 | 17 |
| P27824 | Calnexin OS=Homo sapiens GN=CANX PE=1 SV=2 - [CALX_HUMAN] | 12 | 15 | 17 |
| Q02790 | Peptidyl-prolyl cis-trans isomerase FKBP4 OS=Homo sapiens GN=FKBP4 PE=1 SV=3 - [FKBP4_HUMAN] | 9 | 14 | 17 |
| Q16643 | Drebrin OS=Homo sapiens GN=DBN1 PE=1 SV=4 - [DREB_HUMAN] | 14 | 13 | 17 |
| P61247 | 40S ribosomal protein S3a OS=Homo sapiens GN=RPS3A PE=1 SV=2 - [RS3A_HUMAN] | 14 | 10 | 17 |
| P50454 | Serpin H1 OS=Homo sapiens GN=SERPINH1 PE=1 SV=2 - [SERPH_HUMAN] | 6 | 10 | 17 |
| P25705 | ATP synthase subunit alpha, mitochondrial OS=Homo sapiens GN=ATP5A1 PE=1 SV=1 - [ATPA_HUMAN] | 26 | 9 | 17 |
| P09874 | Poly [ADP-ribose] polymerase 1 OS=Homo sapiens GN=PARP1 PE=1 SV=4 - [PARP1_HUMAN] | 11 | 9 | 17 |
| P35232 | Prohibitin OS=Homo sapiens GN=PHB PE=1 SV=1 - [PHB_HUMAN] | 25 | 16 | 16 |
| O14980 | Exportin-1 OS=Homo sapiens GN=XPO1 PE=1 SV=1 - [XPO1_HUMAN] | 20 | 16 | 16 |
| Q12905 | Interleukin enhancer-binding factor 2 OS=Homo sapiens GN=ILF2 PE=1 SV=2 - [ILF2_HUMAN] | 18 | 16 | 16 |
| Q92945 | Far upstream element-binding protein 2 OS=Homo sapiens GN=KHSRP PE=1 SV=4 - [FUBP2_HUMAN] | 14 | 16 | 16 |
| P12277 | Creatine kinase B-type OS=Homo sapiens GN=CKB PE=1 SV=1 - [KCRB_HUMAN] | 38 | 15 | 16 |
| P08865 | 40S ribosomal protein SA OS=Homo sapiens GN=RPSA PE=1 SV=4 - [RSSA_HUMAN] | 14 | 15 | 16 |
| P54577 | Tyrosyl-tRNA synthetase, cytoplasmic OS=Homo sapiens GN=YARS PE=1 SV=4 - [SYYC_HUMAN] | 12 | 15 | 16 |
| Q9UHD8 | Septin-9 OS=Homo sapiens GN=SEPT9 PE=1 SV=2 - [SEPT9_HUMAN] | 9 | 15 | 16 |
| P12270 | Nucleoprotein TPR OS=Homo sapiens GN=TPR PE=1 SV=3 - [TPR_HUMAN] | 3 | 15 | 16 |
| Q86UP2 | Kinectin OS=Homo sapiens GN=KTN1 PE=1 SV=1 - [KTN1_HUMAN] | 20 | 12 | 16 |
| Q9Y265 | RuvB-like 1 OS=Homo sapiens GN=RUVBL1 PE=1 SV=1 - [RUVB1_HUMAN] | 11 | 12 | 16 |
| Q9Y230 | RuvB-like 2 OS=Homo sapiens GN=RUVBL2 PE=1 SV=3 - [RUVB2_HUMAN] | 8 | 12 | 16 |
| Q16658 | Fascin OS=Homo sapiens GN=FSCN1 PE=1 SV=3 - [FSCN1_HUMAN] | 21 | 11 | 16 |
| P00338 | L-lactate dehydrogenase A chain OS=Homo sapiens GN=LDHA PE=1 SV=2 - [LDHA_HUMAN] | 21 | 11 | 16 |
| Q13162 | Peroxiredoxin-4 OS=Homo sapiens GN=PRDX4 PE=1 SV=1 - [PRDX4_HUMAN] | 14 | 11 | 16 |
| O95373 | Importin-7 OS=Homo sapiens GN=IPO7 PE=1 SV=1 - [IPO7_HUMAN] | 12 | 11 | 16 |
| P67809 | Nuclease-sensitive element-binding protein 1 OS=Homo sapiens GN=YBX1 PE=1 SV=3 - [YBOX1_HUMAN] | 11 | 11 | 16 |
| P39687 | Acidic leucine-rich nuclear phosphoprotein 32 family member A OS=Homo sapiens GN=ANP32A PE=1 SV=1 - [AN32A_HUMAN] | 18 | 9 | 16 |
| P62701 | 40S ribosomal protein S4, X isoform OS=Homo sapiens GN=RPS4X PE=1 SV=2 - [RS4X_HUMAN] | 11 | 9 | 16 |
| Q15365 | Poly(rC)-binding protein 1 OS=Homo sapiens GN=PCBP1 PE=1 SV=2 - [PCBP1_HUMAN] | 13 | 8 | 16 |
| Q99832 | T-complex protein 1 subunit eta OS=Homo sapiens GN=CCT7 PE=1 SV=2 - [TCPH_HUMAN] | 24 | 16 | 15 |
| O43390 | Heterogeneous nuclear ribonucleoprotein R OS=Homo sapiens GN=HNRNPR PE=1 SV=1 - [HNRPR_HUMAN] | 13 | 16 | 15 |
| P78347 | General transcription factor II-I OS=Homo sapiens GN=GTF2I PE=1 SV=2 - [GTF2I_HUMAN] | 11 | 15 | 15 |
| O60506 | Heterogeneous nuclear ribonucleoprotein Q OS=Homo sapiens GN=SYNCRIP PE=1 SV=2 - [HNRPQ_HUMAN] | 15 | 14 | 15 |
| P62805 | Histone H4 OS=Homo sapiens GN=HIST1H4A PE=1 SV=2 - [H4_HUMAN] | 13 | 14 | 15 |
| Q8N163 | Protein KIAA1967 OS=Homo sapiens GN=KIAA1967 PE=1 SV=2 - [K1967_HUMAN] | 7 | 13 | 15 |
| P11586 | C-1-tetrahydrofolate synthase, cytoplasmic OS=Homo sapiens GN=MTHFD1 PE=1 SV=3 - [C1TC_HUMAN] | 5 | 13 | 15 |
| P42166 | Lamina-associated polypeptide 2, isoform alpha OS=Homo sapiens GN=TMPO PE=1 SV=2 - [LAP2A_HUMAN] | 16 | 12 | 15 |
| P54136 | Arginyl-tRNA synthetase, cytoplasmic OS=Homo sapiens GN=RARS PE=1 SV=2 - [SYRC_HUMAN] | 13 | 12 | 15 |
| P52272 | Heterogeneous nuclear ribonucleoprotein M OS=Homo sapiens GN=HNRNPM PE=1 SV=3 - [HNRPM_HUMAN] | 12 | 12 | 15 |
| P34897 | Serine hydroxymethyltransferase, mitochondrial OS=Homo sapiens GN=SHMT2 PE=1 SV=3 - [GLYM_HUMAN] | 12 | 12 | 15 |
| P46782 | 40S ribosomal protein S5 OS=Homo sapiens GN=RPS5 PE=1 SV=4 - [RS5_HUMAN] | 12 | 11 | 15 |
| O00231 | 26S proteasome non-ATPase regulatory subunit 11 OS=Homo sapiens GN=PSMD11 PE=1 SV=3 - [PSD11_HUMAN] | 7 | 11 | 15 |
| Q96CX2 | BTB/POZ domain-containing protein KCTD12 OS=Homo sapiens GN=KCTD12 PE=1 SV=1 - [KCD12_HUMAN] | 6 | 11 | 15 |
| P29401 | Transketolase OS=Homo sapiens GN=TKT PE=1 SV=3 - [TKT_HUMAN] | 16 | 10 | 15 |
| P05388 | 60S acidic ribosomal protein P0 OS=Homo sapiens GN=RPLP0 PE=1 SV=1 - [RLA0_HUMAN] | 14 | 10 | 15 |
| P37235 | Hippocalcin-like protein 1 OS=Homo sapiens GN=HPCAL1 PE=1 SV=3 - [HPCL1_HUMAN] | 13 | 10 | 15 |
| P61289 | Proteasome activator complex subunit 3 OS=Homo sapiens GN=PSME3 PE=1 SV=1 - [PSME3_HUMAN] | 13 | 10 | 15 |
| P25205 | DNA replication licensing factor MCM3 OS=Homo sapiens GN=MCM3 PE=1 SV=3 - [MCM3_HUMAN] | 12 | 10 | 15 |
| Q9UQ80 | Proliferation-associated protein 2G4 OS=Homo sapiens GN=PA2G4 PE=1 SV=3 - [PA2G4_HUMAN] | 11 | 10 | 15 |
| P30086 | Phosphatidylethanolamine-binding protein 1 OS=Homo sapiens GN=PEBP1 PE=1 SV=3 - [PEBP1_HUMAN] | 16 | 9 | 15 |
| P29590 | Probable transcription factor PML OS=Homo sapiens GN=PML PE=1 SV=3 - [PML_HUMAN] | 0 | 8 | 15 |
| Q14157 | Ubiquitin-associated protein 2-like OS=Homo sapiens GN=UBAP2L PE=1 SV=2 - [UBP2L_HUMAN] | 14 | 7 | 15 |
| P33316 | Deoxyuridine 5'-triphosphate nucleotidohydrolase, mitochondrial OS=Homo sapiens GN=DUT PE=1 SV=4 - [DUT_HUMAN] | 8 | 7 | 15 |
| Q99497 | Protein DJ-1 OS=Homo sapiens GN=PARK7 PE=1 SV=2 - [PARK7_HUMAN] | 16 | 6 | 15 |
| P68104 | Elongation factor 1-alpha 1 OS=Homo sapiens GN=EEF1A1 PE=1 SV=1 - [EF1A1_HUMAN] | 18 | 18 | 14 |
| P36873 | Serine/threonine-protein phosphatase PP1-gamma catalytic subunit OS=Homo sapiens GN=PPP1CC PE=1 SV=1 - [PP1G_HUMAN] | 13 | 16 | 14 |
| P48735 | Isocitrate dehydrogenase [NADP], mitochondrial OS=Homo sapiens GN=IDH2 PE=1 SV=2 - [IDHP_HUMAN] | 18 | 14 | 14 |
| P40227 | T-complex protein 1 subunit zeta OS=Homo sapiens GN=CCT6A PE=1 SV=3 - [TCPZ_HUMAN] | 26 | 13 | 14 |
| Q96AE4 | Far upstream element-binding protein 1 OS=Homo sapiens GN=FUBP1 PE=1 SV=3 - [FUBP1_HUMAN] | 11 | 13 | 14 |
| P15531 | Nucleoside diphosphate kinase A OS=Homo sapiens GN=NME1 PE=1 SV=1 - [NDKA_HUMAN] | 24 | 12 | 14 |
| P49591 | Seryl-tRNA synthetase, cytoplasmic OS=Homo sapiens GN=SARS PE=1 SV=3 - [SYSC_HUMAN] | 17 | 12 | 14 |
| P62979 | Ubiquitin-40S ribosomal protein S27a OS=Homo sapiens GN=RPS27A PE=1 SV=2 - [RS27A_HUMAN] | 18 | 10 | 14 |
| P23246 | Splicing factor, proline- and glutamine-rich OS=Homo sapiens GN=SFPQ PE=1 SV=2 - [SFPQ_HUMAN] | 9 | 10 | 14 |
| P05091 | Aldehyde dehydrogenase, mitochondrial OS=Homo sapiens GN=ALDH2 PE=1 SV=2 - [ALDH2_HUMAN] | 7 | 10 | 14 |
| P22392 | Nucleoside diphosphate kinase B OS=Homo sapiens GN=NME2 PE=1 SV=1 - [NDKB_HUMAN] | 26 | 9 | 14 |
| P26583 | High mobility group protein B2 OS=Homo sapiens GN=HMGB2 PE=1 SV=2 - [HMGB2_HUMAN] | 15 | 9 | 14 |
| P46783 | 40S ribosomal protein S10 OS=Homo sapiens GN=RPS10 PE=1 SV=1 - [RS10_HUMAN] | 11 | 8 | 14 |
| P37802 | Transgelin-2 OS=Homo sapiens GN=TAGLN2 PE=1 SV=3 - [TAGL2_HUMAN] | 16 | 7 | 14 |
| Q99623 | Prohibitin-2 OS=Homo sapiens GN=PHB2 PE=1 SV=2 - [PHB2_HUMAN] | 14 | 7 | 14 |
| Q9NQG5 | Regulation of nuclear pre-mRNA domain-containing protein 1B OS=Homo sapiens GN=RPRD1B PE=1 SV=1 - [RPR1B_HUMAN] | 6 | 7 | 14 |
| Q9Y696 | Chloride intracellular channel protein 4 OS=Homo sapiens GN=CLIC4 PE=1 SV=4 - [CLIC4_HUMAN] | 5 | 7 | 14 |
| O75116 | Rho-associated protein kinase 2 OS=Homo sapiens GN=ROCK2 PE=1 SV=4 - [ROCK2_HUMAN] | 6 | 16 | 13 |
| Q8WUM4 | Programmed cell death 6-interacting protein OS=Homo sapiens GN=PDCD6IP PE=1 SV=1 - [PDC6I_HUMAN] | 7 | 14 | 13 |
| Q02952 | A-kinase anchor protein 12 OS=Homo sapiens GN=AKAP12 PE=1 SV=4 - [AKA12_HUMAN] | 3 | 13 | 13 |
| P51991 | Heterogeneous nuclear ribonucleoprotein A3 OS=Homo sapiens GN=HNRNPA3 PE=1 SV=2 - [ROA3_HUMAN] | 20 | 12 | 13 |
| P55209 | Nucleosome assembly protein 1-like 1 OS=Homo sapiens GN=NAP1L1 PE=1 SV=1 - [NP1L1_HUMAN] | 14 | 12 | 13 |
| P21796 | Voltage-dependent anion-selective channel protein 1 OS=Homo sapiens GN=VDAC1 PE=1 SV=2 - [VDAC1_HUMAN] | 13 | 12 | 13 |
| P30154 | Serine/threonine-protein phosphatase 2A 65 kDa regulatory subunit A beta isoform OS=Homo sapiens GN=PPP2R1B PE=1 SV=3 - [2AAB_HUMAN] | 12 | 11 | 13 |
| Q96AG4 | Leucine-rich repeat-containing protein 59 OS=Homo sapiens GN=LRRC59 PE=1 SV=1 - [LRC59_HUMAN] | 15 | 10 | 13 |
| P06753 | Tropomyosin alpha-3 chain OS=Homo sapiens GN=TPM3 PE=1 SV=2 - [TPM3_HUMAN] | 6 | 10 | 13 |
| P09493 | Tropomyosin alpha-1 chain OS=Homo sapiens GN=TPM1 PE=1 SV=2 - [TPM1_HUMAN] | 4 | 10 | 13 |
| P11047 | Laminin subunit gamma-1 OS=Homo sapiens GN=LAMC1 PE=1 SV=3 - [LAMC1_HUMAN] | 0 | 10 | 13 |
| Q92688 | Acidic leucine-rich nuclear phosphoprotein 32 family member B OS=Homo sapiens GN=ANP32B PE=1 SV=1 - [AN32B_HUMAN] | 17 | 9 | 13 |
| Q99459 | Cell division cycle 5-like protein OS=Homo sapiens GN=CDC5L PE=1 SV=2 - [CDC5L_HUMAN] | 6 | 9 | 13 |
| P18669 | Phosphoglycerate mutase 1 OS=Homo sapiens GN=PGAM1 PE=1 SV=2 - [PGAM1_HUMAN] | 16 | 8 | 13 |
| P46060 | Ran GTPase-activating protein 1 OS=Homo sapiens GN=RANGAP1 PE=1 SV=1 - [RAGP1_HUMAN] | 11 | 7 | 13 |
| Q96QK1 | Vacuolar protein sorting-associated protein 35 OS=Homo sapiens GN=VPS35 PE=1 SV=2 - [VPS35_HUMAN] | 19 | 5 | 13 |
| P28331 | NADH-ubiquinone oxidoreductase 75 kDa subunit, mitochondrial OS=Homo sapiens GN=NDUFS1 PE=1 SV=3 - [NDUS1_HUMAN] | 7 | 4 | 13 |
| Q04917 | 14-3-3 protein eta OS=Homo sapiens GN=YWHAH PE=1 SV=4 - [1433F_HUMAN] | 12 | 15 | 12 |
| P62136 | Serine/threonine-protein phosphatase PP1-alpha catalytic subunit OS=Homo sapiens GN=PPP1CA PE=1 SV=1 - [PP1A_HUMAN] | 0 | 15 | 12 |
| P62140 | Serine/threonine-protein phosphatase PP1-beta catalytic subunit OS=Homo sapiens GN=PPP1CB PE=1 SV=3 - [PP1B_HUMAN] | 12 | 14 | 12 |
| Q99798 | Aconitate hydratase, mitochondrial OS=Homo sapiens GN=ACO2 PE=1 SV=2 - [ACON_HUMAN] | 18 | 12 | 12 |
| P33176 | Kinesin-1 heavy chain OS=Homo sapiens GN=KIF5B PE=1 SV=1 - [KINH_HUMAN] | 13 | 12 | 12 |
| Q9NQC3 | Reticulon-4 OS=Homo sapiens GN=RTN4 PE=1 SV=2 - [RTN4_HUMAN] | 10 | 12 | 12 |
| P53396 | ATP-citrate synthase OS=Homo sapiens GN=ACLY PE=1 SV=3 - [ACLY_HUMAN] | 21 | 11 | 12 |
| P34932 | Heat shock 70 kDa protein 4 OS=Homo sapiens GN=HSPA4 PE=1 SV=4 - [HSP74_HUMAN] | 12 | 11 | 12 |
| Q92841 | Probable ATP-dependent RNA helicase DDX17 OS=Homo sapiens GN=DDX17 PE=1 SV=2 - [DDX17_HUMAN] | 10 | 11 | 12 |
| P35579 | Myosin-9 OS=Homo sapiens GN=MYH9 PE=1 SV=4 - [MYH9_HUMAN] | 5 | 11 | 12 |
| P50395 | Rab GDP dissociation inhibitor beta OS=Homo sapiens GN=GDI2 PE=1 SV=2 - [GDIB_HUMAN] | 17 | 10 | 12 |
| P22234 | Multifunctional protein ADE2 OS=Homo sapiens GN=PAICS PE=1 SV=3 - [PUR6_HUMAN] | 14 | 10 | 12 |
| Q15785 | Mitochondrial import receptor subunit TOM34 OS=Homo sapiens GN=TOMM34 PE=1 SV=2 - [TOM34_HUMAN] | 10 | 10 | 12 |
| Q13200 | 26S proteasome non-ATPase regulatory subunit 2 OS=Homo sapiens GN=PSMD2 PE=1 SV=3 - [PSMD2_HUMAN] | 9 | 10 | 12 |
| P49588 | Alanyl-tRNA synthetase, cytoplasmic OS=Homo sapiens GN=AARS PE=1 SV=2 - [SYAC_HUMAN] | 15 | 9 | 12 |
| P67936 | Tropomyosin alpha-4 chain OS=Homo sapiens GN=TPM4 PE=1 SV=3 - [TPM4_HUMAN] | 7 | 9 | 12 |
| P49736 | DNA replication licensing factor MCM2 OS=Homo sapiens GN=MCM2 PE=1 SV=4 - [MCM2_HUMAN] | 9 | 8 | 12 |
| P07737 | Profilin-1 OS=Homo sapiens GN=PFN1 PE=1 SV=2 - [PROF1_HUMAN] | 13 | 7 | 12 |
| Q15417 | Calponin-3 OS=Homo sapiens GN=CNN3 PE=1 SV=1 - [CNN3_HUMAN] | 9 | 7 | 12 |
| P33993 | DNA replication licensing factor MCM7 OS=Homo sapiens GN=MCM7 PE=1 SV=4 - [MCM7_HUMAN] | 8 | 7 | 12 |
| P29692 | Elongation factor 1-delta OS=Homo sapiens GN=EEF1D PE=1 SV=5 - [EF1D_HUMAN] | 7 | 6 | 12 |
| P35637 | RNA-binding protein FUS OS=Homo sapiens GN=FUS PE=1 SV=1 - [FUS_HUMAN] | 13 | 5 | 12 |
| P13489 | Ribonuclease inhibitor OS=Homo sapiens GN=RNH1 PE=1 SV=2 - [RINI_HUMAN] | 3 | 5 | 12 |
| P02768 | Serum albumin OS=Homo sapiens GN=ALB PE=1 SV=2 - [ALBU_HUMAN] | 9 | 15 | 11 |
| P18206 | Vinculin OS=Homo sapiens GN=VCL PE=1 SV=4 - [VINC_HUMAN] | 3 | 15 | 11 |
| Q15019 | Septin-2 OS=Homo sapiens GN=SEPT2 PE=1 SV=1 - [SEPT2_HUMAN] | 11 | 13 | 11 |
| Q96AY3 | Peptidyl-prolyl cis-trans isomerase FKBP10 OS=Homo sapiens GN=FKBP10 PE=1 SV=1 - [FKB10_HUMAN] | 2 | 13 | 11 |
| P09936 | Ubiquitin carboxyl-terminal hydrolase isozyme L1 OS=Homo sapiens GN=UCHL1 PE=1 SV=2 - [UCHL1_HUMAN] | 21 | 10 | 11 |
| P23284 | Peptidyl-prolyl cis-trans isomerase B OS=Homo sapiens GN=PPIB PE=1 SV=2 - [PPIB_HUMAN] | 11 | 10 | 11 |
| O00425 | Insulin-like growth factor 2 mRNA-binding protein 3 OS=Homo sapiens GN=IGF2BP3 PE=1 SV=2 - [IF2B3_HUMAN] | 10 | 10 | 11 |
| Q9NSD9 | Phenylalanine--tRNA ligase beta subunit OS=Homo sapiens GN=FARSB PE=1 SV=3 - [SYFB_HUMAN] | 10 | 10 | 11 |
| P52597 | Heterogeneous nuclear ribonucleoprotein F OS=Homo sapiens GN=HNRNPF PE=1 SV=3 - [HNRPF_HUMAN] | 9 | 10 | 11 |
| P42167 | Lamina-associated polypeptide 2, isoforms beta/gamma OS=Homo sapiens GN=TMPO PE=1 SV=2 - [LAP2B_HUMAN] | 15 | 9 | 11 |
| Q99714 | 3-hydroxyacyl-CoA dehydrogenase type-2 OS=Homo sapiens GN=HSD17B10 PE=1 SV=3 - [HCD2_HUMAN] | 14 | 9 | 11 |
| P30040 | Endoplasmic reticulum resident protein 29 OS=Homo sapiens GN=ERP29 PE=1 SV=4 - [ERP29_HUMAN] | 12 | 9 | 11 |
| Q9HB71 | Calcyclin-binding protein OS=Homo sapiens GN=CACYBP PE=1 SV=2 - [CYBP_HUMAN] | 11 | 9 | 11 |
| P62826 | GTP-binding nuclear protein Ran OS=Homo sapiens GN=RAN PE=1 SV=3 - [RAN_HUMAN] | 11 | 9 | 11 |
| P38159 | Heterogeneous nuclear ribonucleoprotein G OS=Homo sapiens GN=RBMX PE=1 SV=3 - [HNRPG_HUMAN] | 10 | 9 | 11 |
| P07910 | Heterogeneous nuclear ribonucleoproteins C1/C2 OS=Homo sapiens GN=HNRNPC PE=1 SV=4 - [HNRPC_HUMAN] | 9 | 9 | 11 |
| P45880 | Voltage-dependent anion-selective channel protein 2 OS=Homo sapiens GN=VDAC2 PE=1 SV=2 - [VDAC2_HUMAN] | 9 | 9 | 11 |
| P51149 | Ras-related protein Rab-7a OS=Homo sapiens GN=RAB7A PE=1 SV=1 - [RAB7A_HUMAN] | 6 | 9 | 11 |
| P12081 | Histidyl-tRNA synthetase, cytoplasmic OS=Homo sapiens GN=HARS PE=1 SV=2 - [SYHC_HUMAN] | 14 | 8 | 11 |
| P30050 | 60S ribosomal protein L12 OS=Homo sapiens GN=RPL12 PE=1 SV=1 - [RL12_HUMAN] | 12 | 8 | 11 |
| Q12931 | Heat shock protein 75 kDa, mitochondrial OS=Homo sapiens GN=TRAP1 PE=1 SV=3 - [TRAP1_HUMAN] | 12 | 8 | 11 |
| P78417 | Glutathione S-transferase omega-1 OS=Homo sapiens GN=GSTO1 PE=1 SV=2 - [GSTO1_HUMAN] | 11 | 8 | 11 |
| Q9NVA2 | Septin-11 OS=Homo sapiens GN=SEPT11 PE=1 SV=3 - [SEP11_HUMAN] | 7 | 8 | 11 |
| P45974 | Ubiquitin carboxyl-terminal hydrolase 5 OS=Homo sapiens GN=USP5 PE=1 SV=2 - [UBP5_HUMAN] | 7 | 8 | 11 |
| P38606 | V-type proton ATPase catalytic subunit A OS=Homo sapiens GN=ATP6V1A PE=1 SV=2 - [VATA_HUMAN] | 7 | 8 | 11 |
| P36578 | 60S ribosomal protein L4 OS=Homo sapiens GN=RPL4 PE=1 SV=5 - [RL4_HUMAN] | 14 | 7 | 11 |
| P09429 | High mobility group protein B1 OS=Homo sapiens GN=HMGB1 PE=1 SV=3 - [HMGB1_HUMAN] | 14 | 7 | 11 |
| Q9NTK5 | Obg-like ATPase 1 OS=Homo sapiens GN=OLA1 PE=1 SV=2 - [OLA1_HUMAN] | 14 | 7 | 11 |
| P62081 | 40S ribosomal protein S7 OS=Homo sapiens GN=RPS7 PE=1 SV=1 - [RS7_HUMAN] | 12 | 7 | 11 |
| P63010 | AP-2 complex subunit beta OS=Homo sapiens GN=AP2B1 PE=1 SV=1 - [AP2B1_HUMAN] | 11 | 7 | 11 |
| P13804 | Electron transfer flavoprotein subunit alpha, mitochondrial OS=Homo sapiens GN=ETFA PE=1 SV=1 - [ETFA_HUMAN] | 10 | 7 | 11 |
| P00441 | Superoxide dismutase [Cu-Zn] OS=Homo sapiens GN=SOD1 PE=1 SV=2 - [SODC_HUMAN] | 9 | 7 | 11 |
| P49419 | Alpha-aminoadipic semialdehyde dehydrogenase OS=Homo sapiens GN=ALDH7A1 PE=1 SV=5 - [AL7A1_HUMAN] | 8 | 7 | 11 |
| P26378 | ELAV-like protein 4 OS=Homo sapiens GN=ELAVL4 PE=1 SV=2 - [ELAV4_HUMAN] | 7 | 7 | 11 |
| Q15691 | Microtubule-associated protein RP/EB family member 1 OS=Homo sapiens GN=MAPRE1 PE=1 SV=3 - [MARE1_HUMAN] | 13 | 6 | 11 |
| P36776 | Lon protease homolog, mitochondrial OS=Homo sapiens GN=LONP1 PE=1 SV=2 - [LONM_HUMAN] | 11 | 6 | 11 |
| P17174 | Aspartate aminotransferase, cytoplasmic OS=Homo sapiens GN=GOT1 PE=1 SV=3 - [AATC_HUMAN] | 10 | 6 | 11 |
| Q15366 | Poly(rC)-binding protein 2 OS=Homo sapiens GN=PCBP2 PE=1 SV=1 - [PCBP2_HUMAN] | 10 | 6 | 11 |
| Q13177 | Serine/threonine-protein kinase PAK 2 OS=Homo sapiens GN=PAK2 PE=1 SV=3 - [PAK2_HUMAN] | 6 | 6 | 11 |
| P35998 | 26S protease regulatory subunit 7 OS=Homo sapiens GN=PSMC2 PE=1 SV=3 - [PRS7_HUMAN] | 4 | 6 | 11 |
| P35249 | Replication factor C subunit 4 OS=Homo sapiens GN=RFC4 PE=1 SV=2 - [RFC4_HUMAN] | 12 | 5 | 11 |
| Q14697 | Neutral alpha-glucosidase AB OS=Homo sapiens GN=GANAB PE=1 SV=3 - [GANAB_HUMAN] | 10 | 5 | 11 |
| Q6PKG0 | La-related protein 1 OS=Homo sapiens GN=LARP1 PE=1 SV=2 - [LARP1_HUMAN] | 8 | 5 | 11 |
| P78344 | Eukaryotic translation initiation factor 4 gamma 2 OS=Homo sapiens GN=EIF4G2 PE=1 SV=1 - [IF4G2_HUMAN] | 7 | 5 | 11 |
| Q93009 | Ubiquitin carboxyl-terminal hydrolase 7 OS=Homo sapiens GN=USP7 PE=1 SV=2 - [UBP7_HUMAN] | 3 | 4 | 11 |
| Q16352 | Alpha-internexin OS=Homo sapiens GN=INA PE=1 SV=2 - [AINX_HUMAN] | 34 | 3 | 11 |
| Q9Y224 | UPF0568 protein C14orf166 OS=Homo sapiens GN=C14orf166 PE=1 SV=1 - [CN166_HUMAN] | 12 | 3 | 11 |
| O76003 | Glutaredoxin-3 OS=Homo sapiens GN=GLRX3 PE=1 SV=2 - [GLRX3_HUMAN] | 8 | 3 | 11 |
| P52788 | Spermine synthase OS=Homo sapiens GN=SMS PE=1 SV=2 - [SPSY_HUMAN] | 8 | 3 | 11 |
| Q9UIG0 | Tyrosine-protein kinase BAZ1B OS=Homo sapiens GN=BAZ1B PE=1 SV=2 - [BAZ1B_HUMAN] | 1 | 3 | 11 |
| Q13310 | Polyadenylate-binding protein 4 OS=Homo sapiens GN=PABPC4 PE=1 SV=1 - [PABP4_HUMAN] | 9 | 13 | 10 |
| P40925 | Malate dehydrogenase, cytoplasmic OS=Homo sapiens GN=MDH1 PE=1 SV=4 - [MDHC_HUMAN] | 12 | 12 | 10 |
| Q00341 | Vigilin OS=Homo sapiens GN=HDLBP PE=1 SV=2 - [VIGLN_HUMAN] | 2 | 11 | 10 |
| Q9UJZ1 | Stomatin-like protein 2 OS=Homo sapiens GN=STOML2 PE=1 SV=1 - [STML2_HUMAN] | 16 | 10 | 10 |
| P27695 | DNA-(apurinic or apyrimidinic site) lyase OS=Homo sapiens GN=APEX1 PE=1 SV=2 - [APEX1_HUMAN] | 14 | 10 | 10 |
| P43686 | 26S protease regulatory subunit 6B OS=Homo sapiens GN=PSMC4 PE=1 SV=2 - [PRS6B_HUMAN] | 12 | 10 | 10 |
| O60814 | Histone H2B type 1-K OS=Homo sapiens GN=HIST1H2BK PE=1 SV=3 - [H2B1K_HUMAN] | 9 | 10 | 10 |
| Q99615 | DnaJ homolog subfamily C member 7 OS=Homo sapiens GN=DNAJC7 PE=1 SV=2 - [DNJC7_HUMAN] | 7 | 10 | 10 |
| O60841 | Eukaryotic translation initiation factor 5B OS=Homo sapiens GN=EIF5B PE=1 SV=4 - [IF2P_HUMAN] | 3 | 10 | 10 |
| O60701 | UDP-glucose 6-dehydrogenase OS=Homo sapiens GN=UGDH PE=1 SV=1 - [UGDH_HUMAN] | 3 | 10 | 10 |
| Q08257 | Quinone oxidoreductase OS=Homo sapiens GN=CRYZ PE=1 SV=1 - [QOR_HUMAN] | 0 | 10 | 10 |
| O75822 | Eukaryotic translation initiation factor 3 subunit J OS=Homo sapiens GN=EIF3J PE=1 SV=2 - [EIF3J_HUMAN] | 8 | 9 | 10 |
| Q8IV08 | Phospholipase D3 OS=Homo sapiens GN=PLD3 PE=1 SV=1 - [PLD3_HUMAN] | 2 | 9 | 10 |
| Q12765 | Secernin-1 OS=Homo sapiens GN=SCRN1 PE=1 SV=2 - [SCRN1_HUMAN] | 14 | 8 | 10 |
| Q14103 | Heterogeneous nuclear ribonucleoprotein D0 OS=Homo sapiens GN=HNRNPD PE=1 SV=1 - [HNRPD_HUMAN] | 9 | 8 | 10 |
| P47756 | F-actin-capping protein subunit beta OS=Homo sapiens GN=CAPZB PE=1 SV=4 - [CAPZB_HUMAN] | 7 | 8 | 10 |
| O00299 | Chloride intracellular channel protein 1 OS=Homo sapiens GN=CLIC1 PE=1 SV=4 - [CLIC1_HUMAN] | 6 | 8 | 10 |
| P43490 | Nicotinamide phosphoribosyltransferase OS=Homo sapiens GN=NAMPT PE=1 SV=1 - [NAMPT_HUMAN] | 4 | 8 | 10 |
| O43396 | Thioredoxin-like protein 1 OS=Homo sapiens GN=TXNL1 PE=1 SV=3 - [TXNL1_HUMAN] | 4 | 8 | 10 |
| P63241 | Eukaryotic translation initiation factor 5A-1 OS=Homo sapiens GN=EIF5A PE=1 SV=2 - [IF5A1_HUMAN] | 24 | 7 | 10 |
| Q02878 | 60S ribosomal protein L6 OS=Homo sapiens GN=RPL6 PE=1 SV=3 - [RL6_HUMAN] | 11 | 7 | 10 |
| Q15907 | Ras-related protein Rab-11B OS=Homo sapiens GN=RAB11B PE=1 SV=4 - [RB11B_HUMAN] | 8 | 7 | 10 |
| Q9UBT2 | SUMO-activating enzyme subunit 2 OS=Homo sapiens GN=UBA2 PE=1 SV=2 - [SAE2_HUMAN] | 3 | 7 | 10 |
| P61604 | 10 kDa heat shock protein, mitochondrial OS=Homo sapiens GN=HSPE1 PE=1 SV=2 - [CH10_HUMAN] | 23 | 6 | 10 |
| P25398 | 40S ribosomal protein S12 OS=Homo sapiens GN=RPS12 PE=1 SV=3 - [RS12_HUMAN] | 17 | 6 | 10 |
| Q7KZF4 | Staphylococcal nuclease domain-containing protein 1 OS=Homo sapiens GN=SND1 PE=1 SV=1 - [SND1_HUMAN] | 13 | 6 | 10 |
| O75489 | NADH dehydrogenase [ubiquinone] iron-sulfur protein 3, mitochondrial OS=Homo sapiens GN=NDUFS3 PE=1 SV=1 - [NDUS3_HUMAN] | 9 | 6 | 10 |
| O94826 | Mitochondrial import receptor subunit TOM70 OS=Homo sapiens GN=TOMM70A PE=1 SV=1 - [TOM70_HUMAN] | 8 | 6 | 10 |
| Q9Y285 | Phenylalanyl-tRNA synthetase alpha chain OS=Homo sapiens GN=FARSA PE=1 SV=3 - [SYFA_HUMAN] | 8 | 6 | 10 |
| Q8N1G4 | Leucine-rich repeat-containing protein 47 OS=Homo sapiens GN=LRRC47 PE=1 SV=1 - [LRC47_HUMAN] | 7 | 6 | 10 |
| P09972 | Fructose-bisphosphate aldolase C OS=Homo sapiens GN=ALDOC PE=1 SV=2 - [ALDOC_HUMAN] | 6 | 6 | 10 |
| O75369 | Filamin-B OS=Homo sapiens GN=FLNB PE=1 SV=2 - [FLNB_HUMAN] | 0 | 6 | 10 |
| P27816 | Microtubule-associated protein 4 OS=Homo sapiens GN=MAP4 PE=1 SV=3 - [MAP4_HUMAN] | 0 | 6 | 10 |
| O14979 | Heterogeneous nuclear ribonucleoprotein D-like OS=Homo sapiens GN=HNRPDL PE=1 SV=3 - [HNRDL_HUMAN] | 7 | 5 | 10 |
| P50502 | Hsc70-interacting protein OS=Homo sapiens GN=ST13 PE=1 SV=2 - [F10A1_HUMAN] | 7 | 5 | 10 |
| Q7Z460 | CLIP-associating protein 1 OS=Homo sapiens GN=CLASP1 PE=1 SV=1 - [CLAP1_HUMAN] | 2 | 5 | 10 |
| O14776 | Transcription elongation regulator 1 OS=Homo sapiens GN=TCERG1 PE=1 SV=2 - [TCRG1_HUMAN] | 2 | 5 | 10 |
| P25787 | Proteasome subunit alpha type-2 OS=Homo sapiens GN=PSMA2 PE=1 SV=2 - [PSA2_HUMAN] | 10 | 4 | 10 |
| P09661 | U2 small nuclear ribonucleoprotein A' OS=Homo sapiens GN=SNRPA1 PE=1 SV=2 - [RU2A_HUMAN] | 10 | 4 | 10 |
| Q9UNM6 | 26S proteasome non-ATPase regulatory subunit 13 OS=Homo sapiens GN=PSMD13 PE=1 SV=2 - [PSD13_HUMAN] | 8 | 4 | 10 |
| P21266 | Glutathione S-transferase Mu 3 OS=Homo sapiens GN=GSTM3 PE=1 SV=3 - [GSTM3_HUMAN] | 8 | 4 | 10 |
| P54819 | Adenylate kinase 2, mitochondrial OS=Homo sapiens GN=AK2 PE=1 SV=2 - [KAD2_HUMAN] | 8 | 3 | 10 |
| P51665 | 26S proteasome non-ATPase regulatory subunit 7 OS=Homo sapiens GN=PSMD7 PE=1 SV=2 - [PSD7_HUMAN] | 10 | 2 | 10 |
| Q05639 | Elongation factor 1-alpha 2 OS=Homo sapiens GN=EEF1A2 PE=1 SV=1 - [EF1A2_HUMAN] | 12 | 12 | 9 |
| P13797 | Plastin-3 OS=Homo sapiens GN=PLS3 PE=1 SV=4 - [PLST_HUMAN] | 7 | 11 | 9 |
| P16152 | Carbonyl reductase [NADPH] 1 OS=Homo sapiens GN=CBR1 PE=1 SV=3 - [CBR1_HUMAN] | 5 | 11 | 9 |
| Q00610 | Clathrin heavy chain 1 OS=Homo sapiens GN=CLTC PE=1 SV=5 - [CLH1_HUMAN] | 12 | 10 | 9 |
| P16401 | Histone H1.5 OS=Homo sapiens GN=HIST1H1B PE=1 SV=3 - [H15_HUMAN] | 5 | 10 | 9 |
| Q96I24 | Far upstream element-binding protein 3 OS=Homo sapiens GN=FUBP3 PE=1 SV=2 - [FUBP3_HUMAN] | 8 | 9 | 9 |
| P06899 | Histone H2B type 1-J OS=Homo sapiens GN=HIST1H2BJ PE=1 SV=3 - [H2B1J_HUMAN] | 8 | 9 | 9 |
| P54727 | UV excision repair protein RAD23 homolog B OS=Homo sapiens GN=RAD23B PE=1 SV=1 - [RD23B_HUMAN] | 8 | 9 | 9 |
| P52907 | F-actin-capping protein subunit alpha-1 OS=Homo sapiens GN=CAPZA1 PE=1 SV=3 - [CAZA1_HUMAN] | 7 | 9 | 9 |
| P12004 | Proliferating cell nuclear antigen OS=Homo sapiens GN=PCNA PE=1 SV=1 - [PCNA_HUMAN] | 7 | 9 | 9 |
| Q16181 | Septin-7 OS=Homo sapiens GN=SEPT7 PE=1 SV=2 - [SEPT7_HUMAN] | 5 | 9 | 9 |
| Q99729 | Heterogeneous nuclear ribonucleoprotein A/B OS=Homo sapiens GN=HNRNPAB PE=1 SV=2 - [ROAA_HUMAN] | 9 | 8 | 9 |
| P26639 | Threonyl-tRNA synthetase, cytoplasmic OS=Homo sapiens GN=TARS PE=1 SV=3 - [SYTC_HUMAN] | 9 | 8 | 9 |
| Q9UNZ2 | NSFL1 cofactor p47 OS=Homo sapiens GN=NSFL1C PE=1 SV=2 - [NSF1C_HUMAN] | 7 | 8 | 9 |
| Q14566 | DNA replication licensing factor MCM6 OS=Homo sapiens GN=MCM6 PE=1 SV=1 - [MCM6_HUMAN] | 6 | 8 | 9 |
| P08195 | 4F2 cell-surface antigen heavy chain OS=Homo sapiens GN=SLC3A2 PE=1 SV=3 - [4F2_HUMAN] | 12 | 7 | 9 |
| Q07021 | Complement component 1 Q subcomponent-binding protein, mitochondrial OS=Homo sapiens GN=C1QBP PE=1 SV=1 - [C1QBP_HUMAN] | 11 | 7 | 9 |
| Q99733 | Nucleosome assembly protein 1-like 4 OS=Homo sapiens GN=NAP1L4 PE=1 SV=1 - [NP1L4_HUMAN] | 11 | 7 | 9 |
| P60228 | Eukaryotic translation initiation factor 3 subunit E OS=Homo sapiens GN=EIF3E PE=1 SV=1 - [EIF3E_HUMAN] | 9 | 7 | 9 |
| P62269 | 40S ribosomal protein S18 OS=Homo sapiens GN=RPS18 PE=1 SV=3 - [RS18_HUMAN] | 8 | 7 | 9 |
| P29966 | Myristoylated alanine-rich C-kinase substrate OS=Homo sapiens GN=MARCKS PE=1 SV=4 - [MARCS_HUMAN] | 8 | 7 | 9 |
| P23396 | 40S ribosomal protein S3 OS=Homo sapiens GN=RPS3 PE=1 SV=2 - [RS3_HUMAN] | 4 | 7 | 9 |
| P15880 | 40S ribosomal protein S2 OS=Homo sapiens GN=RPS2 PE=1 SV=2 - [RS2_HUMAN] | 11 | 6 | 9 |
| P52209 | 6-phosphogluconate dehydrogenase, decarboxylating OS=Homo sapiens GN=PGD PE=1 SV=3 - [6PGD_HUMAN] | 11 | 6 | 9 |
| P30048 | Thioredoxin-dependent peroxide reductase, mitochondrial OS=Homo sapiens GN=PRDX3 PE=1 SV=3 - [PRDX3_HUMAN] | 11 | 6 | 9 |
| P61158 | Actin-related protein 3 OS=Homo sapiens GN=ACTR3 PE=1 SV=3 - [ARP3_HUMAN] | 7 | 6 | 9 |
| Q9Y266 | Nuclear migration protein nudC OS=Homo sapiens GN=NUDC PE=1 SV=1 - [NUDC_HUMAN] | 7 | 6 | 9 |
| Q13185 | Chromobox protein homolog 3 OS=Homo sapiens GN=CBX3 PE=1 SV=4 - [CBX3_HUMAN] | 5 | 6 | 9 |
| Q15185 | Prostaglandin E synthase 3 OS=Homo sapiens GN=PTGES3 PE=1 SV=1 - [TEBP_HUMAN] | 10 | 5 | 9 |
| P46781 | 40S ribosomal protein S9 OS=Homo sapiens GN=RPS9 PE=1 SV=3 - [RS9_HUMAN] | 8 | 5 | 9 |
| Q13151 | Heterogeneous nuclear ribonucleoprotein A0 OS=Homo sapiens GN=HNRNPA0 PE=1 SV=1 - [ROA0_HUMAN] | 8 | 5 | 9 |
| Q9BWF3 | RNA-binding protein 4 OS=Homo sapiens GN=RBM4 PE=1 SV=1 - [RBM4_HUMAN] | 7 | 5 | 9 |
| P16615 | Sarcoplasmic/endoplasmic reticulum calcium ATPase 2 OS=Homo sapiens GN=ATP2A2 PE=1 SV=1 - [AT2A2_HUMAN] | 5 | 5 | 9 |
| Q9UKX7 | Nuclear pore complex protein Nup50 OS=Homo sapiens GN=NUP50 PE=1 SV=2 - [NUP50_HUMAN] | 4 | 5 | 9 |
| Q9UNF1 | Melanoma-associated antigen D2 OS=Homo sapiens GN=MAGED2 PE=1 SV=2 - [MAGD2_HUMAN] | 1 | 5 | 9 |
| P00492 | Hypoxanthine-guanine phosphoribosyltransferase OS=Homo sapiens GN=HPRT1 PE=1 SV=2 - [HPRT_HUMAN] | 11 | 4 | 9 |
| P68400 | Casein kinase II subunit alpha OS=Homo sapiens GN=CSNK2A1 PE=1 SV=1 - [CSK21_HUMAN] | 8 | 4 | 9 |
| P61586 | Transforming protein RhoA OS=Homo sapiens GN=RHOA PE=1 SV=1 - [RHOA_HUMAN] | 8 | 4 | 9 |
| P25685 | DnaJ homolog subfamily B member 1 OS=Homo sapiens GN=DNAJB1 PE=1 SV=4 - [DNJB1_HUMAN] | 5 | 4 | 9 |
| P48047 | ATP synthase subunit O, mitochondrial OS=Homo sapiens GN=ATP5O PE=1 SV=1 - [ATPO_HUMAN] | 10 | 3 | 9 |
| P20290 | Transcription factor BTF3 OS=Homo sapiens GN=BTF3 PE=1 SV=1 - [BTF3_HUMAN] | 9 | 3 | 9 |
| P05023 | Sodium/potassium-transporting ATPase subunit alpha-1 OS=Homo sapiens GN=ATP1A1 PE=1 SV=1 - [AT1A1_HUMAN] | 3 | 12 | 8 |
| P60842 | Eukaryotic initiation factor 4A-I OS=Homo sapiens GN=EIF4A1 PE=1 SV=1 - [IF4A1_HUMAN] | 9 | 9 | 8 |
| Q13247 | Splicing factor, arginine/serine-rich 6 OS=Homo sapiens GN=SFRS6 PE=1 SV=2 - [SFRS6_HUMAN] | 6 | 9 | 8 |
| P22102 | Trifunctional purine biosynthetic protein adenosine-3 OS=Homo sapiens GN=GART PE=1 SV=1 - [PUR2_HUMAN] | 4 | 9 | 8 |
| P13010 | ATP-dependent DNA helicase 2 subunit 2 OS=Homo sapiens GN=XRCC5 PE=1 SV=3 - [KU86_HUMAN] | 18 | 8 | 8 |
| P41250 | Glycine--tRNA ligase OS=Homo sapiens GN=GARS PE=1 SV=3 - [SYG_HUMAN] | 18 | 8 | 8 |
| O75874 | Isocitrate dehydrogenase [NADP] cytoplasmic OS=Homo sapiens GN=IDH1 PE=1 SV=2 - [IDHC_HUMAN] | 11 | 8 | 8 |
| P30084 | Enoyl-CoA hydratase, mitochondrial OS=Homo sapiens GN=ECHS1 PE=1 SV=4 - [ECHM_HUMAN] | 8 | 8 | 8 |
| Q15459 | Splicing factor 3A subunit 1 OS=Homo sapiens GN=SF3A1 PE=1 SV=1 - [SF3A1_HUMAN] | 8 | 8 | 8 |
| O14818 | Proteasome subunit alpha type-7 OS=Homo sapiens GN=PSMA7 PE=1 SV=1 - [PSA7_HUMAN] | 7 | 8 | 8 |
| Q01518 | Adenylyl cyclase-associated protein 1 OS=Homo sapiens GN=CAP1 PE=1 SV=5 - [CAP1_HUMAN] | 4 | 8 | 8 |
| Q9BXP5 | Serrate RNA effector molecule homolog OS=Homo sapiens GN=SRRT PE=1 SV=1 - [SRRT_HUMAN] | 2 | 8 | 8 |
| P08708 | 40S ribosomal protein S17 OS=Homo sapiens GN=RPS17 PE=1 SV=2 - [RS17_HUMAN] | 13 | 7 | 8 |
| P31150 | Rab GDP dissociation inhibitor alpha OS=Homo sapiens GN=GDI1 PE=1 SV=2 - [GDIA_HUMAN] | 13 | 7 | 8 |
| P04632 | Calpain small subunit 1 OS=Homo sapiens GN=CAPNS1 PE=1 SV=1 - [CPNS1_HUMAN] | 7 | 7 | 8 |
| Q15233 | Non-POU domain-containing octamer-binding protein OS=Homo sapiens GN=NONO PE=1 SV=4 - [NONO_HUMAN] | 7 | 7 | 8 |
| Q14257 | Reticulocalbin-2 OS=Homo sapiens GN=RCN2 PE=1 SV=1 - [RCN2_HUMAN] | 6 | 7 | 8 |
| Q14247 | Src substrate cortactin OS=Homo sapiens GN=CTTN PE=1 SV=2 - [SRC8_HUMAN] | 4 | 7 | 8 |
| Q08J23 | tRNA (cytosine-5-)-methyltransferase NSUN2 OS=Homo sapiens GN=NSUN2 PE=1 SV=2 - [NSUN2_HUMAN] | 4 | 7 | 8 |
| O75390 | Citrate synthase, mitochondrial OS=Homo sapiens GN=CS PE=1 SV=2 - [CISY_HUMAN] | 10 | 6 | 8 |
| Q13561 | Dynactin subunit 2 OS=Homo sapiens GN=DCTN2 PE=1 SV=4 - [DCTN2_HUMAN] | 10 | 6 | 8 |
| P38919 | Eukaryotic initiation factor 4A-III OS=Homo sapiens GN=EIF4A3 PE=1 SV=4 - [IF4A3_HUMAN] | 9 | 6 | 8 |
| Q9Y2Z0 | Suppressor of G2 allele of SKP1 homolog OS=Homo sapiens GN=SUGT1 PE=1 SV=3 - [SUGT1_HUMAN] | 9 | 6 | 8 |
| O00629 | Importin subunit alpha-4 OS=Homo sapiens GN=KPNA4 PE=1 SV=1 - [IMA4_HUMAN] | 8 | 6 | 8 |
| P50213 | Isocitrate dehydrogenase [NAD] subunit alpha, mitochondrial OS=Homo sapiens GN=IDH3A PE=1 SV=1 - [IDH3A_HUMAN] | 8 | 6 | 8 |
| Q9Y5M8 | Signal recognition particle receptor subunit beta OS=Homo sapiens GN=SRPRB PE=1 SV=3 - [SRPRB_HUMAN] | 8 | 6 | 8 |
| O43852 | Calumenin OS=Homo sapiens GN=CALU PE=1 SV=2 - [CALU_HUMAN] | 7 | 6 | 8 |
| Q9Y6E2 | Basic leucine zipper and W2 domain-containing protein 2 OS=Homo sapiens GN=BZW2 PE=1 SV=1 - [BZW2_HUMAN] | 6 | 6 | 8 |
| O76021 | Ribosomal L1 domain-containing protein 1 OS=Homo sapiens GN=RSL1D1 PE=1 SV=3 - [RL1D1_HUMAN] | 4 | 6 | 8 |
| Q9UHB9 | Signal recognition particle 68 kDa protein OS=Homo sapiens GN=SRP68 PE=1 SV=2 - [SRP68_HUMAN] | 4 | 6 | 8 |
| Q92922 | SWI/SNF complex subunit SMARCC1 OS=Homo sapiens GN=SMARCC1 PE=1 SV=3 - [SMRC1_HUMAN] | 1 | 6 | 8 |
| Q9Y281 | Cofilin-2 OS=Homo sapiens GN=CFL2 PE=1 SV=1 - [COF2_HUMAN] | 10 | 5 | 8 |
| P07954 | Fumarate hydratase, mitochondrial OS=Homo sapiens GN=FH PE=1 SV=3 - [FUMH_HUMAN] | 10 | 5 | 8 |
| Q8IUE6 | Histone H2A type 2-B OS=Homo sapiens GN=HIST2H2AB PE=1 SV=3 - [H2A2B_HUMAN] | 10 | 5 | 8 |
| Q86V81 | THO complex subunit 4 OS=Homo sapiens GN=THOC4 PE=1 SV=3 - [THOC4_HUMAN] | 10 | 5 | 8 |
| P40939 | Trifunctional enzyme subunit alpha, mitochondrial OS=Homo sapiens GN=HADHA PE=1 SV=2 - [ECHA_HUMAN] | 10 | 5 | 8 |
| P31930 | Cytochrome b-c1 complex subunit 1, mitochondrial OS=Homo sapiens GN=UQCRC1 PE=1 SV=3 - [QCR1_HUMAN] | 8 | 5 | 8 |
| O00499 | Myc box-dependent-interacting protein 1 OS=Homo sapiens GN=BIN1 PE=1 SV=1 - [BIN1_HUMAN] | 7 | 5 | 8 |
| P43246 | DNA mismatch repair protein Msh2 OS=Homo sapiens GN=MSH2 PE=1 SV=1 - [MSH2_HUMAN] | 6 | 5 | 8 |
| O43837 | Isocitrate dehydrogenase [NAD] subunit beta, mitochondrial OS=Homo sapiens GN=IDH3B PE=1 SV=2 - [IDH3B_HUMAN] | 5 | 5 | 8 |
| Q10567 | AP-1 complex subunit beta-1 OS=Homo sapiens GN=AP1B1 PE=1 SV=2 - [AP1B1_HUMAN] | 0 | 5 | 8 |
| P05387 | 60S acidic ribosomal protein P2 OS=Homo sapiens GN=RPLP2 PE=1 SV=1 - [RLA2_HUMAN] | 11 | 4 | 8 |
| P52306 | Rap1 GTPase-GDP dissociation stimulator 1 OS=Homo sapiens GN=RAP1GDS1 PE=1 SV=3 - [GDS1_HUMAN] | 8 | 4 | 8 |
| P31689 | DnaJ homolog subfamily A member 1 OS=Homo sapiens GN=DNAJA1 PE=1 SV=2 - [DNJA1_HUMAN] | 7 | 4 | 8 |
| P17980 | 26S protease regulatory subunit 6A OS=Homo sapiens GN=PSMC3 PE=1 SV=3 - [PRS6A_HUMAN] | 6 | 4 | 8 |
| P00568 | Adenylate kinase isoenzyme 1 OS=Homo sapiens GN=AK1 PE=1 SV=3 - [KAD1_HUMAN] | 6 | 4 | 8 |
| O43809 | Cleavage and polyadenylation specificity factor subunit 5 OS=Homo sapiens GN=NUDT21 PE=1 SV=1 - [CPSF5_HUMAN] | 15 | 3 | 8 |
| Q9BTT0 | Acidic leucine-rich nuclear phosphoprotein 32 family member E OS=Homo sapiens GN=ANP32E PE=1 SV=1 - [AN32E_HUMAN] | 11 | 3 | 8 |
| Q12904 | Aminoacyl tRNA synthetase complex-interacting multifunctional protein 1 OS=Homo sapiens GN=AIMP1 PE=1 SV=2 - [AIMP1_HUMAN] | 9 | 3 | 8 |
| Q9UL46 | Proteasome activator complex subunit 2 OS=Homo sapiens GN=PSME2 PE=1 SV=4 - [PSME2_HUMAN] | 9 | 3 | 8 |
| P06493 | Cyclin-dependent kinase 1 OS=Homo sapiens GN=CDK1 PE=1 SV=3 - [CDK1_HUMAN] | 8 | 3 | 8 |
| Q8NC51 | Plasminogen activator inhibitor 1 RNA-binding protein OS=Homo sapiens GN=SERBP1 PE=1 SV=2 - [PAIRB_HUMAN] | 7 | 3 | 8 |
| Q14693 | Phosphatidate phosphatase LPIN1 OS=Homo sapiens GN=LPIN1 PE=2 SV=2 - [LPIN1_HUMAN] | 6 | 3 | 8 |
| O43290 | U4/U6.U5 tri-snRNP-associated protein 1 OS=Homo sapiens GN=SART1 PE=1 SV=1 - [SNUT1_HUMAN] | 4 | 3 | 8 |
| Q05682 | Caldesmon OS=Homo sapiens GN=CALD1 PE=1 SV=3 - [CALD1_HUMAN] | 2 | 3 | 8 |
| P98175 | RNA-binding protein 10 OS=Homo sapiens GN=RBM10 PE=1 SV=3 - [RBM10_HUMAN] | 1 | 3 | 8 |
| Q9UQE7 | Structural maintenance of chromosomes protein 3 OS=Homo sapiens GN=SMC3 PE=1 SV=2 - [SMC3_HUMAN] | 1 | 3 | 8 |
| O15042 | U2-associated protein SR140 OS=Homo sapiens GN=SR140 PE=1 SV=2 - [SR140_HUMAN] | 6 | 2 | 8 |
| P43487 | Ran-specific GTPase-activating protein OS=Homo sapiens GN=RANBP1 PE=1 SV=1 - [RANG_HUMAN] | 5 | 2 | 8 |
| Q09028 | Histone-binding protein RBBP4 OS=Homo sapiens GN=RBBP4 PE=1 SV=3 - [RBBP4_HUMAN] | 13 | 10 | 7 |
| Q14240 | Eukaryotic initiation factor 4A-II OS=Homo sapiens GN=EIF4A2 PE=1 SV=2 - [IF4A2_HUMAN] | 10 | 10 | 7 |
| P62333 | 26S protease regulatory subunit S10B OS=Homo sapiens GN=PSMC6 PE=1 SV=1 - [PRS10_HUMAN] | 9 | 10 | 7 |
| Q12874 | Splicing factor 3A subunit 3 OS=Homo sapiens GN=SF3A3 PE=1 SV=1 - [SF3A3_HUMAN] | 15 | 9 | 7 |
| P80723 | Brain acid soluble protein 1 OS=Homo sapiens GN=BASP1 PE=1 SV=2 - [BASP1_HUMAN] | 10 | 8 | 7 |
| Q01105 | Protein SET OS=Homo sapiens GN=SET PE=1 SV=3 - [SET_HUMAN] | 7 | 8 | 7 |
| Q07866 | Kinesin light chain 1 OS=Homo sapiens GN=KLC1 PE=1 SV=2 - [KLC1_HUMAN] | 16 | 7 | 7 |
| P61088 | Ubiquitin-conjugating enzyme E2 N OS=Homo sapiens GN=UBE2N PE=1 SV=1 - [UBE2N_HUMAN] | 11 | 7 | 7 |
| Q8NBS9 | Thioredoxin domain-containing protein 5 OS=Homo sapiens GN=TXNDC5 PE=1 SV=2 - [TXND5_HUMAN] | 8 | 7 | 7 |
| P42765 | 3-ketoacyl-CoA thiolase, mitochondrial OS=Homo sapiens GN=ACAA2 PE=1 SV=2 - [THIM_HUMAN] | 7 | 7 | 7 |
| Q16576 | Histone-binding protein RBBP7 OS=Homo sapiens GN=RBBP7 PE=1 SV=1 - [RBBP7_HUMAN] | 7 | 7 | 7 |
| O00505 | Importin subunit alpha-3 OS=Homo sapiens GN=KPNA3 PE=1 SV=2 - [IMA3_HUMAN] | 7 | 7 | 7 |
| Q08211 | ATP-dependent RNA helicase A OS=Homo sapiens GN=DHX9 PE=1 SV=4 - [DHX9_HUMAN] | 6 | 7 | 7 |
| P62906 | 60S ribosomal protein L10a OS=Homo sapiens GN=RPL10A PE=1 SV=2 - [RL10A_HUMAN] | 5 | 7 | 7 |
| O95232 | Luc7-like protein 3 OS=Homo sapiens GN=LUC7L3 PE=1 SV=2 - [LC7L3_HUMAN] | 5 | 7 | 7 |
| Q9UBB4 | Ataxin-10 OS=Homo sapiens GN=ATXN10 PE=1 SV=1 - [ATX10_HUMAN] | 12 | 6 | 7 |
| P60900 | Proteasome subunit alpha type-6 OS=Homo sapiens GN=PSMA6 PE=1 SV=1 - [PSA6_HUMAN] | 8 | 6 | 7 |
| Q9UBE0 | SUMO-activating enzyme subunit 1 OS=Homo sapiens GN=SAE1 PE=1 SV=1 - [SAE1_HUMAN] | 8 | 6 | 7 |
| P25786 | Proteasome subunit alpha type-1 OS=Homo sapiens GN=PSMA1 PE=1 SV=1 - [PSA1_HUMAN] | 7 | 6 | 7 |
| P55036 | 26S proteasome non-ATPase regulatory subunit 4 OS=Homo sapiens GN=PSMD4 PE=1 SV=1 - [PSMD4_HUMAN] | 6 | 6 | 7 |
| P17812 | CTP synthase 1 OS=Homo sapiens GN=CTPS PE=1 SV=2 - [PYRG1_HUMAN] | 6 | 6 | 7 |
| Q13765 | Nascent polypeptide-associated complex subunit alpha OS=Homo sapiens GN=NACA PE=1 SV=1 - [NACA_HUMAN] | 6 | 6 | 7 |
| P51148 | Ras-related protein Rab-5C OS=Homo sapiens GN=RAB5C PE=1 SV=2 - [RAB5C_HUMAN] | 6 | 6 | 7 |
| O94925 | Glutaminase kidney isoform, mitochondrial OS=Homo sapiens GN=GLS PE=1 SV=1 - [GLSK_HUMAN] | 5 | 6 | 7 |
| P10515 | Dihydrolipoyllysine-residue acetyltransferase component of pyruvate dehydrogenase complex, mitochondrial OS=Homo sapiens GN=DLAT PE=1 SV=3 - [ODP2_HUMAN] | 4 | 6 | 7 |
| O60313 | Dynamin-like 120 kDa protein, mitochondrial OS=Homo sapiens GN=OPA1 PE=1 SV=3 - [OPA1_HUMAN] | 3 | 6 | 7 |
| Q14684 | Ribosomal RNA processing protein 1 homolog B OS=Homo sapiens GN=RRP1B PE=1 SV=3 - [RRP1B_HUMAN] | 2 | 6 | 7 |
| Q01469 | Fatty acid-binding protein, epidermal OS=Homo sapiens GN=FABP5 PE=1 SV=3 - [FABP5_HUMAN] | 13 | 5 | 7 |
| P61163 | Alpha-centractin OS=Homo sapiens GN=ACTR1A PE=1 SV=1 - [ACTZ_HUMAN] | 10 | 5 | 7 |
| P16949 | Stathmin OS=Homo sapiens GN=STMN1 PE=1 SV=3 - [STMN1_HUMAN] | 9 | 5 | 7 |
| Q96FW1 | Ubiquitin thioesterase OTUB1 OS=Homo sapiens GN=OTUB1 PE=1 SV=2 - [OTUB1_HUMAN] | 9 | 5 | 7 |
| P26368 | Splicing factor U2AF 65 kDa subunit OS=Homo sapiens GN=U2AF2 PE=1 SV=4 - [U2AF2_HUMAN] | 8 | 5 | 7 |
| P08621 | U1 small nuclear ribonucleoprotein 70 kDa OS=Homo sapiens GN=SNRNP70 PE=1 SV=2 - [RU17_HUMAN] | 8 | 5 | 7 |
| P49327 | Fatty acid synthase OS=Homo sapiens GN=FASN PE=1 SV=3 - [FAS_HUMAN] | 7 | 5 | 7 |
| P54578 | Ubiquitin carboxyl-terminal hydrolase 14 OS=Homo sapiens GN=USP14 PE=1 SV=3 - [UBP14_HUMAN] | 7 | 5 | 7 |
| Q00534 | Cell division protein kinase 6 OS=Homo sapiens GN=CDK6 PE=1 SV=1 - [CDK6_HUMAN] | 6 | 5 | 7 |
| O00303 | Eukaryotic translation initiation factor 3 subunit F OS=Homo sapiens GN=EIF3F PE=1 SV=1 - [EIF3F_HUMAN] | 6 | 5 | 7 |
| Q9H0U4 | Ras-related protein Rab-1B OS=Homo sapiens GN=RAB1B PE=1 SV=1 - [RAB1B_HUMAN] | 6 | 5 | 7 |
| Q9HC38 | Glyoxalase domain-containing protein 4 OS=Homo sapiens GN=GLOD4 PE=1 SV=1 - [GLOD4_HUMAN] | 5 | 5 | 7 |
| Q9Y3F4 | Serine-threonine kinase receptor-associated protein OS=Homo sapiens GN=STRAP PE=1 SV=1 - [STRAP_HUMAN] | 5 | 5 | 7 |
| P35613 | Basigin OS=Homo sapiens GN=BSG PE=1 SV=2 - [BASI_HUMAN] | 4 | 5 | 7 |
| O60749 | Sorting nexin-2 OS=Homo sapiens GN=SNX2 PE=1 SV=2 - [SNX2_HUMAN] | 4 | 5 | 7 |
| Q08945 | FACT complex subunit SSRP1 OS=Homo sapiens GN=SSRP1 PE=1 SV=1 - [SSRP1_HUMAN] | 3 | 5 | 7 |
| P11310 | Medium-chain specific acyl-CoA dehydrogenase, mitochondrial OS=Homo sapiens GN=ACADM PE=1 SV=1 - [ACADM_HUMAN] | 3 | 5 | 7 |
| O75400 | Pre-mRNA-processing factor 40 homolog A OS=Homo sapiens GN=PRPF40A PE=1 SV=2 - [PR40A_HUMAN] | 3 | 5 | 7 |
| Q15424 | Scaffold attachment factor B1 OS=Homo sapiens GN=SAFB PE=1 SV=4 - [SAFB1_HUMAN] | 3 | 5 | 7 |
| Q13126 | S-methyl-5'-thioadenosine phosphorylase OS=Homo sapiens GN=MTAP PE=1 SV=2 - [MTAP_HUMAN] | 3 | 5 | 7 |
| O75821 | Eukaryotic translation initiation factor 3 subunit G OS=Homo sapiens GN=EIF3G PE=1 SV=2 - [EIF3G_HUMAN] | 2 | 5 | 7 |
| Q9BZZ5 | Apoptosis inhibitor 5 OS=Homo sapiens GN=API5 PE=1 SV=3 - [API5_HUMAN] | 9 | 4 | 7 |
| P22695 | Cytochrome b-c1 complex subunit 2, mitochondrial OS=Homo sapiens GN=UQCRC2 PE=1 SV=3 - [QCR2_HUMAN] | 9 | 4 | 7 |
| Q04760 | Lactoylglutathione lyase OS=Homo sapiens GN=GLO1 PE=1 SV=4 - [LGUL_HUMAN] | 8 | 4 | 7 |
| Q9P2K5 | Myelin expression factor 2 OS=Homo sapiens GN=MYEF2 PE=1 SV=3 - [MYEF2_HUMAN] | 6 | 4 | 7 |
| Q9UKK9 | ADP-sugar pyrophosphatase OS=Homo sapiens GN=NUDT5 PE=1 SV=1 - [NUDT5_HUMAN] | 5 | 4 | 7 |
| Q9Y3I0 | UPF0027 protein C22orf28 OS=Homo sapiens GN=C22orf28 PE=1 SV=1 - [CV028_HUMAN] | 5 | 4 | 7 |
| Q9GZS3 | WD repeat-containing protein 61 OS=Homo sapiens GN=WDR61 PE=1 SV=1 - [WDR61_HUMAN] | 5 | 4 | 7 |
| Q9H3N1 | Thioredoxin-related transmembrane protein 1 OS=Homo sapiens GN=TMX1 PE=1 SV=1 - [TMX1_HUMAN] | 3 | 4 | 7 |
| O75534 | Cold shock domain-containing protein E1 OS=Homo sapiens GN=CSDE1 PE=1 SV=2 - [CSDE1_HUMAN] | 1 | 4 | 7 |
| P15121 | Aldose reductase OS=Homo sapiens GN=AKR1B1 PE=1 SV=3 - [ALDR_HUMAN] | 8 | 3 | 7 |
| O75340 | Programmed cell death protein 6 OS=Homo sapiens GN=PDCD6 PE=1 SV=1 - [PDCD6_HUMAN] | 7 | 3 | 7 |
| P28066 | Proteasome subunit alpha type-5 OS=Homo sapiens GN=PSMA5 PE=1 SV=3 - [PSA5_HUMAN] | 7 | 3 | 7 |
| Q9Y5K5 | Ubiquitin carboxyl-terminal hydrolase isozyme L5 OS=Homo sapiens GN=UCHL5 PE=1 SV=3 - [UCHL5_HUMAN] | 7 | 3 | 7 |
| Q13148 | TAR DNA-binding protein 43 OS=Homo sapiens GN=TARDBP PE=1 SV=1 - [TADBP_HUMAN] | 6 | 3 | 7 |
| Q13573 | SNW domain-containing protein 1 OS=Homo sapiens GN=SNW1 PE=1 SV=1 - [SNW1_HUMAN] | 5 | 3 | 7 |
| P49959 | Double-strand break repair protein MRE11A OS=Homo sapiens GN=MRE11A PE=1 SV=3 - [MRE11_HUMAN] | 4 | 3 | 7 |
| Q8NEJ9 | Neuroguidin OS=Homo sapiens GN=NGDN PE=1 SV=1 - [NGDN_HUMAN] | 3 | 3 | 7 |
| P49756 | RNA-binding protein 25 OS=Homo sapiens GN=RBM25 PE=1 SV=3 - [RBM25_HUMAN] | 2 | 3 | 7 |
| Q96I25 | Splicing factor 45 OS=Homo sapiens GN=RBM17 PE=1 SV=1 - [SPF45_HUMAN] | 2 | 3 | 7 |
| P17677 | Neuromodulin OS=Homo sapiens GN=GAP43 PE=1 SV=1 - [NEUM_HUMAN] | 1 | 3 | 7 |
| Q96KB5 | Lymphokine-activated killer T-cell-originated protein kinase OS=Homo sapiens GN=PBK PE=1 SV=3 - [TOPK_HUMAN] | 0 | 3 | 7 |
| Q8WU90 | Zinc finger CCCH domain-containing protein 15 OS=Homo sapiens GN=ZC3H15 PE=1 SV=1 - [ZC3HF_HUMAN] | 8 | 2 | 7 |
| P30520 | Adenylosuccinate synthetase isozyme 2 OS=Homo sapiens GN=ADSS PE=1 SV=3 - [PURA2_HUMAN] | 3 | 2 | 7 |
| Q9UKV3 | Apoptotic chromatin condensation inducer in the nucleus OS=Homo sapiens GN=ACIN1 PE=1 SV=2 - [ACINU_HUMAN] | 3 | 2 | 7 |
| P62633 | Cellular nucleic acid-binding protein OS=Homo sapiens GN=CNBP PE=1 SV=1 - [CNBP_HUMAN] | 3 | 2 | 7 |
| Q9BY77 | Polymerase delta-interacting protein 3 OS=Homo sapiens GN=POLDIP3 PE=1 SV=2 - [PDIP3_HUMAN] | 3 | 2 | 7 |
| O76094 | Signal recognition particle 72 kDa protein OS=Homo sapiens GN=SRP72 PE=1 SV=3 - [SRP72_HUMAN] | 3 | 2 | 7 |
| O60610 | Protein diaphanous homolog 1 OS=Homo sapiens GN=DIAPH1 PE=1 SV=2 - [DIAP1_HUMAN] | 0 | 2 | 7 |
| Q9UQ35 | Serine/arginine repetitive matrix protein 2 OS=Homo sapiens GN=SRRM2 PE=1 SV=2 - [SRRM2_HUMAN] | 0 | 2 | 7 |
| Q7L0Y3 | Mitochondrial ribonuclease P protein 1 OS=Homo sapiens GN=RG9MTD1 PE=1 SV=2 - [MRRP1_HUMAN] | 10 | 1 | 7 |
| Q9UBQ5 | Eukaryotic translation initiation factor 3 subunit K OS=Homo sapiens GN=EIF3K PE=1 SV=1 - [EIF3K_HUMAN] | 6 | 1 | 7 |
| Q13907 | Isopentenyl-diphosphate Delta-isomerase 1 OS=Homo sapiens GN=IDI1 PE=1 SV=2 - [IDI1_HUMAN] | 4 | 1 | 7 |
| P25713 | Metallothionein-3, Growth inhibitory factor, OS=Homo sapiens GN=MT3 PE=1 SV=2 - [MT3_HUMAN] | 3 | 1 | 7 |
| P02786 | Transferrin receptor protein 1 OS=Homo sapiens GN=TFRC PE=1 SV=2 - [TFR1_HUMAN] | 4 | 10 | 6 |
| Q9HAV4 | Exportin-5 OS=Homo sapiens GN=XPO5 PE=1 SV=1 - [XPO5_HUMAN] | 8 | 9 | 6 |
| P28838 | Cytosol aminopeptidase OS=Homo sapiens GN=LAP3 PE=1 SV=3 - [AMPL_HUMAN] | 6 | 9 | 6 |
| Q9BT78 | COP9 signalosome complex subunit 4 OS=Homo sapiens GN=COPS4 PE=1 SV=1 - [CSN4_HUMAN] | 10 | 8 | 6 |
| P55084 | Trifunctional enzyme subunit beta, mitochondrial OS=Homo sapiens GN=HADHB PE=1 SV=3 - [ECHB_HUMAN] | 9 | 8 | 6 |
| P62241 | 40S ribosomal protein S8 OS=Homo sapiens GN=RPS8 PE=1 SV=2 - [RS8_HUMAN] | 6 | 7 | 6 |
| Q8TAT6 | Nuclear protein localization protein 4 homolog OS=Homo sapiens GN=NPLOC4 PE=1 SV=3 - [NPL4_HUMAN] | 6 | 7 | 6 |
| P38117 | Electron transfer flavoprotein subunit beta OS=Homo sapiens GN=ETFB PE=1 SV=3 - [ETFB_HUMAN] | 4 | 7 | 6 |
| P39019 | 40S ribosomal protein S19 OS=Homo sapiens GN=RPS19 PE=1 SV=2 - [RS19_HUMAN] | 10 | 6 | 6 |
| P62277 | 40S ribosomal protein S13 OS=Homo sapiens GN=RPS13 PE=1 SV=2 - [RS13_HUMAN] | 8 | 6 | 6 |
| Q14194 | Dihydropyrimidinase-related protein 1 OS=Homo sapiens GN=CRMP1 PE=1 SV=1 - [DPYL1_HUMAN] | 8 | 6 | 6 |
| P52565 | Rho GDP-dissociation inhibitor 1 OS=Homo sapiens GN=ARHGDIA PE=1 SV=3 - [GDIR1_HUMAN] | 8 | 6 | 6 |
| Q9HDC9 | Adipocyte plasma membrane-associated protein OS=Homo sapiens GN=APMAP PE=1 SV=2 - [APMAP_HUMAN] | 6 | 6 | 6 |
| P24534 | Elongation factor 1-beta OS=Homo sapiens GN=EEF1B2 PE=1 SV=3 - [EF1B_HUMAN] | 6 | 6 | 6 |
| O95747 | Serine/threonine-protein kinase OSR1 OS=Homo sapiens GN=OXSR1 PE=1 SV=1 - [OXSR1_HUMAN] | 6 | 6 | 6 |
| P12955 | Xaa-Pro dipeptidase OS=Homo sapiens GN=PEPD PE=1 SV=3 - [PEPD_HUMAN] | 6 | 6 | 6 |
| P04843 | Dolichyl-diphosphooligosaccharide--protein glycosyltransferase subunit 1 OS=Homo sapiens GN=RPN1 PE=1 SV=1 - [RPN1_HUMAN] | 5 | 6 | 6 |
| P63092 | Guanine nucleotide-binding protein G(s) subunit alpha isoforms short OS=Homo sapiens GN=GNAS PE=1 SV=1 - [GNAS2_HUMAN] | 5 | 6 | 6 |
| P04181 | Ornithine aminotransferase, mitochondrial OS=Homo sapiens GN=OAT PE=1 SV=1 - [OAT_HUMAN] | 5 | 6 | 6 |
| O15355 | Protein phosphatase 1G OS=Homo sapiens GN=PPM1G PE=1 SV=1 - [PPM1G_HUMAN] | 5 | 6 | 6 |
| P11387 | DNA topoisomerase 1 OS=Homo sapiens GN=TOP1 PE=1 SV=2 - [TOP1_HUMAN] | 4 | 6 | 6 |
| P49354 | Protein farnesyltransferase/geranylgeranyltransferase type-1 subunit alpha OS=Homo sapiens GN=FNTA PE=1 SV=1 - [FNTA_HUMAN] | 4 | 6 | 6 |
| Q9Y2W1 | Thyroid hormone receptor-associated protein 3 OS=Homo sapiens GN=THRAP3 PE=1 SV=2 - [TR150_HUMAN] | 4 | 6 | 6 |
| P20042 | Eukaryotic translation initiation factor 2 subunit 2 OS=Homo sapiens GN=EIF2S2 PE=1 SV=2 - [IF2B_HUMAN] | 3 | 6 | 6 |
| Q00796 | Sorbitol dehydrogenase OS=Homo sapiens GN=SORD PE=1 SV=4 - [DHSO_HUMAN] | 2 | 6 | 6 |
| P24666 | Low molecular weight phosphotyrosine protein phosphatase OS=Homo sapiens GN=ACP1 PE=1 SV=3 - [PPAC_HUMAN] | 11 | 5 | 6 |
| Q00688 | Peptidyl-prolyl cis-trans isomerase FKBP3 OS=Homo sapiens GN=FKBP3 PE=1 SV=1 - [FKBP3_HUMAN] | 9 | 5 | 6 |
| O95433 | Activator of 90 kDa heat shock protein ATPase homolog 1 OS=Homo sapiens GN=AHSA1 PE=1 SV=1 - [AHSA1_HUMAN] | 7 | 5 | 6 |
| P68036 | Ubiquitin-conjugating enzyme E2 L3 OS=Homo sapiens GN=UBE2L3 PE=1 SV=1 - [UB2L3_HUMAN] | 7 | 5 | 6 |
| P00505 | Aspartate aminotransferase, mitochondrial OS=Homo sapiens GN=GOT2 PE=1 SV=3 - [AATM_HUMAN] | 6 | 5 | 6 |
| Q96HE7 | ERO1-like protein alpha OS=Homo sapiens GN=ERO1L PE=1 SV=2 - [ERO1A_HUMAN] | 6 | 5 | 6 |
| Q14498 | RNA-binding protein 39 OS=Homo sapiens GN=RBM39 PE=1 SV=2 - [RBM39_HUMAN] | 6 | 5 | 6 |
| P46109 | Crk-like protein OS=Homo sapiens GN=CRKL PE=1 SV=1 - [CRKL_HUMAN] | 5 | 5 | 6 |
| P14866 | Heterogeneous nuclear ribonucleoprotein L OS=Homo sapiens GN=HNRNPL PE=1 SV=2 - [HNRPL_HUMAN] | 5 | 5 | 6 |
| O60684 | Importin subunit alpha-7 OS=Homo sapiens GN=KPNA6 PE=1 SV=1 - [IMA7_HUMAN] | 5 | 5 | 6 |
| Q07666 | KH domain-containing, RNA-binding, signal transduction-associated protein 1 OS=Homo sapiens GN=KHDRBS1 PE=1 SV=1 - [KHDR1_HUMAN] | 5 | 5 | 6 |
| Q13838 | Spliceosome RNA helicase BAT1 OS=Homo sapiens GN=BAT1 PE=1 SV=1 - [UAP56_HUMAN] | 5 | 5 | 6 |
| Q5SSJ5 | Heterochromatin protein 1-binding protein 3 OS=Homo sapiens GN=HP1BP3 PE=1 SV=1 - [HP1B3_HUMAN] | 4 | 5 | 6 |
| O95202 | LETM1 and EF-hand domain-containing protein 1, mitochondrial OS=Homo sapiens GN=LETM1 PE=1 SV=1 - [LETM1_HUMAN] | 4 | 5 | 6 |
| Q99848 | Probable rRNA-processing protein EBP2 OS=Homo sapiens GN=EBNA1BP2 PE=1 SV=2 - [EBP2_HUMAN] | 4 | 5 | 6 |
| P37198 | Nuclear pore glycoprotein p62 OS=Homo sapiens GN=NUP62 PE=1 SV=3 - [NUP62_HUMAN] | 3 | 5 | 6 |
| Q13435 | Splicing factor 3B subunit 2 OS=Homo sapiens GN=SF3B2 PE=1 SV=2 - [SF3B2_HUMAN] | 2 | 5 | 6 |
| Q9BTE3 | Mini-chromosome maintenance complex-binding protein OS=Homo sapiens GN=MCMBP PE=1 SV=2 - [MCMBP_HUMAN] | 1 | 5 | 6 |
| P32969 | 60S ribosomal protein L9 OS=Homo sapiens GN=RPL9 PE=1 SV=1 - [RL9_HUMAN] | 9 | 4 | 6 |
| O95292 | Vesicle-associated membrane protein-associated protein B/C OS=Homo sapiens GN=VAPB PE=1 SV=3 - [VAPB_HUMAN] | 9 | 4 | 6 |
| Q15102 | Platelet-activating factor acetylhydrolase IB subunit gamma OS=Homo sapiens GN=PAFAH1B3 PE=1 SV=1 - [PA1B3_HUMAN] | 8 | 4 | 6 |
| Q9H773 | dCTP pyrophosphatase 1 OS=Homo sapiens GN=DCTPP1 PE=1 SV=1 - [DCTP1_HUMAN] | 6 | 4 | 6 |
| P14314 | Glucosidase 2 subunit beta OS=Homo sapiens GN=PRKCSH PE=1 SV=2 - [GLU2B_HUMAN] | 5 | 4 | 6 |
| Q9NZL9 | Methionine adenosyltransferase 2 subunit beta OS=Homo sapiens GN=MAT2B PE=1 SV=1 - [MAT2B_HUMAN] | 5 | 4 | 6 |
| Q86UE4 | Protein LYRIC OS=Homo sapiens GN=MTDH PE=1 SV=2 - [LYRIC_HUMAN] | 5 | 4 | 6 |
| P14550 | Alcohol dehydrogenase [NADP+] OS=Homo sapiens GN=AKR1A1 PE=1 SV=3 - [AK1A1_HUMAN] | 4 | 4 | 6 |
| Q96F85 | CB1 cannabinoid receptor-interacting protein 1 OS=Homo sapiens GN=CNRIP1 PE=1 SV=1 - [CNRP1_HUMAN] | 4 | 4 | 6 |
| O75937 | DnaJ homolog subfamily C member 8 OS=Homo sapiens GN=DNAJC8 PE=1 SV=2 - [DNJC8_HUMAN] | 4 | 4 | 6 |
| P56537 | Eukaryotic translation initiation factor 6 OS=Homo sapiens GN=EIF6 PE=1 SV=1 - [IF6_HUMAN] | 4 | 4 | 6 |
| Q16629 | Splicing factor, arginine/serine-rich 7 OS=Homo sapiens GN=SFRS7 PE=1 SV=1 - [SFRS7_HUMAN] | 4 | 4 | 6 |
| Q8WWM7 | Ataxin-2-like protein OS=Homo sapiens GN=ATXN2L PE=1 SV=2 - [ATX2L_HUMAN] | 3 | 4 | 6 |
| P54687 | Branched-chain-amino-acid aminotransferase, cytosolic OS=Homo sapiens GN=BCAT1 PE=1 SV=3 - [BCAT1_HUMAN] | 3 | 4 | 6 |
| P62495 | Eukaryotic peptide chain release factor subunit 1 OS=Homo sapiens GN=ETF1 PE=1 SV=3 - [ERF1_HUMAN] | 3 | 4 | 6 |
| Q05519 | Splicing factor, arginine/serine-rich 11 OS=Homo sapiens GN=SFRS11 PE=1 SV=1 - [SFR11_HUMAN] | 3 | 4 | 6 |
| Q9H0D6 | 5'-3' exoribonuclease 2 OS=Homo sapiens GN=XRN2 PE=1 SV=1 - [XRN2_HUMAN] | 2 | 4 | 6 |
| Q96KR1 | Zinc finger RNA-binding protein OS=Homo sapiens GN=ZFR PE=1 SV=2 - [ZFR_HUMAN] | 2 | 4 | 6 |
| Q29RF7 | Sister chromatid cohesion protein PDS5 homolog A OS=Homo sapiens GN=PDS5A PE=1 SV=1 - [PDS5A_HUMAN] | 1 | 4 | 6 |
| Q7L1Q6 | Basic leucine zipper and W2 domain-containing protein 1 OS=Homo sapiens GN=BZW1 PE=1 SV=1 - [BZW1_HUMAN] | 11 | 3 | 6 |
| P11216 | Glycogen phosphorylase, brain form OS=Homo sapiens GN=PYGB PE=1 SV=5 - [PYGB_HUMAN] | 11 | 3 | 6 |
| P63173 | 60S ribosomal protein L38 OS=Homo sapiens GN=RPL38 PE=1 SV=2 - [RL38_HUMAN] | 9 | 3 | 6 |
| Q14444 | Caprin-1 OS=Homo sapiens GN=CAPRIN1 PE=1 SV=2 - [CAPR1_HUMAN] | 8 | 3 | 6 |
| Q8TEX9 | Importin-4 OS=Homo sapiens GN=IPO4 PE=1 SV=2 - [IPO4_HUMAN] | 8 | 3 | 6 |
| P18621 | 60S ribosomal protein L17 OS=Homo sapiens GN=RPL17 PE=1 SV=3 - [RL17_HUMAN] | 7 | 3 | 6 |
| Q7L2H7 | Eukaryotic translation initiation factor 3 subunit M OS=Homo sapiens GN=EIF3M PE=1 SV=1 - [EIF3M_HUMAN] | 7 | 3 | 6 |
| Q15631 | Translin OS=Homo sapiens GN=TSN PE=1 SV=1 - [TSN_HUMAN] | 7 | 3 | 6 |
| Q5SW79 | Centrosomal protein of 170 kDa OS=Homo sapiens GN=CEP170 PE=1 SV=1 - [CE170_HUMAN] | 6 | 3 | 6 |
| Q96KP4 | Cytosolic non-specific dipeptidase OS=Homo sapiens GN=CNDP2 PE=1 SV=2 - [CNDP2_HUMAN] | 6 | 3 | 6 |
| P19105 | Myosin regulatory light chain 12A OS=Homo sapiens GN=MYL12A PE=1 SV=2 - [ML12A_HUMAN] | 6 | 3 | 6 |
| Q9UKM9 | RNA-binding protein Raly OS=Homo sapiens GN=RALY PE=1 SV=1 - [RALY_HUMAN] | 6 | 3 | 6 |
| Q16630 | Cleavage and polyadenylation specificity factor subunit 6 OS=Homo sapiens GN=CPSF6 PE=1 SV=2 - [CPSF6_HUMAN] | 5 | 3 | 6 |
| P49720 | Proteasome subunit beta type-3 OS=Homo sapiens GN=PSMB3 PE=1 SV=2 - [PSB3_HUMAN] | 4 | 3 | 6 |
| P32322 | Pyrroline-5-carboxylate reductase 1, mitochondrial OS=Homo sapiens GN=PYCR1 PE=1 SV=2 - [P5CR1_HUMAN] | 3 | 3 | 6 |
| Q96PZ0 | Pseudouridylate synthase 7 homolog OS=Homo sapiens GN=PUS7 PE=1 SV=2 - [PUS7_HUMAN] | 1 | 3 | 6 |
| Q8WXI9 | Transcriptional repressor p66-beta OS=Homo sapiens GN=GATAD2B PE=1 SV=1 - [P66B_HUMAN] | 1 | 3 | 6 |
| P61204 | ADP-ribosylation factor 3 OS=Homo sapiens GN=ARF3 PE=1 SV=2 - [ARF3_HUMAN] | 0 | 3 | 6 |
| Q02809 | Procollagen-lysine,2-oxoglutarate 5-dioxygenase 1 OS=Homo sapiens GN=PLOD1 PE=1 SV=2 - [PLOD1_HUMAN] | 0 | 3 | 6 |
| Q14151 | Scaffold attachment factor B2 OS=Homo sapiens GN=SAFB2 PE=1 SV=1 - [SAFB2_HUMAN] | 0 | 3 | 6 |
| O75533 | Splicing factor 3B subunit 1 OS=Homo sapiens GN=SF3B1 PE=1 SV=3 - [SF3B1_HUMAN] | 8 | 2 | 6 |
| Q9UMX0 | Ubiquilin-1 OS=Homo sapiens GN=UBQLN1 PE=1 SV=2 - [UBQL1_HUMAN] | 7 | 2 | 6 |
| P30837 | Aldehyde dehydrogenase X, mitochondrial OS=Homo sapiens GN=ALDH1B1 PE=1 SV=3 - [AL1B1_HUMAN] | 6 | 2 | 6 |
| P53634 | Dipeptidyl peptidase 1 OS=Homo sapiens GN=CTSC PE=1 SV=2 - [CATC_HUMAN] | 6 | 2 | 6 |
| O00264 | Membrane-associated progesterone receptor component 1 OS=Homo sapiens GN=PGRMC1 PE=1 SV=3 - [PGRC1_HUMAN] | 6 | 2 | 6 |
| Q92522 | Histone H1x OS=Homo sapiens GN=H1FX PE=1 SV=1 - [H1X_HUMAN] | 3 | 2 | 6 |
| Q9NWY4 | Histone PARylation factor 1 OS=Homo sapiens GN=HPF1 PE=1 SV=2 - [HPF1_HUMAN] | 3 | 2 | 6 |
| Q8NE71 | ATP-binding cassette sub-family F member 1 OS=Homo sapiens GN=ABCF1 PE=1 SV=2 - [ABCF1_HUMAN] | 2 | 2 | 6 |
| Q13596 | Sorting nexin-1 OS=Homo sapiens GN=SNX1 PE=1 SV=3 - [SNX1_HUMAN] | 2 | 2 | 6 |
| O14497 | AT-rich interactive domain-containing protein 1A OS=Homo sapiens GN=ARID1A PE=1 SV=3 - [ARI1A_HUMAN] | 0 | 2 | 6 |
| Q8IY81 | pre-rRNA processing protein FTSJ3 OS=Homo sapiens GN=FTSJ3 PE=1 SV=2 - [SPB1_HUMAN] | 0 | 2 | 6 |
| P21283 | V-type proton ATPase subunit C 1 OS=Homo sapiens GN=ATP6V1C1 PE=1 SV=4 - [VATC1_HUMAN] | 6 | 1 | 6 |
| P49755 | Transmembrane emp24 domain-containing protein 10 OS=Homo sapiens GN=TMED10 PE=1 SV=2 - [TMEDA_HUMAN] | 4 | 1 | 6 |
| P08574 | Cytochrome c1, heme protein, mitochondrial OS=Homo sapiens GN=CYC1 PE=1 SV=3 - [CY1_HUMAN] | 2 | 1 | 6 |
| O00451 | GDNF family receptor alpha-2 OS=Homo sapiens GN=GFRA2 PE=2 SV=2 - [GFRA2_HUMAN] | 2 | 1 | 6 |
| Q9BVJ6 | U3 small nucleolar RNA-associated protein 14 homolog A OS=Homo sapiens GN=UTP14A PE=1 SV=1 - [UT14A_HUMAN] | 2 | 1 | 6 |
| Q8IWX8 | Calcium homeostasis endoplasmic reticulum protein OS=Homo sapiens GN=CHERP PE=1 SV=3 - [CHERP_HUMAN] | 1 | 1 | 6 |
| O94903 | Proline synthetase co-transcribed bacterial homolog protein OS=Homo sapiens GN=PROSC PE=1 SV=1 - [PROSC_HUMAN] | 1 | 1 | 6 |
| Q96TA2 | ATP-dependent metalloprotease YME1L1 OS=Homo sapiens GN=YME1L1 PE=1 SV=2 - [YMEL1_HUMAN] | 0 | 1 | 6 |
| Q96C86 | m7GpppX diphosphatase OS=Homo sapiens GN=DCPS PE=1 SV=2 - [DCPS_HUMAN] | 0 | 1 | 6 |
| Q9Y520 | Protein PRRC2C OS=Homo sapiens GN=PRRC2C PE=1 SV=4 - [PRC2C_HUMAN] | 0 | 1 | 6 |
| Q99543 | DnaJ homolog subfamily C member 2 OS=Homo sapiens GN=DNAJC2 PE=1 SV=4 - [DNJC2_HUMAN] | 1 | 0 | 6 |
| P00367 | Glutamate dehydrogenase 1, mitochondrial OS=Homo sapiens GN=GLUD1 PE=1 SV=2 - [DHE3_HUMAN] | 8 | 8 | 5 |
| P35580 | Myosin-10 OS=Homo sapiens GN=MYH10 PE=1 SV=3 - [MYH10_HUMAN] | 2 | 8 | 5 |
| Q8TAQ2 | SWI/SNF complex subunit SMARCC2 OS=Homo sapiens GN=SMARCC2 PE=1 SV=1 - [SMRC2_HUMAN] | 2 | 8 | 5 |
| Q9P289 | Serine/threonine-protein kinase MST4 OS=Homo sapiens GN=MST4 PE=1 SV=2 - [MST4_HUMAN] | 0 | 8 | 5 |
| P10768 | S-formylglutathione hydrolase OS=Homo sapiens GN=ESD PE=1 SV=2 - [ESTD_HUMAN] | 16 | 7 | 5 |
| P62191 | 26S protease regulatory subunit 4 OS=Homo sapiens GN=PSMC1 PE=1 SV=1 - [PRS4_HUMAN] | 5 | 7 | 5 |
| P31942 | Heterogeneous nuclear ribonucleoprotein H3 OS=Homo sapiens GN=HNRNPH3 PE=1 SV=2 - [HNRH3_HUMAN] | 5 | 7 | 5 |
| O60282 | Kinesin heavy chain isoform 5C OS=Homo sapiens GN=KIF5C PE=1 SV=1 - [KIF5C_HUMAN] | 5 | 7 | 5 |
| P51114 | Fragile X mental retardation syndrome-related protein 1 OS=Homo sapiens GN=FXR1 PE=1 SV=3 - [FXR1_HUMAN] | 3 | 7 | 5 |
| P30566 | Adenylosuccinate lyase OS=Homo sapiens GN=ADSL PE=1 SV=2 - [PUR8_HUMAN] | 8 | 6 | 5 |
| Q5RKV6 | Exosome complex exonuclease MTR3 OS=Homo sapiens GN=EXOSC6 PE=1 SV=1 - [EXOS6_HUMAN] | 5 | 6 | 5 |
| O75306 | NADH dehydrogenase [ubiquinone] iron-sulfur protein 2, mitochondrial OS=Homo sapiens GN=NDUFS2 PE=1 SV=2 - [NDUS2_HUMAN] | 5 | 6 | 5 |
| Q16851 | UTP--glucose-1-phosphate uridylyltransferase OS=Homo sapiens GN=UGP2 PE=1 SV=5 - [UGPA_HUMAN] | 4 | 6 | 5 |
| P61201 | COP9 signalosome complex subunit 2 OS=Homo sapiens GN=COPS2 PE=1 SV=1 - [CSN2_HUMAN] | 3 | 6 | 5 |
| P62995 | Transformer-2 protein homolog beta OS=Homo sapiens GN=TRA2B PE=1 SV=1 - [TRA2B_HUMAN] | 3 | 6 | 5 |
| Q96P70 | Importin-9 OS=Homo sapiens GN=IPO9 PE=1 SV=3 - [IPO9_HUMAN] | 8 | 5 | 5 |
| P62195 | 26S protease regulatory subunit 8 OS=Homo sapiens GN=PSMC5 PE=1 SV=1 - [PRS8_HUMAN] | 7 | 5 | 5 |
| P09622 | Dihydrolipoyl dehydrogenase, mitochondrial OS=Homo sapiens GN=DLD PE=1 SV=2 - [DLDH_HUMAN] | 6 | 5 | 5 |
| Q9BS26 | Endoplasmic reticulum resident protein 44 OS=Homo sapiens GN=ERP44 PE=1 SV=1 - [ERP44_HUMAN] | 6 | 5 | 5 |
| Q13347 | Eukaryotic translation initiation factor 3 subunit I OS=Homo sapiens GN=EIF3I PE=1 SV=1 - [EIF3I_HUMAN] | 6 | 5 | 5 |
| Q96PK6 | RNA-binding protein 14 OS=Homo sapiens GN=RBM14 PE=1 SV=2 - [RBM14_HUMAN] | 6 | 5 | 5 |
| Q5JTV8 | Torsin-1A-interacting protein 1 OS=Homo sapiens GN=TOR1AIP1 PE=1 SV=2 - [TOIP1_HUMAN] | 6 | 5 | 5 |
| Q16698 | 2,4-dienoyl-CoA reductase, mitochondrial OS=Homo sapiens GN=DECR1 PE=1 SV=1 - [DECR_HUMAN] | 5 | 5 | 5 |
| P62851 | 40S ribosomal protein S25 OS=Homo sapiens GN=RPS25 PE=1 SV=1 - [RS25_HUMAN] | 5 | 5 | 5 |
| P60953 | Cell division control protein 42 homolog OS=Homo sapiens GN=CDC42 PE=1 SV=2 - [CDC42_HUMAN] | 5 | 5 | 5 |
| Q15717 | ELAV-like protein 1 OS=Homo sapiens GN=ELAVL1 PE=1 SV=2 - [ELAV1_HUMAN] | 5 | 5 | 5 |
| P46379 | Large proline-rich protein BAT3 OS=Homo sapiens GN=BAT3 PE=1 SV=2 - [BAT3_HUMAN] | 5 | 5 | 5 |
| Q8TBC4 | NEDD8-activating enzyme E1 catalytic subunit OS=Homo sapiens GN=UBA3 PE=1 SV=2 - [UBA3_HUMAN] | 3 | 5 | 5 |
| Q15274 | Nicotinate-nucleotide pyrophosphorylase [carboxylating] OS=Homo sapiens GN=QPRT PE=1 SV=3 - [NADC_HUMAN] | 3 | 5 | 5 |
| Q92599 | Septin-8 OS=Homo sapiens GN=SEPT8 PE=1 SV=4 - [SEPT8_HUMAN] | 3 | 5 | 5 |
| Q9BTV4 | Transmembrane protein 43 OS=Homo sapiens GN=TMEM43 PE=1 SV=1 - [TMM43_HUMAN] | 3 | 5 | 5 |
| P53999 | Activated RNA polymerase II transcriptional coactivator p15 OS=Homo sapiens GN=SUB1 PE=1 SV=3 - [TCP4_HUMAN] | 2 | 5 | 5 |
| Q9UBC2 | Epidermal growth factor receptor substrate 15-like 1 OS=Homo sapiens GN=EPS15L1 PE=1 SV=1 - [EP15R_HUMAN] | 1 | 5 | 5 |
| P00966 | Argininosuccinate synthase OS=Homo sapiens GN=ASS1 PE=1 SV=2 - [ASSY_HUMAN] | 15 | 4 | 5 |
| P15311 | Ezrin OS=Homo sapiens GN=EZR PE=1 SV=4 - [EZRI_HUMAN] | 15 | 4 | 5 |
| Q9Y617 | Phosphoserine aminotransferase OS=Homo sapiens GN=PSAT1 PE=1 SV=2 - [SERC_HUMAN] | 11 | 4 | 5 |
| O43776 | Asparaginyl-tRNA synthetase, cytoplasmic OS=Homo sapiens GN=NARS PE=1 SV=1 - [SYNC_HUMAN] | 9 | 4 | 5 |
| Q13155 | Aminoacyl tRNA synthetase complex-interacting multifunctional protein 2 OS=Homo sapiens GN=AIMP2 PE=1 SV=2 - [AIMP2_HUMAN] | 7 | 4 | 5 |
| P23588 | Eukaryotic translation initiation factor 4B OS=Homo sapiens GN=EIF4B PE=1 SV=2 - [IF4B_HUMAN] | 7 | 4 | 5 |
| Q01081 | Splicing factor U2AF 35 kDa subunit OS=Homo sapiens GN=U2AF1 PE=1 SV=3 - [U2AF1_HUMAN] | 7 | 4 | 5 |
| P61081 | NEDD8-conjugating enzyme Ubc12 OS=Homo sapiens GN=UBE2M PE=1 SV=1 - [UBC12_HUMAN] | 6 | 4 | 5 |
| Q8IZL8 | Proline-, glutamic acid- and leucine-rich protein 1 OS=Homo sapiens GN=PELP1 PE=1 SV=2 - [PELP1_HUMAN] | 6 | 4 | 5 |
| Q53FA7 | Quinone oxidoreductase PIG3 OS=Homo sapiens GN=TP53I3 PE=1 SV=2 - [QORX_HUMAN] | 6 | 4 | 5 |
| Q9UN86 | Ras GTPase-activating protein-binding protein 2 OS=Homo sapiens GN=G3BP2 PE=1 SV=2 - [G3BP2_HUMAN] | 6 | 4 | 5 |
| P50914 | 60S ribosomal protein L14 OS=Homo sapiens GN=RPL14 PE=1 SV=4 - [RL14_HUMAN] | 5 | 4 | 5 |
| Q9BQA1 | Methylosome protein 50 OS=Homo sapiens GN=WDR77 PE=1 SV=1 - [MEP50_HUMAN] | 5 | 4 | 5 |
| Q3ZCQ8 | Mitochondrial import inner membrane translocase subunit TIM50 OS=Homo sapiens GN=TIMM50 PE=1 SV=2 - [TIM50_HUMAN] | 5 | 4 | 5 |
| Q9NXG2 | THUMP domain-containing protein 1 OS=Homo sapiens GN=THUMPD1 PE=1 SV=2 - [THUM1_HUMAN] | 5 | 4 | 5 |
| Q9UHD9 | Ubiquilin-2 OS=Homo sapiens GN=UBQLN2 PE=1 SV=2 - [UBQL2_HUMAN] | 5 | 4 | 5 |
| Q13011 | Delta(3,5)-Delta(2,4)-dienoyl-CoA isomerase, mitochondrial OS=Homo sapiens GN=ECH1 PE=1 SV=2 - [ECH1_HUMAN] | 4 | 4 | 5 |
| P50579 | Methionine aminopeptidase 2 OS=Homo sapiens GN=METAP2 PE=1 SV=1 - [AMPM2_HUMAN] | 4 | 4 | 5 |
| Q13564 | NEDD8-activating enzyme E1 regulatory subunit OS=Homo sapiens GN=NAE1 PE=1 SV=1 - [ULA1_HUMAN] | 4 | 4 | 5 |
| P25789 | Proteasome subunit alpha type-4 OS=Homo sapiens GN=PSMA4 PE=1 SV=1 - [PSA4_HUMAN] | 4 | 4 | 5 |
| Q9H9B4 | Sideroflexin-1 OS=Homo sapiens GN=SFXN1 PE=1 SV=4 - [SFXN1_HUMAN] | 4 | 4 | 5 |
| Q9NZL4 | Hsp70-binding protein 1 OS=Homo sapiens GN=HSPBP1 PE=1 SV=1 - [HPBP1_HUMAN] | 3 | 4 | 5 |
| Q9Y2W2 | WW domain-binding protein 11 OS=Homo sapiens GN=WBP11 PE=1 SV=1 - [WBP11_HUMAN] | 3 | 4 | 5 |
| P39748 | Flap endonuclease 1 OS=Homo sapiens GN=FEN1 PE=1 SV=1 - [FEN1_HUMAN] | 2 | 4 | 5 |
| Q7Z4S6 | Kinesin-like protein KIF21A OS=Homo sapiens GN=KIF21A PE=1 SV=2 - [KI21A_HUMAN] | 2 | 4 | 5 |
| Q15293 | Reticulocalbin-1 OS=Homo sapiens GN=RCN1 PE=1 SV=1 - [RCN1_HUMAN] | 2 | 4 | 5 |
| Q9NVI7 | ATPase family AAA domain-containing protein 3A OS=Homo sapiens GN=ATAD3A PE=1 SV=2 - [ATD3A_HUMAN] | 1 | 4 | 5 |
| Q9NUQ9 | Protein FAM49B OS=Homo sapiens GN=FAM49B PE=1 SV=1 - [FA49B_HUMAN] | 1 | 4 | 5 |
| Q15154 | Pericentriolar material 1 protein OS=Homo sapiens GN=PCM1 PE=1 SV=4 - [PCM1_HUMAN] | 0 | 4 | 5 |
| P30044 | Peroxiredoxin-5, mitochondrial OS=Homo sapiens GN=PRDX5 PE=1 SV=4 - [PRDX5_HUMAN] | 16 | 3 | 5 |
| P05386 | 60S acidic ribosomal protein P1 OS=Homo sapiens GN=RPLP1 PE=1 SV=1 - [RLA1_HUMAN] | 14 | 3 | 5 |
| O60884 | DnaJ homolog subfamily A member 2 OS=Homo sapiens GN=DNAJA2 PE=1 SV=1 - [DNJA2_HUMAN] | 9 | 3 | 5 |
| Q9NQ29 | Putative RNA-binding protein Luc7-like 1 OS=Homo sapiens GN=LUC7L PE=1 SV=1 - [LUC7L_HUMAN] | 9 | 3 | 5 |
| Q15046 | Lysyl-tRNA synthetase OS=Homo sapiens GN=KARS PE=1 SV=3 - [SYK_HUMAN] | 8 | 3 | 5 |
| Q9Y383 | Putative RNA-binding protein Luc7-like 2 OS=Homo sapiens GN=LUC7L2 PE=1 SV=2 - [LC7L2_HUMAN] | 8 | 3 | 5 |
| P18124 | 60S ribosomal protein L7 OS=Homo sapiens GN=RPL7 PE=1 SV=1 - [RL7_HUMAN] | 7 | 3 | 5 |
| P62249 | 40S ribosomal protein S16 OS=Homo sapiens GN=RPS16 PE=1 SV=2 - [RS16_HUMAN] | 6 | 3 | 5 |
| Q6NUK1 | Calcium-binding mitochondrial carrier protein SCaMC-1 OS=Homo sapiens GN=SLC25A24 PE=1 SV=2 - [SCMC1_HUMAN] | 6 | 3 | 5 |
| P28161 | Glutathione S-transferase Mu 2 OS=Homo sapiens GN=GSTM2 PE=1 SV=2 - [GSTM2_HUMAN] | 6 | 3 | 5 |
| Q92542 | Nicastrin OS=Homo sapiens GN=NCSTN PE=1 SV=2 - [NICA_HUMAN] | 6 | 3 | 5 |
| Q15637 | Splicing factor 1 OS=Homo sapiens GN=SF1 PE=1 SV=4 - [SF01_HUMAN] | 6 | 3 | 5 |
| P83731 | 60S ribosomal protein L24 OS=Homo sapiens GN=RPL24 PE=1 SV=1 - [RL24_HUMAN] | 5 | 3 | 5 |
| Q7Z434 | Mitochondrial antiviral-signaling protein OS=Homo sapiens GN=MAVS PE=1 SV=2 - [MAVS_HUMAN] | 5 | 3 | 5 |
| P50897 | Palmitoyl-protein thioesterase 1 OS=Homo sapiens GN=PPT1 PE=1 SV=1 - [PPT1_HUMAN] | 5 | 3 | 5 |
| Q15006 | Tetratricopeptide repeat protein 35 OS=Homo sapiens GN=TTC35 PE=1 SV=1 - [TTC35_HUMAN] | 5 | 3 | 5 |
| P25325 | 3-mercaptopyruvate sulfurtransferase OS=Homo sapiens GN=MPST PE=1 SV=3 - [THTM_HUMAN] | 4 | 3 | 5 |
| P63151 | Serine/threonine-protein phosphatase 2A 55 kDa regulatory subunit B alpha isoform OS=Homo sapiens GN=PPP2R2A PE=1 SV=1 - [2ABA_HUMAN] | 4 | 3 | 5 |
| P19623 | Spermidine synthase OS=Homo sapiens GN=SRM PE=1 SV=1 - [SPEE_HUMAN] | 4 | 3 | 5 |
| Q9P0L0 | Vesicle-associated membrane protein-associated protein A OS=Homo sapiens GN=VAPA PE=1 SV=3 - [VAPA_HUMAN] | 4 | 3 | 5 |
| Q92820 | Gamma-glutamyl hydrolase OS=Homo sapiens GN=GGH PE=1 SV=2 - [GGH_HUMAN] | 3 | 3 | 5 |
| Q9BQ67 | Glutamate-rich WD repeat-containing protein 1 OS=Homo sapiens GN=GRWD1 PE=1 SV=1 - [GRWD1_HUMAN] | 3 | 3 | 5 |
| Q9UHX1 | Poly(U)-binding-splicing factor PUF60 OS=Homo sapiens GN=PUF60 PE=1 SV=1 - [PUF60_HUMAN] | 3 | 3 | 5 |
| P84103 | Splicing factor, arginine/serine-rich 3 OS=Homo sapiens GN=SFRS3 PE=1 SV=1 - [SFRS3_HUMAN] | 3 | 3 | 5 |
| P23193 | Transcription elongation factor A protein 1 OS=Homo sapiens GN=TCEA1 PE=1 SV=2 - [TCEA1_HUMAN] | 3 | 3 | 5 |
| P51784 | Ubiquitin carboxyl-terminal hydrolase 11 OS=Homo sapiens GN=USP11 PE=1 SV=3 - [UBP11_HUMAN] | 3 | 3 | 5 |
| O94888 | UBX domain-containing protein 7 OS=Homo sapiens GN=UBXN7 PE=1 SV=2 - [UBXN7_HUMAN] | 3 | 3 | 5 |
| Q96A33 | Coiled-coil domain-containing protein 47 OS=Homo sapiens GN=CCDC47 PE=1 SV=1 - [CCD47_HUMAN] | 2 | 3 | 5 |
| P29083 | General transcription factor IIE subunit 1 OS=Homo sapiens GN=GTF2E1 PE=1 SV=2 - [T2EA_HUMAN] | 2 | 3 | 5 |
| Q15758 | Neutral amino acid transporter B(0) OS=Homo sapiens GN=SLC1A5 PE=1 SV=2 - [AAAT_HUMAN] | 2 | 3 | 5 |
| P18754 | Regulator of chromosome condensation OS=Homo sapiens GN=RCC1 PE=1 SV=1 - [RCC1_HUMAN] | 2 | 3 | 5 |
| Q9NRN7 | L-aminoadipate-semialdehyde dehydrogenase-phosphopantetheinyl transferase OS=Homo sapiens GN=AASDHPPT PE=1 SV=2 - [ADPPT_HUMAN] | 1 | 3 | 5 |
| O75152 | Zinc finger CCCH domain-containing protein 11A OS=Homo sapiens GN=ZC3H11A PE=1 SV=3 - [ZC11A_HUMAN] | 1 | 3 | 5 |
| Q9C0B1 | Protein fto OS=Homo sapiens GN=FTO PE=1 SV=3 - [FTO_HUMAN] | 9 | 2 | 5 |
| Q9H2U2 | Inorganic pyrophosphatase 2, mitochondrial OS=Homo sapiens GN=PPA2 PE=1 SV=2 - [IPYR2_HUMAN] | 8 | 2 | 5 |
| P60660 | Myosin light polypeptide 6 OS=Homo sapiens GN=MYL6 PE=1 SV=2 - [MYL6_HUMAN] | 8 | 2 | 5 |
| P12532 | Creatine kinase U-type, mitochondrial OS=Homo sapiens GN=CKMT1A PE=1 SV=1 - [KCRU_HUMAN] | 7 | 2 | 5 |
| P13591 | Neural cell adhesion molecule 1 OS=Homo sapiens GN=NCAM1 PE=1 SV=3 - [NCAM1_HUMAN] | 7 | 2 | 5 |
| Q15126 | Phosphomevalonate kinase OS=Homo sapiens GN=PMVK PE=1 SV=3 - [PMVK_HUMAN] | 6 | 2 | 5 |
| P25788 | Proteasome subunit alpha type-3 OS=Homo sapiens GN=PSMA3 PE=1 SV=2 - [PSA3_HUMAN] | 6 | 2 | 5 |
| Q9GZT3 | SRA stem-loop-interacting RNA-binding protein, mitochondrial OS=Homo sapiens GN=SLIRP PE=1 SV=1 - [SLIRP_HUMAN] | 6 | 2 | 5 |
| P46108 | Adapter molecule crk OS=Homo sapiens GN=CRK PE=1 SV=2 - [CRK_HUMAN] | 5 | 2 | 5 |
| P39656 | Dolichyl-diphosphooligosaccharide--protein glycosyltransferase 48 kDa subunit OS=Homo sapiens GN=DDOST PE=1 SV=4 - [OST48_HUMAN] | 4 | 2 | 5 |
| Q9NQX3 | Gephyrin OS=Homo sapiens GN=GPHN PE=1 SV=1 - [GEPH_HUMAN] | 4 | 2 | 5 |
| P53990 | IST1 homolog OS=Homo sapiens GN=KIAA0174 PE=1 SV=1 - [IST1_HUMAN] | 4 | 2 | 5 |
| P62913 | 60S ribosomal protein L11 OS=Homo sapiens GN=RPL11 PE=1 SV=2 - [RL11_HUMAN] | 3 | 2 | 5 |
| Q9BTE6 | Alanyl-tRNA editing protein Aarsd1 OS=Homo sapiens GN=AARSD1 PE=1 SV=2 - [AASD1_HUMAN] | 3 | 2 | 5 |
| Q99653 | Calcium-binding protein p22 OS=Homo sapiens GN=CHP PE=1 SV=3 - [CHP1_HUMAN] | 3 | 2 | 5 |
| P07858 | Cathepsin B OS=Homo sapiens GN=CTSB PE=1 SV=3 - [CATB_HUMAN] | 3 | 2 | 5 |
| Q9UKL0 | REST corepressor 1 OS=Homo sapiens GN=RCOR1 PE=1 SV=1 - [RCOR1_HUMAN] | 3 | 2 | 5 |
| Q15050 | Ribosome biogenesis regulatory protein homolog OS=Homo sapiens GN=RRS1 PE=1 SV=2 - [RRS1_HUMAN] | 3 | 2 | 5 |
| Q9NS69 | Mitochondrial import receptor subunit TOM22 homolog OS=Homo sapiens GN=TOMM22 PE=1 SV=3 - [TOM22_HUMAN] | 2 | 2 | 5 |
| P37268 | Squalene synthase OS=Homo sapiens GN=FDFT1 PE=1 SV=1 - [FDFT_HUMAN] | 2 | 2 | 5 |
| P11172 | Uridine 5'-monophosphate synthase OS=Homo sapiens GN=UMPS PE=1 SV=1 - [UMPS_HUMAN] | 2 | 2 | 5 |
| Q99829 | Copine-1 OS=Homo sapiens GN=CPNE1 PE=1 SV=1 - [CPNE1_HUMAN] | 1 | 2 | 5 |
| Q14166 | Tubulin--tyrosine ligase-like protein 12 OS=Homo sapiens GN=TTLL12 PE=1 SV=2 - [TTL12_HUMAN] | 1 | 2 | 5 |
| P49792 | E3 SUMO-protein ligase RanBP2 OS=Homo sapiens GN=RANBP2 PE=1 SV=2 - [RBP2_HUMAN] | 0 | 2 | 5 |
| O43432 | Eukaryotic translation initiation factor 4 gamma 3 OS=Homo sapiens GN=EIF4G3 PE=1 SV=2 - [IF4G3_HUMAN] | 0 | 2 | 5 |
| Q9BY32 | Inosine triphosphate pyrophosphatase OS=Homo sapiens GN=ITPA PE=1 SV=2 - [ITPA_HUMAN] | 8 | 1 | 5 |
| Q9BSD7 | Nucleoside-triphosphatase C1orf57 OS=Homo sapiens GN=C1orf57 PE=1 SV=1 - [CA057_HUMAN] | 8 | 1 | 5 |
| Q8WZA9 | Immunity-related GTPase family Q protein OS=Homo sapiens GN=IRGQ PE=1 SV=1 - [IRGQ_HUMAN] | 7 | 1 | 5 |
| Q9Y295 | Developmentally-regulated GTP-binding protein 1 OS=Homo sapiens GN=DRG1 PE=1 SV=1 - [DRG1_HUMAN] | 6 | 1 | 5 |
| Q14978 | Nucleolar and coiled-body phosphoprotein 1 OS=Homo sapiens GN=NOLC1 PE=1 SV=2 - [NOLC1_HUMAN] | 6 | 1 | 5 |
| P49721 | Proteasome subunit beta type-2 OS=Homo sapiens GN=PSMB2 PE=1 SV=1 - [PSB2_HUMAN] | 6 | 1 | 5 |
| Q6IAA8 | RhoA activator C11orf59 OS=Homo sapiens GN=C11orf59 PE=1 SV=2 - [CK059_HUMAN] | 6 | 1 | 5 |
| P55010 | Eukaryotic translation initiation factor 5 OS=Homo sapiens GN=EIF5 PE=1 SV=2 - [IF5_HUMAN] | 5 | 1 | 5 |
| P35269 | General transcription factor IIF subunit 1 OS=Homo sapiens GN=GTF2F1 PE=1 SV=2 - [T2FA_HUMAN] | 5 | 1 | 5 |
| Q15435 | Protein phosphatase 1 regulatory subunit 7 OS=Homo sapiens GN=PPP1R7 PE=1 SV=1 - [PP1R7_HUMAN] | 4 | 1 | 5 |
| Q9H2W6 | 39S ribosomal protein L46, mitochondrial OS=Homo sapiens GN=MRPL46 PE=1 SV=1 - [RM46_HUMAN] | 3 | 1 | 5 |
| P45973 | Chromobox protein homolog 5 OS=Homo sapiens GN=CBX5 PE=1 SV=1 - [CBX5_HUMAN] | 3 | 1 | 5 |
| Q9BYT8 | Neurolysin, mitochondrial OS=Homo sapiens GN=NLN PE=1 SV=1 - [NEUL_HUMAN] | 3 | 1 | 5 |
| Q13451 | Peptidyl-prolyl cis-trans isomerase FKBP5 OS=Homo sapiens GN=FKBP5 PE=1 SV=2 - [FKBP5_HUMAN] | 3 | 1 | 5 |
| Q9BVK6 | Transmembrane emp24 domain-containing protein 9 OS=Homo sapiens GN=TMED9 PE=1 SV=2 - [TMED9_HUMAN] | 3 | 1 | 5 |
| P16455 | Methylated-DNA--protein-cysteine methyltransferase OS=Homo sapiens GN=MGMT PE=1 SV=1 - [MGMT_HUMAN] | 2 | 1 | 5 |
| O75475 | PC4 and SFRS1-interacting protein OS=Homo sapiens GN=PSIP1 PE=1 SV=1 - [PSIP1_HUMAN] | 2 | 1 | 5 |
| Q99986 | Serine/threonine-protein kinase VRK1 OS=Homo sapiens GN=VRK1 PE=1 SV=1 - [VRK1_HUMAN] | 1 | 1 | 5 |
| P27635 | 60S ribosomal protein L10 OS=Homo sapiens GN=RPL10 PE=1 SV=4 - [RL10_HUMAN] | 0 | 1 | 5 |
| Q8NHH9 | Atlastin-2 OS=Homo sapiens GN=ATL2 PE=1 SV=2 - [ATLA2_HUMAN] | 0 | 1 | 5 |
| Q5QJE6 | Deoxynucleotidyltransferase terminal-interacting protein 2 OS=Homo sapiens GN=DNTTIP2 PE=1 SV=2 - [TDIF2_HUMAN] | 0 | 1 | 5 |
| P02794 | Ferritin heavy chain OS=Homo sapiens GN=FTH1 PE=1 SV=2 - [FRIH_HUMAN] | 5 | 0 | 5 |
| P41223 | Protein BUD31 homolog OS=Homo sapiens GN=BUD31 PE=1 SV=2 - [BUD31_HUMAN] | 5 | 0 | 5 |
| Q53EL6 | Programmed cell death protein 4 OS=Homo sapiens GN=PDCD4 PE=1 SV=2 - [PDCD4_HUMAN] | 3 | 0 | 5 |
| P23526 | Adenosylhomocysteinase OS=Homo sapiens GN=AHCY PE=1 SV=4 - [SAHH_HUMAN] | 10 | 8 | 4 |
| Q06323 | Proteasome activator complex subunit 1 OS=Homo sapiens GN=PSME1 PE=1 SV=1 - [PSME1_HUMAN] | 11 | 7 | 4 |
| P62873 | Guanine nucleotide-binding protein G(I)/G(S)/G(T) subunit beta-1 OS=Homo sapiens GN=GNB1 PE=1 SV=3 - [GBB1_HUMAN] | 6 | 6 | 4 |
| P0C0S5 | Histone H2A.Z OS=Homo sapiens GN=H2AFZ PE=1 SV=2 - [H2AZ_HUMAN] | 6 | 6 | 4 |
| P17987 | T-complex protein 1 subunit alpha OS=Homo sapiens GN=TCP1 PE=1 SV=1 - [TCPA_HUMAN] | 14 | 5 | 4 |
| O95336 | 6-phosphogluconolactonase OS=Homo sapiens GN=PGLS PE=1 SV=2 - [6PGL_HUMAN] | 8 | 5 | 4 |
| P62424 | 60S ribosomal protein L7a OS=Homo sapiens GN=RPL7A PE=1 SV=2 - [RL7A_HUMAN] | 6 | 5 | 4 |
| Q08752 | Peptidyl-prolyl cis-trans isomerase D OS=Homo sapiens GN=PPID PE=1 SV=3 - [PPID_HUMAN] | 6 | 5 | 4 |
| O43765 | Small glutamine-rich tetratricopeptide repeat-containing protein alpha OS=Homo sapiens GN=SGTA PE=1 SV=1 - [SGTA_HUMAN] | 6 | 5 | 4 |
| P11177 | Pyruvate dehydrogenase E1 component subunit beta, mitochondrial OS=Homo sapiens GN=PDHB PE=1 SV=3 - [ODPB_HUMAN] | 4 | 5 | 4 |
| P13674 | Prolyl 4-hydroxylase subunit alpha-1 OS=Homo sapiens GN=P4HA1 PE=1 SV=2 - [P4HA1_HUMAN] | 3 | 5 | 4 |
| Q16186 | Proteasomal ubiquitin receptor ADRM1 OS=Homo sapiens GN=ADRM1 PE=1 SV=2 - [ADRM1_HUMAN] | 3 | 5 | 4 |
| Q9H9A6 | Leucine-rich repeat-containing protein 40 OS=Homo sapiens GN=LRRC40 PE=1 SV=1 - [LRC40_HUMAN] | 2 | 5 | 4 |
| Q00577 | Transcriptional activator protein Pur-alpha OS=Homo sapiens GN=PURA PE=1 SV=2 - [PURA_HUMAN] | 2 | 5 | 4 |
| P46459 | Vesicle-fusing ATPase OS=Homo sapiens GN=NSF PE=1 SV=3 - [NSF_HUMAN] | 2 | 5 | 4 |
| P09382 | Galectin-1 OS=Homo sapiens GN=LGALS1 PE=1 SV=2 - [LEG1_HUMAN] | 0 | 5 | 4 |
| P62750 | 60S ribosomal protein L23a OS=Homo sapiens GN=RPL23A PE=1 SV=1 - [RL23A_HUMAN] | 10 | 4 | 4 |
| P23381 | Tryptophanyl-tRNA synthetase, cytoplasmic OS=Homo sapiens GN=WARS PE=1 SV=2 - [SYWC_HUMAN] | 8 | 4 | 4 |
| Q9UJS0 | Calcium-binding mitochondrial carrier protein Aralar2 OS=Homo sapiens GN=SLC25A13 PE=1 SV=2 - [CMC2_HUMAN] | 7 | 4 | 4 |
| O15067 | Phosphoribosylformylglycinamidine synthase OS=Homo sapiens GN=PFAS PE=1 SV=4 - [PUR4_HUMAN] | 6 | 4 | 4 |
| O43399 | Tumor protein D54 OS=Homo sapiens GN=TPD52L2 PE=1 SV=2 - [TPD54_HUMAN] | 6 | 4 | 4 |
| P21964 | Catechol O-methyltransferase OS=Homo sapiens GN=COMT PE=1 SV=2 - [COMT_HUMAN] | 5 | 4 | 4 |
| P60981 | Destrin OS=Homo sapiens GN=DSTN PE=1 SV=3 - [DEST_HUMAN] | 5 | 4 | 4 |
| P50402 | Emerin OS=Homo sapiens GN=EMD PE=1 SV=1 - [EMD_HUMAN] | 5 | 4 | 4 |
| P62993 | Growth factor receptor-bound protein 2 OS=Homo sapiens GN=GRB2 PE=1 SV=1 - [GRB2_HUMAN] | 5 | 4 | 4 |
| P37108 | Signal recognition particle 14 kDa protein OS=Homo sapiens GN=SRP14 PE=1 SV=2 - [SRP14_HUMAN] | 5 | 4 | 4 |
| P60891 | Ribose-phosphate pyrophosphokinase 1 OS=Homo sapiens GN=PRPS1 PE=1 SV=2 - [PRPS1_HUMAN] | 4 | 4 | 4 |
| P46777 | 60S ribosomal protein L5 OS=Homo sapiens GN=RPL5 PE=1 SV=3 - [RL5_HUMAN] | 3 | 4 | 4 |
| O94906 | Pre-mRNA-processing factor 6 OS=Homo sapiens GN=PRPF6 PE=1 SV=1 - [PRP6_HUMAN] | 3 | 4 | 4 |
| Q68EM7 | Rho GTPase-activating protein 17 OS=Homo sapiens GN=ARHGAP17 PE=1 SV=1 - [RHG17_HUMAN] | 3 | 4 | 4 |
| P82979 | SAP domain-containing ribonucleoprotein OS=Homo sapiens GN=SARNP PE=1 SV=3 - [SARNP_HUMAN] | 3 | 4 | 4 |
| Q08209 | Serine/threonine-protein phosphatase 2B catalytic subunit alpha isoform OS=Homo sapiens GN=PPP3CA PE=1 SV=1 - [PP2BA_HUMAN] | 3 | 4 | 4 |
| P52888 | Thimet oligopeptidase OS=Homo sapiens GN=THOP1 PE=1 SV=2 - [THOP1_HUMAN] | 3 | 4 | 4 |
| O75351 | Vacuolar protein sorting-associated protein 4B OS=Homo sapiens GN=VPS4B PE=1 SV=2 - [VPS4B_HUMAN] | 3 | 4 | 4 |
| Q9UH62 | Armadillo repeat-containing X-linked protein 3 OS=Homo sapiens GN=ARMCX3 PE=1 SV=1 - [ARMX3_HUMAN] | 2 | 4 | 4 |
| Q96GG9 | DCN1-like protein 1 OS=Homo sapiens GN=DCUN1D1 PE=1 SV=1 - [DCNL1_HUMAN] | 2 | 4 | 4 |
| Q15738 | Sterol-4-alpha-carboxylate 3-dehydrogenase, decarboxylating OS=Homo sapiens GN=NSDHL PE=1 SV=2 - [NSDHL_HUMAN] | 2 | 4 | 4 |
| P52594 | Arf-GAP domain and FG repeats-containing protein 1 OS=Homo sapiens GN=AGFG1 PE=1 SV=2 - [AGFG1_HUMAN] | 1 | 4 | 4 |
| Q9BYG3 | MKI67 FHA domain-interacting nucleolar phosphoprotein OS=Homo sapiens GN=NIFK PE=1 SV=1 - [MK67I_HUMAN] | 1 | 4 | 4 |
| P31153 | S-adenosylmethionine synthetase isoform type-2 OS=Homo sapiens GN=MAT2A PE=1 SV=1 - [METK2_HUMAN] | 1 | 4 | 4 |
| O14639 | Actin-binding LIM protein 1 OS=Homo sapiens GN=ABLIM1 PE=1 SV=3 - [ABLM1_HUMAN] | 0 | 4 | 4 |
| Q86XP3 | ATP-dependent RNA helicase DDX42 OS=Homo sapiens GN=DDX42 PE=1 SV=1 - [DDX42_HUMAN] | 0 | 4 | 4 |
| Q15029 | 116 kDa U5 small nuclear ribonucleoprotein component OS=Homo sapiens GN=EFTUD2 PE=1 SV=1 - [U5S1_HUMAN] | 8 | 3 | 4 |
| P07814 | Bifunctional aminoacyl-tRNA synthetase OS=Homo sapiens GN=EPRS PE=1 SV=5 - [SYEP_HUMAN] | 8 | 3 | 4 |
| P62158 | Calmodulin OS=Homo sapiens GN=CALM1 PE=1 SV=2 - [CALM_HUMAN] | 8 | 3 | 4 |
| Q9Y678 | Coatomer subunit gamma OS=Homo sapiens GN=COPG PE=1 SV=1 - [COPG_HUMAN] | 8 | 3 | 4 |
| Q52LJ0 | Protein FAM98B OS=Homo sapiens GN=FAM98B PE=1 SV=1 - [FA98B_HUMAN] | 8 | 3 | 4 |
| P60866 | 40S ribosomal protein S20 OS=Homo sapiens GN=RPS20 PE=1 SV=1 - [RS20_HUMAN] | 7 | 3 | 4 |
| Q6FI81 | Anamorsin OS=Homo sapiens GN=CIAPIN1 PE=1 SV=2 - [CPIN1_HUMAN] | 7 | 3 | 4 |
| Q9UBS4 | DnaJ homolog subfamily B member 11 OS=Homo sapiens GN=DNAJB11 PE=1 SV=1 - [DJB11_HUMAN] | 7 | 3 | 4 |
| Q9UMS4 | Pre-mRNA-processing factor 19 OS=Homo sapiens GN=PRPF19 PE=1 SV=1 - [PRP19_HUMAN] | 7 | 3 | 4 |
| P22061 | Protein-L-isoaspartate(D-aspartate) O-methyltransferase OS=Homo sapiens GN=PCMT1 PE=1 SV=4 - [PIMT_HUMAN] | 7 | 3 | 4 |
| P24752 | Acetyl-CoA acetyltransferase, mitochondrial OS=Homo sapiens GN=ACAT1 PE=1 SV=1 - [THIL_HUMAN] | 6 | 3 | 4 |
| O43237 | Cytoplasmic dynein 1 light intermediate chain 2 OS=Homo sapiens GN=DYNC1LI2 PE=1 SV=1 - [DC1L2_HUMAN] | 6 | 3 | 4 |
| Q08380 | Galectin-3-binding protein OS=Homo sapiens GN=LGALS3BP PE=1 SV=1 - [LG3BP_HUMAN] | 6 | 3 | 4 |
| P41252 | Isoleucyl-tRNA synthetase, cytoplasmic OS=Homo sapiens GN=IARS PE=1 SV=2 - [SYIC_HUMAN] | 6 | 3 | 4 |
| Q8N8S7 | Protein enabled homolog OS=Homo sapiens GN=ENAH PE=1 SV=2 - [ENAH_HUMAN] | 6 | 3 | 4 |
| P63208 | S-phase kinase-associated protein 1 OS=Homo sapiens GN=SKP1 PE=1 SV=2 - [SKP1_HUMAN] | 6 | 3 | 4 |
| P30085 | UMP-CMP kinase OS=Homo sapiens GN=CMPK1 PE=1 SV=3 - [KCY_HUMAN] | 6 | 3 | 4 |
| P42785 | Lysosomal Pro-X carboxypeptidase OS=Homo sapiens GN=PRCP PE=1 SV=1 - [PCP_HUMAN] | 5 | 3 | 4 |
| Q9UHV9 | Prefoldin subunit 2 OS=Homo sapiens GN=PFDN2 PE=1 SV=1 - [PFD2_HUMAN] | 5 | 3 | 4 |
| P61106 | Ras-related protein Rab-14 OS=Homo sapiens GN=RAB14 PE=1 SV=4 - [RAB14_HUMAN] | 5 | 3 | 4 |
| O14787 | Transportin-2 OS=Homo sapiens GN=TNPO2 PE=1 SV=3 - [TNPO2_HUMAN] | 5 | 3 | 4 |
| Q9NPD8 | Ubiquitin-conjugating enzyme E2 T OS=Homo sapiens GN=UBE2T PE=1 SV=1 - [UBE2T_HUMAN] | 5 | 3 | 4 |
| P62847 | 40S ribosomal protein S24 OS=Homo sapiens GN=RPS24 PE=1 SV=1 - [RS24_HUMAN] | 4 | 3 | 4 |
| P48444 | Coatomer subunit delta OS=Homo sapiens GN=ARCN1 PE=1 SV=1 - [COPD_HUMAN] | 4 | 3 | 4 |
| Q15056 | Eukaryotic translation initiation factor 4H OS=Homo sapiens GN=EIF4H PE=1 SV=5 - [IF4H_HUMAN] | 4 | 3 | 4 |
| P05556 | Integrin beta-1 OS=Homo sapiens GN=ITGB1 PE=1 SV=2 - [ITB1_HUMAN] | 4 | 3 | 4 |
| P15586 | N-acetylglucosamine-6-sulfatase OS=Homo sapiens GN=GNS PE=1 SV=3 - [GNS_HUMAN] | 4 | 3 | 4 |
| Q86U42 | Polyadenylate-binding protein 2 OS=Homo sapiens GN=PABPN1 PE=1 SV=3 - [PABP2_HUMAN] | 4 | 3 | 4 |
| P61758 | Prefoldin subunit 3 OS=Homo sapiens GN=VBP1 PE=1 SV=3 - [PFD3_HUMAN] | 4 | 3 | 4 |
| P35250 | Replication factor C subunit 2 OS=Homo sapiens GN=RFC2 PE=1 SV=3 - [RFC2_HUMAN] | 4 | 3 | 4 |
| Q5T8P6 | RNA-binding protein 26 OS=Homo sapiens GN=RBM26 PE=1 SV=3 - [RBM26_HUMAN] | 4 | 3 | 4 |
| Q9Y5S9 | RNA-binding protein 8A OS=Homo sapiens GN=RBM8A PE=1 SV=1 - [RBM8A_HUMAN] | 4 | 3 | 4 |
| P61086 | Ubiquitin-conjugating enzyme E2 K OS=Homo sapiens GN=UBE2K PE=1 SV=3 - [UBE2K_HUMAN] | 4 | 3 | 4 |
| Q9Y277 | Voltage-dependent anion-selective channel protein 3 OS=Homo sapiens GN=VDAC3 PE=1 SV=1 - [VDAC3_HUMAN] | 4 | 3 | 4 |
| O95831 | Apoptosis-inducing factor 1, mitochondrial OS=Homo sapiens GN=AIFM1 PE=1 SV=1 - [AIFM1_HUMAN] | 3 | 3 | 4 |
| P13995 | Bifunctional methylenetetrahydrofolate dehydrogenase/cyclohydrolase, mitochondrial OS=Homo sapiens GN=MTHFD2 PE=1 SV=2 - [MTDC_HUMAN] | 3 | 3 | 4 |
| Q9Y371 | Endophilin-B1 OS=Homo sapiens GN=SH3GLB1 PE=1 SV=1 - [SHLB1_HUMAN] | 3 | 3 | 4 |
| Q14696 | LDLR chaperone MESD OS=Homo sapiens GN=MESDC2 PE=1 SV=2 - [MESD_HUMAN] | 3 | 3 | 4 |
| Q9BV86 | Methyltransferase-like protein 11A OS=Homo sapiens GN=METTL11A PE=1 SV=3 - [ME11A_HUMAN] | 3 | 3 | 4 |
| Q9BXJ9 | NMDA receptor-regulated protein 1 OS=Homo sapiens GN=NARG1 PE=1 SV=1 - [NARG1_HUMAN] | 3 | 3 | 4 |
| Q9H2J4 | Phosducin-like protein 3 OS=Homo sapiens GN=PDCL3 PE=1 SV=1 - [PDCL3_HUMAN] | 3 | 3 | 4 |
| Q9H074 | Polyadenylate-binding protein-interacting protein 1 OS=Homo sapiens GN=PAIP1 PE=1 SV=1 - [PAIP1_HUMAN] | 3 | 3 | 4 |
| Q9UNH7 | Sorting nexin-6 OS=Homo sapiens GN=SNX6 PE=1 SV=1 - [SNX6_HUMAN] | 3 | 3 | 4 |
| Q96A49 | Synapse-associated protein 1 OS=Homo sapiens GN=SYAP1 PE=1 SV=1 - [SYAP1_HUMAN] | 3 | 3 | 4 |
| Q01130 | Splicing factor, arginine/serine-rich 2 OS=Homo sapiens GN=SFRS2 PE=1 SV=4 - [SFRS2_HUMAN] | 2 | 3 | 4 |
| Q86Y82 | Syntaxin-12 OS=Homo sapiens GN=STX12 PE=1 SV=1 - [STX12_HUMAN] | 2 | 3 | 4 |
| P07305 | Histone H1.0 OS=Homo sapiens GN=H1F0 PE=1 SV=3 - [H10_HUMAN] | 1 | 3 | 4 |
| P23786 | Carnitine O-palmitoyltransferase 2, mitochondrial OS=Homo sapiens GN=CPT2 PE=1 SV=2 - [CPT2_HUMAN] | 0 | 3 | 4 |
| Q8IYB3 | Serine/arginine repetitive matrix protein 1 OS=Homo sapiens GN=SRRM1 PE=1 SV=2 - [SRRM1_HUMAN] | 0 | 3 | 4 |
| P13861 | cAMP-dependent protein kinase type II-alpha regulatory subunit OS=Homo sapiens GN=PRKAR2A PE=1 SV=2 - [KAP2_HUMAN] | 10 | 2 | 4 |
| Q99471 | Prefoldin subunit 5 OS=Homo sapiens GN=PFDN5 PE=1 SV=2 - [PFD5_HUMAN] | 9 | 2 | 4 |
| Q9H9Q2 | COP9 signalosome complex subunit 7b OS=Homo sapiens GN=COPS7B PE=1 SV=1 - [CSN7B_HUMAN] | 8 | 2 | 4 |
| P62917 | 60S ribosomal protein L8 OS=Homo sapiens GN=RPL8 PE=1 SV=2 - [RL8_HUMAN] | 5 | 2 | 4 |
| Q9H4A6 | Golgi phosphoprotein 3 OS=Homo sapiens GN=GOLPH3 PE=1 SV=1 - [GOLP3_HUMAN] | 5 | 2 | 4 |
| P51858 | Hepatoma-derived growth factor OS=Homo sapiens GN=HDGF PE=1 SV=1 - [HDGF_HUMAN] | 5 | 2 | 4 |
| O15347 | High mobility group protein B3 OS=Homo sapiens GN=HMGB3 PE=1 SV=4 - [HMGB3_HUMAN] | 5 | 2 | 4 |
| O15173 | Membrane-associated progesterone receptor component 2 OS=Homo sapiens GN=PGRMC2 PE=1 SV=1 - [PGRC2_HUMAN] | 5 | 2 | 4 |
| P20340 | Ras-related protein Rab-6A OS=Homo sapiens GN=RAB6A PE=1 SV=3 - [RAB6A_HUMAN] | 5 | 2 | 4 |
| Q15427 | Splicing factor 3B subunit 4 OS=Homo sapiens GN=SF3B4 PE=1 SV=1 - [SF3B4_HUMAN] | 5 | 2 | 4 |
| Q16881 | Thioredoxin reductase 1, cytoplasmic OS=Homo sapiens GN=TXNRD1 PE=1 SV=3 - [TRXR1_HUMAN] | 5 | 2 | 4 |
| P31937 | 3-hydroxyisobutyrate dehydrogenase, mitochondrial OS=Homo sapiens GN=HIBADH PE=1 SV=2 - [3HIDH_HUMAN] | 4 | 2 | 4 |
| O96019 | Actin-like protein 6A OS=Homo sapiens GN=ACTL6A PE=1 SV=1 - [ACL6A_HUMAN] | 4 | 2 | 4 |
| Q9NX63 | Coiled-coil-helix-coiled-coil-helix domain-containing protein 3, mitochondrial OS=Homo sapiens GN=CHCHD3 PE=1 SV=1 - [CHCH3_HUMAN] | 4 | 2 | 4 |
| Q9UQ88 | Cyclin-dependent kinase 11A OS=Homo sapiens GN=CDK11A PE=1 SV=4 - [CD11A_HUMAN] | 4 | 2 | 4 |
| P36957 | Dihydrolipoyllysine-residue succinyltransferase component of 2-oxoglutarate dehydrogenase complex, mitochondrial OS=Homo sapiens GN=DLST PE=1 SV=4 - [ODO2_HUMAN] | 4 | 2 | 4 |
| O15371 | Eukaryotic translation initiation factor 3 subunit D OS=Homo sapiens GN=EIF3D PE=1 SV=1 - [EIF3D_HUMAN] | 4 | 2 | 4 |
| P28072 | Proteasome subunit beta type-6 OS=Homo sapiens GN=PSMB6 PE=1 SV=4 - [PSB6_HUMAN] | 4 | 2 | 4 |
| Q9NR45 | Sialic acid synthase OS=Homo sapiens GN=NANS PE=1 SV=2 - [SIAS_HUMAN] | 4 | 2 | 4 |
| P30626 | Sorcin OS=Homo sapiens GN=SRI PE=1 SV=1 - [SORCN_HUMAN] | 4 | 2 | 4 |
| O75494 | Splicing factor, arginine/serine-rich 13A OS=Homo sapiens GN=SFRS13A PE=1 SV=1 - [SF13A_HUMAN] | 4 | 2 | 4 |
| P23919 | Thymidylate kinase OS=Homo sapiens GN=DTYMK PE=1 SV=4 - [KTHY_HUMAN] | 4 | 2 | 4 |
| P36543 | V-type proton ATPase subunit E 1 OS=Homo sapiens GN=ATP6V1E1 PE=1 SV=1 - [VATE1_HUMAN] | 4 | 2 | 4 |
| Q9Y376 | Calcium-binding protein 39 OS=Homo sapiens GN=CAB39 PE=1 SV=1 - [CAB39_HUMAN] | 3 | 2 | 4 |
| Q9NPD3 | Exosome complex exonuclease RRP41 OS=Homo sapiens GN=EXOSC4 PE=1 SV=3 - [EXOS4_HUMAN] | 3 | 2 | 4 |
| P47755 | F-actin-capping protein subunit alpha-2 OS=Homo sapiens GN=CAPZA2 PE=1 SV=3 - [CAZA2_HUMAN] | 3 | 2 | 4 |
| P48637 | Glutathione synthetase OS=Homo sapiens GN=GSS PE=1 SV=1 - [GSHB_HUMAN] | 3 | 2 | 4 |
| P13473 | Lysosome-associated membrane glycoprotein 2 OS=Homo sapiens GN=LAMP2 PE=1 SV=2 - [LAMP2_HUMAN] | 3 | 2 | 4 |
| Q9BRP8 | Partner of Y14 and mago OS=Homo sapiens GN=PYM1 PE=1 SV=1 - [PYM1_HUMAN] | 3 | 2 | 4 |
| P40938 | Replication factor C subunit 3 OS=Homo sapiens GN=RFC3 PE=1 SV=2 - [RFC3_HUMAN] | 3 | 2 | 4 |
| O43681 | ATPase ASNA1 OS=Homo sapiens GN=ASNA1 PE=1 SV=2 - [ASNA_HUMAN] | 2 | 2 | 4 |
| Q13895 | Bystin OS=Homo sapiens GN=BYSL PE=1 SV=3 - [BYST_HUMAN] | 2 | 2 | 4 |
| P30042 | ES1 protein homolog, mitochondrial OS=Homo sapiens GN=C21orf33 PE=1 SV=3 - [ES1_HUMAN] | 2 | 2 | 4 |
| P14324 | Farnesyl pyrophosphate synthase OS=Homo sapiens GN=FDPS PE=1 SV=4 - [FPPS_HUMAN] | 2 | 2 | 4 |
| P49790 | Nuclear pore complex protein Nup153 OS=Homo sapiens GN=NUP153 PE=1 SV=2 - [NU153_HUMAN] | 2 | 2 | 4 |
| O15305 | Phosphomannomutase 2 OS=Homo sapiens GN=PMM2 PE=1 SV=1 - [PMM2_HUMAN] | 2 | 2 | 4 |
| Q8NBJ5 | Procollagen galactosyltransferase 1 OS=Homo sapiens GN=GLT25D1 PE=1 SV=1 - [GT251_HUMAN] | 2 | 2 | 4 |
| Q14160 | Protein scribble homolog OS=Homo sapiens GN=SCRIB PE=1 SV=4 - [SCRIB_HUMAN] | 2 | 2 | 4 |
| Q9NW13 | RNA-binding protein 28 OS=Homo sapiens GN=RBM28 PE=1 SV=3 - [RBM28_HUMAN] | 2 | 2 | 4 |
| P54725 | UV excision repair protein RAD23 homolog A OS=Homo sapiens GN=RAD23A PE=1 SV=1 - [RD23A_HUMAN] | 2 | 2 | 4 |
| P01023 | Alpha-2-macroglobulin OS=Homo sapiens GN=A2M PE=1 SV=3 - [A2MG_HUMAN] | 1 | 2 | 4 |
| Q9UQB8 | Brain-specific angiogenesis inhibitor 1-associated protein 2 OS=Homo sapiens GN=BAIAP2 PE=1 SV=1 - [BAIP2_HUMAN] | 1 | 2 | 4 |
| Q96DG6 | Carboxymethylenebutenolidase homolog OS=Homo sapiens GN=CMBL PE=1 SV=1 - [CMBL_HUMAN] | 0 | 2 | 4 |
| Q15075 | Early endosome antigen 1 OS=Homo sapiens GN=EEA1 PE=1 SV=2 - [EEA1_HUMAN] | 0 | 2 | 4 |
| Q969X5 | Endoplasmic reticulum-Golgi intermediate compartment protein 1 OS=Homo sapiens GN=ERGIC1 PE=1 SV=1 - [ERGI1_HUMAN] | 0 | 2 | 4 |
| O00461 | Golgi integral membrane protein 4 OS=Homo sapiens GN=GOLIM4 PE=1 SV=1 - [GOLI4_HUMAN] | 0 | 2 | 4 |
| Q9BWU0 | Kanadaptin OS=Homo sapiens GN=SLC4A1AP PE=1 SV=1 - [NADAP_HUMAN] | 0 | 2 | 4 |
| P07942 | Laminin subunit beta-1 OS=Homo sapiens GN=LAMB1 PE=1 SV=2 - [LAMB1_HUMAN] | 0 | 2 | 4 |
| Q8NCA5 | Protein FAM98A OS=Homo sapiens GN=FAM98A PE=1 SV=1 - [FA98A_HUMAN] | 0 | 2 | 4 |
| Q8ND56 | Protein LSM14 homolog A OS=Homo sapiens GN=LSM14A PE=1 SV=3 - [LS14A_HUMAN] | 0 | 2 | 4 |
| Q86TG7 | Retrotransposon-derived protein PEG10 OS=Homo sapiens GN=PEG10 PE=1 SV=2 - [PEG10_HUMAN] | 0 | 2 | 4 |
| Q9UI10 | Translation initiation factor eIF-2B subunit delta OS=Homo sapiens GN=EIF2B4 PE=1 SV=2 - [EI2BD_HUMAN] | 0 | 2 | 4 |
| Q16850 | Lanosterol 14-alpha demethylase OS=Homo sapiens GN=CYP51A1 PE=1 SV=3 - [CP51A_HUMAN] | 8 | 1 | 4 |
| P63279 | SUMO-conjugating enzyme UBC9 OS=Homo sapiens GN=UBE2I PE=1 SV=1 - [UBC9_HUMAN] | 7 | 1 | 4 |
| P04179 | Superoxide dismutase [Mn], mitochondrial OS=Homo sapiens GN=SOD2 PE=1 SV=2 - [SODM_HUMAN] | 7 | 1 | 4 |
| P23258 | Tubulin gamma-1 chain OS=Homo sapiens GN=TUBG1 PE=1 SV=2 - [TBG1_HUMAN] | 7 | 1 | 4 |
| P55957 | BH3-interacting domain death agonist OS=Homo sapiens GN=BID PE=1 SV=1 - [BID_HUMAN] | 6 | 1 | 4 |
| Q96JB5 | CDK5 regulatory subunit-associated protein 3 OS=Homo sapiens GN=CDK5RAP3 PE=1 SV=2 - [CK5P3_HUMAN] | 6 | 1 | 4 |
| P06730 | Eukaryotic translation initiation factor 4E OS=Homo sapiens GN=EIF4E PE=1 SV=2 - [IF4E_HUMAN] | 6 | 1 | 4 |
| P28074 | Proteasome subunit beta type-5 OS=Homo sapiens GN=PSMB5 PE=1 SV=3 - [PSB5_HUMAN] | 6 | 1 | 4 |
| P13693 | Translationally-controlled tumor protein OS=Homo sapiens GN=TPT1 PE=1 SV=1 - [TCTP_HUMAN] | 6 | 1 | 4 |
| Q15813 | Tubulin-specific chaperone E OS=Homo sapiens GN=TBCE PE=1 SV=1 - [TBCE_HUMAN] | 5 | 1 | 4 |
| Q07020 | 60S ribosomal protein L18 OS=Homo sapiens GN=RPL18 PE=1 SV=2 - [RL18_HUMAN] | 4 | 1 | 4 |
| Q15021 | Condensin complex subunit 1 OS=Homo sapiens GN=NCAPD2 PE=1 SV=3 - [CND1_HUMAN] | 4 | 1 | 4 |
| O95793 | Double-stranded RNA-binding protein Staufen homolog 1 OS=Homo sapiens GN=STAU1 PE=1 SV=2 - [STAU1_HUMAN] | 4 | 1 | 4 |
| P82930 | 28S ribosomal protein S34, mitochondrial OS=Homo sapiens GN=MRPS34 PE=1 SV=2 - [RT34_HUMAN] | 3 | 1 | 4 |
| P54920 | Alpha-soluble NSF attachment protein OS=Homo sapiens GN=NAPA PE=1 SV=3 - [SNAA_HUMAN] | 3 | 1 | 4 |
| Q8WYA6 | Beta-catenin-like protein 1 OS=Homo sapiens GN=CTNNBL1 PE=1 SV=1 - [CTBL1_HUMAN] | 3 | 1 | 4 |
| O75439 | Mitochondrial-processing peptidase subunit beta OS=Homo sapiens GN=PMPCB PE=1 SV=2 - [MPPB_HUMAN] | 3 | 1 | 4 |
| Q01844 | RNA-binding protein EWS OS=Homo sapiens GN=EWSR1 PE=1 SV=1 - [EWS_HUMAN] | 3 | 1 | 4 |
| Q14677 | Clathrin interactor 1 OS=Homo sapiens GN=CLINT1 PE=1 SV=1 - [EPN4_HUMAN] | 2 | 1 | 4 |
| Q99627 | COP9 signalosome complex subunit 8 OS=Homo sapiens GN=COPS8 PE=1 SV=1 - [CSN8_HUMAN] | 2 | 1 | 4 |
| O00567 | Nucleolar protein 56 OS=Homo sapiens GN=NOP56 PE=1 SV=4 - [NOP56_HUMAN] | 2 | 1 | 4 |
| Q3MHD2 | Protein LSM12 homolog OS=Homo sapiens GN=LSM12 PE=1 SV=2 - [LSM12_HUMAN] | 2 | 1 | 4 |
| Q96DH6 | RNA-binding protein Musashi homolog 2 OS=Homo sapiens GN=MSI2 PE=1 SV=1 - [MSI2H_HUMAN] | 2 | 1 | 4 |
| Q9Y5X3 | Sorting nexin-5 OS=Homo sapiens GN=SNX5 PE=1 SV=1 - [SNX5_HUMAN] | 2 | 1 | 4 |
| P53701 | Cytochrome c-type heme lyase OS=Homo sapiens GN=HCCS PE=1 SV=1 - [CCHL_HUMAN] | 1 | 1 | 4 |
| Q7Z5L9 | Interferon regulatory factor 2-binding protein 2 OS=Homo sapiens GN=IRF2BP2 PE=1 SV=2 - [I2BP2_HUMAN] | 1 | 1 | 4 |
| Q96GM5 | SWI/SNF-related matrix-associated actin-dependent regulator of chromatin subfamily D member 1 OS=Homo sapiens GN=SMARCD1 PE=1 SV=2 - [SMRD1_HUMAN] | 1 | 1 | 4 |
| P51571 | Translocon-associated protein subunit delta OS=Homo sapiens GN=SSR4 PE=1 SV=1 - [SSRD_HUMAN] | 1 | 1 | 4 |
| P40222 | Alpha-taxilin OS=Homo sapiens GN=TXLNA PE=1 SV=3 - [TXLNA_HUMAN] | 0 | 1 | 4 |
| P12109 | Collagen alpha-1(VI) chain OS=Homo sapiens GN=COL6A1 PE=1 SV=3 - [CO6A1_HUMAN] | 0 | 1 | 4 |
| Q16836 | Hydroxyacyl-coenzyme A dehydrogenase, mitochondrial OS=Homo sapiens GN=HADH PE=1 SV=3 - [HCDH_HUMAN] | 0 | 1 | 4 |
| O43663 | Protein regulator of cytokinesis 1 OS=Homo sapiens GN=PRC1 PE=1 SV=2 - [PRC1_HUMAN] | 0 | 1 | 4 |
| O95218 | Zinc finger Ran-binding domain-containing protein 2 OS=Homo sapiens GN=ZRANB2 PE=1 SV=2 - [ZRAB2_HUMAN] | 0 | 1 | 4 |
| Q9Y3Z3 | SAM domain and HD domain-containing protein 1 OS=Homo sapiens GN=SAMHD1 PE=1 SV=2 - [SAMH1_HUMAN] | 9 | 0 | 4 |
| P20618 | Proteasome subunit beta type-1 OS=Homo sapiens GN=PSMB1 PE=1 SV=2 - [PSB1_HUMAN] | 7 | 0 | 4 |
| Q68E01 | Integrator complex subunit 3 OS=Homo sapiens GN=INTS3 PE=1 SV=1 - [INT3_HUMAN] | 5 | 0 | 4 |
| P61923 | Coatomer subunit zeta-1 OS=Homo sapiens GN=COPZ1 PE=1 SV=1 - [COPZ1_HUMAN] | 4 | 0 | 4 |
| Q9Y3D9 | 28S ribosomal protein S23, mitochondrial OS=Homo sapiens GN=MRPS23 PE=1 SV=2 - [RT23_HUMAN] | 3 | 0 | 4 |
| Q9Y6G9 | Cytoplasmic dynein 1 light intermediate chain 1 OS=Homo sapiens GN=DYNC1LI1 PE=1 SV=3 - [DC1L1_HUMAN] | 3 | 0 | 4 |
| P11802 | Cell division protein kinase 4 OS=Homo sapiens GN=CDK4 PE=1 SV=2 - [CDK4_HUMAN] | 2 | 0 | 4 |
| P00374 | Dihydrofolate reductase OS=Homo sapiens GN=DHFR PE=1 SV=2 - [DYR_HUMAN] | 2 | 0 | 4 |
| Q8IVD9 | NudC domain-containing protein 3 OS=Homo sapiens GN=NUDCD3 PE=1 SV=3 - [NUDC3_HUMAN] | 2 | 0 | 4 |
| Q9H6T3 | RNA polymerase II-associated protein 3 OS=Homo sapiens GN=RPAP3 PE=1 SV=2 - [RPAP3_HUMAN] | 2 | 0 | 4 |
| Q13442 | 28 kDa heat- and acid-stable phosphoprotein OS=Homo sapiens GN=PDAP1 PE=1 SV=1 - [HAP28_HUMAN] | 1 | 0 | 4 |
| P30519 | Heme oxygenase 2 OS=Homo sapiens GN=HMOX2 PE=1 SV=2 - [HMOX2_HUMAN] | 1 | 0 | 4 |
| Q15904 | V-type proton ATPase subunit S1 OS=Homo sapiens GN=ATP6AP1 PE=1 SV=2 - [VAS1_HUMAN] | 1 | 0 | 4 |
| P15170 | Eukaryotic peptide chain release factor GTP-binding subunit ERF3A OS=Homo sapiens GN=GSPT1 PE=1 SV=1 - [ERF3A_HUMAN] | 0 | 0 | 4 |
| Q9H307 | Pinin OS=Homo sapiens GN=PNN PE=1 SV=4 - [PININ_HUMAN] | 0 | 0 | 4 |
| Q8IVL6 | Prolyl 3-hydroxylase 3 OS=Homo sapiens GN=LEPREL2 PE=2 SV=1 - [P3H3_HUMAN] | 0 | 0 | 4 |
| Q99613 | Eukaryotic translation initiation factor 3 subunit C OS=Homo sapiens GN=EIF3C PE=1 SV=1 - [EIF3C_HUMAN] | 4 | 8 | 3 |
| Q96T88 | E3 ubiquitin-protein ligase UHRF1 OS=Homo sapiens GN=UHRF1 PE=1 SV=1 - [UHRF1_HUMAN] | 1 | 6 | 3 |
| Q13098 | COP9 signalosome complex subunit 1 OS=Homo sapiens GN=GPS1 PE=1 SV=4 - [CSN1_HUMAN] | 0 | 6 | 3 |
| Q14108 | Lysosome membrane protein 2 OS=Homo sapiens GN=SCARB2 PE=1 SV=2 - [SCRB2_HUMAN] | 8 | 5 | 3 |
| Q15370 | Transcription elongation factor B polypeptide 2 OS=Homo sapiens GN=TCEB2 PE=1 SV=1 - [ELOB_HUMAN] | 5 | 5 | 3 |
| Q8WVM8 | Sec1 family domain-containing protein 1 OS=Homo sapiens GN=SCFD1 PE=1 SV=4 - [SCFD1_HUMAN] | 2 | 5 | 3 |
| O75347 | Tubulin-specific chaperone A OS=Homo sapiens GN=TBCA PE=1 SV=3 - [TBCA_HUMAN] | 8 | 4 | 3 |
| Q16718 | NADH dehydrogenase [ubiquinone] 1 alpha subcomplex subunit 5 OS=Homo sapiens GN=NDUFA5 PE=1 SV=3 - [NDUA5_HUMAN] | 7 | 4 | 3 |
| P63220 | 40S ribosomal protein S21 OS=Homo sapiens GN=RPS21 PE=1 SV=1 - [RS21_HUMAN] | 5 | 4 | 3 |
| P05198 | Eukaryotic translation initiation factor 2 subunit 1 OS=Homo sapiens GN=EIF2S1 PE=1 SV=3 - [IF2A_HUMAN] | 5 | 4 | 3 |
| P69905 | Hemoglobin subunit alpha OS=Homo sapiens GN=HBA1 PE=1 SV=2 - [HBA_HUMAN] | 5 | 4 | 3 |
| O60664 | Perilipin-3 OS=Homo sapiens GN=PLIN3 PE=1 SV=3 - [PLIN3_HUMAN] | 5 | 4 | 3 |
| P62888 | 60S ribosomal protein L30 OS=Homo sapiens GN=RPL30 PE=1 SV=2 - [RL30_HUMAN] | 4 | 4 | 3 |
[truncated: 123,202 more chars]
